# Construction and validation of a necroptosis-related lncRNA signature for predicting the prognosis of gastrointestinal cancer patients

**DOI:** 10.3389/fimmu.2025.1591252

**Published:** 2025-08-14

**Authors:** Weicheng Huang, Yuchen Liu, Ruyi Liu, Chi Feng, Jiehua Wu, Xing Sun, Pengfei Zhu, Pengxiang Chen, Yufeng Cheng

**Affiliations:** ^1^ Department of Radiation Oncology, Qilu Hospital of Shandong University, Jinan, China; ^2^ Neutron Medical Center, Qilu Hospital of Shandong University, Jinan, China; ^3^ Shandong Provincial Key Laboratory of Malignant Tumor Precision Treatment, Qilu Hospital of Shandong University, Jinan, China; ^4^ Shandong Provincial Engineering Research Center for Tumor Precision Treatment, Qilu Hospital of Shandong University, Jinan, China

**Keywords:** gastrointestinal cancers, necroptosis, prognostic prediction, immunotherapy, tumor immune microenvironment

## Abstract

**Background:**

Given the high incidence and mortality rates of gastrointestinal (GI) cancer, along with the lack of effective prognostic markers, this study aimed to construct a prognostic signature to identify high-risk patients facilitate precision medicine, and ultimately improve patient outcomes.

**Methods:**

We analyzed transcriptomic data for COAD, ESCA, READ, and STAD from the TCGA and GTEx databases. Using co-expression analysis, Cox regression, and least absolute shrinkage and selection operator (LASSO) regression, we developed a necroptosis-related lncRNA signature, termed the Necro-lnc score. The predictive performance of the score was validated and assessed through survival analysis, receiver operating characteristic (ROC) analysis, and Cox regression analysis. Additionally, we conducted gene set enrichment analysis (GSEA), immune landscape profiling, and drug sensitivity prediction based on half-maximal inhibitory concentration (IC50) values. The robustness of the score was further supported by cluster analysis, and the biological functions of the selected lncRNAs were experimentally validated through phenotypic assays.

**Results:**

We constructed a prognostic signature comprising five necroptosis-related lncRNAs, referred to as the Necro-lnc score. Calibration plots and areas under the ROC curves (AUCs) confirmed the strong prognostic predictive capability of the score. Kaplan-Meier (K-M) survival curves revealed a significant correlation between the Necro-lnc score and patient outcomes, with high-score patients exhibiting markedly poorer prognoses. Immune landscape and drug susceptibility analyses indicated that the high-score group was characterized by hot tumors and showed greater sensitivity to immunotherapeutic drugs and targeted drugs, while the low-score group associated with cold tumors, was more responsive to chemotherapeutic agents. Furthermore, *in vitro* phenotypic assays demonstrated that the lncRNAs included in the Necro-lnc score play critical roles in the progression and metastasis of GI cancer.

**Conclusion:**

This study developed the promising Necro-lnc score, which demonstrates potential for predicting prognosis and distinguishing between cold and hot tumors, thereby improving personalized treatment strategies for patients with GI cancer.

## 1 Introduction

Gastrointestinal (GI) cancers are the most common malignancies worldwide (1). Among them, stomach, rectal, colon and esophageal cancers are the top 10 most common cancers and the top 10 leading causes of cancer-related death (2). Similar to other types of cancer, the most conventional cancer treatments, including surgery, chemotherapy and radiotherapy, play important roles in treating GI cancer. Although traditional therapies can prolong the survival of patients with GI cancer, many problems remain that cannot be ignored (3). Surgery and radiotherapy can significantly shrink the tumor (4–6). However, these treatments cannot prevent recurrence, and the high rate of postoperative recurrence leads to a low five-year survival rate (7). Therefore, the purpose of this study was to identify prognostic markers of GI cancer and to construct a prognostic signature to screen high-risk patients, which can facilitate personalized therapeutic strategies and contribute to improved survival outcomes in these patients.

Necroptosis, a newly discovered mechanism of cell death, is mediated by RIP1, RIP3, and MLKL (8). As most tumors are resistant to innate apoptosis, the importance of necroptosis in promising treatment strategies has gradually been recognized as promising therapeutic target (9). Necroptosis has the characteristics of both necrosis and apoptosis, which can trigger and accelerate antitumor immunity during immunotherapy for malignancy (10). Recent studies have shown that genes involved in the necroptosis pathway suppress many cancers, including colon cancer, esophageal cancer, and gastric cancer (11–14). Moreover, downregulation of the expression of various key molecules in the necroptosis pathway has been detected in numerous types of cancer cells, suggesting that cancer cells may escape necroptosis to survive (10). Therefore, we wanted to explore whether necroptosis is involved in the development and progression of GI cancer and to construct a signature associated with necroptosis.

LncRNAs are RNA molecules with a transcript length of more than 200 nt and no protein-coding potential. Recent research has shown that lncRNAs play vital roles in human tumorigenesis and progression by serving as tumor oncogenes or suppressors (15). Emerging studies have indicated that lncRNAs are useful for evaluating patient prognosis and assessing the effects of immunotherapy (16, 17). At present, many studies have investigated that lncRNAs have been implicated in various modes of regulated cell death in GI cancers, encompassing necroptosis, anoikis, and cuproptosis (18, 19). Furthermore, a variety of studies have effectively predicted the tumor prognosis using lncRNA signatures (20–22). Moreover, necroptosis-related lncRNAs can be used to evaluate the prognosis of gastric cancer and colon cancer patients (23, 24), which suggests that necroptosis-related lncRNAs may play nonnegligible roles in predicting the prognosis of GI cancers. Therefore, a complete and meaningful lncRNA analysis based on GI cancer gene samples from The Cancer Genome Atlas (TCGA) can provide insights into this field.

Since its introduction in the 19th century, immunotherapy has revolutionized cancer research and treatment (25). Despite the positive effects of immunotherapy, some problems, such as a low remission rate and a lack of effective markers, still exist (26, 27). In solid tumors, responsiveness to immune checkpoint inhibitors (ICIs) is closely associated with distinct immunophenotypes: the “hot” (immune-inflamed) phenotype, characterized by abundant tumor-infiltrating lymphocytes and elevated PD-L1 expression, and the “cold” (immune-desert) phenotype, marked by minimal immune infiltration and an immunosuppressive microenvironment (28). This classification fundamentally linked to the tumor microenvironment, enables the prognostic evaluation of immunotherapy response based on differential patterns of immune cell infiltration (29). Distinguishing between hot and cold tumors and promoting the transformation of cold tumors into hot tumors will improve the antitumor effects of immunotherapy. This finding has major implications for immunotherapy. However, we still lack a simple and effective method for distinguishing between hot and cold tumors (30). As mentioned above, necroptosis activates the immune system, and at the same time, lncRNAs have been extensively evaluated as new cancer biomarkers. Therefore, we aimed to regroup patients with GI cancer based on necroptosis-related lncRNAs and effectively identify hot tumors to improve the efficacy of immunotherapy in clinical practice (10, 31, 32).

The purpose of this study was to develop a prognostic prediction signature for GI cancer. The prognosis of patients can be predicted using this signature. We can stratify patients into high- and low-risk groups and provide appropriate treatment (such as immunotherapy) to patients in the high-risk group to improve their prognosis. In this study, we investigated the prognostic and therapeutic roles of necroptosis-related lncRNAs in GI cancer. Univariate Cox proportional hazard regression analysis was used to screen 5 necroptosis-related lncRNAs and construct a prognostic risk signature; furthermore, we validated the performance of the risk signature in predicting the prognosis and identifying hot and cold tumors.

## 2 Materials and methods

### 2.1 Acquisition of information from gastrointestinal cancer patients

Synthetic data matrices related to tumor and normal tissues of colon adenocarcinoma (COAD), esophageal carcinoma (ESCA), rectum adenocarcinoma (READ) and stomach adenocarcinoma (STAD) were obtained by downloading RNA transcriptome datasets (HTSeq–Counts and HTSeq–FPKM) and relevant clinical information from TCGA (https://portal.gdc.cancer.gov/). Then, we obtained the FPKM synthetic data matrix. The FPKM value matrix was used for both identifying differentially expressed lncRNAs and performing the other analyses. With respect to relevant clinical information, we retrieved data from 1006 patients and then used the Strawberry Perl (version 5.30), R (version 4.2.2) and Caret (version 6.0.93) R packages to randomly divide them into a training risk group and a test risk group. The ratio between the two groups was 1:1.

### 2.2 Selection of necroptosis-related genes and lncRNAs

The necroptosis gene set M24779.gmt, which contains eight necroptosis genes, was downloaded from the Gene Set Enrichment Analysis (GSEA) database (http://www.gsea-msigdb.org/gsea/index.jsp). Additionally, the eight necroptosis genes were included in a profile of 67 necroptosis-related genes identified in previous studies of necroptosis, and in our study, we selected these 67 necroptosis-related genes for a necroptosis gene profile (33) (**Supplementary Table S3**). A correlation analysis was performed between the 67 necroptosis-related genes and differentially expressed lncRNAs in the combined matrices. A total of 3,429 lncRNAs correlated with necroptosis-related genes with Pearson’s correlation coefficients > 0.4, and p < 0.001 were considered necroptosis-related lncRNAs. Finally, we identified 310 differentially expressed lncRNAs (Log2 fold change (FC) > 2, false discovery rate (FDR) < 0.01, and p < 0.01) after screening the synthetic data matrix using the Strawberry Perl and limma (version 3.52.4) R packages.

### 2.3 Establishment and validation of the risk signature

According to the clinical data of gastrointestinal cancer patients in TCGA, we performed a univariate Cox (uni-Cox) proportional hazard regression analysis to screen for lncRNAs related to survival among the necroptosis-related lncRNAs (p < 0.05). Then, least absolute shrinkage and selection operator (LASSO) regression was performed with 10-fold cross-validation and a p value of 0.05, and we ran it for 1,000 cycles. A random stimulation was set up 1,000 times for each cycle to prevent overfitting. We then established a signature called the Necro-lnc score. The 1-, 2-, and 3-year time-dependent receiver operating characteristic (ROC) curves of the signature were plotted via the calculation procedure. We calculated the risk score via the following formula.


$$Necro-lnc\;score=\sum_{k=1}^{n}\left(coef\left(lncRNA^{k}\right)*expr\left(lncRNA^{k}\right)\right)$$

where coef ( $lncRNA^{k}$ ) represents the coefficient of lncRNAs correlated with survival, and expr ( $lncRNA^{k}$ ) represents the expression of lncRNAs. The low- and high-score groups were distinguished based on the median risk score (34, 35). In addition, in the validation phase, the criteria for regrouping each tumor type were based on cutoff values, which were based on the highest “Youden’s index”.

### 2.4 Independent factors and ROC curves for survival

We developed uni-Cox and multivariate Cox (multi-Cox) regression analyses to evaluate whether the risk score and clinical characteristics were independent variables and constructed ROC curves to compare the abilities of different factors to predict survival.

### 2.5 Nomogram and calibration

With the rms (version 6.4.0) R package, we used the risk score, age, sex, tumor stage, T stage, M stage and N stage to establish a nomogram for 1-, 2-, and 3-year overall survival (OS). In addition, correction curves based on the Hosmer–Lemeshow test were also plotted to illustrate whether the predicted outcome was highly consistent with the practical outcome.

### 2.6 Gene set enrichment analysis

With a curated gene set (kegg.v7.4.symbols.gmt), GSEA software (https://www.gsea-msigdb.org/gsea/login.jsp) was applied to identify the significantly enriched pathways between the low- and high-score groups. We selected the 15 pathways with the most significant enrichment in the low- and high-score groups and then plotted the top 5 pathways in the figure.

### 2.7 Investigation of the immune landscape

According to the GSEA results, we analyzed immune cell infiltration in the risk groups. We calculated the immune infiltration status of gastrointestinal cancer patients from TCGA using TIMER, CIBERSORT, XCELL, QUANTISEQ, MCPcounter, EPIC, and CIBERSORT-ABS with TIMER 2.0 (http://timer.cistrome.org/). Alternatively, we downloaded the profile of infiltration estimates for all TCGA tumors using the same website. The Wilcoxon signed-rank test, limma, scales (version 1.2.1), ggplot2 (version 3.4.0), and ggtext (version 0.1.2) R packages were used to analyze the differences in infiltrating immune cell contents, and the results are shown in a bubble chart. In addition, we used the ggpubr (version 0.5.0) R package to compare tumor immune microenvironment (TIME) scores and immune checkpoint activation between the low- and high-score groups.

### 2.8 Drug sensitivity analysis

We used the R package pRRophetic (version 0.5) to evaluate the drug sensitivity of GI cancer patients, as determined by the half-maximal inhibitory concentration (IC50) of each gastrointestinal cancer patient, on the Genomics of Drug Sensitivity in Cancer (GDSC) website (https://www.cancerrxgene.org/) (36).

### 2.9 Cluster analysis of the 5 necroptosis-related lncRNAs

We explored potential molecular subgroups using the ConsensusClusterPlus (CC) (version 1.72.0) R package based on the prognostic lncRNA expression levels to assess the responses of patients with GI cancer to immunotherapy (37). principal component analysis (PCA), t-SNE, and Kaplan–Meier survival analyses were performed using the Rtsne R (version 0.17) package. In addition, we analyzed immunity and compared drug sensitivity using the gene set variation analysis (GSVA) base and pRRophetic R packages.

### 2.10 Cell culture and transfection

KYSE150, HGC27 and CACO2 cells were obtained from the China Center for Type Culture Collection (CCTCC, Wuhan, China). CACO2 cells were cultured in DMEM (Gibco, Life Technologies Inc., Grand Island, NY, USA), and the other cells were cultured in RPMI 1640 media (Gibco). Fetal bovine serum (FBS, Gibco, Brazil) and 1% antibiotics (penicillin G and streptomycin, Solarbio, Beijing, China) were added to the medium. All the cells were cultured at 37°C in a 5% CO2 incubator and plated in 25 cm2 culture flasks. Based on the Necro-lnc score constructed in the previous analysis, LINC02106 and AC026471.3 were selected as the two lncRNAs with the most significant regression coefficients in promoting and suppressing cancer, respectively.

The transient AC026471.3 overexpression plasmid, which contains ENST00000565137.1 in pcDNA3.1, was constructed by Jinan Boshang Biotechnology Co., Ltd. (Jinan, China), with an empty plasmid used as a negative control. Small interfering RNA (siRNA) oligonucleotides targeting LINC02106 and its corresponding control oligonucleotides were designed and synthesized by GenePharma (Shanghai, China). Plasmids and siRNAs were transfected with jetPRIME (Polyplus Transfection, France). The sequences of the siRNAs are listed in **Supplementary Table S1**.

AC026471.3-overexpressing cell lines were constructed by transiently transfecting plasmids. LINC02106-knockdown cell lines were constructed by transfecting small interfering RNAs. Cell lines with transient AC026471.3 overexpression and LINC02106 knockdown were established in KYSE150, HGC27 and CACO2 cells.

### 2.11 Cell proliferation and colony formation assays

The cells were seeded in triplicate in 96-well plates (3 × 103 cells/well). CCK-8 kits (Bioss, Beijing, China) were used to measure the cell absorbance at 48 h, 72 h and 96 h after plating according to the manufacturer’s protocol. The absorbance value of the CCK-8 assay at 450 nm was measured with a spectrophotometer (Tecan, Männedorf, Switzerland).

For the colony formation assay, single suspended cells were seeded in six-well plates (2.0 × 103 cells per well) in triplicate. Colonies were stained with crystal violet and counted. The KYSE150 cells were counted on Day 10, the HGC27 cells were counted on Day 7, and the CACO2 cells were counted on Day 12.

### 2.12 Transwell assays

Approximately 200 µL of serum-free medium containing cells was loaded into the upper chamber of a 24-well Transwell system (Corning, New York, USA), and then 800 µL of medium supplemented with 20% FBS (Gibco, Brazil) was loaded into the lower chambers, in triplicate. After 24 h, the migrated cells were stained with crystal violet and counted under a microscope (Olympus BX51, Tokyo, Japan).

### 2.13 Quantitative real-time PCR

Total RNA was extracted from the cell lines with InvitrogenTM TRIzol reagent (Thermo Fisher Scientific, Waltham, MA, USA). A SureScript First-Strand cDNA Synthesis Kit (GeneCopoeia, Rockville, MD, USA) was used for cDNA synthesis, and for qPCR, a BlazeTaq SYBR Green qPCR mix 2.0 Kit (GeneCopoeia, Rockville, MD, USA) was used. All reverse transcription and qRT–PCR experiments were performed in triplicate according to the manufacturer’s instructions. β-Actin was used as an internal control. The primers for AC026471.3, LINC02106 and β-actin were synthesized by GeneCopoeia (Rockville, MD, USA) and TaKaRa (Dalian, China). The primer sequences are listed in **Supplementary Table S2**. The results were analyzed by the relative 2^-ΔΔCt^ method.

### 2.14 Western blot and antibodies

RIPA buffer (Solarbio, Beijing, China) supplemented with PMSF was used to lyse the cells on ice. Protein concentrations were measured with a BCA protein assay kit (Beyotime, Shanghai, China). After being separated on SDS–polyacrylamide gels, the proteins were transferred onto polyvinylidene fluoride (PVDF) membranes (Millipore, MA, USA) and visualized via autoradiography after Western blotting. The primary antibodies used were as follows: p-RIP1 antibody at a 1:1000 dilution (HUABIO, Hangzhou, China), p-RIP3 antibody at a 1:1000 dilution (HUABIO, Hangzhou, China), p-MLKL antibody at a 1:1000 dilution (HUABIO, Hangzhou, China), and β-actin antibody at a 1:2000 dilution (Proteintech, Wuhan, China). The goat-anti-rabbit secondary antibody was used at a dilution of 1:5000 (ZSGB-BIO, Beijing, China).

### 2.15 Statistical analysis

Statistical analyses were performed using R (version 4.2.2), GraphPad Prism (version 8.0), and SPSS (version 23.0). Comparisons between two independent groups were conducted using the Wilcoxon test. Parametric data were analyzed using the Student’s t-test or one-way ANOVA, while the Kruskal-Wallis test was applied for non-parametric comparisons among multiple groups. A *p*-value < 0.05 was considered statistically significant.

## 3 Results

### 3.1 Necroptosis-related lncRNAs in gastrointestinal cancer patients

**Figure 1:**
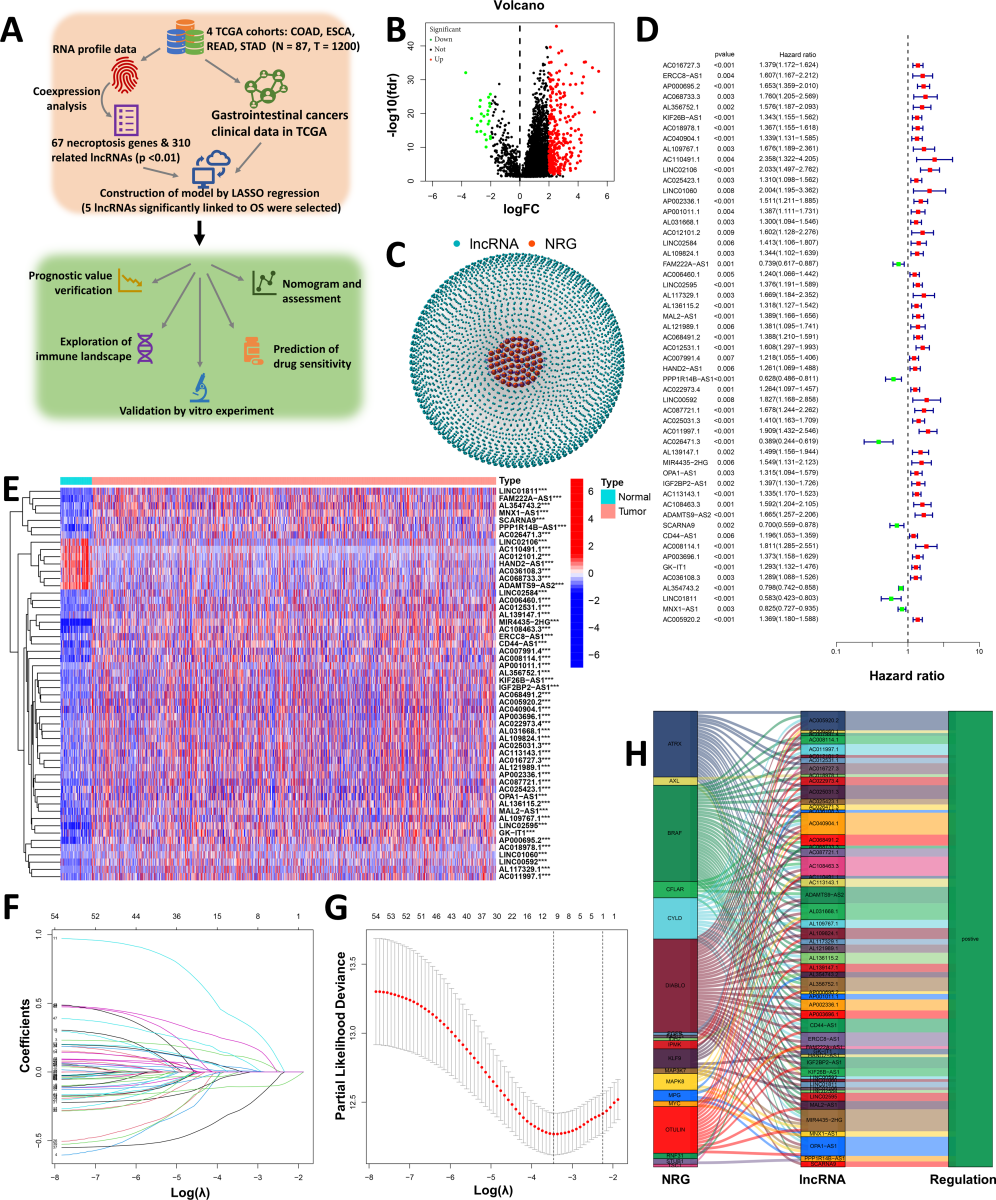
Construction of a necroptosis-related prognostic signature (Necro-lnc score). **(A)** Design and workflow of our study, including datasets and analysis methods. **(B)** Volcano plot of 310 differentially expressed necroptosis-related genes (|Log2FC| > 1 and FDR < 0.05). **(C)** Network of necroptosis-related genes and lncRNAs (correlation coefficients > 0.4 and p < 0.001). **(D)** The 54 necroptosis-related prognostic lncRNAs were extracted via univariate Cox regression analysis (p < 0.05). **(E)** Heatmap visualizing the detailed expression of 54 necroptosis-related lncRNAs in tumor and normal tissues. **(F)** The 10-fold cross-validation for variable selection in the LASSO model. **(G)** LASSO coefficient profile of the 5 necroptosis-related lncRNAs. **(H)** Sankey diagram of the NRGs and 54 necroptosis-related lncRNAs. ***p < 0.001.

The research flow chart is shown in **Figure 1A**. We obtained 87 normal samples and 1113 tumor samples from TCGA to identify necroptosis-related lncRNAs associated with GI cancers. Then, the relevant clinical information for 1006 patients was retrieved. According to the expression of 67 necroptosis-related genes (**Supplementary Table S3**) and differentially expressed lncRNAs (|Log2FC| > 1 and p < 0.05) between normal and tumor samples, 310 necroptosis-related lncRNAs (|Log2FC| > 2 and p < 0.01) were identified (35, 38). Among those genes, 287 were upregulated, and the rest were downregulated (**Figure 1B**). The network figure and comparison of for necroptosis-related genes (NRGs), such as AXL and BCL2, and lncRNAs are shown in **Figure 1C** and **Additional File 2**. **Figure 1C** shows which lncRNAs and NRG were associated with each NRG, and **Additional File 2** shows the correlation coefficients and p values between the NRGs and lncRNAs.

### 3.2 Construction of the Necro-lnc score

Univariate Cox (uni-Cox) regression analysis was performed to identify necroptosis-related lncRNAs that could serve as independent risk prognostic factors, and based on the results of the uni-Cox regression analysis, 54 necroptosis-related lncRNAs with the highest significance (minimum p value) were selected as possible prognostic factors (**Figure 1D**) (p < 0.05). Furthermore, a heatmap comparing these 54 necroptosis-related lncRNAs between tumor tissues and normal tissues was drawn (**Figure 1E**). The heatmap revealed that the 54 lncRNAs were significantly differentially expressed between tumor tissues and normal tissues, which confirmed that these necroptosis-related lncRNAs may be prognostic factors.

We performed LASSO regression on these lncRNAs to avoid overfitting and extracted 5 necroptosis-related lncRNAs from GI cancer patients when the first-rank value of Log(λ) was the minimum likelihood of deviance (**Figures 1F, G**). In addition, we found that all 5 lncRNAs were upregulated, as shown in the Sankey diagram (**Figure 1H**). We call this score the Necro-lnc score to reflect the relationship between this prognostic score, necroptosis and lncRNAs.

Using these 5 lncRNAs, we developed a formula to calculate the Necro-lnc score: Necro-lnc score = AP000695.2 × (0.19195) + LINC02106 × (0.52026) + AC011997.1 × (0.40859) + AC026471.3 × (-0.57136) + AL354743.2 × (-0.12547). Each coefficient in the formula was derived from the results of the multi-Cox regression analysis.

### 3.3 Verification of the prognostic value of the Necro-lnc score

**Figure 2:**
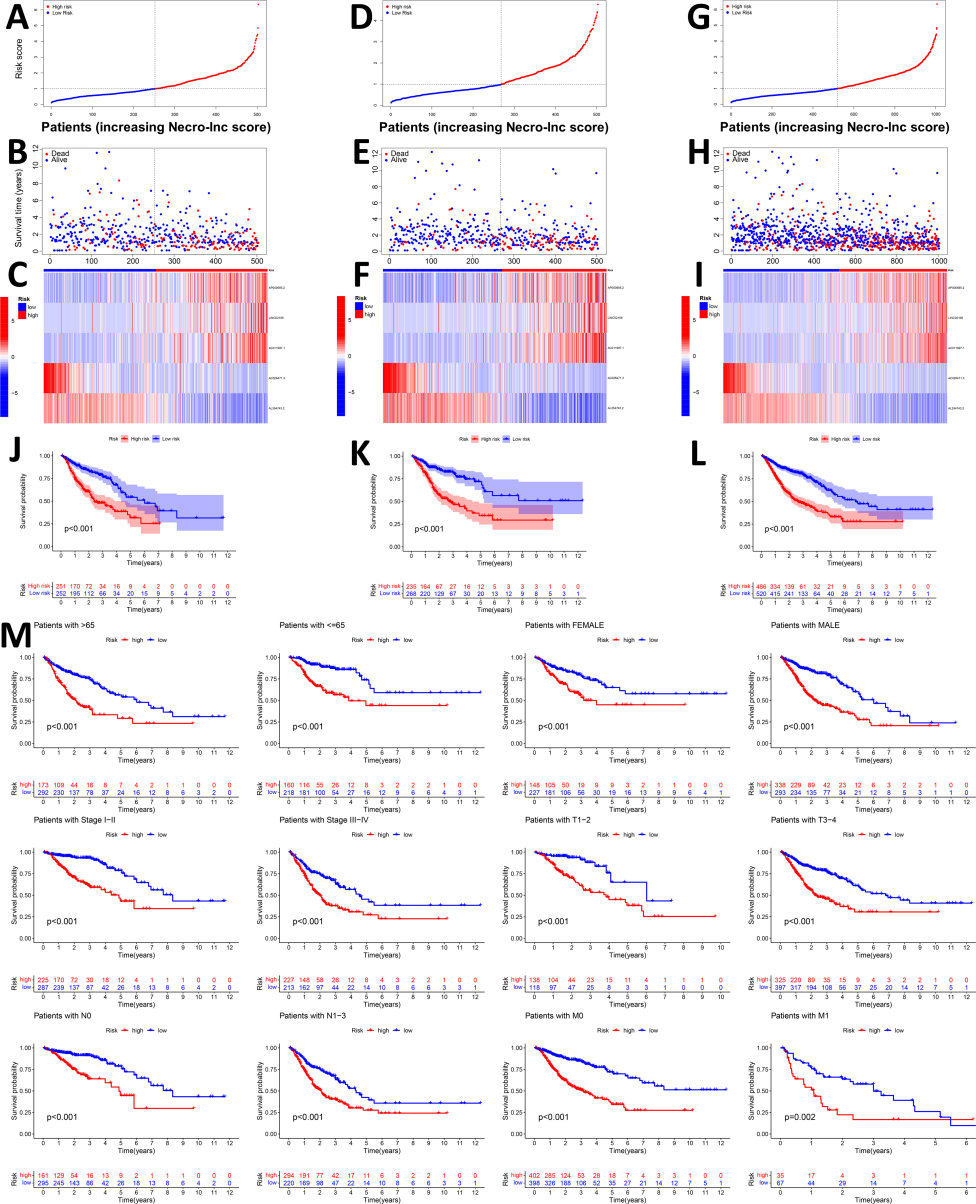
Prognostic value of the Necro-lnc score in the training, test, and entire sets. **(A, D, G)** Grouping the patients based on Necro-lnc score in the training **(A)**, test **(D)**, and entire sets **(G)**, respectively. **(B, E, H)** Comparison of the survival times and survival statuses between the low- and high-score groups of the training **(B)**, test **(E)**, and entire sets **(H)**, respectively. **(C, F, I)** Heatmaps of the expression of 5 lncRNAs in the training **(C)**, test **(F)**, and entire sets **(I)**, respectively. **(J–L)** Kaplan–Meier survival curves of the OS (survival probability) of patients in the low- and high-score groups in the training **(J)**, test **(K)**, and entire sets **(L)**, respectively. **(M)** Kaplan–Meier survival curves of the prognostic value of OS (survival probability) stratified by age, sex, stage, T stage, N stage, or M stage between the low- and high-score groups in the entire set.

With the above formula, the distributions of the Necro-lnc score, survival status, survival time, and relevant expression levels of these lncRNAs in patients were compared between the low- and high-score groups in the training, test, and entire sets. In the training set (**Figures 2A–C**), we first distinguished the low- and high-score groups according to the Necro-lnc score (**Figure 2A**), and using this information, we plotted the relationship between the survival status and the Necro-lnc score (**Figure 2B**). We found that with an increasing Necro-lnc score, the survival time of patients decreased significantly, and more patients died. We generated a heatmap of the expression levels of the 5 necroptosis-related lncRNAs and the Necro-lnc score (**Figure 2C**). With an increasing Necro-lnc score, the expression of three lncRNAs increased significantly (AP000695.2, LINC02106, and AC011997.1), whereas the expression of AC026471.3 and AL354743.2 was clearly decreased, which proves that the first three lncRNAs are high-risk lncRNAs and that the latter two lncRNAs are low-risk lncRNAs. We obtained the same results as the training set in the validation of the other sets. In the test set, the patients were also grouped by the Necro-lnc score (**Figure 2D**). We also found that patients with higher Necro-lnc scores experienced shorter survival times and had poor survival statuses (**Figure 2E**). The heatmap revealed the same correlation between the Necro-lnc score and the five lncRNAs as in the training set (**Figure 2F**). These results were also confirmed in the entire set (**Figures 2G–I**). The relationship between the Necro-lnc score and survival prognosis was further analyzed by constructing the K–M curves of the above three sets for the low- and high-Necro-lnc score groups. The results revealed that in the training set (**Figure 2J**), test set (**Figure 2K**) and entire set (**Figure 2L**), the survival time of the high-score group was significantly shorter than that of the low-score group. Simultaneously, we grouped patients according to conventional clinicopathological characteristics such as age, sex, stage, T stage, N stage, and M stage. Within each subgroup, K–M curves were generated according to the risk score. The high-score group of patients had distinctly poorer survival prognoses across all subgroups of age (>65 and ≤65 years old), sex (female and male), stage (I-II and III-IV), T stage (T1**–**2 and T3-4), N stage (N0 and N1-3), and M stage (M0 and M1) (**Figure 2M**).

### 3.4 Assessment of the Necro-lnc score and GSEA

**Figure 3:**
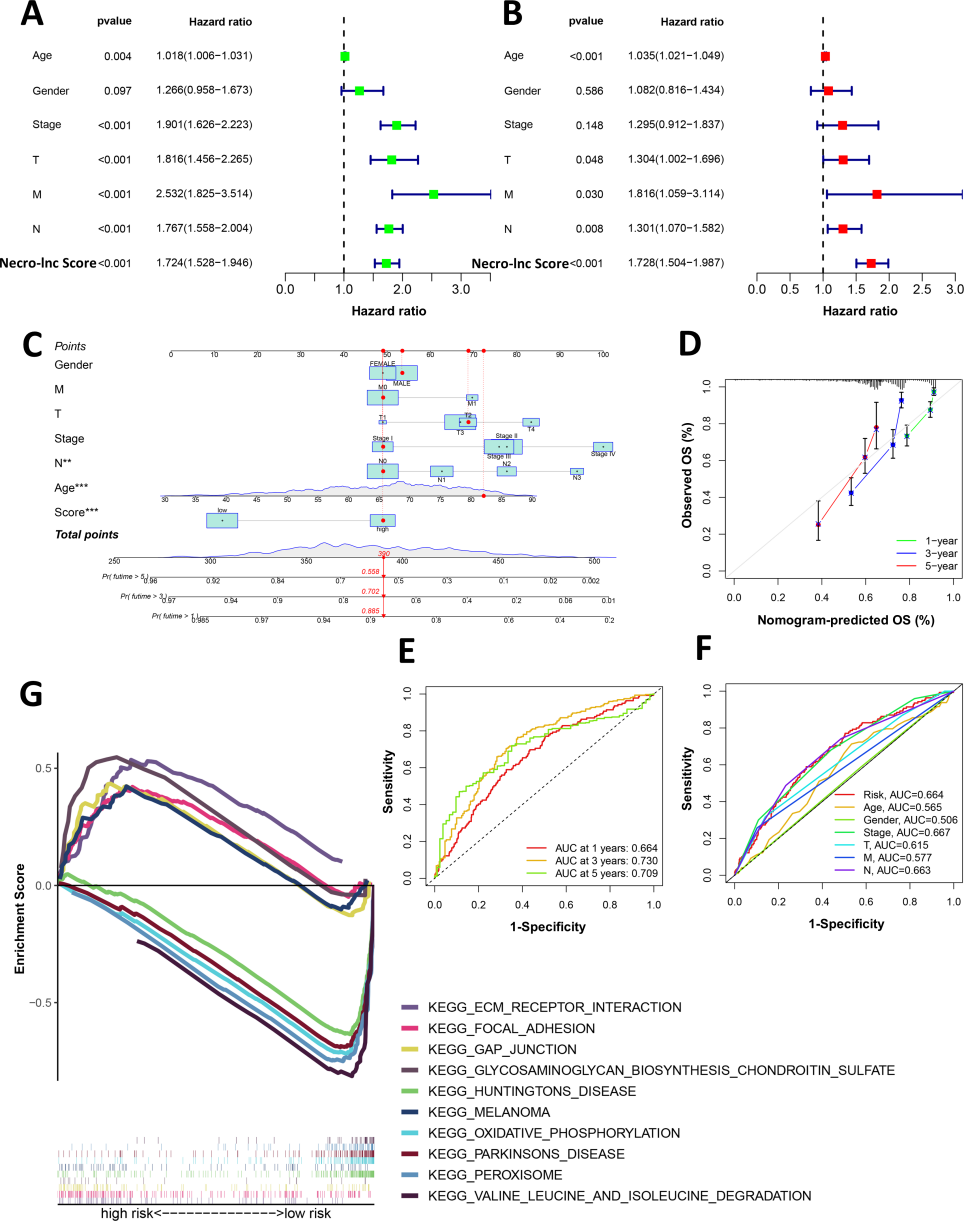
Assessment and GSEA of the Necro-lnc score. **(A, B)** Univariate **(A)** and multivariate **(B)** Cox analyses of the associations of clinical factors and the Necro-lnc score with OS. **(C)** The nomogram-integrated Necro-lnc score, age, sex, stage, and T, N and M stages predict the probabilities of 1-, 3-, and 5-year OS, respectively. **(D)** Calibration curves for the 1-, 3-, and 5-year OS of the entire cohort. **(E)** The 1-, 2-, and 3-year ROC curves of the entire set. **(F)** The 1-year ROC curves of the Necro-lnc score and clinical characteristics. **(G)** GSEA of the top 5 pathways significantly enriched in the low- and high-score groups. **p < 0.01, and ***p < 0.001.

Uni-Cox regression analysis, multi-Cox regression analysis and a nomogram were performed to verify whether the Necro-lnc score affected the prognosis of patients (**Figures 3A–D**). Uni- Cox regression analysis (**Figure 3A**) revealed that several clinicopathologic characteristics (such as age, stage, T stage, N stage, and M stage) and the Necro-lnc score were related to the prognosis of patients, among which the hazard ratio (HR) and 95% confidence interval (95% CI) of the Necro-lnc score were 1.724 and 1.528–1.946 (p < 0.001), which were close to the HR and 95% CI of the stage (1.901 and 1.626–2.223; p < 0.001), T stage (1.816 and 1.456–2.265; p < 0.001) and N stage (1.767 and 1.558–2.004; p < 0.001). the HR and 95% CI of the Necro-lnc score were significantly improved compared with those of age (1.018 and 1.006–1.031; p = 0.004). These findings prove that the Necro-lnc score has the same potential as the stage, T stage and N stage in assessing patient prognosis.

We conducted a multi-Cox regression analysis to further verify whether the Necro-lnc score could be an independent prognostic factor for patients (**Figure 3B**). The HR and 95% CI of the Necro-lnc score were 1.728 and 1.504–1.987 (p<0.001), which proves that the prognosis determine with the Necro-lnc score is independent of conventional clinicopathologic characteristics (age, stage, T stage, N stage and M stage). Additionally, we could conclude that the HR and 95% CI of the Necro-lnc score were better than the HR and 95% CI of age (1.035 and 1.021–1.049; p<0.001), stage (1.295 and 0.912–1.837; p=0.148), T stage (1.304 and 1.002–1.696; p=0.048), N stage (1.301 and 1.070–1.582; p=0.008) and M stage (1.816 and 1.059–3.114; p=0.030). These findings prove that the Necro-lnc score is meaningful for assessing the patient prognosis.

We constructed a nomogram to predict the 1-year, 3-year, and 5-year OS in GI cancer patients based on the following independent prognostic factors to verify the efficacy of the Necro-lnc score for determining the prognosis of patients: the Necro-lnc score, age, sex, stage, T stage, N stage and M stage (**Figure 3C**). According to the nomogram, we concluded that the gap in the score between the high-score group and the low-score group was almost 40 points, and when the total score of the nomogram and the patients’ survival rate changed, a corresponding change in the Necro-lnc score was observed. The 1-, 3-, and 5-year calibration plots demonstrated that the nomogram had good concordance with the predictions of 1-, 3-, and 5-year OS (**Figure 3D**), which proves the accuracy of the nomogram.

We then constructed time-dependent ROC curves to evaluate the sensitivity and specificity of the prognostic signature. The outcomes of the ROC curve analysis are illustrated by the area under the ROC curve (AUC). The 1-, 3-, and 5-year AUCs of the entire set were 0.664, 0.730, and 0.709, respectively (**Figure 3E**). In the 1-year ROC curve of the risk signature, the top 3 AUCs of those clinicopathologic characteristics were the Necro-lnc score (0.664), stage (0.667) and N stage (0.663), which showed their main predictive abilities (**Figure 3F**). These findings prove that the prognostic value of the Necro-lnc score is as important as the TNM stage.

We used GSEA software to assess the Kyoto Encyclopedia of Genes and Genomes (KEGG) pathways that were enriched in the high-score groups and low-score groups across the entire set and to investigate differences in biological functions between risk groups (**Figure 3G**). In the high-score group, gap junction, ECM–receptor interaction, focal adhesion, regulation of the actin cytoskeleton, calcium signaling pathway, phosphatidylinositol signaling system, and JAK STAT signaling pathway among the top 15 pathways were strongly associated with tumor progression and metastasis, whereas other pathways, such as melanoma, renal cell carcinoma, and glioma, were also enriched, which proves that these necroptosis-related lncRNAs may also play a role in pancancer regulation (**Supplementary Figure S1A**). In the low-score group, oxidative phosphorylation, peroxisome, citrate cycle, TCA cycle, selenoamino acid metabolism, arginine and proline metabolism, pyruvate metabolism, aminoacyl tRNA biosynthesis, and base excision repair were obviously enriched among the top 15 pathways and associated with tumor initiation and progression (all p < 0.05; FDR < 0.25; |normalized enrichment score (NES)| > 1.5) (**Supplementary Figure S1B**). Therefore, the prognostic value of necroptosis-related lncRNAs may be related to the above pathways, but the specific molecular mechanisms require further experiments.

### 3.5 Investigation of the tumor immune landscape in the groups stratified based on the Necro-lnc score

Immunotherapy has become a powerful clinical strategy for treating cancer, but effective prognostic markers for immunotherapy are currently lacking. Considering that necroptosis is an immunogenic form of cell death and may be closely related to tumor immunity, we analyzed the relationship between the Necro-lnc score and immunity and analyzed the differences in immunity in different score groups.

**Figure 4:**
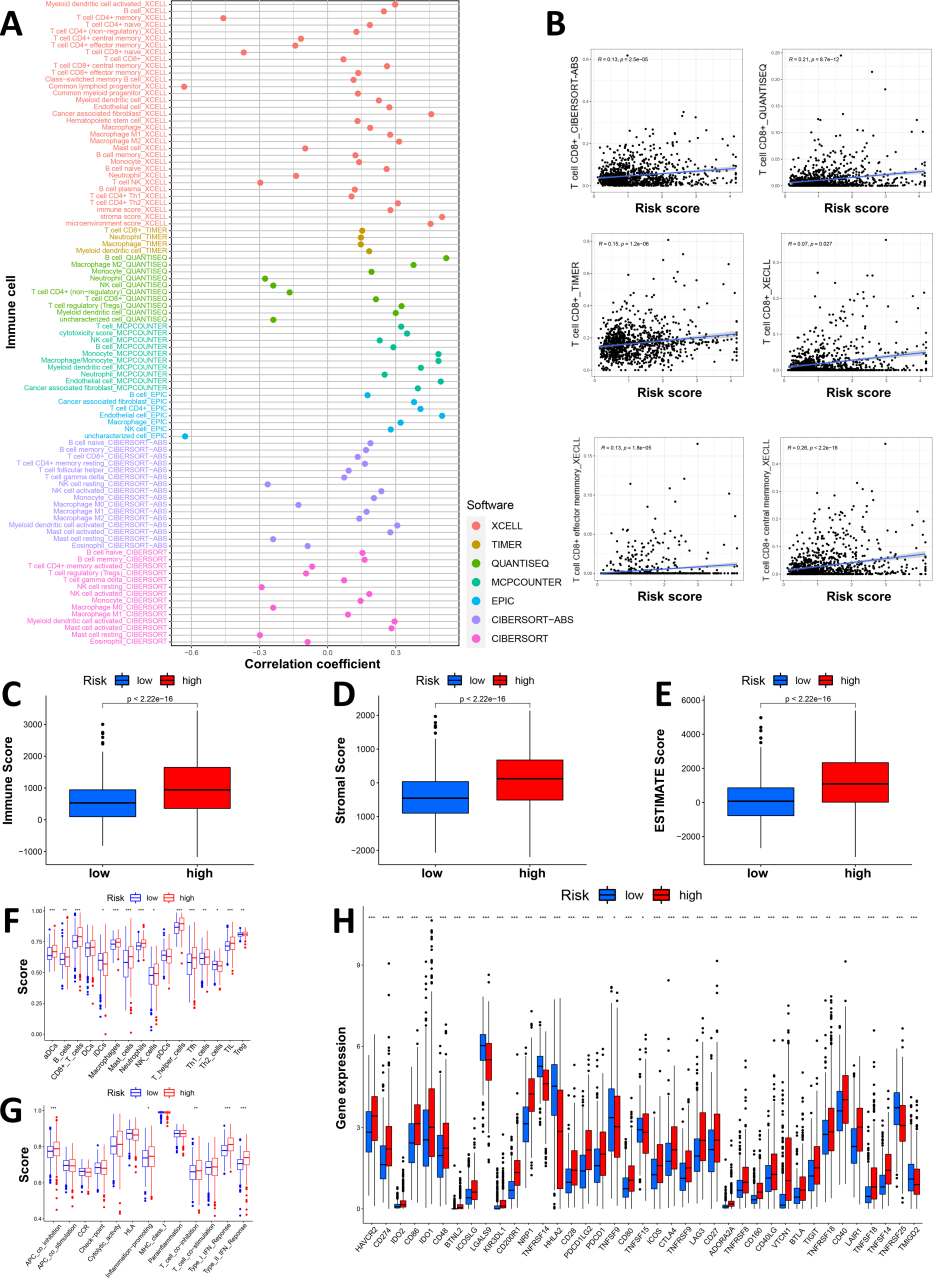
Investigation of the tumor immune landscape. **(A)** The immune cell bubble plot of the risk groups. **(B)** The correlation between the Necro-lnc score and CD8^+^ T cells on different platforms. **(C–E)** Comparison of TIME scores, including the immune score **(C)**, stromal score **(D)** and ESTIMATE score **(E)**, between the low- and high-score groups. **(F, G)** ssGSEA scores of immune cells **(F)** and immune functions **(G)** in the score groups. **(H)** Differences in the expression of the 39 checkpoint genes across the score groups. *p < 0.05, **p < 0.01, and ***p < 0.001.

We first plotted an immune bubble plot of the low- and high-score groups of patients with GI cancers (**Figure 4A**). Among the infiltrating immune cells in the CIBERSORT platform, the index of activated NK cells was significantly positively correlated with the Necro-lnc score, and the index of resting NK cells was significantly negatively correlated. These findings suggest that the Necro-lnc score potentially reflects the function of NK cells, which are important indicators of tumor immunity. The Necro-lnc score was also significantly correlated with other infiltrating immune cells in each platform (all p < 0.05) (**Supplementary Table S4**). For example, the Necro-lnc score was positively correlated with neutrophils, macrophages (M1), and activated dendritic cells (aDCs) and negatively correlated with macrophages (M0). In particular, Necro-lnc scores were significantly associated with CD8+ T cells across all platforms (**Figure 4B**). The score of CD8+ T cells increased with increasing Necro-lnc score, suggesting that patients in the high-score group may respond better to immunotherapy.

We further explored the differences in the TIME between the low- and high-score groups. The relationship between the TIME score and the Necro-lnc score was plotted to verify the correlation between the TIME score and the Necro-lnc score (**Figures 4C–E**). According to the immune score, the infiltration of immune cells was greater in the high-score group than in the low-score group, which is a sign of hot tumors (**Figure 4C**). The high-score group had a higher stromal score (**Figure 4D**) and a higher ESTIMATE score (**Figure 4E**), which indicated a TIME with more infiltrating immune cells than the low-score group. We subsequently performed single-sample GSEA (ssGSEA) to explore the differences in the TIME of tumor-infiltrating immune cells and immune-related functions between the low- and high-Necro-lnc-score groups. The results for tumor-infiltrating immune cells revealed that 12 immune cell types (including aDCs, B cells, CD8+ T cells, macrophages, neutrophils, NK cells, T helper cells, Tfh cells, Th1 cells, TILs and Tregs) had higher ssGSEA scores in the high Necro-lnc score group (**Figure 4F**). Five immune-related functions (including inflammation-promoting, APC coinhibitory, T-cell coinhibitory, type I IFN response and type II IFN response functions) were also more active in the high Necro-lnc score group (**Figure 4G**). We also analyzed the relationship between the Necro-lnc score and immune checkpoints (**Figure 4H**). As shown in **Figure 4H**, most immune checkpoints also showed greater activation in the high-score group than in the low-score group. The co-occurrences of ligands and receptors, such as PDCD1LG2/CD274-PDCD1, CD86/CD80-CD28/CTLA4, TNFRSF9-TNFSF9 and TNFRSF14-TNFSF14, were confirmed. The results revealed that the TIME in the high-score group had more active immune cells and a greater immune cell infiltration status, indicating that these patients had hot tumors and could respond to immunotherapy more sensitively (30, 39). Therefore, we can distinguish hot and cold tumors by the Necro-lnc score. Thus, we can select appropriate checkpoints for patients with hot tumors and better immunotherapy responses and optimize these patients’ treatment plans (40, 41).

### 3.6 Validation of the prognosis and immune status in patients with different tumor types

**Figure 5:**
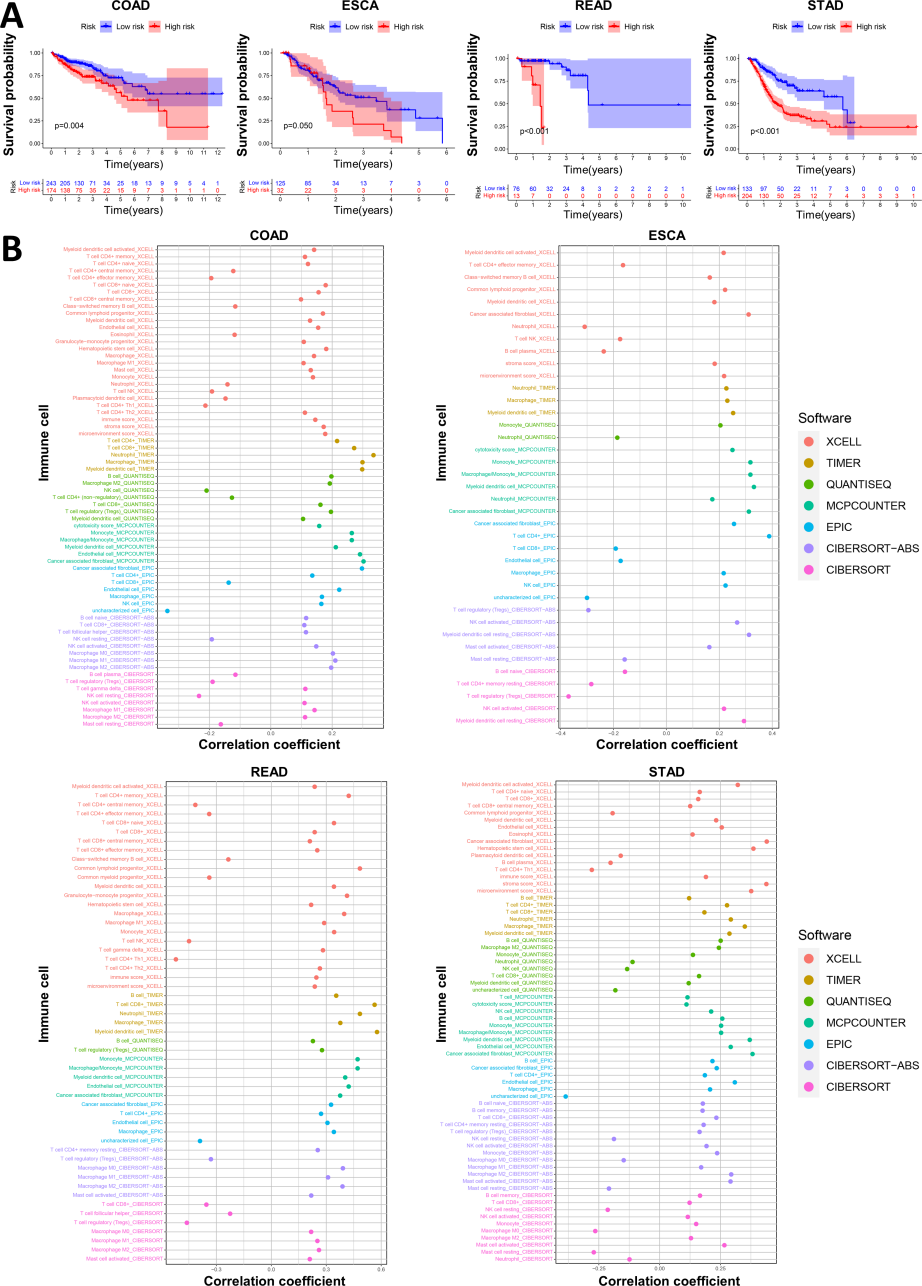
Validation of the prognosis and immunity in patients with different tumor types. **(A)** K–M curves of Necro-lnc score groups of patients with COAD, ESCA, READ and STAD. **(B)** The immune bubble plots of the Necro-lnc score groups obtained using the CIBERSORT, EPIC, MCPCOUNTER, QUANTISEQ, TIMER and XCELL platforms for patients with COAD, ESCA, READ and STAD, respectively.

In our study, we found that the Necro-lnc score was effective at predicting the prognosis of GI cancer patients and that significant differences in immune status existed between the high- and low-score groups. We avoided the confounding factor of different tumor types that could distort the prognosis and grouping effect of the signature by grouping the GI cancer patients according to different tumor types, including COAD, ESCA, READ and STAD. We plotted K–M curves (**Figure 5A**) and performed an immune analysis (**Figure 5B**) separately for each GI cancer. **Figure 5A** shows that in patients with each type of GI cancer, significant differences in the survival prognosis were still observed between the low- and high-score groups. According to the K–M curves (**Figure 5A**), the p value of COAD was 0.004, the p value of ESCA was 0.05, and the p values of READ and STAD were both less than 0.001. We subsequently evaluated the associations between the Necro-lnc score and the infiltration of immune cells in ESCA, STAD, COAD and READ (**Figure 5B**). CD4+ T cells, macrophages and myeloid dendritic cells were positively correlated with the Necro-lnc score in all four tumor types. In particular, neutrophils and activated NK cells were positively correlated with the Necro-lnc score in COAD, ESCA, and STAD. CD8+ T cells were positively correlated with the Necro-lnc score in COAD, READ, and STAD. Tregs were negatively correlated with Necro-lnc scores in ESCA and READ patients. Moreover, the TIME score and Necro-lnc score were positively correlated in each GI cancer (**Figure 5B**). We concluded that the immune cell infiltration of the four tumor types was highly consistent with the results for overall GI cancer. In each GI cancer patient, high scores were associated with a poor prognosis and high levels of infiltrating immune cell, consistent with the overall data. These results indicated that the tumor type was not a confounding factor affecting the Necro-lnc score, and we can evaluate the prognosis and immune function of patients with GI cancer via the Necro-lnc score.

### 3.7 Correlation between the Necro-lnc score and drug sensitivity

Drug therapy is an important approach for the comprehensive treatment of GI cancer. Therefore, we performed a drug sensitivity analysis between the low- and high-score groups using the pRRophetic R package. We found that for the 138 drugs in the GDSC2 database, the IC50 values of 110 drugs were significantly different between the low- and high-score groups (**Supplementary Table S5**). Among the 110 drugs, 69 drugs had lower IC50 values in the high-score group than in the low-score group, which meant that patients in the high-score group had higher sensitivity to thee 69 drugs, whereas patients in the low-score group were more sensitive to the other 41 drugs.

**Figure 6:**
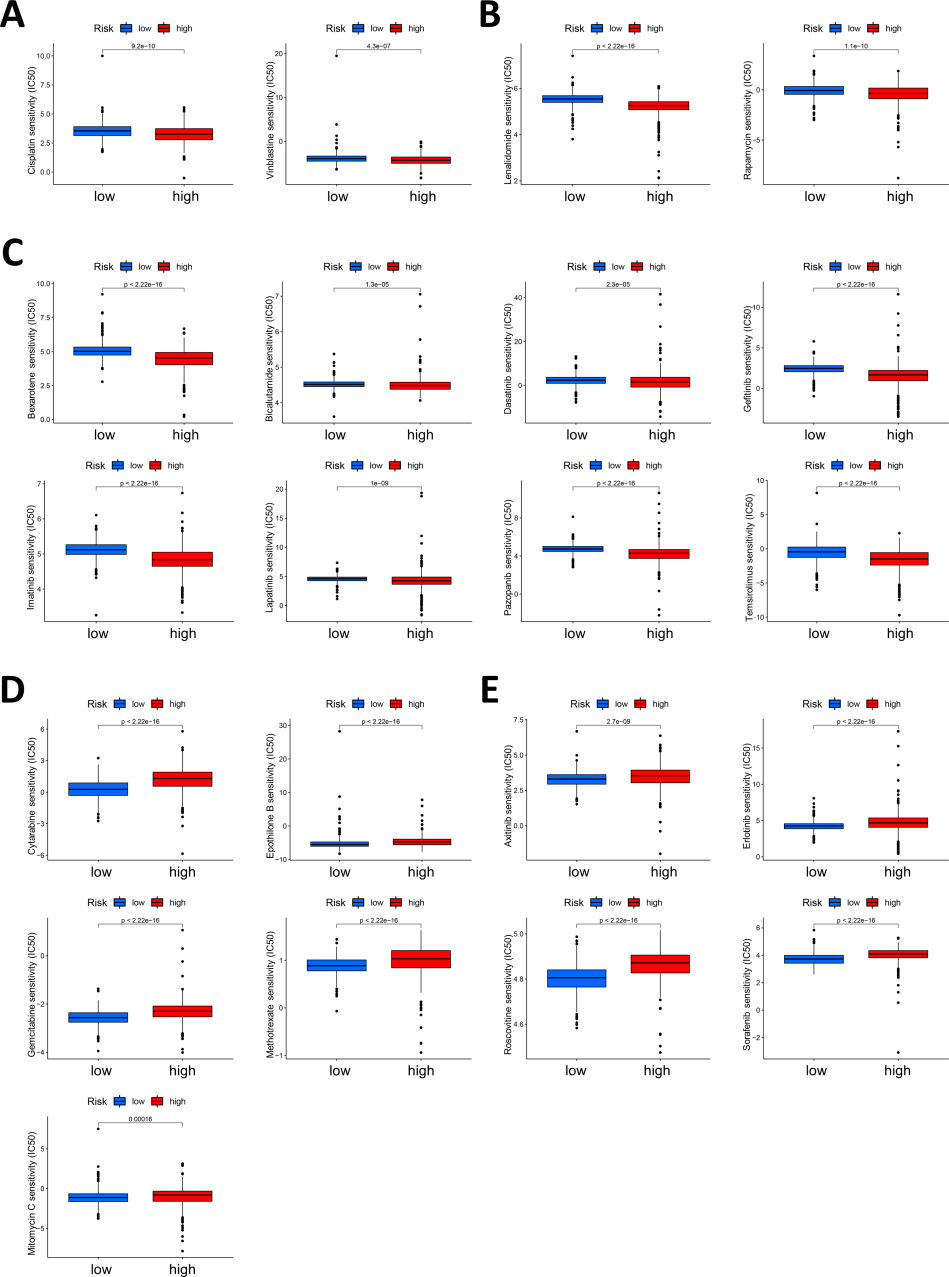
Correlation between the Necro-lnc score and drug sensitivity. **(A)** Analysis of the chemotherapeutic drugs to which the high-score group displayed greater sensitivity, including cisplatin and vinblastine. **(B)** Analysis of the immunotherapeutic drugs to which the high-score group was more sensitive, including lenalidomide and rapamycin. **(C)** Analysis of the targeted drugs to which the high-score group was more sensitive, including bexarotene, bicalutamide, dasatinib, gefitinib, imatinib, lapatinib, pazopanib and temsirolimus. **(D)** Analysis of the chemotherapeutic drugs to which the low-score group was more sensitive, including cytarabine, epothilone B, gemcitabine, methotrexate and mitomycin **(C, E)** Analysis of the targeted drugs to which the low-score group was more sensitive, including axitinib, erlotinib, roscovotine and sorafenib.

The high-score group was more sensitive to 2 chemotherapeutic drugs, namely, cisplatin and vinblastine (**Figure 6A**). We also found that the high-score group was more sensitive to 2 immunotherapeutic drugs (lenalidomide and rapamycin) (**Figure 6B**), which indicates that, compared with cold tumors in the low-score group, hot tumors in the high-score group may be more suitable for immunotherapy. Patients in the high-score group were more sensitive to various targeted drugs, including bexarotene, bicalutamide, dasatinib, gefitinib, imatinib, lapatinib, pazopanib and temsirolimus (**Figure 6C**, **Supplementary Figure S2A**). The high-score group was more sensitive to more types of targeted drugs, were more than the low-score group, suggesting that the high-score group may be more suitable for treatment with targeted therapy.

The low-score group displayed the greatest sensitivity to the chemotherapeutic drugs cytarabine, epothilone B, gemcitabine, methotrexate and mitomycin C (**Figure 6D**). These patients were sensitive to more types of chemotherapeutic drugs than patients in the high-score group, indicating that patients in the low-score group could benefit more from chemotherapy. Moreover, some targeted drugs, including axitinib, erlotinib, roscovotine and sorafenib, still had lower IC50 values in the low-score group (**Figure 6E**, **Supplementary Figure S2B**).

Our analysis suggested that the low- and high-score groups had different drug sensitivities. Overall, patients with high Necro-lnc scores are more sensitive to drug treatment, especially targeted therapy and immunotherapy, and patients with low Necro-lnc scores are more suitable for treatment with chemotherapy. These findings could provide valuable guidance for GI cancer treatment, which is beneficial for improving the patient prognosis.

### 3.8 Validation of the Necro-lnc score by a clustering analysis

**Figure 7:**
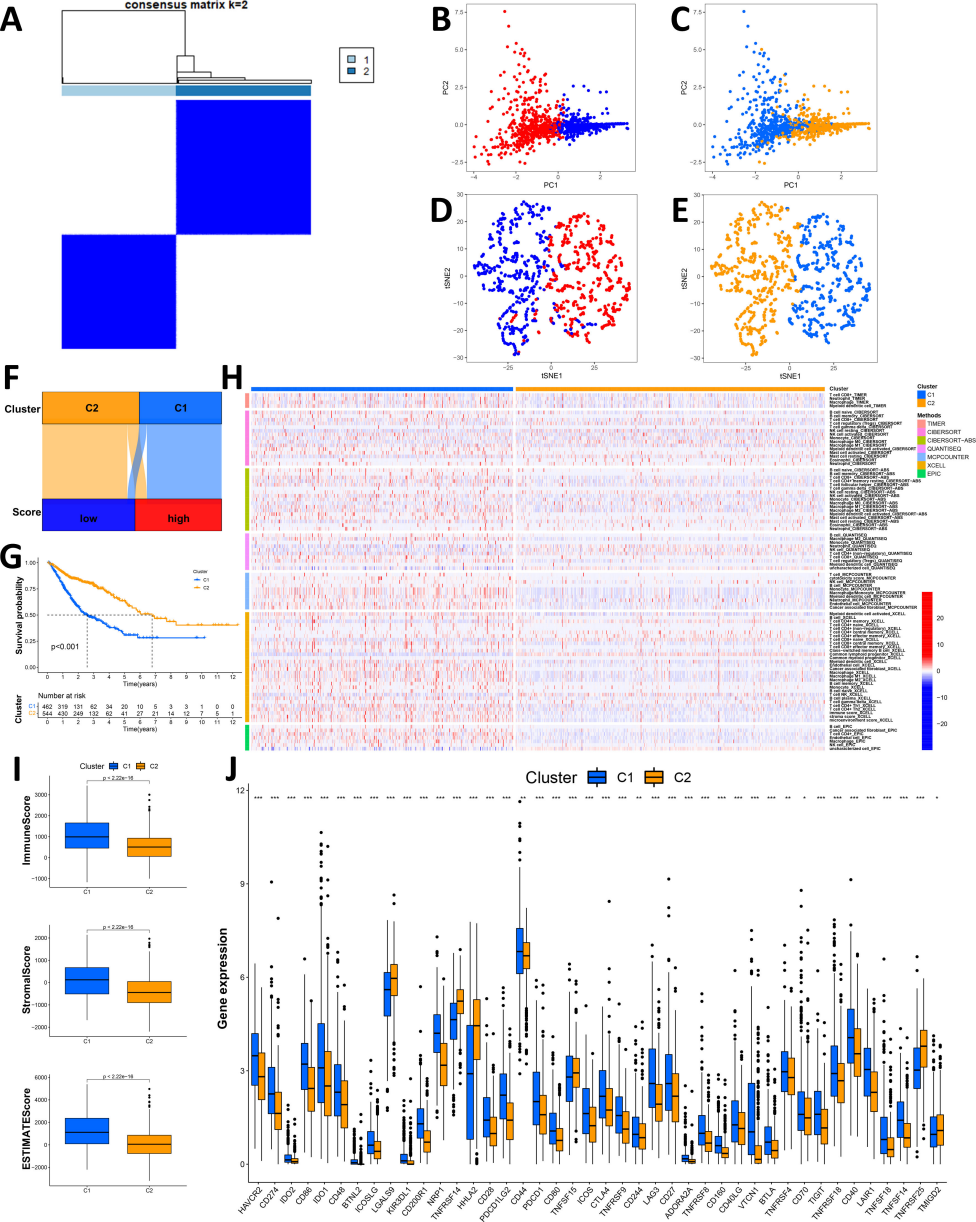
Validation of the Necro-lnc score by a clustering analysis. **(A)** Patients were divided into two clusters by ConsensusClusterPlus. **(B, C)** PCA of Necro-lnc score groups **(B)** and clusters **(C)**, respectively. **(D, E)** t-SNE analysis of Necro-lnc score groups **(D)** and clusters **(D)**, respectively. **(F)** Sankey diagram of Necro-lnc score groups and clusters. **(G)** Kaplan–Meier curves of OS across clusters. **(H)** Heatmap of immune cells in clusters obtained via the CIBERSORT, EPIC, MCPCOUNTER, QUANTISEQ, TIMER and XCELL platforms. **(I)** Comparison of immune-related scores between Clusters 1 and 2. **(J)** Differences in the expression of 42 immune checkpoint genes across clusters. *p < 0.05, **p < 0.01, and ***p < 0.001.

We performed a clustering analysis of the patients in our cohort based on the expression levels of the 5 lncRNAs in the Necro-lnc score formula to validate the biological correlation of the Necro-lnc score with GI cancer (37). The patients were regrouped into two clusters with the ConsensusClusterPlus (CC) R package (**Figure 7A**, **Supplementary Figure S3A**). Then, we employed PCA for dimensionality reduction and visualization of the data characteristics. The results verified that both the Necro-lnc score groups (**Figure 7B**) and the cluster groups (**Figure 7C**) could be clearly distinguished. t-SNE was performed for both the Necro-lnc score groups (**Figure 7D**) and the cluster groups (**Figure 7E**) to show the Necro-lnc score groups more intuitively. The data characteristics of the groups were further dispersed and still clearly distinguishable. In addition, Cluster 1 basically corresponded to the high-score group, and Cluster 2 basically corresponded to the low-score group. A Sankey diagram further verified the consistency of the Necro-lnc score groups and the clusters from the corresponding samples (**Figure 7F**). These findings suggested that the Necro-lnc score could classify patients into clusters based on 5 necroptosis-related lncRNAs.

Since the clusters were grouped based on 5 necroptosis-related lncRNAs without clinical data, the clustering analysis could avoid confounding bias when we constructed the Necro-lnc score using clinical data. Verifying the differences between Cluster 1 and Cluster 2 in terms of clinical characteristics could better confirm the biological correlations between lncRNAs and these clinical characteristics. We generated a K–M curve for the clusters (**Figure 7G**), and the results revealed that patients in Cluster 2 had a longer OS than patients in Cluster 1 (p < 0.001), which is consistent with the conclusion that Cluster 1 corresponds to the high-score group and that Cluster 2 corresponds to the low-score group. We subsequently generated a heatmap that reflected the immune cell infiltration of the TIME (**Figure 7H**). Tumors from patients in Cluster 1 were more significantly infiltrated by many types of immune cells, including CD8+ T cells, CD4+ T cells, NK cells, macrophages, neutrophils, B cells, monocytes and myeloid dendritic cells, on different platforms (**Supplementary Table S6**). We subsequently explored the differences in the TIMEs between Cluster 1 and Cluster 2, and we plotted the TIME scores for the different clusters (**Figure 7I**). Cluster 1 had higher immune scores, stromal scores and ESTIMATE scores, indicating a greater number of infiltrating immune cells in the TIME. An immune checkpoint analysis revealed that most of the immune checkpoints, such as PDCD1LG2/CD274-PDCD1, CD86/CD80-CD28/CTLA4, TNFRSF14-TNFSF14 and TNFRSF18-TNFSF18, were expressed at high levels in Cluster 1 (**Figure 7J**). The infiltration of immune cells, high TIME scores and activation of immune checkpoints indicated that Cluster 1 potentially consists essentially of hot tumors, similar to the high Necro-lnc score group. These results proved that the 5 lncRNAs associated with the Necro-lnc score were directly correlated with the immune landscape. Drug sensitivity was assessed, and the results for the clusters were compared with those of the Necro-lnc score groups. We found that the results for the clusters were basically consistent with those of the Necro-lnc score groups. Compared with those in the high-score group, the chemotherapeutic drugs (**Supplementary Figure S3B**), immunotherapeutic drugs (**Supplementary Figure S3C**) and targeted drugs (**Supplementary Figure S3D**) to which Cluster 1 was sensitive were almost the same as those of the high-score group. In Cluster 2, the chemotherapeutic drugs (**Supplementary Figure S3E**) and targeted drugs (**Supplementary Figure S3F**) identified were similar to those identified in the low-score group.

The results revealed that different clusters were correlated with patient survival, the tumor immune landscape, and drug sensitivity, which indicated the biological associations between the 5 necroptosis-related lncRNAs and these clinical characteristics. These findings suggest that the construction and clinical significance of the Necro-lnc score is biologically justified.

### 3.9 Validation of the Necro-lnc score *in vitro*

**Figure 8:**
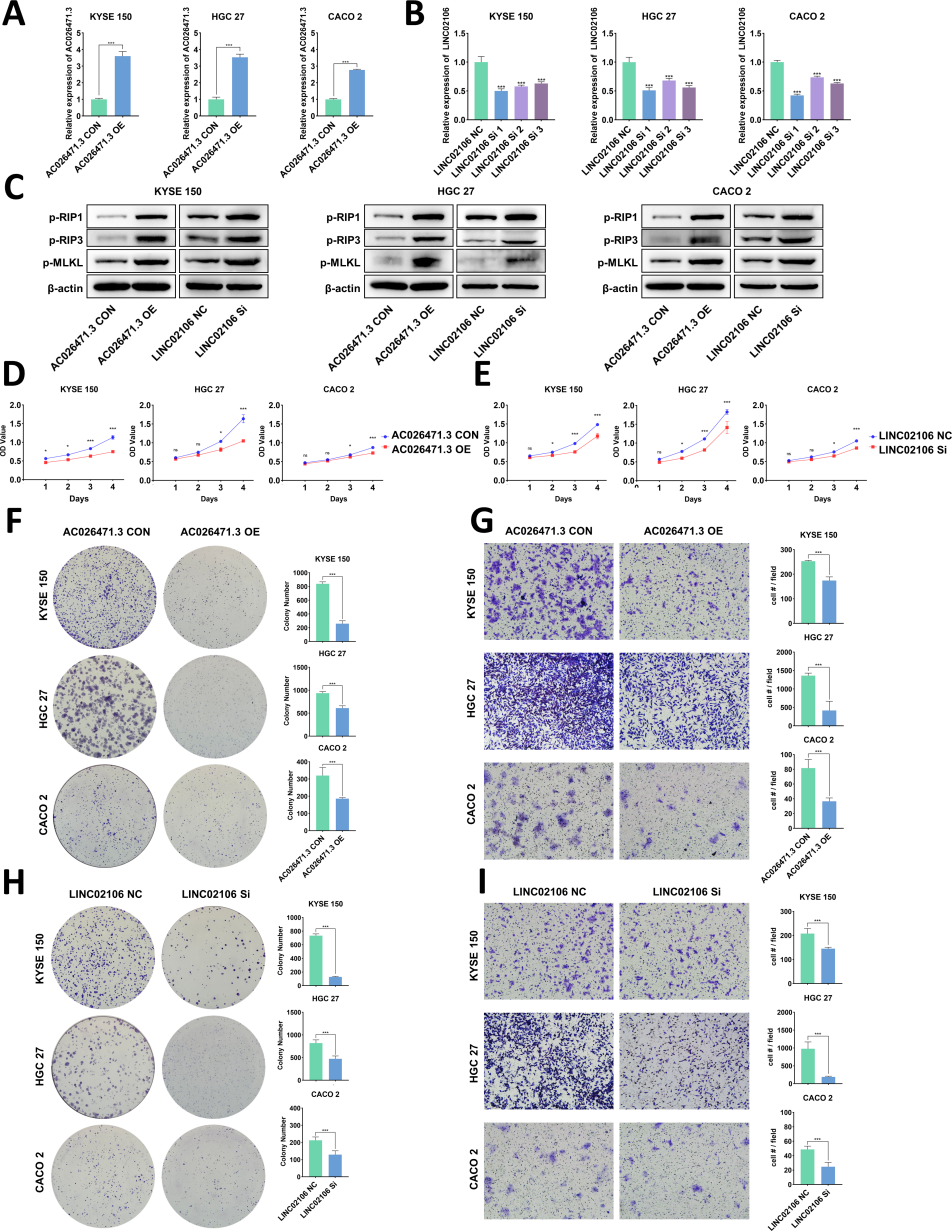
*In vitro* validation of candidate necroptosis-related lncRNAs (AC026471.3 and LINC02106). **(A, B)** Validation of AC026471.3 upregulation **(A)** and LINC02106 downregulation **(B)** via qPCR. **(C)** Validation of the effects of AC026471.3 overexpression and LINC02106 knockdown on the levels of necroptosis-related proteins (RIP 1, RIP 3, and MLKL) in KYSE150, HGC27, and CACO2 cells by WB. **(D, E)** Cell proliferation curves for AC026471.3-overexpressing **(D)** and LINC02106-knockdown **(E)** KYSE150, HGC27 and CACO2 cells determined using CCK-8 assays. **(F, H)** Colony formation assays of AC026471.3-overexpressing **(F)** and LINC02106-knockdown **(H)** KYSE150, HGC27 and CACO2 cells. **(G, I)** Transwell migration assays of AC026471.3-overexpressing **(G)** and LINC02106-knockdown **(I)** KYSE150, HGC27 and CACO2 cells. *p < 0.05; ***p < 0.001; and ns, not significant.

We tried to show that the lncRNAs in the Necro-lnc score had the same effect on tumors as the score itself as a method to confirm the scientific validity of the Necro-lnc score. In the construction of the Necro-lnc score, 3 lncRNAs (AP000695.2, LINC02106, and AC011997.1) had positive regression coefficients, and 2 lncRNAs (AC026471.3 and AL354743.2) had negative regression coefficients in the calculation formula among the 5 necroptosis-related lncRNAs. The regression coefficients represent their tumor-promoting and tumor-suppressing effects. Regression coefficients with larger absolute values represent stronger effects. The most significant tumor-promoting lncRNA (LINC02106) and the most significant tumor-suppressing lncRNA (AC026471.3) were selected for the experiment assessing the phenotypes of GI cancer cells to verify their biological function and the scientific validity of the Necro-lnc score. Cell lines with transient AC026471.3 overexpression and LINC02106 knockdown were established in KYSE 150, HGC 27 and CACO 2 cells. The expression of the target lncRNA was measured by qRT–PCR, and the results confirmed that the transiently transfected cell lines were successfully constructed (p < 0.001) (**Figures 8A, B**). LINC02106 Si 1 was selected as the small interfering RNA for subsequent *in vitro* experiments to obtain a more significant difference in LINC02106 expression.

The key proteins of the necroptosis pathway (RIP 1, RIP 3, and MLKL) were detected in the three transient cell lines. The results revealed that AC026471.3 overexpression and LINC02106 knockdown increased the expression of necroptosis-related proteins, suggesting the activation of the necroptosis pathway (**Figure 8C**).

As shown by the results of the CCK-8 assays, AC026471.3 overexpression impeded the proliferation of all three cell lines (**Figure 8D**), and LINC02106 knockdown had the same effect (**Figure 8E**). We also performed colony formation assays. AC026471.3-overexpressing cells (**Figure 8F**) and LINC02106-knockdown cells (**Figure 8H**) presented reduced colony formation. In addition, Transwell migration assays were performed to evaluate cell motility, and both AC026471.3 overexpression (**Figure 8G**) and LINC02106 knockdown (**Figure 8I**) suppressed cell migration in these assays.

## 4 Discussion

At present, the prognosis of patients with GI cancers is unsatisfactory, and reliable markers are needed to predict the prognosis of patients with GI cancers; however, the current markers still need to be further improved. Necroptosis is a newly discovered form of programmed cell death. Accumulating evidence suggests that the activation or inactivation of necroptosis-related pathways plays crucial roles in tumor progression, metastasis and the TIME (10). Moreover, the important roles of lncRNAs in tumors have been widely reported (42). However, the effects of necroptosis-related lncRNAs on predicting the prognosis of patients with GI cancers and guiding drug treatment still need to be further confirmed. Therefore, we aimed to construct a Necro-lnc score utilizing necroptosis-related lncRNAs to distinguish high- and low-risk GI cancer patients, which could provide novel insights for prognostic prediction and personalized treatment strategies in GI cancer patients.

Previous studies have investigated the relationship between lncRNAs and non-apoptotic cell death mechanisms constructing prognostic signatures based on lncRNA expression (43–45). In contrast to these studies which were limited to specific cancer types, our research explores necroptosis-related lncRNAs across pan-GI cancers, highlighting the broader applicability and clinical relevance of the Necro-lnc score. In our study, we analyzed the expression of necroptosis-related lncRNAs in GI cancer patients and the clinical information of these patients in TCGA database and constructed a risk signature (Necro-lnc score) based on 5 necroptosis-related lncRNAs. We then verified the predictive effect of the Necro-lnc score by constructing K–M curves and found that it can predict the patient prognosis well. By further analyzing immune landscapes, we found that the Necro-lnc score could be used as a marker to distinguish cold and hot tumors. In the drug sensitivity analysis, the high-score group was more sensitive to targeted drugs and immunotherapeutic drugs, whereas the low-score group was more sensitive to more chemotherapeutic drugs. These findings suggest that the Necro-lnc score has potential value in facilitating personalized immunotherapy. Furthermore, we performed *in vitro* experiments to verify these results and reported that the AC026471.3-overexpressing and LINC02106-knockdown cell lines presented decreased proliferation and reduced metastasis, consistent with the trend toward an improved prognosis for patients in the low-score group.

We performed a KEGG enrichment analysis of the low- and high-score groups. The results revealed that differentially expressed genes were enriched in many signaling pathways, including the JAK/STAT signaling pathway. The JAK-STAT pathway has been reported to play a role in the pathogenesis and progression of GI cancer (46, 47). Moreover, the STAT3 signaling pathway plays an important role in the development of a tumorigenic inflammatory microenvironment (48). These findings suggest that the JAK/STAT signaling pathway may be closely related to the TIME, with more immune cells infiltrating GI cancer.

Immunotherapy has emerged as a major therapeutic modality in oncology. Currently, however, the majority of patients with cancer do not benefit from immunotherapy. The most direct evidence is that 50–80% of patients with GI cancers treated with ICIs do not benefit from these drugs, and many patients experience severe adverse events (49). Current mainstream immunotherapies, including ICIs and chimeric antigen receptor (CAR)-T cells, have certain limitations: weak selectivity, large side effects and poor immunotherapy response rates (33, 50). Although immunotherapy is a potential treatment, effective markers are still lacking. The immune cell infiltration must be evaluated status and immune checkpoints must be screened via the Necro-lnc score to overcome these limitations.

Recent advances in immunology have enabled the identification of patients who are more likely to respond to immunotherapy (30). Related studies have shown that molecular subtypes, also known as cold and hot tumors, are closely related to the TIME (51, 52). Different subtypes have different TIMEs, leading to different prognoses and immunotherapy responses (40, 41). By further analyzing immune landscapes, we found that the high-score group presented greater immune cell infiltration, which is generally considered to indicate hot tumors (30, 39), suggesting that the Necro-lnc score could be used as a marker to distinguish cold and hot tumors. In addition, many immune checkpoints, such as PDCD1LG2/CD274-PDCD1, CD86/CD80-CD28/CTLA4, TNFRSF9-TNFSF9 and TNFRSF14-TNFSF14, were highly expressed in the high-score group, which indicates that these molecules are feasible therapeutic targets for immunotherapy.

In the drug sensitivity analysis, the high-score group was more sensitive to two immunotherapeutic drugs (lenalidomide and rapamycin), suggesting that immunotherapy potentially becomes a more effective treatment for hot tumors in the high-score group. Moreover, the high-score group exhibited greater sensitivity to more targeted drugs, whereas the low-score group displayed greater sensitivity to more chemotherapeutic drugs, indicating that the Necro-lnc score can guide the choice of more appropriate treatments for patients. These findings suggest that the Necro-lnc score can not only predict the patient prognosis and evaluate the TIME but also is helpful for individual GI cancer therapy.

Our Western blot analysis revealed consistent upregulation of p-RIP1, p-RIP3, and p-MLKL in LINC02106-knockdown and AC026471.3-overexpressing cell lines compared to controls, suggesting that these lncRNAs play regulatory roles in necroptosis. These results align with previously established mechanisms of lncRNA-mediated necroptosis modulation, for instance, lncRNA NRF suppresses miR-873, leading to the activation of RIP1/RIP3 (53), while depletion of LINC00176 has been shown to disrupt the cell cycle and induce necroptosis in hepatocellular carcinoma via the release of tumor-suppressive miRNAs (54). Collectively, these findings offer new insights into the regulatory roles of lncRNAs in necroptosis.

Since the Necro-lnc score was constructed based on the expression of necroptosis-related genes and clinical data, confounding bias may exist in the correlation analysis of the score with the immune landscape and drug sensitivity. We further clarified the biological associations of the 5 necroptosis-related genes with the immune landscape and drug sensitivity by dividing patients into groups, and we divided patients into two clusters based on the expression of the 5 necroptosis-related lncRNAs without clinical data (37). The results of the correlation analysis of clusters were almost identical to those of the Necro-lnc score groups, excluding the possibility of confounding bias.

The lncRNA-based evaluation score makes it easier to obtain biological samples for a molecular biology-based diagnosis. Moreover, the Necro-lnc score showed values both in predicting the prognosis and evaluating immune cell infiltration, indicating the potential of this score for clinical application. Therefore, the Necro-lnc score is helpful for the precise treatment of patients with GI cancer.

This study has several limitations. Some shortcomings and deficiencies persist in our Necro-lnc score. In this study, when we constructed a prediction signature of necroptosis-related pathways, we focused only on the RNA level and did not analyze the protein level; rather, necroptosis was determined at the protein level. Therefore, necroptosis in GI cancer cannot be fully reflected by RNA alone. However, at the same time, focusing only on the RNA level makes it easier to obtain samples for detection and evaluation. Moreover, targeted therapy for lncRNAs is not complete at the current stage, and thus if a protein-based signature can be added, it will be more conducive to GI cancer treatment. Due to the limited availability of patient samples, this study lacked validation in ICI cohorts, and thus the immunotherapy prediction capability of the Necro-lnc score requires further verification. However, this limitation is common among studies investigating novel biomarkers at the developmental stage (55). Despite this, our TCGA-based findings are robust, and the potential of the Necro-lnc score to predict both prognosis and immunotherapy response in pan-GI cancers remains noteworthy and promising.

## 5 Conclusion

In this study, the Necro-lnc score, a new predictive signature, was developed to predict the prognosis, and evaluate immune cell infiltration and drug sensitivity in patients with GI cancer. Therefore, the Necro-lnc score is helpful for formulating individualized treatment plans for patients with GI cancer and promoting the development of precision treatment for patients with GI cancer. This study reveals that targeting necroptosis and lncRNAs is potentially valuable for GI cancer immunotherapy. The feasibility needs to be confirmed and optimized in future studies.

## Data Availability

The original contributions presented in the study are included in the article/**Supplementary Material**. Further inquiries can be directed to the corresponding authors.
