# Supplementary material for: Construction and validation of a necroptosis-related lncRNA signature for predicting the prognosis of gastrointestinal cancer patients
Source: Front Immunol. 2025 Aug 14;16:1591252. doi: 10.3389/fimmu.2025.1591252 (PMC12391139; doi:10.3389/fimmu.2025.1591252)
Supplement: Supplementary file 2 [file Table2.doc]

| NRG | lncRNA | cor | pvalue | Regulation |
| --- | --- | --- | --- | --- |
| TSC1 | AC004148.1 | 0.429583279 | 3.37E-51 | positive |
| DIABLO | AC004148.1 | 0.468200979 | 1.02E-61 | positive |
| RNF31 | AC004148.1 | 0.428098829 | 8.05E-51 | positive |
| OTULIN | AL391863.2 | 0.43590805 | 7.88E-53 | positive |
| DIABLO | AL391863.2 | 0.777392588 | 6.32E-226 | positive |
| BRAF | AL391863.2 | 0.65134829 | 2.36E-135 | positive |
| OTULIN | AC092802.1 | 0.43822247 | 1.95E-53 | positive |
| CYLD | AC092802.1 | 0.470992898 | 1.57E-62 | positive |
| DIABLO | AC092802.1 | 0.775916082 | 1.57E-224 | positive |
| BRAF | AC092802.1 | 0.706998876 | 2.40E-169 | positive |
| ATRX | AC092802.1 | 0.451575847 | 5.06E-57 | positive |
| DIABLO | AC008883.2 | 0.686761676 | 4.25E-156 | positive |
| BRAF | AC008883.2 | 0.602839882 | 4.36E-111 | positive |
| MAPK8 | AC091152.2 | 0.441467458 | 2.72E-54 | positive |
| IPMK | AC091152.2 | 0.449672859 | 1.68E-56 | positive |
| OTULIN | AC091152.2 | 0.438254984 | 1.92E-53 | positive |
| CYLD | AC091152.2 | 0.403531579 | 7.80E-45 | positive |
| MAP3K7 | AC091152.2 | 0.443977203 | 5.82E-55 | positive |
| DIABLO | AC091152.2 | 0.63443489 | 2.13E-126 | positive |
| BRAF | AC091152.2 | 0.636995359 | 1.02E-127 | positive |
| ATRX | AC091152.2 | 0.507101472 | 9.07E-74 | positive |
| DIABLO | AL121772.2 | 0.630293948 | 2.73E-124 | positive |
| BRAF | AL121772.2 | 0.534731217 | 2.51E-83 | positive |
| KLF9 | PGM5P4-AS1 | 0.431765342 | 9.31E-52 | positive |
| MAPK8 | AC007684.2 | 0.401531342 | 2.28E-44 | positive |
| OTULIN | AC007684.2 | 0.463370699 | 2.51E-60 | positive |
| CYLD | AC007684.2 | 0.419478401 | 1.16E-48 | positive |
| DIABLO | AC007684.2 | 0.747220525 | 2.25E-199 | positive |
| BRAF | AC007684.2 | 0.684859975 | 6.57E-155 | positive |
| OTULIN | AL353704.1 | 0.436327464 | 6.13E-53 | positive |
| DIABLO | AL353704.1 | 0.560748959 | 3.57E-93 | positive |
| BRAF | AL353704.1 | 0.549276701 | 1.00E-88 | positive |
| ATRX | AL353704.1 | 0.435981055 | 7.54E-53 | positive |
| DIABLO | AP001020.2 | 0.695227388 | 1.66E-161 | positive |
| BRAF | AP001020.2 | 0.515014601 | 2.04E-76 | positive |
| ATRX | AP001020.2 | 0.409586559 | 2.90E-46 | positive |
| RNF31 | AP001020.2 | 0.436073896 | 7.14E-53 | positive |
| OTULIN | AC084824.5 | 0.440823907 | 4.02E-54 | positive |
| DIABLO | AC084824.5 | 0.622151944 | 3.09E-120 | positive |
| BRAF | AC084824.5 | 0.567164011 | 9.73E-96 | positive |
| ATRX | AC084824.5 | 0.411919123 | 8.02E-47 | positive |
| CYLD | AC090971.2 | 0.411150477 | 1.23E-46 | positive |
| DIABLO | AC090971.2 | 0.740937458 | 2.57E-194 | positive |
| BRAF | AC090971.2 | 0.61427181 | 2.00E-116 | positive |
| ATRX | AC090971.2 | 0.407762612 | 7.87E-46 | positive |
| AXL | AC010754.1 | 0.535180533 | 1.73E-83 | positive |
| DIABLO | AL109809.4 | 0.45635684 | 2.40E-58 | positive |
| BRAF | AL109809.4 | 0.472273136 | 6.60E-63 | positive |
| BACH2 | LINC00900 | 0.504133497 | 8.57E-73 | positive |
| BRAF | AL355377.4 | 0.409963761 | 2.36E-46 | positive |
| ATRX | AL355377.4 | 0.4815071 | 1.15E-65 | positive |
| OTULIN | AC044781.1 | 0.435665547 | 9.12E-53 | positive |
| CYLD | AC044781.1 | 0.410249482 | 2.01E-46 | positive |
| DIABLO | AC044781.1 | 0.667707501 | 1.40E-144 | positive |
| BRAF | AC044781.1 | 0.61609823 | 2.67E-117 | positive |
| OTULIN | ARHGAP15-AS1 | 0.423212597 | 1.37E-49 | positive |
| CYLD | ARHGAP15-AS1 | 0.495764108 | 4.29E-70 | positive |
| DIABLO | ARHGAP15-AS1 | 0.748193543 | 3.60E-200 | positive |
| CFLAR | ARHGAP15-AS1 | 0.424859492 | 5.29E-50 | positive |
| BRAF | ARHGAP15-AS1 | 0.683941182 | 2.45E-154 | positive |
| ATRX | ARHGAP15-AS1 | 0.428538444 | 6.22E-51 | positive |
| TSC1 | AL158071.2 | 0.421722777 | 3.22E-49 | positive |
| DIABLO | AC010487.1 | 0.401214851 | 2.70E-44 | positive |
| DIABLO | AC139887.5 | 0.409312206 | 3.37E-46 | positive |
| MAPK8 | AC010186.3 | 0.431855904 | 8.83E-52 | positive |
| OTULIN | AC010186.3 | 0.559161253 | 1.51E-92 | positive |
| CYLD | AC010186.3 | 0.574740556 | 7.68E-99 | positive |
| MAP3K7 | AC010186.3 | 0.469508751 | 4.25E-62 | positive |
| DIABLO | AC010186.3 | 0.691725424 | 3.03E-159 | positive |
| CFLAR | AC010186.3 | 0.446197946 | 1.47E-55 | positive |
| BRAF | AC010186.3 | 0.702395361 | 3.11E-166 | positive |
| ATRX | AC010186.3 | 0.485237837 | 8.35E-67 | positive |
| SIRT3 | AP001505.1 | 0.444406016 | 4.47E-55 | positive |
| TRAF2 | AP001505.1 | 0.494698342 | 9.34E-70 | positive |
| SLC39A7 | AP001505.1 | 0.416149156 | 7.58E-48 | positive |
| TARDBP | AP001505.1 | 0.438058618 | 2.16E-53 | positive |
| DIABLO | AC005520.5 | 0.573101748 | 3.66E-98 | positive |
| BRAF | AC005520.5 | 0.481807521 | 9.32E-66 | positive |
| DIABLO | TMED2-DT | 0.504658593 | 5.77E-73 | positive |
| RNF31 | TMED2-DT | 0.417635998 | 3.28E-48 | positive |
| OTULIN | AL353768.2 | 0.454250386 | 9.25E-58 | positive |
| CYLD | AL353768.2 | 0.410625781 | 1.64E-46 | positive |
| DIABLO | AL353768.2 | 0.743193514 | 4.08E-196 | positive |
| BRAF | AL353768.2 | 0.66190236 | 3.07E-141 | positive |
| OTULIN | AL356124.1 | 0.406993937 | 1.20E-45 | positive |
| DIABLO | AL356124.1 | 0.693599264 | 1.89E-160 | positive |
| BRAF | AL356124.1 | 0.634496757 | 1.98E-126 | positive |
| ATRX | AL356124.1 | 0.400742864 | 3.47E-44 | positive |
| DIABLO | AF129075.2 | 0.401527788 | 2.28E-44 | positive |
| DIABLO | AP000302.1 | 0.563752959 | 2.28E-94 | positive |
| BRAF | AP000302.1 | 0.477102079 | 2.44E-64 | positive |
| MAPK8 | AC003681.1 | 0.420938862 | 5.04E-49 | positive |
| IPMK | AC003681.1 | 0.406172801 | 1.87E-45 | positive |
| OTULIN | AC003681.1 | 0.492908963 | 3.43E-69 | positive |
| CYLD | AC003681.1 | 0.492914051 | 3.42E-69 | positive |
| DIABLO | AC003681.1 | 0.8231416 | 2.54E-275 | positive |
| CFLAR | AC003681.1 | 0.44980057 | 1.55E-56 | positive |
| BRAF | AC003681.1 | 0.746064912 | 1.97E-198 | positive |
| ATRX | AC003681.1 | 0.491123675 | 1.25E-68 | positive |
| OTULIN | AC016727.3 | 0.416580581 | 5.95E-48 | positive |
| DIABLO | AC016727.3 | 0.731354699 | 7.03E-187 | positive |
| BRAF | AC016727.3 | 0.637932291 | 3.33E-128 | positive |
| ATRX | AC016727.3 | 0.448623658 | 3.24E-56 | positive |
| OTULIN | AL022311.1 | 0.430515279 | 1.95E-51 | positive |
| CYLD | AL022311.1 | 0.405656797 | 2.47E-45 | positive |
| DIABLO | AL022311.1 | 0.803573643 | 1.34E-252 | positive |
| BRAF | AL022311.1 | 0.655606191 | 1.06E-137 | positive |
| ATRX | AL022311.1 | 0.532171696 | 2.11E-82 | positive |
| RNF31 | AL022311.1 | 0.50845378 | 3.24E-74 | positive |
| DIABLO | LRP4-AS1 | 0.440063503 | 6.40E-54 | positive |
| DIABLO | AC093817.1 | 0.469950074 | 3.16E-62 | positive |
| BRAF | AC093817.1 | 0.43548357 | 1.02E-52 | positive |
| DIABLO | AC022211.1 | 0.595232276 | 1.19E-107 | positive |
| CFLAR | AC022211.1 | 0.417163758 | 4.28E-48 | positive |
| BRAF | AC022211.1 | 0.528908005 | 3.08E-81 | positive |
| ATRX | AC022211.1 | 0.477168865 | 2.33E-64 | positive |
| RNF31 | AC022211.1 | 0.437085089 | 3.88E-53 | positive |
| OTULIN | AC129803.1 | 0.472698385 | 4.94E-63 | positive |
| CYLD | AC129803.1 | 0.426379903 | 2.19E-50 | positive |
| DIABLO | AC129803.1 | 0.775297115 | 6.00E-224 | positive |
| BRAF | AC129803.1 | 0.694726312 | 3.52E-161 | positive |
| ATRX | AC129803.1 | 0.410584355 | 1.68E-46 | positive |
| CD40 | AL023653.1 | 0.588660328 | 9.32E-105 | positive |
| DIABLO | AC245014.3 | 0.576074185 | 2.14E-99 | positive |
| BRAF | AC245014.3 | 0.478344096 | 1.04E-64 | positive |
| TSC1 | AL136531.1 | 0.459981185 | 2.31E-59 | positive |
| DIABLO | AL136531.1 | 0.602756713 | 4.76E-111 | positive |
| BRAF | AL136531.1 | 0.559767554 | 8.72E-93 | positive |
| ATRX | AL136531.1 | 0.533147992 | 9.38E-83 | positive |
| RNF31 | AL136531.1 | 0.436042854 | 7.27E-53 | positive |
| DIABLO | FBXO36-IT1 | 0.696112279 | 4.42E-162 | positive |
| BRAF | FBXO36-IT1 | 0.600641648 | 4.39E-110 | positive |
| ATRX | FBXO36-IT1 | 0.46467158 | 1.07E-60 | positive |
| OTULIN | AC005225.1 | 0.410690307 | 1.58E-46 | positive |
| CYLD | AC005225.1 | 0.475542869 | 7.11E-64 | positive |
| DIABLO | AC005225.1 | 0.601070213 | 2.80E-110 | positive |
| BRAF | AC005225.1 | 0.607961652 | 1.88E-113 | positive |
| ATRX | AC005225.1 | 0.525538459 | 4.77E-80 | positive |
| OTULIN | AC122710.2 | 0.400173404 | 4.70E-44 | positive |
| TNFRSF1A | AL355803.1 | 0.488381777 | 8.94E-68 | positive |
| EGFR | AL355803.1 | 0.489956647 | 2.89E-68 | positive |
| OTULIN | AC007014.2 | 0.462517613 | 4.40E-60 | positive |
| CYLD | AC007014.2 | 0.410514575 | 1.74E-46 | positive |
| DIABLO | AC007014.2 | 0.738833361 | 1.18E-192 | positive |
| BRAF | AC007014.2 | 0.641494493 | 4.56E-130 | positive |
| ATRX | AC007014.2 | 0.412741686 | 5.08E-47 | positive |
| MAPK8 | AL021918.4 | 0.417678152 | 3.20E-48 | positive |
| OTULIN | AL021918.4 | 0.482436811 | 6.00E-66 | positive |
| CYLD | AL021918.4 | 0.425277463 | 4.16E-50 | positive |
| DIABLO | AL021918.4 | 0.74448596 | 3.73E-197 | positive |
| BRAF | AL021918.4 | 0.663418132 | 4.19E-142 | positive |
| ATRX | AL021918.4 | 0.435313695 | 1.13E-52 | positive |
| CFLAR | AC005086.2 | 0.452024516 | 3.81E-57 | positive |
| MPG | AC005086.2 | -0.417402381 | 3.74E-48 | negative |
| ATRX | AC005086.2 | 0.587116029 | 4.37E-104 | positive |
| RNF31 | AC005086.2 | 0.493289988 | 2.60E-69 | positive |
| OTULIN | AC116428.1 | 0.458874752 | 4.73E-59 | positive |
| CYLD | AC116428.1 | 0.456568732 | 2.10E-58 | positive |
| DIABLO | AC116428.1 | 0.794971872 | 2.09E-243 | positive |
| BRAF | AC116428.1 | 0.687570491 | 1.32E-156 | positive |
| OTULIN | AL035693.1 | 0.401131714 | 2.82E-44 | positive |
| DIABLO | AL035693.1 | 0.698947001 | 6.10E-164 | positive |
| BRAF | AL035693.1 | 0.555011762 | 6.30E-91 | positive |
| MAPK8 | AC109454.2 | 0.424838509 | 5.36E-50 | positive |
| OTULIN | AC109454.2 | 0.401733811 | 2.04E-44 | positive |
| CYLD | AC109454.2 | 0.440070907 | 6.37E-54 | positive |
| DIABLO | AC109454.2 | 0.703314877 | 7.52E-167 | positive |
| CFLAR | AC109454.2 | 0.436302584 | 6.22E-53 | positive |
| BRAF | AC109454.2 | 0.624576967 | 1.97E-121 | positive |
| ATRX | AC109454.2 | 0.522808987 | 4.29E-79 | positive |
| DIABLO | AC092745.1 | 0.62166658 | 5.34E-120 | positive |
| BRAF | AC092745.1 | 0.56474797 | 9.13E-95 | positive |
| ATRX | AC092745.1 | 0.465538443 | 6.01E-61 | positive |
| STUB1 | AL139246.5 | 0.414735147 | 1.67E-47 | positive |
| BACH2 | U62631.1 | 0.591684157 | 4.43E-106 | positive |
| OTULIN | AC007216.4 | 0.444995925 | 3.10E-55 | positive |
| CYLD | AC007216.4 | 0.438586717 | 1.57E-53 | positive |
| DIABLO | AC007216.4 | 0.750797086 | 2.55E-202 | positive |
| CFLAR | AC007216.4 | 0.462522643 | 4.39E-60 | positive |
| BRAF | AC007216.4 | 0.66013216 | 3.10E-140 | positive |
| ATRX | AC007216.4 | 0.507325174 | 7.65E-74 | positive |
| DIABLO | AC104984.4 | 0.555996558 | 2.61E-91 | positive |
| BRAF | AC104984.4 | 0.492023931 | 6.52E-69 | positive |
| DIABLO | AC097359.2 | 0.431030058 | 1.44E-51 | positive |
| TSC1 | GNG12-AS1 | 0.425257955 | 4.20E-50 | positive |
| MAPK8 | GNG12-AS1 | 0.417433901 | 3.68E-48 | positive |
| OTULIN | GNG12-AS1 | 0.413585709 | 3.18E-47 | positive |
| CYLD | GNG12-AS1 | 0.488762868 | 6.81E-68 | positive |
| MAP3K7 | GNG12-AS1 | 0.404677792 | 4.20E-45 | positive |
| DIABLO | GNG12-AS1 | 0.67692506 | 4.82E-150 | positive |
| CFLAR | GNG12-AS1 | 0.475246405 | 8.71E-64 | positive |
| BRAF | GNG12-AS1 | 0.70208299 | 5.03E-166 | positive |
| ATRX | GNG12-AS1 | 0.513790493 | 5.30E-76 | positive |
| KLF9 | GNG12-AS1 | 0.438680656 | 1.48E-53 | positive |
| DIABLO | SNAP47-AS1 | 0.612597194 | 1.25E-115 | positive |
| BRAF | SNAP47-AS1 | 0.472467667 | 5.78E-63 | positive |
| MAPK8 | AC005052.2 | 0.412170695 | 6.97E-47 | positive |
| CYLD | AC005052.2 | 0.420517505 | 6.40E-49 | positive |
| DIABLO | AC005052.2 | 0.507270441 | 7.98E-74 | positive |
| CFLAR | AC005052.2 | 0.449113177 | 2.39E-56 | positive |
| BRAF | AC005052.2 | 0.516330574 | 7.29E-77 | positive |
| ATRX | AC005052.2 | 0.543191126 | 1.96E-86 | positive |
| OTULIN | AC011468.1 | 0.420611973 | 6.07E-49 | positive |
| DIABLO | AC011468.1 | 0.61631827 | 2.10E-117 | positive |
| BRAF | AC011468.1 | 0.575526675 | 3.62E-99 | positive |
| ATRX | AC011468.1 | 0.411562115 | 9.77E-47 | positive |
| DIABLO | AC244100.2 | 0.494960145 | 7.72E-70 | positive |
| BRAF | AC244100.2 | 0.43606948 | 7.15E-53 | positive |
| TSC1 | AL022328.2 | 0.434635358 | 1.69E-52 | positive |
| RNF31 | AL022328.2 | 0.570816512 | 3.18E-97 | positive |
| CYLD | AP003721.2 | 0.450172644 | 1.23E-56 | positive |
| DIABLO | AP003721.2 | 0.54344488 | 1.58E-86 | positive |
| BRAF | AP003721.2 | 0.481031999 | 1.60E-65 | positive |
| DIABLO | AP000350.8 | 0.529337135 | 2.17E-81 | positive |
| BRAF | AP000350.8 | 0.426269749 | 2.34E-50 | positive |
| GATA3 | AP000350.8 | 0.413439992 | 3.45E-47 | positive |
| ATRX | AP000350.8 | 0.438277331 | 1.89E-53 | positive |
| RNF31 | AP000350.8 | 0.436842133 | 4.50E-53 | positive |
| OTULIN | LINC00862 | 0.455462558 | 4.26E-58 | positive |
| DIABLO | LINC00862 | 0.477444075 | 1.93E-64 | positive |
| BRAF | LINC00862 | 0.454376202 | 8.54E-58 | positive |
| OTULIN | AL133260.2 | 0.428928541 | 4.95E-51 | positive |
| CYLD | AL133260.2 | 0.405337437 | 2.94E-45 | positive |
| DIABLO | AL133260.2 | 0.8123606 | 1.80E-262 | positive |
| BRAF | AL133260.2 | 0.68651396 | 6.08E-156 | positive |
| ATRX | AL133260.2 | 0.405414194 | 2.82E-45 | positive |
| MAPK8 | TRAF3IP2-AS1 | 0.436416005 | 5.81E-53 | positive |
| OTULIN | TRAF3IP2-AS1 | 0.527017832 | 1.44E-80 | positive |
| CYLD | TRAF3IP2-AS1 | 0.490526991 | 1.92E-68 | positive |
| MAP3K7 | TRAF3IP2-AS1 | 0.535692731 | 1.13E-83 | positive |
| DIABLO | TRAF3IP2-AS1 | 0.720940141 | 3.75E-179 | positive |
| CFLAR | TRAF3IP2-AS1 | 0.430741215 | 1.71E-51 | positive |
| BRAF | TRAF3IP2-AS1 | 0.694994373 | 2.36E-161 | positive |
| ATRX | TRAF3IP2-AS1 | 0.470370542 | 2.38E-62 | positive |
| MAPK8 | PCBP2-OT1 | 0.404260621 | 5.26E-45 | positive |
| OTULIN | PCBP2-OT1 | 0.424758919 | 5.61E-50 | positive |
| DIABLO | PCBP2-OT1 | 0.705298978 | 3.44E-168 | positive |
| BRAF | PCBP2-OT1 | 0.597777406 | 8.63E-109 | positive |
| OTULIN | AC097382.1 | 0.411576654 | 9.69E-47 | positive |
| DIABLO | AC097382.1 | 0.756263647 | 6.37E-207 | positive |
| BRAF | AC097382.1 | 0.630539346 | 2.06E-124 | positive |
| CYLD | AC010468.1 | 0.415095626 | 1.37E-47 | positive |
| DIABLO | AC010468.1 | 0.588905443 | 7.29E-105 | positive |
| BRAF | AC010468.1 | 0.536983743 | 3.82E-84 | positive |
| DIABLO | AL135926.2 | 0.466074113 | 4.21E-61 | positive |
| DIABLO | AL022323.2 | 0.778654528 | 3.98E-227 | positive |
| BRAF | AL022323.2 | 0.605880209 | 1.74E-112 | positive |
| ATRX | AL022323.2 | 0.428658418 | 5.80E-51 | positive |
| DIABLO | AC103858.2 | 0.450656232 | 9.04E-57 | positive |
| DIABLO | AL032819.1 | 0.487636739 | 1.52E-67 | positive |
| DIABLO | AC020911.1 | 0.615951495 | 3.14E-117 | positive |
| BRAF | AC020911.1 | 0.54422092 | 8.10E-87 | positive |
| OTULIN | ERCC8-AS1 | 0.41262454 | 5.42E-47 | positive |
| CYLD | ERCC8-AS1 | 0.403200855 | 9.31E-45 | positive |
| DIABLO | ERCC8-AS1 | 0.689903693 | 4.40E-158 | positive |
| BRAF | ERCC8-AS1 | 0.654650151 | 3.60E-137 | positive |
| ATRX | ERCC8-AS1 | 0.445926436 | 1.74E-55 | positive |
| DIABLO | AC108861.1 | 0.685690905 | 1.99E-155 | positive |
| BRAF | AC108861.1 | 0.557516581 | 6.67E-92 | positive |
| SPATA2 | AL137077.2 | 0.481760992 | 9.63E-66 | positive |
| OTULIN | AP001029.2 | 0.443286427 | 8.90E-55 | positive |
| DIABLO | AP001029.2 | 0.657773119 | 6.58E-139 | positive |
| BRAF | AP001029.2 | 0.569779728 | 8.44E-97 | positive |
| ATRX | AP001029.2 | 0.407010946 | 1.19E-45 | positive |
| OTULIN | AC027514.2 | 0.478038036 | 1.28E-64 | positive |
| CYLD | AC027514.2 | 0.450258943 | 1.16E-56 | positive |
| DIABLO | AC027514.2 | 0.749482563 | 3.12E-201 | positive |
| BRAF | AC027514.2 | 0.663742812 | 2.73E-142 | positive |
| DIABLO | AL031666.2 | 0.778126796 | 1.27E-226 | positive |
| CFLAR | AL031666.2 | 0.416415702 | 6.53E-48 | positive |
| BRAF | AL031666.2 | 0.647568798 | 2.65E-133 | positive |
| ATRX | AL031666.2 | 0.510394019 | 7.32E-75 | positive |
| OTULIN | AL136146.2 | 0.441798163 | 2.22E-54 | positive |
| DIABLO | AL136146.2 | 0.720267609 | 1.15E-178 | positive |
| BRAF | AL136146.2 | 0.650078479 | 1.16E-134 | positive |
| OTULIN | AC009365.3 | 0.446794778 | 1.02E-55 | positive |
| DIABLO | AC009365.3 | 0.802405864 | 2.53E-251 | positive |
| BRAF | AC009365.3 | 0.67324992 | 7.68E-148 | positive |
| AXL | AP000695.2 | 0.433560187 | 3.21E-52 | positive |
| KLF9 | AC103740.1 | 0.461657848 | 7.73E-60 | positive |
| DIABLO | AL139021.1 | 0.412575337 | 5.57E-47 | positive |
| CYLD | AC092338.2 | 0.452654496 | 2.55E-57 | positive |
| DIABLO | AC092338.2 | 0.756140955 | 8.10E-207 | positive |
| CFLAR | AC092338.2 | 0.515895059 | 1.03E-76 | positive |
| BRAF | AC092338.2 | 0.663800257 | 2.53E-142 | positive |
| ATRX | AC092338.2 | 0.592064981 | 3.01E-106 | positive |
| OTULIN | AP003783.1 | 0.442323606 | 1.61E-54 | positive |
| DIABLO | AP003783.1 | 0.765617644 | 4.36E-215 | positive |
| BRAF | AP003783.1 | 0.62960682 | 6.08E-124 | positive |
| ATRX | AP003783.1 | 0.400193943 | 4.65E-44 | positive |
| OTULIN | LINC02100 | 0.406473572 | 1.59E-45 | positive |
| DIABLO | LINC02100 | 0.401936481 | 1.83E-44 | positive |
| OTULIN | AC004765.1 | 0.490685439 | 1.71E-68 | positive |
| CYLD | AC004765.1 | 0.45015382 | 1.24E-56 | positive |
| DIABLO | AC004765.1 | 0.777198669 | 9.66E-226 | positive |
| BRAF | AC004765.1 | 0.694579642 | 4.38E-161 | positive |
| ATRX | AC004765.1 | 0.40455568 | 4.49E-45 | positive |
| CYLD | ZBED3-AS1 | 0.430945085 | 1.51E-51 | positive |
| DIABLO | ZBED3-AS1 | 0.658830645 | 1.68E-139 | positive |
| BRAF | ZBED3-AS1 | 0.637997984 | 3.08E-128 | positive |
| ATRX | ZBED3-AS1 | 0.535665033 | 1.15E-83 | positive |
| PANX1 | AC134312.5 | 0.416037427 | 8.07E-48 | positive |
| DIABLO | DNM3-IT1 | 0.546927917 | 7.79E-88 | positive |
| OTULIN | AL354696.2 | 0.421243182 | 4.23E-49 | positive |
| MAP3K7 | AL354696.2 | 0.44286213 | 1.16E-54 | positive |
| DIABLO | AL354696.2 | 0.554560731 | 9.42E-91 | positive |
| BRAF | AL354696.2 | 0.574403822 | 1.06E-98 | positive |
| ATRX | AL354696.2 | 0.469499488 | 4.28E-62 | positive |
| RIPK3 | AC009065.2 | 0.404445442 | 4.76E-45 | positive |
| STUB1 | AC009065.2 | 0.653256482 | 2.12E-136 | positive |
| MPG | AC009065.2 | 0.591232653 | 6.99E-106 | positive |
| ATRX | AC009065.2 | -0.459270271 | 3.66E-59 | negative |
| CYLD | AC107959.1 | 0.474413826 | 1.54E-63 | positive |
| CFLAR | AC107959.1 | 0.511339683 | 3.53E-75 | positive |
| AXL | AC107959.1 | 0.479666435 | 4.14E-65 | positive |
| ATRX | AC107959.1 | 0.462814794 | 3.62E-60 | positive |
| KLF9 | AC107959.1 | 0.706552383 | 4.84E-169 | positive |
| DIABLO | AC009075.1 | 0.573194921 | 3.35E-98 | positive |
| BRAF | AC009075.1 | 0.574846506 | 6.94E-99 | positive |
| ATRX | AC009075.1 | 0.439253256 | 1.05E-53 | positive |
| DIABLO | AC107982.2 | 0.488380597 | 8.95E-68 | positive |
| BRAF | AC107982.2 | 0.407828597 | 7.59E-46 | positive |
| ATRX | AC107982.2 | 0.486174549 | 4.30E-67 | positive |
| OTULIN | AC036214.4 | 0.464924728 | 9.02E-61 | positive |
| DIABLO | AC036214.4 | 0.713862708 | 4.22E-174 | positive |
| BRAF | AC036214.4 | 0.658903191 | 1.53E-139 | positive |
| OTULIN | BACH1-IT3 | 0.420651104 | 5.93E-49 | positive |
| CYLD | BACH1-IT3 | 0.413233123 | 3.87E-47 | positive |
| DIABLO | BACH1-IT3 | 0.690411435 | 2.09E-158 | positive |
| CFLAR | BACH1-IT3 | 0.407195682 | 1.07E-45 | positive |
| BRAF | BACH1-IT3 | 0.609328386 | 4.33E-114 | positive |
| ATRX | BACH1-IT3 | 0.427493092 | 1.15E-50 | positive |
| DIABLO | FRY-AS1 | 0.468357707 | 9.20E-62 | positive |
| BRAF | FRY-AS1 | 0.400862959 | 3.25E-44 | positive |
| OTULIN | AC117500.2 | 0.470730099 | 1.87E-62 | positive |
| CYLD | AC117500.2 | 0.484924324 | 1.04E-66 | positive |
| DIABLO | AC117500.2 | 0.833925638 | 4.41E-289 | positive |
| CFLAR | AC117500.2 | 0.475525571 | 7.20E-64 | positive |
| BRAF | AC117500.2 | 0.733716657 | 1.11E-188 | positive |
| ATRX | AC117500.2 | 0.518537039 | 1.28E-77 | positive |
| DIABLO | AC013452.2 | 0.524269004 | 1.33E-79 | positive |
| BRAF | AC013452.2 | 0.431516355 | 1.08E-51 | positive |
| DIABLO | SEMA3F-AS1 | 0.58493617 | 3.81E-103 | positive |
| BRAF | SEMA3F-AS1 | 0.437726049 | 2.64E-53 | positive |
| RNF31 | SEMA3F-AS1 | 0.443039957 | 1.04E-54 | positive |
| DIABLO | AC104695.3 | 0.65394706 | 8.82E-137 | positive |
| BRAF | AC104695.3 | 0.585858269 | 1.53E-103 | positive |
| KLF9 | AC016590.4 | 0.409854722 | 2.50E-46 | positive |
| MAPK8 | PLCG1-AS1 | 0.434593562 | 1.73E-52 | positive |
| IPMK | PLCG1-AS1 | 0.420489817 | 6.50E-49 | positive |
| OTULIN | PLCG1-AS1 | 0.514101634 | 4.16E-76 | positive |
| CYLD | PLCG1-AS1 | 0.484648453 | 1.27E-66 | positive |
| MAP3K7 | PLCG1-AS1 | 0.427875866 | 9.17E-51 | positive |
| DIABLO | PLCG1-AS1 | 0.821571691 | 2.14E-273 | positive |
| CFLAR | PLCG1-AS1 | 0.460577619 | 1.56E-59 | positive |
| BRAF | PLCG1-AS1 | 0.762414734 | 3.00E-212 | positive |
| ATRX | PLCG1-AS1 | 0.514990731 | 2.08E-76 | positive |
| OTULIN | AC026725.1 | 0.410896491 | 1.41E-46 | positive |
| CYLD | AC026725.1 | 0.441598555 | 2.51E-54 | positive |
| DIABLO | AC026725.1 | 0.762959328 | 9.95E-213 | positive |
| BRAF | AC026725.1 | 0.677981665 | 1.11E-150 | positive |
| KLF9 | ZNF710-AS1 | 0.459228858 | 3.76E-59 | positive |
| DIABLO | AC025280.1 | 0.580596866 | 2.70E-101 | positive |
| BRAF | AC025280.1 | 0.54529093 | 3.22E-87 | positive |
| CYLD | CEP250-AS1 | 0.466432132 | 3.32E-61 | positive |
| DIABLO | CEP250-AS1 | 0.544551521 | 6.09E-87 | positive |
| CFLAR | CEP250-AS1 | 0.436135582 | 6.88E-53 | positive |
| BRAF | CEP250-AS1 | 0.485503666 | 6.92E-67 | positive |
| BCL2 | CEP250-AS1 | 0.416551473 | 6.05E-48 | positive |
| BACH2 | CEP250-AS1 | 0.433616513 | 3.10E-52 | positive |
| ATRX | CEP250-AS1 | 0.42716487 | 1.39E-50 | positive |
| CD40 | CEP250-AS1 | 0.4356686 | 9.10E-53 | positive |
| MAPK8 | AP001350.1 | 0.438797061 | 1.38E-53 | positive |
| IPMK | AP001350.1 | 0.41542411 | 1.14E-47 | positive |
| OTULIN | AP001350.1 | 0.526899245 | 1.58E-80 | positive |
| CYLD | AP001350.1 | 0.482023283 | 8.02E-66 | positive |
| MAP3K7 | AP001350.1 | 0.419802395 | 9.62E-49 | positive |
| DIABLO | AP001350.1 | 0.771493061 | 2.05E-220 | positive |
| CFLAR | AP001350.1 | 0.410502208 | 1.75E-46 | positive |
| BRAF | AP001350.1 | 0.713337784 | 9.87E-174 | positive |
| ATRX | AP001350.1 | 0.449232139 | 2.21E-56 | positive |
| OTULIN | AF131216.1 | 0.41960357 | 1.08E-48 | positive |
| CYLD | AF131216.1 | 0.424805799 | 5.46E-50 | positive |
| DIABLO | AF131216.1 | 0.656932275 | 1.94E-138 | positive |
| CFLAR | AF131216.1 | 0.442824931 | 1.18E-54 | positive |
| BRAF | AF131216.1 | 0.627542914 | 6.60E-123 | positive |
| ATRX | AF131216.1 | 0.463834158 | 1.85E-60 | positive |
| ATRX | AC060766.4 | 0.477467977 | 1.90E-64 | positive |
| SIRT3 | AC097639.1 | 0.409641587 | 2.81E-46 | positive |
| SPATA2 | AC097639.1 | 0.402681167 | 1.23E-44 | positive |
| MAPK8 | AL008730.1 | 0.42072285 | 5.70E-49 | positive |
| OTULIN | AL008730.1 | 0.477931007 | 1.38E-64 | positive |
| CYLD | AL008730.1 | 0.465344025 | 6.83E-61 | positive |
| DIABLO | AL008730.1 | 0.793380111 | 9.38E-242 | positive |
| CFLAR | AL008730.1 | 0.413073591 | 4.23E-47 | positive |
| BRAF | AL008730.1 | 0.716589121 | 4.99E-176 | positive |
| ATRX | AL008730.1 | 0.458150364 | 7.56E-59 | positive |
| DIABLO | AC005599.1 | 0.436019346 | 7.37E-53 | positive |
| BRAF | AC005599.1 | 0.435643754 | 9.24E-53 | positive |
| TSC1 | LINC01772 | 0.437243389 | 3.53E-53 | positive |
| CYLD | LINC01772 | 0.469481059 | 4.33E-62 | positive |
| DIABLO | LINC01772 | 0.600458471 | 5.31E-110 | positive |
| CFLAR | LINC01772 | 0.54305742 | 2.20E-86 | positive |
| BRAF | LINC01772 | 0.554387336 | 1.10E-90 | positive |
| BACH2 | LINC01772 | 0.401870667 | 1.90E-44 | positive |
| ATRX | LINC01772 | 0.643966755 | 2.24E-131 | positive |
| RNF31 | LINC01772 | 0.570694878 | 3.57E-97 | positive |
| OTULIN | DIAPH1-AS1 | 0.453637515 | 1.37E-57 | positive |
| CYLD | DIAPH1-AS1 | 0.40954018 | 2.98E-46 | positive |
| DIABLO | DIAPH1-AS1 | 0.807024694 | 2.05E-256 | positive |
| BRAF | DIAPH1-AS1 | 0.678628552 | 4.48E-151 | positive |
| OTULIN | AC025370.2 | 0.422883006 | 1.65E-49 | positive |
| CYLD | AC025370.2 | 0.457295798 | 1.31E-58 | positive |
| DIABLO | AC025370.2 | 0.618442941 | 1.98E-118 | positive |
| BRAF | AC025370.2 | 0.584902859 | 3.93E-103 | positive |
| ATRX | AC025370.2 | 0.483651905 | 2.56E-66 | positive |
| RNF31 | AC025370.2 | 0.430399597 | 2.09E-51 | positive |
| CYLD | AL353586.1 | 0.406743468 | 1.37E-45 | positive |
| DIABLO | AL353586.1 | 0.68717833 | 2.33E-156 | positive |
| BRAF | AL353586.1 | 0.58298713 | 2.60E-102 | positive |
| SIRT3 | PPP1R26-AS1 | 0.416059397 | 7.97E-48 | positive |
| TRAF2 | PPP1R26-AS1 | 0.40509494 | 3.35E-45 | positive |
| OTULIN | AL450226.1 | 0.436515263 | 5.47E-53 | positive |
| DIABLO | AL450226.1 | 0.73831048 | 3.03E-192 | positive |
| BRAF | AL450226.1 | 0.607338214 | 3.67E-113 | positive |
| DIABLO | AC009242.1 | 0.577142798 | 7.67E-100 | positive |
| BRAF | AC009242.1 | 0.523381435 | 2.71E-79 | positive |
| TSC1 | LINC00863 | 0.421967656 | 2.80E-49 | positive |
| MAPK8 | LINC00863 | 0.414433072 | 1.98E-47 | positive |
| CYLD | LINC00863 | 0.428752893 | 5.49E-51 | positive |
| CFLAR | LINC00863 | 0.433059922 | 4.32E-52 | positive |
| BRAF | LINC00863 | 0.479268809 | 5.46E-65 | positive |
| ATRX | LINC00863 | 0.564346924 | 1.32E-94 | positive |
| KLF9 | LINC00863 | 0.430922579 | 1.53E-51 | positive |
| BCL2L11 | LINC00863 | 0.41008606 | 2.20E-46 | positive |
| MAPK8 | SOS1-IT1 | 0.461716104 | 7.44E-60 | positive |
| IPMK | SOS1-IT1 | 0.416440774 | 6.43E-48 | positive |
| OTULIN | SOS1-IT1 | 0.562093048 | 1.05E-93 | positive |
| CYLD | SOS1-IT1 | 0.492947485 | 3.34E-69 | positive |
| MAP3K7 | SOS1-IT1 | 0.481348588 | 1.28E-65 | positive |
| DIABLO | SOS1-IT1 | 0.730260341 | 4.74E-186 | positive |
| CFLAR | SOS1-IT1 | 0.438221363 | 1.96E-53 | positive |
| BRAF | SOS1-IT1 | 0.740769705 | 3.49E-194 | positive |
| ATRX | SOS1-IT1 | 0.510639855 | 6.06E-75 | positive |
| MAPK8 | AC012358.1 | 0.411758492 | 8.76E-47 | positive |
| OTULIN | AC012358.1 | 0.45507764 | 5.45E-58 | positive |
| CYLD | AC012358.1 | 0.453738648 | 1.28E-57 | positive |
| MAP3K7 | AC012358.1 | 0.411164708 | 1.22E-46 | positive |
| DIABLO | AC012358.1 | 0.727599418 | 4.73E-184 | positive |
| CFLAR | AC012358.1 | 0.464557887 | 1.15E-60 | positive |
| BRAF | AC012358.1 | 0.682218863 | 2.85E-153 | positive |
| ATRX | AC012358.1 | 0.526094921 | 3.04E-80 | positive |
| DIABLO | RRM1-AS1 | 0.41261447 | 5.45E-47 | positive |
| OTULIN | AC127164.1 | 0.441228065 | 3.14E-54 | positive |
| CYLD | AC127164.1 | 0.421074067 | 4.66E-49 | positive |
| DIABLO | AC127164.1 | 0.817564459 | 1.44E-268 | positive |
| BRAF | AC127164.1 | 0.699494054 | 2.65E-164 | positive |
| ATRX | AC127164.1 | 0.431503103 | 1.09E-51 | positive |
| DIABLO | AP002762.2 | 0.705882347 | 1.38E-168 | positive |
| BRAF | AP002762.2 | 0.574483108 | 9.83E-99 | positive |
| OTULIN | AC034102.5 | 0.404627097 | 4.32E-45 | positive |
| DIABLO | AC034102.5 | 0.665673524 | 2.11E-143 | positive |
| BRAF | AC034102.5 | 0.588224093 | 1.44E-104 | positive |
| ATRX | AC034102.5 | 0.463521269 | 2.28E-60 | positive |
| DIABLO | GK-AS1 | 0.562464663 | 7.45E-94 | positive |
| BRAF | GK-AS1 | 0.535597895 | 1.22E-83 | positive |
| DIABLO | AP002892.2 | 0.647813592 | 1.96E-133 | positive |
| BRAF | AP002892.2 | 0.573744167 | 1.99E-98 | positive |
| DIABLO | AC087761.1 | 0.468610773 | 7.77E-62 | positive |
| MAPK8 | RAB30-DT | 0.406302507 | 1.74E-45 | positive |
| OTULIN | RAB30-DT | 0.424053282 | 8.43E-50 | positive |
| DIABLO | RAB30-DT | 0.646045862 | 1.74E-132 | positive |
| BRAF | RAB30-DT | 0.620508024 | 1.97E-119 | positive |
| ATRX | RAB30-DT | 0.463725846 | 1.99E-60 | positive |
| DIABLO | AC011383.1 | 0.565460604 | 4.73E-95 | positive |
| BRAF | AC011383.1 | 0.47296277 | 4.13E-63 | positive |
| OTULIN | BACH1-IT2 | 0.457433453 | 1.20E-58 | positive |
| CYLD | BACH1-IT2 | 0.436036398 | 7.30E-53 | positive |
| DIABLO | BACH1-IT2 | 0.74198934 | 3.75E-195 | positive |
| CFLAR | BACH1-IT2 | 0.410432276 | 1.82E-46 | positive |
| BRAF | BACH1-IT2 | 0.656686439 | 2.66E-138 | positive |
| ATRX | BACH1-IT2 | 0.409746979 | 2.66E-46 | positive |
| DIABLO | AC007879.1 | 0.593734309 | 5.51E-107 | positive |
| BRAF | AC007879.1 | 0.530004921 | 1.25E-81 | positive |
| DIABLO | AP000317.1 | 0.435225386 | 1.19E-52 | positive |
| BRAF | AP000317.1 | 0.413411839 | 3.50E-47 | positive |
| TSC1 | AC092653.1 | 0.430965081 | 1.49E-51 | positive |
| MAPK8 | AC092653.1 | 0.436995153 | 4.10E-53 | positive |
| IPMK | AC092653.1 | 0.422540969 | 2.01E-49 | positive |
| OTULIN | AC092653.1 | 0.491366851 | 1.05E-68 | positive |
| CYLD | AC092653.1 | 0.536119002 | 7.88E-84 | positive |
| MAP3K7 | AC092653.1 | 0.406303136 | 1.74E-45 | positive |
| DIABLO | AC092653.1 | 0.777761543 | 2.82E-226 | positive |
| CFLAR | AC092653.1 | 0.527020122 | 1.44E-80 | positive |
| BRAF | AC092653.1 | 0.740909997 | 2.70E-194 | positive |
| ATRX | AC092653.1 | 0.588173553 | 1.52E-104 | positive |
| OTULIN | AL157938.3 | 0.468615761 | 7.74E-62 | positive |
| CYLD | AL157938.3 | 0.416659837 | 5.69E-48 | positive |
| DIABLO | AL157938.3 | 0.803853186 | 6.64E-253 | positive |
| BRAF | AL157938.3 | 0.700164185 | 9.55E-165 | positive |
| ATRX | AL157938.3 | 0.418588911 | 1.92E-48 | positive |
| MAPK8 | USP3-AS1 | 0.426859628 | 1.66E-50 | positive |
| IPMK | USP3-AS1 | 0.403036721 | 1.02E-44 | positive |
| OTULIN | USP3-AS1 | 0.472474783 | 5.75E-63 | positive |
| CYLD | USP3-AS1 | 0.451519443 | 5.24E-57 | positive |
| DIABLO | USP3-AS1 | 0.811334576 | 2.72E-261 | positive |
| CFLAR | USP3-AS1 | 0.424046055 | 8.47E-50 | positive |
| BRAF | USP3-AS1 | 0.718528252 | 2.05E-177 | positive |
| ATRX | USP3-AS1 | 0.444488844 | 4.24E-55 | positive |
| DIABLO | LINC00299 | 0.514541165 | 2.95E-76 | positive |
| BRAF | LINC00299 | 0.458536411 | 5.89E-59 | positive |
| DIABLO | AL022069.1 | 0.473247415 | 3.41E-63 | positive |
| DIABLO | AC012360.1 | 0.661047996 | 9.38E-141 | positive |
| BRAF | AC012360.1 | 0.552169994 | 7.87E-90 | positive |
| MAPK8 | DLEU1 | 0.408716444 | 4.67E-46 | positive |
| IPMK | DLEU1 | 0.40662468 | 1.46E-45 | positive |
| OTULIN | DLEU1 | 0.474408963 | 1.54E-63 | positive |
| MAP3K7 | DLEU1 | 0.444332615 | 4.67E-55 | positive |
| DIABLO | DLEU1 | 0.699688853 | 1.97E-164 | positive |
| BRAF | DLEU1 | 0.637392607 | 6.35E-128 | positive |
| ATRX | DLEU1 | 0.401123772 | 2.83E-44 | positive |
| OTULIN | SNHG26 | 0.451197494 | 6.42E-57 | positive |
| DIABLO | SNHG26 | 0.41722176 | 4.15E-48 | positive |
| BRAF | SNHG26 | 0.410161027 | 2.12E-46 | positive |
| ATRX | SNHG26 | 0.423560856 | 1.12E-49 | positive |
| CYLD | AP001363.1 | 0.415856059 | 8.94E-48 | positive |
| DIABLO | AP001363.1 | 0.653488637 | 1.58E-136 | positive |
| CFLAR | AP001363.1 | 0.488488595 | 8.28E-68 | positive |
| BRAF | AP001363.1 | 0.558183818 | 3.65E-92 | positive |
| ATRX | AP001363.1 | 0.451214274 | 6.36E-57 | positive |
| TSC1 | Z97832.2 | 0.511865417 | 2.36E-75 | positive |
| MAPK8 | Z97832.2 | 0.416811576 | 5.22E-48 | positive |
| OTULIN | Z97832.2 | 0.451906583 | 4.10E-57 | positive |
| CYLD | Z97832.2 | 0.479869153 | 3.60E-65 | positive |
| MAP3K7 | Z97832.2 | 0.430556126 | 1.90E-51 | positive |
| DIABLO | Z97832.2 | 0.659000913 | 1.35E-139 | positive |
| CFLAR | Z97832.2 | 0.501183744 | 7.81E-72 | positive |
| BRAF | Z97832.2 | 0.644472508 | 1.21E-131 | positive |
| ATRX | Z97832.2 | 0.556157452 | 2.26E-91 | positive |
| RNF31 | Z97832.2 | 0.414247866 | 2.20E-47 | positive |
| SIRT3 | AL109627.1 | 0.408845158 | 4.36E-46 | positive |
| OTULIN | AC023590.1 | 0.469443397 | 4.45E-62 | positive |
| DIABLO | AC023590.1 | 0.681660181 | 6.29E-153 | positive |
| BRAF | AC023590.1 | 0.575068933 | 5.61E-99 | positive |
| DIABLO | ARRDC3-AS1 | 0.72577598 | 1.07E-182 | positive |
| BRAF | ARRDC3-AS1 | 0.571035156 | 2.59E-97 | positive |
| KLF9 | CADM3-AS1 | 0.530023592 | 1.24E-81 | positive |
| DIABLO | AC127024.3 | 0.562863421 | 5.17E-94 | positive |
| BRAF | AC127024.3 | 0.476217626 | 4.48E-64 | positive |
| DIABLO | AC010273.2 | 0.470727262 | 1.87E-62 | positive |
| BRAF | AC010273.2 | 0.40895557 | 4.10E-46 | positive |
| CYLD | AC096667.1 | 0.490101229 | 2.61E-68 | positive |
| CFLAR | AC096667.1 | 0.425580271 | 3.49E-50 | positive |
| DIABLO | AP003354.1 | 0.575588996 | 3.41E-99 | positive |
| BRAF | AP003354.1 | 0.457653714 | 1.04E-58 | positive |
| ATRX | AP003354.1 | 0.477937522 | 1.37E-64 | positive |
| RNF31 | AP003354.1 | 0.404951351 | 3.63E-45 | positive |
| DIABLO | AC005546.1 | 0.555068805 | 5.99E-91 | positive |
| BRAF | AC005546.1 | 0.446980404 | 9.05E-56 | positive |
| RNF31 | AC005546.1 | 0.423377094 | 1.25E-49 | positive |
| TSC1 | MRPS9-AS1 | 0.460806115 | 1.35E-59 | positive |
| MAPK8 | MRPS9-AS1 | 0.427591744 | 1.08E-50 | positive |
| OTULIN | MRPS9-AS1 | 0.487045274 | 2.32E-67 | positive |
| CYLD | MRPS9-AS1 | 0.425321813 | 4.05E-50 | positive |
| MAP3K7 | MRPS9-AS1 | 0.429331412 | 3.91E-51 | positive |
| DIABLO | MRPS9-AS1 | 0.666932863 | 3.95E-144 | positive |
| BRAF | MRPS9-AS1 | 0.622260924 | 2.73E-120 | positive |
| ATRX | MRPS9-AS1 | 0.449312887 | 2.10E-56 | positive |
| RNF31 | MRPS9-AS1 | 0.411779091 | 8.66E-47 | positive |
| DIABLO | AC017002.2 | 0.417560529 | 3.42E-48 | positive |
| OTULIN | AC055733.2 | 0.427098116 | 1.44E-50 | positive |
| CYLD | AC055733.2 | 0.410965215 | 1.36E-46 | positive |
| DIABLO | AC055733.2 | 0.750798097 | 2.54E-202 | positive |
| BRAF | AC055733.2 | 0.621962013 | 3.83E-120 | positive |
| DIABLO | AC073332.1 | 0.566458733 | 1.87E-95 | positive |
| BRAF | AC073332.1 | 0.509854304 | 1.11E-74 | positive |
| ATRX | AC073332.1 | 0.449550235 | 1.81E-56 | positive |
| OTULIN | AC134775.1 | 0.40154776 | 2.26E-44 | positive |
| DIABLO | AC134775.1 | 0.688941088 | 1.80E-157 | positive |
| CFLAR | AC134775.1 | 0.409238969 | 3.51E-46 | positive |
| BRAF | AC134775.1 | 0.611696645 | 3.33E-115 | positive |
| ATRX | AC134775.1 | 0.408550241 | 5.12E-46 | positive |
| DIABLO | AC023794.1 | 0.5386286 | 9.54E-85 | positive |
| BRAF | AC023794.1 | 0.47631772 | 4.18E-64 | positive |
| MAPK8 | AC087854.1 | 0.405860039 | 2.22E-45 | positive |
| OTULIN | AC087854.1 | 0.453811412 | 1.22E-57 | positive |
| CYLD | AC087854.1 | 0.498184954 | 7.23E-71 | positive |
| DIABLO | AC087854.1 | 0.753352547 | 1.86E-204 | positive |
| CFLAR | AC087854.1 | 0.467417045 | 1.72E-61 | positive |
| BRAF | AC087854.1 | 0.691895192 | 2.35E-159 | positive |
| ATRX | AC087854.1 | 0.474378002 | 1.58E-63 | positive |
| IPMK | AP005432.1 | 0.401858305 | 1.91E-44 | positive |
| OTULIN | AP005432.1 | 0.449890165 | 1.46E-56 | positive |
| CYLD | AP005432.1 | 0.438952919 | 1.26E-53 | positive |
| DIABLO | AP005432.1 | 0.793019382 | 2.21E-241 | positive |
| BRAF | AP005432.1 | 0.696889119 | 1.37E-162 | positive |
| ATRX | AP005432.1 | 0.468025036 | 1.15E-61 | positive |
| DIABLO | AC245297.3 | 0.45841736 | 6.36E-59 | positive |
| BRAF | AC245297.3 | 0.470477651 | 2.22E-62 | positive |
| ATRX | AC245297.3 | 0.431182675 | 1.31E-51 | positive |
| DIABLO | AC015813.1 | 0.569356222 | 1.26E-96 | positive |
| BRAF | AC015813.1 | 0.51412892 | 4.07E-76 | positive |
| DIABLO | ARHGAP27P1-BPTFP1-KPNA2P3 | 0.411596892 | 9.58E-47 | positive |
| DIABLO | AC105916.1 | 0.651360989 | 2.32E-135 | positive |
| BRAF | AC105916.1 | 0.58917719 | 5.55E-105 | positive |
| ATRX | AC105916.1 | 0.432458094 | 6.18E-52 | positive |
| DIABLO | AL358777.3 | 0.423763667 | 9.97E-50 | positive |
| BRAF | AL358777.3 | 0.421726628 | 3.21E-49 | positive |
| OTULIN | ALG9-IT1 | 0.444620478 | 3.91E-55 | positive |
| CYLD | ALG9-IT1 | 0.410894474 | 1.41E-46 | positive |
| DIABLO | ALG9-IT1 | 0.771795738 | 1.08E-220 | positive |
| BRAF | ALG9-IT1 | 0.68394663 | 2.43E-154 | positive |
| ATRX | ALG9-IT1 | 0.413778661 | 2.85E-47 | positive |
| ATRX | LINC02021 | 0.405151533 | 3.25E-45 | positive |
| CD40 | AC074131.1 | 0.614828071 | 1.08E-116 | positive |
| DIABLO | AC004893.2 | 0.430693652 | 1.75E-51 | positive |
| OTULIN | AC117503.5 | 0.45358574 | 1.41E-57 | positive |
| CYLD | AC117503.5 | 0.416904172 | 4.96E-48 | positive |
| DIABLO | AC117503.5 | 0.802865369 | 7.99E-252 | positive |
| BRAF | AC117503.5 | 0.652637751 | 4.64E-136 | positive |
| DIABLO | FNDC1-IT1 | 0.443467011 | 7.97E-55 | positive |
| BRAF | FNDC1-IT1 | 0.471516539 | 1.10E-62 | positive |
| OTULIN | AC092120.3 | 0.445215709 | 2.71E-55 | positive |
| CYLD | AC092120.3 | 0.40584128 | 2.24E-45 | positive |
| DIABLO | AC092120.3 | 0.767095015 | 2.06E-216 | positive |
| BRAF | AC092120.3 | 0.658284972 | 3.40E-139 | positive |
| DIABLO | GRM8-AS1 | 0.582963234 | 2.66E-102 | positive |
| BRAF | GRM8-AS1 | 0.511343978 | 3.52E-75 | positive |
| TARDBP | SNHG20 | 0.411409065 | 1.06E-46 | positive |
| MAPK8 | AC078778.1 | 0.414178588 | 2.28E-47 | positive |
| OTULIN | AC078778.1 | 0.45901335 | 4.32E-59 | positive |
| CYLD | AC078778.1 | 0.41828945 | 2.27E-48 | positive |
| DIABLO | AC078778.1 | 0.801584602 | 1.97E-250 | positive |
| CFLAR | AC078778.1 | 0.412036095 | 7.51E-47 | positive |
| BRAF | AC078778.1 | 0.664686256 | 7.84E-143 | positive |
| ATRX | AC078778.1 | 0.46523697 | 7.33E-61 | positive |
| MAPK8 | PTPRK-AS1 | 0.401464551 | 2.36E-44 | positive |
| OTULIN | PTPRK-AS1 | 0.44796916 | 4.88E-56 | positive |
| CYLD | PTPRK-AS1 | 0.436450391 | 5.69E-53 | positive |
| DIABLO | PTPRK-AS1 | 0.791951647 | 2.77E-240 | positive |
| CFLAR | PTPRK-AS1 | 0.43748525 | 3.05E-53 | positive |
| BRAF | PTPRK-AS1 | 0.690186501 | 2.91E-158 | positive |
| ATRX | PTPRK-AS1 | 0.521417326 | 1.31E-78 | positive |
| KLF9 | AC068733.3 | 0.645976628 | 1.90E-132 | positive |
| OTULIN | AL356752.1 | 0.410276174 | 1.99E-46 | positive |
| DIABLO | AL356752.1 | 0.690157081 | 3.04E-158 | positive |
| CFLAR | AL356752.1 | 0.462698062 | 3.91E-60 | positive |
| BRAF | AL356752.1 | 0.627338592 | 8.36E-123 | positive |
| ATRX | AL356752.1 | 0.479432923 | 4.87E-65 | positive |
| OTULIN | ARHGAP26-IT1 | 0.411030479 | 1.31E-46 | positive |
| DIABLO | ARHGAP26-IT1 | 0.74798211 | 5.36E-200 | positive |
| BRAF | ARHGAP26-IT1 | 0.617111761 | 8.70E-118 | positive |
| DIABLO | ZNRF3-AS1 | 0.675411977 | 3.92E-149 | positive |
| BRAF | ZNRF3-AS1 | 0.564904088 | 7.91E-95 | positive |
| ATRX | ZNRF3-AS1 | 0.415085415 | 1.38E-47 | positive |
| MAPK8 | AL136221.1 | 0.404693713 | 4.17E-45 | positive |
| IPMK | AL136221.1 | 0.424218005 | 7.67E-50 | positive |
| OTULIN | AL136221.1 | 0.446253172 | 1.42E-55 | positive |
| MAP3K7 | AL136221.1 | 0.406911015 | 1.25E-45 | positive |
| DIABLO | AL136221.1 | 0.727853201 | 3.05E-184 | positive |
| BRAF | AL136221.1 | 0.659789235 | 4.84E-140 | positive |
| ATRX | AL136221.1 | 0.406394576 | 1.66E-45 | positive |
| DIABLO | NXT1-AS1 | 0.445551844 | 2.20E-55 | positive |
| DIABLO | LINC00412 | 0.579187896 | 1.06E-100 | positive |
| BRAF | LINC00412 | 0.519461527 | 6.18E-78 | positive |
| DIABLO | AC099314.1 | 0.599978505 | 8.77E-110 | positive |
| BRAF | AC099314.1 | 0.548199269 | 2.58E-88 | positive |
| ATRX | AC099314.1 | 0.477512802 | 1.84E-64 | positive |
| DIABLO | AL592301.1 | 0.599412577 | 1.58E-109 | positive |
| BRAF | AL592301.1 | 0.450893779 | 7.78E-57 | positive |
| DIABLO | AC013714.1 | 0.638778083 | 1.21E-128 | positive |
| BRAF | AC013714.1 | 0.577680533 | 4.57E-100 | positive |
| TSC1 | AC023908.3 | 0.460419913 | 1.73E-59 | positive |
| MAP3K7 | AC023908.3 | 0.408076544 | 6.63E-46 | positive |
| DIABLO | AC023908.3 | 0.460321074 | 1.85E-59 | positive |
| BRAF | AC023908.3 | 0.439448454 | 9.30E-54 | positive |
| DIABLO | AC103923.1 | 0.481789275 | 9.44E-66 | positive |
| BRAF | AC103923.1 | 0.460677658 | 1.47E-59 | positive |
| ATRX | AC103923.1 | 0.433532162 | 3.26E-52 | positive |
| MAPK8 | LINC02605 | 0.417336399 | 3.89E-48 | positive |
| IPMK | LINC02605 | 0.443739201 | 6.74E-55 | positive |
| CYLD | LINC02605 | 0.419975055 | 8.72E-49 | positive |
| DIABLO | LINC02605 | 0.580868019 | 2.07E-101 | positive |
| CFLAR | LINC02605 | 0.499358291 | 3.03E-71 | positive |
| BRAF | LINC02605 | 0.556069762 | 2.45E-91 | positive |
| ATRX | LINC02605 | 0.4042271 | 5.36E-45 | positive |
| TSC1 | AF235103.3 | 0.44933397 | 2.08E-56 | positive |
| DIABLO | AF235103.3 | 0.525726607 | 4.10E-80 | positive |
| BRAF | AF235103.3 | 0.520150077 | 3.58E-78 | positive |
| ATRX | AF235103.3 | 0.453718772 | 1.30E-57 | positive |
| DIABLO | AL159169.3 | 0.648534778 | 7.98E-134 | positive |
| BRAF | AL159169.3 | 0.559906949 | 7.68E-93 | positive |
| BACH2 | AC009133.3 | 0.422588408 | 1.96E-49 | positive |
| MAPK8 | AC099811.1 | 0.407735205 | 7.99E-46 | positive |
| OTULIN | AC099811.1 | 0.46581213 | 5.01E-61 | positive |
| CYLD | AC099811.1 | 0.447830414 | 5.32E-56 | positive |
| DIABLO | AC099811.1 | 0.790666437 | 5.71E-239 | positive |
| BRAF | AC099811.1 | 0.703892995 | 3.07E-167 | positive |
| ATRX | AC099811.1 | 0.427603277 | 1.08E-50 | positive |
| OTULIN | AC011092.2 | 0.456584466 | 2.07E-58 | positive |
| CYLD | AC011092.2 | 0.40742044 | 9.49E-46 | positive |
| DIABLO | AC011092.2 | 0.768204626 | 2.06E-217 | positive |
| BRAF | AC011092.2 | 0.671322268 | 1.07E-146 | positive |
| ATRX | AC011092.2 | 0.413256703 | 3.82E-47 | positive |
| BCL2 | LINC00092 | 0.401924117 | 1.85E-44 | positive |
| BACH2 | LINC00092 | 0.484148437 | 1.80E-66 | positive |
| KLF9 | LINC00092 | 0.462961929 | 3.29E-60 | positive |
| TSC1 | AC145423.2 | 0.445838858 | 1.84E-55 | positive |
| DIABLO | AC145423.2 | 0.592780719 | 1.45E-106 | positive |
| BRAF | AC145423.2 | 0.505029359 | 4.36E-73 | positive |
| ATRX | AC145423.2 | 0.440662468 | 4.44E-54 | positive |
| RNF31 | AC145423.2 | 0.460036603 | 2.22E-59 | positive |
| MAPK8 | AC107081.1 | 0.43455336 | 1.77E-52 | positive |
| OTULIN | AC107081.1 | 0.516869505 | 4.78E-77 | positive |
| MAP3K7 | AC107081.1 | 0.42760448 | 1.07E-50 | positive |
| DIABLO | AC107081.1 | 0.752104467 | 2.07E-203 | positive |
| CFLAR | AC107081.1 | 0.412059821 | 7.41E-47 | positive |
| BRAF | AC107081.1 | 0.640774618 | 1.09E-129 | positive |
| ATRX | AC107081.1 | 0.466946802 | 2.36E-61 | positive |
| RNF31 | AC107081.1 | 0.418340011 | 2.20E-48 | positive |
| CYLD | RBMS3-AS3 | 0.457794987 | 9.51E-59 | positive |
| DIABLO | RBMS3-AS3 | 0.450359079 | 1.09E-56 | positive |
| BRAF | RBMS3-AS3 | 0.46908557 | 5.65E-62 | positive |
| ATRX | RBMS3-AS3 | 0.45040016 | 1.06E-56 | positive |
| KLF9 | RBMS3-AS3 | 0.417993994 | 2.68E-48 | positive |
| ATRX | AC024560.3 | 0.504589399 | 6.08E-73 | positive |
| RNF31 | AC024560.3 | 0.404130524 | 5.65E-45 | positive |
| TSC1 | AC025917.1 | 0.42479075 | 5.51E-50 | positive |
| MAPK8 | AC025917.1 | 0.461719104 | 7.42E-60 | positive |
| IPMK | AC025917.1 | 0.44676542 | 1.03E-55 | positive |
| OTULIN | AC025917.1 | 0.487706247 | 1.45E-67 | positive |
| CYLD | AC025917.1 | 0.612452902 | 1.46E-115 | positive |
| MAP3K7 | AC025917.1 | 0.422017103 | 2.72E-49 | positive |
| DIABLO | AC025917.1 | 0.722156576 | 4.89E-180 | positive |
| CFLAR | AC025917.1 | 0.567480058 | 7.25E-96 | positive |
| BRAF | AC025917.1 | 0.748069776 | 4.54E-200 | positive |
| ATRX | AC025917.1 | 0.596318619 | 3.89E-108 | positive |
| TSC1 | AL008729.1 | 0.42305007 | 1.50E-49 | positive |
| MAPK8 | AL008729.1 | 0.417707493 | 3.15E-48 | positive |
| OTULIN | AL008729.1 | 0.458864548 | 4.76E-59 | positive |
| CYLD | AL008729.1 | 0.429629017 | 3.28E-51 | positive |
| MAP3K7 | AL008729.1 | 0.408333096 | 5.77E-46 | positive |
| DIABLO | AL008729.1 | 0.658519234 | 2.51E-139 | positive |
| CFLAR | AL008729.1 | 0.447684395 | 5.83E-56 | positive |
| BRAF | AL008729.1 | 0.605660673 | 2.20E-112 | positive |
| ATRX | AL008729.1 | 0.430034333 | 2.59E-51 | positive |
| OTULIN | AC068620.2 | 0.404034487 | 5.95E-45 | positive |
| MAP3K7 | AC068620.2 | 0.406205081 | 1.84E-45 | positive |
| DIABLO | AC068620.2 | 0.616504386 | 1.71E-117 | positive |
| BRAF | AC068620.2 | 0.581602922 | 1.01E-101 | positive |
| ATRX | AC068620.2 | 0.441324278 | 2.96E-54 | positive |
| OTULIN | TTC3-AS1 | 0.470759874 | 1.83E-62 | positive |
| CYLD | TTC3-AS1 | 0.410306655 | 1.95E-46 | positive |
| DIABLO | TTC3-AS1 | 0.750258666 | 7.12E-202 | positive |
| BRAF | TTC3-AS1 | 0.65746362 | 9.80E-139 | positive |
| ATRX | TTC3-AS1 | 0.408886492 | 4.26E-46 | positive |
| DIABLO | AL133255.1 | 0.536617668 | 5.19E-84 | positive |
| BRAF | AL133255.1 | 0.509551747 | 1.40E-74 | positive |
| ATRX | AL133255.1 | 0.449507798 | 1.86E-56 | positive |
| TSC1 | LINC01409 | 0.452809731 | 2.31E-57 | positive |
| MAPK8 | LINC01409 | 0.479127628 | 6.02E-65 | positive |
| IPMK | LINC01409 | 0.455986143 | 3.05E-58 | positive |
| OTULIN | LINC01409 | 0.434257581 | 2.12E-52 | positive |
| CYLD | LINC01409 | 0.434189997 | 2.20E-52 | positive |
| MAP3K7 | LINC01409 | 0.411417088 | 1.06E-46 | positive |
| DIABLO | LINC01409 | 0.730048875 | 6.85E-186 | positive |
| CFLAR | LINC01409 | 0.505601782 | 2.83E-73 | positive |
| BRAF | LINC01409 | 0.701344532 | 1.57E-165 | positive |
| ATRX | LINC01409 | 0.591795483 | 3.95E-106 | positive |
| RNF31 | LINC01409 | 0.424494088 | 6.54E-50 | positive |
| MAPK8 | ENTPD1-AS1 | 0.433799812 | 2.78E-52 | positive |
| IPMK | ENTPD1-AS1 | 0.403374633 | 8.48E-45 | positive |
| OTULIN | ENTPD1-AS1 | 0.438489471 | 1.66E-53 | positive |
| CYLD | ENTPD1-AS1 | 0.503627186 | 1.25E-72 | positive |
| DIABLO | ENTPD1-AS1 | 0.795056772 | 1.70E-243 | positive |
| CFLAR | ENTPD1-AS1 | 0.487948682 | 1.22E-67 | positive |
| BRAF | ENTPD1-AS1 | 0.744363012 | 4.69E-197 | positive |
| ATRX | ENTPD1-AS1 | 0.528497274 | 4.31E-81 | positive |
| RNF31 | AL354836.1 | 0.453866034 | 1.18E-57 | positive |
| GATA3 | TSBP1-AS1 | 0.514231111 | 3.76E-76 | positive |
| CYLD | AC100827.4 | 0.403730719 | 7.00E-45 | positive |
| DIABLO | AC100827.4 | 0.670759985 | 2.29E-146 | positive |
| CFLAR | AC100827.4 | 0.448404262 | 3.72E-56 | positive |
| BRAF | AC100827.4 | 0.607567959 | 2.87E-113 | positive |
| ATRX | AC100827.4 | 0.543136506 | 2.06E-86 | positive |
| DIABLO | AC106052.1 | 0.480277987 | 2.71E-65 | positive |
| BRAF | AC106052.1 | 0.491251194 | 1.14E-68 | positive |
| MAPK8 | FOXP1-IT1 | 0.405434112 | 2.79E-45 | positive |
| OTULIN | FOXP1-IT1 | 0.468594343 | 7.85E-62 | positive |
| CYLD | FOXP1-IT1 | 0.430241051 | 2.29E-51 | positive |
| DIABLO | FOXP1-IT1 | 0.791756023 | 4.40E-240 | positive |
| BRAF | FOXP1-IT1 | 0.695518777 | 1.08E-161 | positive |
| ATRX | FOXP1-IT1 | 0.408302895 | 5.86E-46 | positive |
| ATRX | AC005332.1 | 0.433327945 | 3.68E-52 | positive |
| OTULIN | AL161725.1 | 0.424543692 | 6.35E-50 | positive |
| CYLD | AL161725.1 | 0.513204367 | 8.35E-76 | positive |
| DIABLO | AL161725.1 | 0.694156118 | 8.24E-161 | positive |
| CFLAR | AL161725.1 | 0.462649399 | 4.04E-60 | positive |
| BRAF | AL161725.1 | 0.643199173 | 5.74E-131 | positive |
| TSC1 | AC004918.3 | 0.502920601 | 2.13E-72 | positive |
| MAPK8 | AC004918.3 | 0.495742251 | 4.35E-70 | positive |
| IPMK | AC004918.3 | 0.460004659 | 2.27E-59 | positive |
| OTULIN | AC004918.3 | 0.5165268 | 6.25E-77 | positive |
| CYLD | AC004918.3 | 0.533106221 | 9.71E-83 | positive |
| MAP3K7 | AC004918.3 | 0.47226725 | 6.62E-63 | positive |
| DIABLO | AC004918.3 | 0.669191854 | 1.90E-145 | positive |
| CFLAR | AC004918.3 | 0.534072346 | 4.35E-83 | positive |
| BRAF | AC004918.3 | 0.801179865 | 5.39E-250 | positive |
| ATRX | AC004918.3 | 0.551510895 | 1.41E-89 | positive |
| MAPK8 | AC092821.3 | 0.422416977 | 2.16E-49 | positive |
| OTULIN | AC092821.3 | 0.542134184 | 4.85E-86 | positive |
| CYLD | AC092821.3 | 0.492484084 | 4.67E-69 | positive |
| MAP3K7 | AC092821.3 | 0.436775352 | 4.68E-53 | positive |
| DIABLO | AC092821.3 | 0.708185328 | 3.70E-170 | positive |
| CFLAR | AC092821.3 | 0.406519299 | 1.55E-45 | positive |
| BRAF | AC092821.3 | 0.639954026 | 2.94E-129 | positive |
| ATRX | AC092821.3 | 0.489525018 | 3.94E-68 | positive |
| RNF31 | AC092821.3 | 0.403580899 | 7.59E-45 | positive |
| BCL2L11 | AC092821.3 | 0.424795501 | 5.49E-50 | positive |
| OTULIN | AC112512.1 | 0.412043296 | 7.48E-47 | positive |
| CYLD | AC112512.1 | 0.424634176 | 6.03E-50 | positive |
| DIABLO | AC112512.1 | 0.80286333 | 8.03E-252 | positive |
| CFLAR | AC112512.1 | 0.429154198 | 4.34E-51 | positive |
| BRAF | AC112512.1 | 0.704157264 | 2.04E-167 | positive |
| ATRX | AC112512.1 | 0.540371882 | 2.18E-85 | positive |
| OTULIN | AL354794.1 | 0.402489753 | 1.36E-44 | positive |
| DIABLO | AL354794.1 | 0.748788584 | 1.17E-200 | positive |
| BRAF | AL354794.1 | 0.606364155 | 1.04E-112 | positive |
| DIABLO | AC007278.1 | 0.438315932 | 1.85E-53 | positive |
| RNF31 | OBSCN-AS1 | 0.445726198 | 1.97E-55 | positive |
| DIABLO | AC109361.2 | 0.606470876 | 9.28E-113 | positive |
| BRAF | AC109361.2 | 0.594892765 | 1.68E-107 | positive |
| MAPK8 | AC087521.2 | 0.415810515 | 9.17E-48 | positive |
| OTULIN | AC087521.2 | 0.497396594 | 1.29E-70 | positive |
| CYLD | AC087521.2 | 0.421803926 | 3.07E-49 | positive |
| DIABLO | AC087521.2 | 0.807867348 | 2.33E-257 | positive |
| BRAF | AC087521.2 | 0.695443596 | 1.20E-161 | positive |
| ATRX | AC087521.2 | 0.402008752 | 1.77E-44 | positive |
| DIABLO | AL161663.1 | 0.578822349 | 1.51E-100 | positive |
| BRAF | AL161663.1 | 0.487338294 | 1.88E-67 | positive |
| PANX1 | LUARIS | 0.447653859 | 5.94E-56 | positive |
| DIABLO | WWC2-AS1 | 0.621904995 | 4.08E-120 | positive |
| BRAF | WWC2-AS1 | 0.56341904 | 3.11E-94 | positive |
| TSC1 | MCM3AP-AS1 | 0.536512382 | 5.67E-84 | positive |
| MAPK8 | MCM3AP-AS1 | 0.470033504 | 2.99E-62 | positive |
| IPMK | MCM3AP-AS1 | 0.456533328 | 2.14E-58 | positive |
| OTULIN | MCM3AP-AS1 | 0.539109313 | 6.36E-85 | positive |
| CYLD | MCM3AP-AS1 | 0.400805143 | 3.36E-44 | positive |
| MAP3K7 | MCM3AP-AS1 | 0.500856993 | 9.97E-72 | positive |
| DIABLO | MCM3AP-AS1 | 0.688699656 | 2.55E-157 | positive |
| BRAF | MCM3AP-AS1 | 0.678242726 | 7.69E-151 | positive |
| ATRX | MCM3AP-AS1 | 0.447574099 | 6.25E-56 | positive |
| MAPK8 | AL133227.1 | 0.427994516 | 8.56E-51 | positive |
| OTULIN | AL133227.1 | 0.489376402 | 4.39E-68 | positive |
| CYLD | AL133227.1 | 0.456967483 | 1.62E-58 | positive |
| MAP3K7 | AL133227.1 | 0.424406416 | 6.88E-50 | positive |
| DIABLO | AL133227.1 | 0.807135608 | 1.54E-256 | positive |
| CFLAR | AL133227.1 | 0.430635081 | 1.82E-51 | positive |
| BRAF | AL133227.1 | 0.732580196 | 8.20E-188 | positive |
| ATRX | AL133227.1 | 0.469636595 | 3.90E-62 | positive |
| HDAC9 | MIR4527HG | 0.537559218 | 2.35E-84 | positive |
| DIABLO | ZMIZ1-AS1 | 0.441541855 | 2.60E-54 | positive |
| OTULIN | AL627308.3 | 0.447663219 | 5.91E-56 | positive |
| CYLD | AL627308.3 | 0.426255287 | 2.36E-50 | positive |
| DIABLO | AL627308.3 | 0.802852369 | 8.26E-252 | positive |
| BRAF | AL627308.3 | 0.692456568 | 1.03E-159 | positive |
| ATRX | AL627308.3 | 0.432678403 | 5.42E-52 | positive |
| BCL2 | AC104971.3 | 0.451758761 | 4.51E-57 | positive |
| BCL2L11 | AC104971.3 | 0.419333957 | 1.26E-48 | positive |
| DIABLO | AF228727.1 | 0.50376452 | 1.13E-72 | positive |
| BRAF | AF228727.1 | 0.48236448 | 6.31E-66 | positive |
| DIABLO | AC016999.1 | 0.616113373 | 2.63E-117 | positive |
| CFLAR | AC016999.1 | 0.416754865 | 5.39E-48 | positive |
| BRAF | AC016999.1 | 0.503062697 | 1.92E-72 | positive |
| MAP3K7 | AC107027.3 | 0.401690728 | 2.09E-44 | positive |
| BRAF | AC107027.3 | 0.486446803 | 3.55E-67 | positive |
| DIABLO | AC022210.1 | 0.460604255 | 1.54E-59 | positive |
| DIABLO | TEX41 | 0.458255765 | 7.06E-59 | positive |
| BRAF | TEX41 | 0.422593084 | 1.95E-49 | positive |
| OTULIN | AC009318.1 | 0.412148235 | 7.06E-47 | positive |
| DIABLO | AC009318.1 | 0.686732788 | 4.43E-156 | positive |
| BRAF | AC009318.1 | 0.616555353 | 1.61E-117 | positive |
| CD40 | AL117335.1 | 0.423595063 | 1.10E-49 | positive |
| CYLD | AC090825.1 | 0.484970392 | 1.01E-66 | positive |
| CFLAR | AC090825.1 | 0.458457096 | 6.20E-59 | positive |
| BRAF | AC090825.1 | 0.453627287 | 1.38E-57 | positive |
| ATRX | AC090825.1 | 0.44859562 | 3.30E-56 | positive |
| KLF9 | AC090825.1 | 0.515226235 | 1.73E-76 | positive |
| TSC1 | AC073611.1 | 0.458164103 | 7.49E-59 | positive |
| DIABLO | AC073611.1 | 0.449993628 | 1.37E-56 | positive |
| RNF31 | AC073611.1 | 0.573031185 | 3.91E-98 | positive |
| KLF9 | MBNL1-AS1 | 0.538712398 | 8.89E-85 | positive |
| CYLD | LINC01303 | 0.435481266 | 1.02E-52 | positive |
| DIABLO | LINC01303 | 0.546436077 | 1.19E-87 | positive |
| BRAF | LINC01303 | 0.465549159 | 5.96E-61 | positive |
| DIABLO | LINC02863 | 0.633030685 | 1.11E-125 | positive |
| BRAF | LINC02863 | 0.494276609 | 1.27E-69 | positive |
| RNF31 | AC116914.2 | 0.400332861 | 4.32E-44 | positive |
| EGFR | AC022509.2 | 0.41954037 | 1.12E-48 | positive |
| DIABLO | AC109449.1 | 0.436379869 | 5.94E-53 | positive |
| BRAF | AC079160.1 | 0.434083968 | 2.35E-52 | positive |
| DIABLO | AC104791.1 | 0.667246931 | 2.59E-144 | positive |
| BRAF | AC104791.1 | 0.560508618 | 4.44E-93 | positive |
| OTULIN | AC009137.2 | 0.401690005 | 2.09E-44 | positive |
| DIABLO | AC009137.2 | 0.687269803 | 2.04E-156 | positive |
| BRAF | AC009137.2 | 0.608013606 | 1.78E-113 | positive |
| ATRX | AC009137.2 | 0.430806703 | 1.64E-51 | positive |
| BACH2 | ATP2B1-AS1 | 0.47000417 | 3.05E-62 | positive |
| DIABLO | TMC3-AS1 | 0.608833641 | 7.37E-114 | positive |
| BRAF | TMC3-AS1 | 0.530603438 | 7.67E-82 | positive |
| TSC1 | LINC00910 | 0.400372881 | 4.23E-44 | positive |
| OTULIN | AC005632.6 | 0.409829267 | 2.54E-46 | positive |
| DIABLO | AC005632.6 | 0.722037989 | 5.97E-180 | positive |
| CFLAR | AC005632.6 | 0.45920389 | 3.82E-59 | positive |
| BRAF | AC005632.6 | 0.608593 | 9.55E-114 | positive |
| ATRX | AC005632.6 | 0.478398791 | 9.97E-65 | positive |
| OTULIN | AL445309.1 | 0.416232556 | 7.23E-48 | positive |
| CYLD | AL445309.1 | 0.423429543 | 1.21E-49 | positive |
| DIABLO | AL445309.1 | 0.734681544 | 2.00E-189 | positive |
| CFLAR | AL445309.1 | 0.420005702 | 8.57E-49 | positive |
| BRAF | AL445309.1 | 0.608998924 | 6.17E-114 | positive |
| ATRX | AL445309.1 | 0.410649077 | 1.62E-46 | positive |
| OTULIN | BCL2L1-AS1 | 0.41275301 | 5.05E-47 | positive |
| DIABLO | BCL2L1-AS1 | 0.79288446 | 3.05E-241 | positive |
| CFLAR | BCL2L1-AS1 | 0.403806287 | 6.72E-45 | positive |
| BRAF | BCL2L1-AS1 | 0.647733474 | 2.16E-133 | positive |
| ATRX | BCL2L1-AS1 | 0.402178702 | 1.61E-44 | positive |
| MAPK8 | AC130650.2 | 0.451212462 | 6.36E-57 | positive |
| IPMK | AC130650.2 | 0.428313511 | 7.10E-51 | positive |
| OTULIN | AC130650.2 | 0.509394664 | 1.58E-74 | positive |
| CYLD | AC130650.2 | 0.44486433 | 3.36E-55 | positive |
| MAP3K7 | AC130650.2 | 0.447556585 | 6.32E-56 | positive |
| DIABLO | AC130650.2 | 0.783418236 | 9.86E-232 | positive |
| BRAF | AC130650.2 | 0.68027668 | 4.44E-152 | positive |
| ATRX | AC130650.2 | 0.435918239 | 7.83E-53 | positive |
| MYC | SNHG17 | 0.404699596 | 4.15E-45 | positive |
| SPATA2 | SNHG17 | 0.489474221 | 4.09E-68 | positive |
| DIABLO | AC023480.1 | 0.751676449 | 4.72E-203 | positive |
| BRAF | AC023480.1 | 0.651303794 | 2.49E-135 | positive |
| TSC1 | AC022893.1 | 0.454352146 | 8.67E-58 | positive |
| MAPK8 | AC022893.1 | 0.420110414 | 8.07E-49 | positive |
| OTULIN | AC022893.1 | 0.401385503 | 2.46E-44 | positive |
| CYLD | AC022893.1 | 0.414707869 | 1.70E-47 | positive |
| DIABLO | AC022893.1 | 0.547249172 | 5.89E-88 | positive |
| CFLAR | AC022893.1 | 0.423176378 | 1.40E-49 | positive |
| BRAF | AC022893.1 | 0.607717088 | 2.45E-113 | positive |
| ATRX | AC022893.1 | 0.586454643 | 8.44E-104 | positive |
| KLF9 | AC022893.1 | 0.404944722 | 3.64E-45 | positive |
| OTULIN | AL360093.1 | 0.466388148 | 3.42E-61 | positive |
| DIABLO | AL360093.1 | 0.682224826 | 2.82E-153 | positive |
| BRAF | AL360093.1 | 0.63189423 | 4.23E-125 | positive |
| ATRX | AL360093.1 | 0.404198634 | 5.44E-45 | positive |
| MAPK8 | AL513327.1 | 0.431437869 | 1.13E-51 | positive |
| OTULIN | AL513327.1 | 0.491780984 | 7.77E-69 | positive |
| CYLD | AL513327.1 | 0.459298745 | 3.59E-59 | positive |
| MAP3K7 | AL513327.1 | 0.403351651 | 8.59E-45 | positive |
| DIABLO | AL513327.1 | 0.766909701 | 3.03E-216 | positive |
| CFLAR | AL513327.1 | 0.459774718 | 2.64E-59 | positive |
| BRAF | AL513327.1 | 0.691525881 | 4.06E-159 | positive |
| ATRX | AL513327.1 | 0.425014207 | 4.84E-50 | positive |
| DIABLO | KIF26B-AS1 | 0.583795549 | 1.17E-102 | positive |
| BRAF | KIF26B-AS1 | 0.554412015 | 1.08E-90 | positive |
| ATRX | KIF26B-AS1 | 0.480693076 | 2.03E-65 | positive |
| DIABLO | AGAP1-IT1 | 0.459487295 | 3.18E-59 | positive |
| BCL2L11 | UST-AS2 | 0.453111323 | 1.91E-57 | positive |
| DIABLO | AP003117.1 | 0.482115344 | 7.52E-66 | positive |
| BRAF | AP003117.1 | 0.467183336 | 2.01E-61 | positive |
| ATRX | AP003117.1 | 0.458876199 | 4.72E-59 | positive |
| DIABLO | AL590133.1 | 0.453164144 | 1.85E-57 | positive |
| BRAF | AL590133.1 | 0.401518212 | 2.29E-44 | positive |
| ATRX | AL590133.1 | 0.416928286 | 4.89E-48 | positive |
| RNF31 | AL590133.1 | 0.46571246 | 5.35E-61 | positive |
| DIABLO | AC087588.1 | 0.4479481 | 4.95E-56 | positive |
| OTULIN | RPS6KA2-IT1 | 0.405945091 | 2.12E-45 | positive |
| DIABLO | RPS6KA2-IT1 | 0.587048409 | 4.67E-104 | positive |
| CFLAR | RPS6KA2-IT1 | 0.419534008 | 1.12E-48 | positive |
| BRAF | RPS6KA2-IT1 | 0.56182661 | 1.34E-93 | positive |
| TSC1 | AC138956.2 | 0.593854332 | 4.87E-107 | positive |
| OTULIN | AC138956.2 | 0.404004633 | 6.04E-45 | positive |
| CYLD | AC138956.2 | 0.429455579 | 3.63E-51 | positive |
| DIABLO | AC138956.2 | 0.580534941 | 2.87E-101 | positive |
| CFLAR | AC138956.2 | 0.476301777 | 4.23E-64 | positive |
| BRAF | AC138956.2 | 0.568754528 | 2.21E-96 | positive |
| ATRX | AC138956.2 | 0.467297904 | 1.87E-61 | positive |
| RNF31 | AC138956.2 | 0.512567508 | 1.37E-75 | positive |
| OTULIN | AC108063.1 | 0.467253913 | 1.92E-61 | positive |
| CYLD | AC108063.1 | 0.405945278 | 2.12E-45 | positive |
| DIABLO | AC108063.1 | 0.722179001 | 4.71E-180 | positive |
| BRAF | AC108063.1 | 0.645696473 | 2.68E-132 | positive |
| MAPK8 | AC079684.1 | 0.421322578 | 4.04E-49 | positive |
| OTULIN | AC079684.1 | 0.491348267 | 1.06E-68 | positive |
| MAP3K7 | AC079684.1 | 0.403440221 | 8.19E-45 | positive |
| DIABLO | AC079684.1 | 0.689494305 | 8.01E-158 | positive |
| BRAF | AC079684.1 | 0.584178799 | 8.05E-103 | positive |
| TSC1 | AC018653.3 | 0.466826003 | 2.56E-61 | positive |
| CYLD | AC018653.3 | 0.418090878 | 2.54E-48 | positive |
| RNF31 | AC018653.3 | 0.408844471 | 4.36E-46 | positive |
| DIABLO | AC091769.1 | 0.675773067 | 2.38E-149 | positive |
| CFLAR | AC091769.1 | 0.471180767 | 1.38E-62 | positive |
| BRAF | AC091769.1 | 0.587861426 | 2.07E-104 | positive |
| ATRX | AC091769.1 | 0.532984446 | 1.07E-82 | positive |
| DIABLO | AC100843.1 | 0.489440407 | 4.19E-68 | positive |
| DIABLO | SH3TC2-DT | 0.559659191 | 9.62E-93 | positive |
| BRAF | SH3TC2-DT | 0.484518568 | 1.39E-66 | positive |
| DIABLO | LINC01293 | 0.491865279 | 7.31E-69 | positive |
| BRAF | LINC01293 | 0.482174687 | 7.21E-66 | positive |
| DIABLO | BMS1P4 | 0.497431938 | 1.26E-70 | positive |
| CFLAR | BMS1P4 | 0.455618936 | 3.85E-58 | positive |
| BRAF | BMS1P4 | 0.430403645 | 2.08E-51 | positive |
| ATRX | BMS1P4 | 0.459907319 | 2.42E-59 | positive |
| OTULIN | AC015987.1 | 0.413497528 | 3.34E-47 | positive |
| DIABLO | AC015987.1 | 0.713951128 | 3.66E-174 | positive |
| BRAF | AC015987.1 | 0.62355722 | 6.30E-121 | positive |
| DIABLO | AC020916.2 | 0.583331133 | 1.86E-102 | positive |
| CFLAR | AC020916.2 | 0.451112109 | 6.78E-57 | positive |
| BRAF | AC020916.2 | 0.526586692 | 2.04E-80 | positive |
| ATRX | AC020916.2 | 0.466863871 | 2.49E-61 | positive |
| OTULIN | AC000065.2 | 0.442300622 | 1.63E-54 | positive |
| DIABLO | AC000065.2 | 0.700907036 | 3.06E-165 | positive |
| BRAF | AC000065.2 | 0.608926955 | 6.67E-114 | positive |
| CD40 | LINC02416 | 0.524326401 | 1.27E-79 | positive |
| DIABLO | AC097468.3 | 0.493312529 | 2.56E-69 | positive |
| BRAF | AC097468.3 | 0.468746732 | 7.09E-62 | positive |
| RNF31 | AC109460.3 | 0.459192771 | 3.85E-59 | positive |
| OTULIN | ATP1A1-AS1 | 0.441636503 | 2.45E-54 | positive |
| DIABLO | ATP1A1-AS1 | 0.688677433 | 2.64E-157 | positive |
| BRAF | ATP1A1-AS1 | 0.618298843 | 2.33E-118 | positive |
| KLF9 | AC053503.3 | 0.535209696 | 1.69E-83 | positive |
| OTULIN | AC127035.1 | 0.40607787 | 1.97E-45 | positive |
| CYLD | AC127035.1 | 0.410600385 | 1.66E-46 | positive |
| DIABLO | AC127035.1 | 0.822481297 | 1.65E-274 | positive |
| BRAF | AC127035.1 | 0.664409722 | 1.13E-142 | positive |
| ATRX | AC127035.1 | 0.426384977 | 2.19E-50 | positive |
| DIABLO | STXBP5-AS1 | 0.413337557 | 3.65E-47 | positive |
| DIABLO | AC100778.1 | 0.682555669 | 1.77E-153 | positive |
| BRAF | AC100778.1 | 0.526764135 | 1.77E-80 | positive |
| CD40 | FAM30A | 0.574813791 | 7.17E-99 | positive |
| OTULIN | AC005480.1 | 0.434415722 | 1.93E-52 | positive |
| CYLD | AC005480.1 | 0.47145746 | 1.15E-62 | positive |
| DIABLO | AC005480.1 | 0.819227762 | 1.48E-270 | positive |
| CFLAR | AC005480.1 | 0.433037683 | 4.38E-52 | positive |
| BRAF | AC005480.1 | 0.692901068 | 5.32E-160 | positive |
| ATRX | AC005480.1 | 0.499863248 | 2.09E-71 | positive |
| DIABLO | AP002993.1 | 0.618301764 | 2.32E-118 | positive |
| CFLAR | AP002993.1 | 0.429679108 | 3.19E-51 | positive |
| BRAF | AP002993.1 | 0.534570626 | 2.87E-83 | positive |
| ATRX | AP002993.1 | 0.544088202 | 9.08E-87 | positive |
| TSC1 | RAD51-AS1 | 0.509510073 | 1.44E-74 | positive |
| RNF31 | RAD51-AS1 | 0.402141435 | 1.64E-44 | positive |
| CYLD | AC005730.3 | 0.42983858 | 2.90E-51 | positive |
| DIABLO | AC005730.3 | 0.707836192 | 6.43E-170 | positive |
| CFLAR | AC005730.3 | 0.480402802 | 2.48E-65 | positive |
| BRAF | AC005730.3 | 0.65277432 | 3.90E-136 | positive |
| ATRX | AC005730.3 | 0.599335005 | 1.71E-109 | positive |
| OTULIN | AL157932.1 | 0.440429188 | 5.12E-54 | positive |
| MAP3K7 | AL157932.1 | 0.464408641 | 1.27E-60 | positive |
| DIABLO | AL157932.1 | 0.57335751 | 2.87E-98 | positive |
| BRAF | AL157932.1 | 0.572418016 | 7.00E-98 | positive |
| ATRX | AL157932.1 | 0.442120107 | 1.82E-54 | positive |
| DIABLO | AC092718.1 | 0.710677853 | 7.07E-172 | positive |
| BRAF | AC092718.1 | 0.616232937 | 2.30E-117 | positive |
| ATRX | AC092718.1 | 0.405663445 | 2.47E-45 | positive |
| DIABLO | NPAS2-AS1 | 0.627740686 | 5.26E-123 | positive |
| CFLAR | NPAS2-AS1 | 0.433399043 | 3.53E-52 | positive |
| BRAF | NPAS2-AS1 | 0.515712947 | 1.18E-76 | positive |
| ATRX | NPAS2-AS1 | 0.447402749 | 6.95E-56 | positive |
| RNF31 | NPAS2-AS1 | 0.412842219 | 4.80E-47 | positive |
| DIABLO | BX842570.1 | 0.448741486 | 3.01E-56 | positive |
| DIABLO | AL355001.1 | 0.516572832 | 6.03E-77 | positive |
| BRAF | AL355001.1 | 0.407088786 | 1.14E-45 | positive |
| ATRX | AL355001.1 | 0.443337371 | 8.63E-55 | positive |
| DIABLO | PKP4-AS1 | 0.472784336 | 4.66E-63 | positive |
| BRAF | PKP4-AS1 | 0.458944683 | 4.52E-59 | positive |
| GATA3 | AC010735.1 | 0.428359988 | 6.91E-51 | positive |
| AXL | AC018978.1 | 0.410252426 | 2.01E-46 | positive |
| OTULIN | AC080013.2 | 0.454577961 | 7.50E-58 | positive |
| CYLD | AC080013.2 | 0.436109418 | 6.98E-53 | positive |
| DIABLO | AC080013.2 | 0.604463043 | 7.85E-112 | positive |
| BRAF | AC080013.2 | 0.5865718 | 7.51E-104 | positive |
| ATRX | AC080013.2 | 0.460715056 | 1.43E-59 | positive |
| DIABLO | AC008870.4 | 0.61331705 | 5.68E-116 | positive |
| BRAF | AC008870.4 | 0.496117909 | 3.31E-70 | positive |
| DIABLO | AC007688.3 | 0.700837065 | 3.41E-165 | positive |
| BRAF | AC007688.3 | 0.556585661 | 1.54E-91 | positive |
| DIABLO | AL353600.1 | 0.484428384 | 1.48E-66 | positive |
| BRAF | AL353600.1 | 0.47732741 | 2.09E-64 | positive |
| TSC1 | AC138035.1 | 0.445731349 | 1.97E-55 | positive |
| ATRX | AC138035.1 | 0.510174845 | 8.66E-75 | positive |
| CYLD | AL445985.1 | 0.409533178 | 2.99E-46 | positive |
| DIABLO | AL445985.1 | 0.608938773 | 6.58E-114 | positive |
| BRAF | AL445985.1 | 0.544561723 | 6.04E-87 | positive |
| BACH2 | AL139020.1 | 0.538542805 | 1.03E-84 | positive |
| TSC1 | AC005519.1 | 0.594611321 | 2.25E-107 | positive |
| OTULIN | AC005519.1 | 0.48031509 | 2.64E-65 | positive |
| CYLD | AC005519.1 | 0.435196081 | 1.21E-52 | positive |
| MAP3K7 | AC005519.1 | 0.422923854 | 1.62E-49 | positive |
| DIABLO | AC005519.1 | 0.586891217 | 5.46E-104 | positive |
| CFLAR | AC005519.1 | 0.474698316 | 1.27E-63 | positive |
| BRAF | AC005519.1 | 0.602197188 | 8.59E-111 | positive |
| ATRX | AC005519.1 | 0.568816041 | 2.08E-96 | positive |
| RNF31 | AC005519.1 | 0.612304454 | 1.72E-115 | positive |
| CYLD | CHRM3-AS2 | 0.422835447 | 1.70E-49 | positive |
| DIABLO | CHRM3-AS2 | 0.627966656 | 4.05E-123 | positive |
| CFLAR | CHRM3-AS2 | 0.415283074 | 1.23E-47 | positive |
| BRAF | CHRM3-AS2 | 0.583187072 | 2.14E-102 | positive |
| ATRX | CHRM3-AS2 | 0.429826648 | 2.92E-51 | positive |
| DIABLO | SNHG22 | 0.71348624 | 7.77E-174 | positive |
| BRAF | SNHG22 | 0.54829111 | 2.38E-88 | positive |
| OTULIN | AC007362.1 | 0.440196231 | 5.90E-54 | positive |
| CYLD | AC007362.1 | 0.459788638 | 2.61E-59 | positive |
| DIABLO | AC007362.1 | 0.709182391 | 7.64E-171 | positive |
| CFLAR | AC007362.1 | 0.402755361 | 1.18E-44 | positive |
| BRAF | AC007362.1 | 0.668934519 | 2.69E-145 | positive |
| DIABLO | FSIP2-AS1 | 0.547639399 | 4.20E-88 | positive |
| BRAF | FSIP2-AS1 | 0.490924973 | 1.44E-68 | positive |
| OTULIN | AC016746.1 | 0.41473765 | 1.67E-47 | positive |
| DIABLO | AC016746.1 | 0.64825621 | 1.13E-133 | positive |
| BRAF | AC016746.1 | 0.577294188 | 6.63E-100 | positive |
| DIABLO | LINC00535 | 0.420742456 | 5.63E-49 | positive |
| DIABLO | AC078883.2 | 0.588946349 | 7.00E-105 | positive |
| BRAF | AC078883.2 | 0.442487098 | 1.45E-54 | positive |
| DIABLO | AC009248.3 | 0.555982789 | 2.64E-91 | positive |
| BRAF | AC009248.3 | 0.425121989 | 4.55E-50 | positive |
| DIABLO | AC024681.2 | 0.444138817 | 5.27E-55 | positive |
| DIABLO | AC068189.1 | 0.466742184 | 2.70E-61 | positive |
| CYLD | AC114980.1 | 0.468550036 | 8.09E-62 | positive |
| DIABLO | AC114980.1 | 0.739073144 | 7.63E-193 | positive |
| CFLAR | AC114980.1 | 0.46127749 | 9.91E-60 | positive |
| BRAF | AC114980.1 | 0.685419415 | 2.94E-155 | positive |
| ATRX | AC114980.1 | 0.52048129 | 2.75E-78 | positive |
| DIABLO | AL596087.3 | 0.693026406 | 4.42E-160 | positive |
| BRAF | AL596087.3 | 0.603115378 | 3.26E-111 | positive |
| OTULIN | MACC1-AS1 | 0.42963972 | 3.26E-51 | positive |
| DIABLO | MACC1-AS1 | 0.734184333 | 4.84E-189 | positive |
| BRAF | MACC1-AS1 | 0.629643326 | 5.82E-124 | positive |
| DIABLO | AL023803.3 | 0.425376699 | 3.92E-50 | positive |
| CYLD | AC110611.2 | 0.439339167 | 9.93E-54 | positive |
| CFLAR | AC110611.2 | 0.44706048 | 8.61E-56 | positive |
| KLF9 | AC110611.2 | 0.439788169 | 7.56E-54 | positive |
| DIABLO | AP006545.1 | 0.479053912 | 6.34E-65 | positive |
| BRAF | AP006545.1 | 0.452807788 | 2.32E-57 | positive |
| DIABLO | AP002812.2 | 0.445368101 | 2.46E-55 | positive |
| ATRX | AP002812.2 | 0.500608274 | 1.20E-71 | positive |
| RNF31 | AP002812.2 | 0.541964816 | 5.61E-86 | positive |
| GATA3 | EDIL3-DT | 0.475040833 | 1.00E-63 | positive |
| DIABLO | AL512652.1 | 0.623664644 | 5.57E-121 | positive |
| BRAF | AL512652.1 | 0.490287468 | 2.28E-68 | positive |
| PANX1 | C10orf55 | 0.439427932 | 9.41E-54 | positive |
| OTULIN | AC022400.2 | 0.420094031 | 8.15E-49 | positive |
| CYLD | AC022400.2 | 0.405520387 | 2.66E-45 | positive |
| DIABLO | AC022400.2 | 0.767328597 | 1.27E-216 | positive |
| CFLAR | AC022400.2 | 0.463979509 | 1.68E-60 | positive |
| BRAF | AC022400.2 | 0.664001348 | 1.94E-142 | positive |
| ATRX | AC022400.2 | 0.50779268 | 5.36E-74 | positive |
| CYLD | AC073655.3 | 0.456795909 | 1.81E-58 | positive |
| DIABLO | AC073655.3 | 0.659402719 | 7.99E-140 | positive |
| CFLAR | AC073655.3 | 0.428677987 | 5.74E-51 | positive |
| BRAF | AC073655.3 | 0.59757734 | 1.06E-108 | positive |
| ATRX | AC073655.3 | 0.415371839 | 1.17E-47 | positive |
| MAP3K7 | AC004837.4 | 0.401069367 | 2.92E-44 | positive |
| DIABLO | AC004837.4 | 0.630083422 | 3.49E-124 | positive |
| CFLAR | AC004837.4 | 0.40040799 | 4.15E-44 | positive |
| BRAF | AC004837.4 | 0.580532033 | 2.88E-101 | positive |
| ATRX | AC004837.4 | 0.423013193 | 1.53E-49 | positive |
| MAPK8 | AC097448.1 | 0.441304446 | 3.00E-54 | positive |
| OTULIN | AC097448.1 | 0.43665326 | 5.04E-53 | positive |
| CYLD | AC097448.1 | 0.427661643 | 1.04E-50 | positive |
| MAP3K7 | AC097448.1 | 0.514845057 | 2.33E-76 | positive |
| DIABLO | AC097448.1 | 0.436148794 | 6.82E-53 | positive |
| BRAF | AC097448.1 | 0.496071466 | 3.42E-70 | positive |
| CD40 | MIR3681HG | 0.533855576 | 5.21E-83 | positive |
| OTULIN | AC084117.1 | 0.426002482 | 2.73E-50 | positive |
| DIABLO | AC084117.1 | 0.620942592 | 1.21E-119 | positive |
| BRAF | AC084117.1 | 0.508205424 | 3.91E-74 | positive |
| FASLG | AC022126.1 | 0.450661445 | 9.01E-57 | positive |
| DIABLO | LINC02163 | 0.44337974 | 8.41E-55 | positive |
| BRAF | LINC02163 | 0.430043031 | 2.57E-51 | positive |
| GATA3 | AC087477.2 | 0.522350417 | 6.20E-79 | positive |
| KLF9 | AC087477.2 | 0.435642442 | 9.24E-53 | positive |
| OTULIN | AC006160.1 | 0.421612497 | 3.43E-49 | positive |
| CYLD | AC006160.1 | 0.482236958 | 6.90E-66 | positive |
| DIABLO | AC006160.1 | 0.752491099 | 9.83E-204 | positive |
| CFLAR | AC006160.1 | 0.469169489 | 5.34E-62 | positive |
| BRAF | AC006160.1 | 0.621876 | 4.22E-120 | positive |
| ATRX | AC006160.1 | 0.411716171 | 8.97E-47 | positive |
| DIABLO | ADD3-AS1 | 0.401127561 | 2.83E-44 | positive |
| DIABLO | LINC01050 | 0.489368902 | 4.41E-68 | positive |
| BRAF | LINC01050 | 0.447018678 | 8.83E-56 | positive |
| OTULIN | AC016866.2 | 0.459341317 | 3.49E-59 | positive |
| CYLD | AC016866.2 | 0.406296269 | 1.75E-45 | positive |
| DIABLO | AC016866.2 | 0.764373896 | 5.58E-214 | positive |
| BRAF | AC016866.2 | 0.657985245 | 5.00E-139 | positive |
| DIABLO | C15orf54 | 0.469696809 | 3.75E-62 | positive |
| BRAF | C15orf54 | 0.46829227 | 9.61E-62 | positive |
| RNF31 | AC116552.1 | 0.400073882 | 4.95E-44 | positive |
| RNF31 | PP7080 | 0.465497311 | 6.17E-61 | positive |
| DIABLO | AC005046.1 | 0.506440158 | 1.50E-73 | positive |
| BRAF | AC005046.1 | 0.475892265 | 5.60E-64 | positive |
| DIABLO | AL157392.2 | 0.723946627 | 2.40E-181 | positive |
| CFLAR | AL157392.2 | 0.409919568 | 2.42E-46 | positive |
| BRAF | AL157392.2 | 0.622064981 | 3.41E-120 | positive |
| ATRX | AL157392.2 | 0.40473367 | 4.08E-45 | positive |
| MAPK8 | AC026124.2 | 0.410573829 | 1.69E-46 | positive |
| OTULIN | AC026124.2 | 0.446237898 | 1.44E-55 | positive |
| DIABLO | AC026124.2 | 0.735745966 | 3.02E-190 | positive |
| BRAF | AC026124.2 | 0.620620019 | 1.73E-119 | positive |
| SIRT3 | TMEM147-AS1 | 0.426426552 | 2.13E-50 | positive |
| MYC | TMEM147-AS1 | 0.42061544 | 6.06E-49 | positive |
| DIABLO | SPAG5-AS1 | 0.526572661 | 2.06E-80 | positive |
| BRAF | SPAG5-AS1 | 0.445740417 | 1.96E-55 | positive |
| RNF31 | SPAG5-AS1 | 0.406993158 | 1.20E-45 | positive |
| DIABLO | AC025449.2 | 0.519952847 | 4.19E-78 | positive |
| BRAF | AC025449.2 | 0.437900843 | 2.37E-53 | positive |
| OTULIN | AC093788.1 | 0.460140991 | 2.08E-59 | positive |
| CYLD | AC093788.1 | 0.413273787 | 3.78E-47 | positive |
| MAP3K7 | AC093788.1 | 0.402286277 | 1.52E-44 | positive |
| DIABLO | AC093788.1 | 0.689935211 | 4.20E-158 | positive |
| BRAF | AC093788.1 | 0.651160397 | 2.99E-135 | positive |
| ATRX | AC093788.1 | 0.462645345 | 4.05E-60 | positive |
| LEF1 | AC116049.1 | 0.429692132 | 3.16E-51 | positive |
| DIABLO | AC100839.1 | 0.56393904 | 1.93E-94 | positive |
| BRAF | AC100839.1 | 0.464469421 | 1.22E-60 | positive |
| DIABLO | AC011330.2 | 0.447648431 | 5.97E-56 | positive |
| BRAF | AC011330.2 | 0.4024272 | 1.41E-44 | positive |
| OTULIN | NBAT1 | 0.411379806 | 1.08E-46 | positive |
| DIABLO | NBAT1 | 0.656831488 | 2.21E-138 | positive |
| BRAF | NBAT1 | 0.614597961 | 1.40E-116 | positive |
| MAP3K7 | AL354892.3 | 0.41605621 | 7.99E-48 | positive |
| DIABLO | AL354892.3 | 0.642305082 | 1.70E-130 | positive |
| CFLAR | AL354892.3 | 0.44189651 | 2.09E-54 | positive |
| BRAF | AL354892.3 | 0.563683328 | 2.44E-94 | positive |
| ATRX | AL354892.3 | 0.5348073 | 2.36E-83 | positive |
| RNF31 | AL354892.3 | 0.447354855 | 7.16E-56 | positive |
| CYLD | AC027682.1 | 0.421871882 | 2.95E-49 | positive |
| DIABLO | AC027682.1 | 0.499984563 | 1.91E-71 | positive |
| BRAF | AC027682.1 | 0.457516598 | 1.14E-58 | positive |
| ATRX | AC027682.1 | 0.408721444 | 4.66E-46 | positive |
| DIABLO | AC011442.1 | 0.634351755 | 2.35E-126 | positive |
| BRAF | AC011442.1 | 0.537382428 | 2.73E-84 | positive |
| ATRX | AC011442.1 | 0.427338337 | 1.25E-50 | positive |
| MAPK8 | AL031772.1 | 0.411289904 | 1.14E-46 | positive |
| OTULIN | AL031772.1 | 0.468932594 | 6.26E-62 | positive |
| CYLD | AL031772.1 | 0.471603553 | 1.04E-62 | positive |
| DIABLO | AL031772.1 | 0.816391082 | 3.55E-267 | positive |
| CFLAR | AL031772.1 | 0.407914396 | 7.25E-46 | positive |
| BRAF | AL031772.1 | 0.720617628 | 6.41E-179 | positive |
| ATRX | AL031772.1 | 0.42918192 | 4.27E-51 | positive |
| TSC1 | CAPN10-DT | 0.414468036 | 1.94E-47 | positive |
| SPATA2 | CAPN10-DT | 0.404382224 | 4.93E-45 | positive |
| TSC1 | AL049840.4 | 0.466876667 | 2.47E-61 | positive |
| IPMK | AL049840.4 | 0.415704696 | 9.73E-48 | positive |
| PANX1 | AL049840.4 | 0.404163028 | 5.55E-45 | positive |
| OTULIN | AL049840.4 | 0.472713718 | 4.89E-63 | positive |
| CYLD | AL049840.4 | 0.499142975 | 3.56E-71 | positive |
| MAP3K7 | AL049840.4 | 0.413915806 | 2.64E-47 | positive |
| CFLAR | AL049840.4 | 0.433121784 | 4.16E-52 | positive |
| BRAF | AL049840.4 | 0.506782585 | 1.16E-73 | positive |
| ATRX | AL049840.4 | 0.480751691 | 1.95E-65 | positive |
| OTULIN | AC009303.2 | 0.43480649 | 1.52E-52 | positive |
| CYLD | AC009303.2 | 0.413222002 | 3.89E-47 | positive |
| DIABLO | AC009303.2 | 0.803067021 | 4.81E-252 | positive |
| BRAF | AC009303.2 | 0.672387593 | 2.50E-147 | positive |
| DIABLO | AC010542.1 | 0.51576646 | 1.13E-76 | positive |
| CYLD | AL512306.3 | 0.420855688 | 5.28E-49 | positive |
| CFLAR | AL512306.3 | 0.483414944 | 3.02E-66 | positive |
| ATRX | AL512306.3 | 0.417243258 | 4.10E-48 | positive |
| ATRX | LINC00174 | 0.404921627 | 3.68E-45 | positive |
| RNF31 | LINC00174 | 0.459687852 | 2.79E-59 | positive |
| MAPK8 | AL158163.1 | 0.432441716 | 6.24E-52 | positive |
| IPMK | AL158163.1 | 0.400930418 | 3.14E-44 | positive |
| CYLD | AL158163.1 | 0.506705047 | 1.23E-73 | positive |
| DIABLO | AL158163.1 | 0.71033121 | 1.23E-171 | positive |
| CFLAR | AL158163.1 | 0.517949492 | 2.04E-77 | positive |
| BRAF | AL158163.1 | 0.686404231 | 7.13E-156 | positive |
| ATRX | AL158163.1 | 0.615651723 | 4.37E-117 | positive |
| OTULIN | AC024267.5 | 0.413973216 | 2.56E-47 | positive |
| CYLD | AC024267.5 | 0.443417051 | 8.22E-55 | positive |
| DIABLO | AC024267.5 | 0.774276674 | 5.41E-223 | positive |
| CFLAR | AC024267.5 | 0.472603823 | 5.27E-63 | positive |
| BRAF | AC024267.5 | 0.69041514 | 2.08E-158 | positive |
| ATRX | AC024267.5 | 0.52461086 | 1.01E-79 | positive |
| CYLD | AL035106.1 | 0.449095761 | 2.41E-56 | positive |
| DIABLO | AL035106.1 | 0.696806809 | 1.55E-162 | positive |
| CFLAR | AL035106.1 | 0.422489586 | 2.07E-49 | positive |
| BRAF | AL035106.1 | 0.630151245 | 3.23E-124 | positive |
| MAPK8 | IQCH-AS1 | 0.541449379 | 8.71E-86 | positive |
| IPMK | IQCH-AS1 | 0.462835652 | 3.57E-60 | positive |
| DIABLO | IQCH-AS1 | 0.493761713 | 1.85E-69 | positive |
| BRAF | IQCH-AS1 | 0.546562892 | 1.07E-87 | positive |
| SIRT1 | IQCH-AS1 | 0.418547301 | 1.96E-48 | positive |
| ATRX | IQCH-AS1 | 0.420706509 | 5.75E-49 | positive |
| OTULIN | AC016542.1 | 0.409345128 | 3.31E-46 | positive |
| DIABLO | AC016542.1 | 0.712730489 | 2.63E-173 | positive |
| BRAF | AC016542.1 | 0.61688844 | 1.11E-117 | positive |
| DIABLO | LINC02718 | 0.4462154 | 1.46E-55 | positive |
| BRAF | LINC02718 | 0.432994264 | 4.49E-52 | positive |
| ATRX | LINC02718 | 0.404129522 | 5.65E-45 | positive |
| MAPK8 | AC016597.1 | 0.412878511 | 4.71E-47 | positive |
| OTULIN | AC016597.1 | 0.467695008 | 1.43E-61 | positive |
| CYLD | AC016597.1 | 0.426927004 | 1.59E-50 | positive |
| MAP3K7 | AC016597.1 | 0.404932298 | 3.66E-45 | positive |
| DIABLO | AC016597.1 | 0.772481984 | 2.51E-221 | positive |
| CFLAR | AC016597.1 | 0.404722992 | 4.10E-45 | positive |
| BRAF | AC016597.1 | 0.681206709 | 1.19E-152 | positive |
| ATRX | AC016597.1 | 0.477080782 | 2.47E-64 | positive |
| CYLD | AC025188.1 | 0.428778477 | 5.41E-51 | positive |
| DIABLO | AC025188.1 | 0.705499622 | 2.52E-168 | positive |
| CFLAR | AC025188.1 | 0.425669525 | 3.31E-50 | positive |
| BRAF | AC025188.1 | 0.650148319 | 1.06E-134 | positive |
| ATRX | AC025188.1 | 0.445496232 | 2.27E-55 | positive |
| DIABLO | AC087277.2 | 0.590666348 | 1.24E-105 | positive |
| BRAF | AC087277.2 | 0.49220743 | 5.71E-69 | positive |
| OTULIN | Z98749.1 | 0.470083103 | 2.89E-62 | positive |
| CYLD | Z98749.1 | 0.442899289 | 1.13E-54 | positive |
| DIABLO | Z98749.1 | 0.742479506 | 1.52E-195 | positive |
| BRAF | Z98749.1 | 0.674096543 | 2.40E-148 | positive |
| ATRX | Z98749.1 | 0.406539471 | 1.53E-45 | positive |
| TSC1 | LINC01128 | 0.469530733 | 4.19E-62 | positive |
| MAPK8 | LINC01128 | 0.445647847 | 2.07E-55 | positive |
| PANX1 | LINC01128 | 0.448182498 | 4.27E-56 | positive |
| CYLD | LINC01128 | 0.44247013 | 1.47E-54 | positive |
| MAP3K7 | LINC01128 | 0.440483132 | 4.95E-54 | positive |
| STAT3 | LINC01128 | 0.411773517 | 8.69E-47 | positive |
| BRAF | LINC01128 | 0.441942824 | 2.03E-54 | positive |
| ATRX | LINC01128 | 0.430480913 | 1.99E-51 | positive |
| KLF9 | LINC01128 | 0.452234912 | 3.33E-57 | positive |
| DIABLO | AC097724.1 | 0.423710707 | 1.03E-49 | positive |
| BRAF | AC097724.1 | 0.404479782 | 4.68E-45 | positive |
| ATRX | AC097724.1 | 0.438014928 | 2.22E-53 | positive |
| IPMK | AL354813.1 | 0.404313649 | 5.12E-45 | positive |
| OTULIN | AL354813.1 | 0.460644333 | 1.50E-59 | positive |
| CYLD | AL354813.1 | 0.483951522 | 2.07E-66 | positive |
| DIABLO | AL354813.1 | 0.738207112 | 3.65E-192 | positive |
| CFLAR | AL354813.1 | 0.476940651 | 2.73E-64 | positive |
| BRAF | AL354813.1 | 0.64758097 | 2.61E-133 | positive |
| ATRX | AL354813.1 | 0.515253835 | 1.69E-76 | positive |
| RNF31 | AL354813.1 | 0.447298774 | 7.42E-56 | positive |
| OTULIN | AC018557.2 | 0.428703018 | 5.65E-51 | positive |
| CYLD | AC018557.2 | 0.406014662 | 2.04E-45 | positive |
| DIABLO | AC018557.2 | 0.792395065 | 9.72E-241 | positive |
| BRAF | AC018557.2 | 0.66768754 | 1.44E-144 | positive |
| ATRX | AC018557.2 | 0.4449417 | 3.21E-55 | positive |
| DIABLO | AC129510.2 | 0.712995647 | 1.71E-173 | positive |
| BRAF | AC129510.2 | 0.581415122 | 1.21E-101 | positive |
| DIABLO | RNF216P1 | 0.410733818 | 1.54E-46 | positive |
| DIABLO | AL031705.1 | 0.545239195 | 3.37E-87 | positive |
| BRAF | AL031705.1 | 0.432860301 | 4.86E-52 | positive |
| CYLD | ZNF790-AS1 | 0.4103624 | 1.89E-46 | positive |
| DIABLO | ZNF790-AS1 | 0.591154654 | 7.57E-106 | positive |
| CFLAR | ZNF790-AS1 | 0.414171649 | 2.29E-47 | positive |
| BRAF | ZNF790-AS1 | 0.568929661 | 1.87E-96 | positive |
| ATRX | ZNF790-AS1 | 0.453881894 | 1.17E-57 | positive |
| DIABLO | AC015845.1 | 0.680653415 | 2.61E-152 | positive |
| CFLAR | AC015845.1 | 0.451650326 | 4.83E-57 | positive |
| BRAF | AC015845.1 | 0.618745133 | 1.41E-118 | positive |
| ATRX | AC015845.1 | 0.526383434 | 2.41E-80 | positive |
| OTULIN | CFLAR-AS1 | 0.440974615 | 3.67E-54 | positive |
| CYLD | CFLAR-AS1 | 0.46986351 | 3.35E-62 | positive |
| DIABLO | CFLAR-AS1 | 0.827598387 | 6.83E-281 | positive |
| CFLAR | CFLAR-AS1 | 0.505288669 | 3.59E-73 | positive |
| BRAF | CFLAR-AS1 | 0.70517868 | 4.15E-168 | positive |
| ATRX | CFLAR-AS1 | 0.445587109 | 2.15E-55 | positive |
| TSC1 | AC073046.1 | 0.413377267 | 3.57E-47 | positive |
| PANX1 | AC073046.1 | 0.448665631 | 3.16E-56 | positive |
| STAT3 | AC073046.1 | 0.424662461 | 5.93E-50 | positive |
| BRAF | AC073046.1 | 0.435590692 | 9.54E-53 | positive |
| ATRX | AC073046.1 | 0.477648966 | 1.67E-64 | positive |
| BCL2L11 | AC073046.1 | 0.424747351 | 5.65E-50 | positive |
| OTULIN | CLYBL-AS2 | 0.427166153 | 1.39E-50 | positive |
| DIABLO | CLYBL-AS2 | 0.666380216 | 8.25E-144 | positive |
| BRAF | CLYBL-AS2 | 0.559349985 | 1.27E-92 | positive |
| MAPK8 | AC010285.3 | 0.409057235 | 3.88E-46 | positive |
| OTULIN | AC010285.3 | 0.494640437 | 9.74E-70 | positive |
| CYLD | AC010285.3 | 0.41884124 | 1.66E-48 | positive |
| DIABLO | AC010285.3 | 0.715675717 | 2.22E-175 | positive |
| BRAF | AC010285.3 | 0.641427793 | 4.95E-130 | positive |
| ATRX | AC010285.3 | 0.428040731 | 8.33E-51 | positive |
| OTULIN | AC004549.1 | 0.421449016 | 3.76E-49 | positive |
| CYLD | AC004549.1 | 0.432341682 | 6.62E-52 | positive |
| DIABLO | AC004549.1 | 0.740670303 | 4.19E-194 | positive |
| CFLAR | AC004549.1 | 0.405851982 | 2.23E-45 | positive |
| BRAF | AC004549.1 | 0.662987146 | 7.39E-142 | positive |
| ATRX | AC004549.1 | 0.416148287 | 7.58E-48 | positive |
| OTULIN | GABPB1-IT1 | 0.413356164 | 3.61E-47 | positive |
| CYLD | GABPB1-IT1 | 0.479395794 | 5.00E-65 | positive |
| MAP3K7 | GABPB1-IT1 | 0.465466931 | 6.30E-61 | positive |
| DIABLO | GABPB1-IT1 | 0.584432617 | 6.26E-103 | positive |
| CFLAR | GABPB1-IT1 | 0.41449162 | 1.92E-47 | positive |
| BRAF | GABPB1-IT1 | 0.578553375 | 1.97E-100 | positive |
| BCL2 | GABPB1-IT1 | 0.406296577 | 1.75E-45 | positive |
| ATRX | GABPB1-IT1 | 0.428833075 | 5.24E-51 | positive |
| TSC1 | YEATS2-AS1 | 0.534407392 | 3.29E-83 | positive |
| OTULIN | YEATS2-AS1 | 0.552695938 | 4.94E-90 | positive |
| CYLD | YEATS2-AS1 | 0.505002479 | 4.45E-73 | positive |
| MAP3K7 | YEATS2-AS1 | 0.431369046 | 1.18E-51 | positive |
| DIABLO | YEATS2-AS1 | 0.668504568 | 4.80E-145 | positive |
| CFLAR | YEATS2-AS1 | 0.419168068 | 1.38E-48 | positive |
| BRAF | YEATS2-AS1 | 0.616692749 | 1.38E-117 | positive |
| ATRX | YEATS2-AS1 | 0.526144143 | 2.92E-80 | positive |
| RNF31 | YEATS2-AS1 | 0.522711955 | 4.64E-79 | positive |
| DIABLO | AC091588.1 | 0.67111693 | 1.41E-146 | positive |
| BRAF | AC091588.1 | 0.537522868 | 2.42E-84 | positive |
| KLF9 | AL357054.4 | 0.607072758 | 4.88E-113 | positive |
| CYLD | ADPGK-AS1 | 0.458978119 | 4.42E-59 | positive |
| DIABLO | ADPGK-AS1 | 0.667242781 | 2.61E-144 | positive |
| CFLAR | ADPGK-AS1 | 0.41648146 | 6.29E-48 | positive |
| BRAF | ADPGK-AS1 | 0.597847169 | 8.03E-109 | positive |
| ATRX | ADPGK-AS1 | 0.414724855 | 1.68E-47 | positive |
| OTULIN | AC009563.1 | 0.447726214 | 5.68E-56 | positive |
| CYLD | AC009563.1 | 0.403159726 | 9.52E-45 | positive |
| DIABLO | AC009563.1 | 0.709520454 | 4.47E-171 | positive |
| BRAF | AC009563.1 | 0.646103633 | 1.62E-132 | positive |
| ATRX | AC009563.1 | 0.408942853 | 4.13E-46 | positive |
| TSC1 | HCG25 | 0.433074612 | 4.28E-52 | positive |
| MAPK8 | HCG25 | 0.420287491 | 7.30E-49 | positive |
| OTULIN | HCG25 | 0.419187879 | 1.36E-48 | positive |
| CYLD | HCG25 | 0.471483862 | 1.13E-62 | positive |
| DIABLO | HCG25 | 0.701787178 | 7.94E-166 | positive |
| CFLAR | HCG25 | 0.52103602 | 1.77E-78 | positive |
| BRAF | HCG25 | 0.641787762 | 3.20E-130 | positive |
| ATRX | HCG25 | 0.669049984 | 2.31E-145 | positive |
| RNF31 | HCG25 | 0.459265612 | 3.67E-59 | positive |
| OTULIN | PSPC1-AS2 | 0.478016514 | 1.30E-64 | positive |
| MAP3K7 | PSPC1-AS2 | 0.475071246 | 9.82E-64 | positive |
| DIABLO | PSPC1-AS2 | 0.641126502 | 7.13E-130 | positive |
| BRAF | PSPC1-AS2 | 0.584981355 | 3.64E-103 | positive |
| ATRX | PSPC1-AS2 | 0.4067579 | 1.36E-45 | positive |
| DIABLO | HNF4A-AS1 | 0.716030818 | 1.24E-175 | positive |
| BRAF | HNF4A-AS1 | 0.597005937 | 1.92E-108 | positive |
| DIABLO | AL121748.1 | 0.469787478 | 3.53E-62 | positive |
| CFLAR | AL121748.1 | 0.420791336 | 5.48E-49 | positive |
| BRAF | AL121748.1 | 0.480285395 | 2.69E-65 | positive |
| ATRX | AL121748.1 | 0.492340049 | 5.19E-69 | positive |
| DIABLO | AC027796.1 | 0.61831425 | 2.29E-118 | positive |
| BRAF | AC027796.1 | 0.52507465 | 6.94E-80 | positive |
| ATRX | AC027796.1 | 0.457012394 | 1.57E-58 | positive |
| TSC1 | AC141002.1 | 0.428936319 | 4.93E-51 | positive |
| DIABLO | AC141002.1 | 0.581736407 | 8.87E-102 | positive |
| BRAF | AC141002.1 | 0.524200487 | 1.40E-79 | positive |
| DIABLO | LINC02003 | 0.483213209 | 3.48E-66 | positive |
| BRAF | LINC02003 | 0.446742663 | 1.05E-55 | positive |
| DIABLO | MED8-AS1 | 0.548966917 | 1.32E-88 | positive |
| BRAF | MED8-AS1 | 0.478540293 | 9.04E-65 | positive |
| CYLD | SGSM3-AS1 | 0.40976075 | 2.64E-46 | positive |
| DIABLO | SGSM3-AS1 | 0.745665671 | 4.15E-198 | positive |
| CFLAR | SGSM3-AS1 | 0.435534551 | 9.86E-53 | positive |
| BRAF | SGSM3-AS1 | 0.635625706 | 5.20E-127 | positive |
| ATRX | SGSM3-AS1 | 0.47195862 | 8.16E-63 | positive |
| GATA3 | MIR1915HG | 0.583293452 | 1.93E-102 | positive |
| TSC1 | AL445931.1 | 0.432638868 | 5.55E-52 | positive |
| DIABLO | AL445931.1 | 0.578106717 | 3.03E-100 | positive |
| BRAF | AL445931.1 | 0.447051072 | 8.66E-56 | positive |
| RNF31 | AL445931.1 | 0.490222797 | 2.39E-68 | positive |
| CD40 | AL365434.2 | 0.644989899 | 6.40E-132 | positive |
| CYLD | AC114284.1 | 0.437447429 | 3.12E-53 | positive |
| CFLAR | AC114284.1 | 0.454045747 | 1.05E-57 | positive |
| BRAF | AC114284.1 | 0.464716701 | 1.03E-60 | positive |
| DIABLO | LINC02615 | 0.480314062 | 2.64E-65 | positive |
| BRAF | LINC02615 | 0.423182473 | 1.39E-49 | positive |
| DIABLO | BX284668.6 | 0.421760438 | 3.15E-49 | positive |
| MAPK8 | AC040904.1 | 0.432685172 | 5.40E-52 | positive |
| IPMK | AC040904.1 | 0.424031643 | 8.54E-50 | positive |
| OTULIN | AC040904.1 | 0.465811296 | 5.01E-61 | positive |
| CYLD | AC040904.1 | 0.437332374 | 3.35E-53 | positive |
| DIABLO | AC040904.1 | 0.777379567 | 6.51E-226 | positive |
| CFLAR | AC040904.1 | 0.421912358 | 2.89E-49 | positive |
| BRAF | AC040904.1 | 0.702773419 | 1.74E-166 | positive |
| ATRX | AC040904.1 | 0.490623133 | 1.79E-68 | positive |
| TSC1 | ITFG1-AS1 | 0.455429065 | 4.35E-58 | positive |
| MAPK8 | ITFG1-AS1 | 0.461654291 | 7.74E-60 | positive |
| IPMK | ITFG1-AS1 | 0.494916574 | 7.97E-70 | positive |
| OTULIN | ITFG1-AS1 | 0.491069623 | 1.30E-68 | positive |
| CYLD | ITFG1-AS1 | 0.55421238 | 1.29E-90 | positive |
| MAP3K7 | ITFG1-AS1 | 0.452253852 | 3.29E-57 | positive |
| DIABLO | ITFG1-AS1 | 0.681945423 | 4.20E-153 | positive |
| CFLAR | ITFG1-AS1 | 0.504180693 | 8.27E-73 | positive |
| BRAF | ITFG1-AS1 | 0.729686521 | 1.29E-185 | positive |
| ATRX | ITFG1-AS1 | 0.612237621 | 1.85E-115 | positive |
| RNF31 | ITFG1-AS1 | 0.414613291 | 1.79E-47 | positive |
| MAPK8 | AC006017.1 | 0.402323373 | 1.49E-44 | positive |
| OTULIN | AC006017.1 | 0.462632563 | 4.08E-60 | positive |
| CYLD | AC006017.1 | 0.436338447 | 6.09E-53 | positive |
| MAP3K7 | AC006017.1 | 0.405418625 | 2.82E-45 | positive |
| DIABLO | AC006017.1 | 0.754686837 | 1.39E-205 | positive |
| CFLAR | AC006017.1 | 0.420206558 | 7.64E-49 | positive |
| BRAF | AC006017.1 | 0.710404652 | 1.09E-171 | positive |
| ATRX | AC006017.1 | 0.499746573 | 2.28E-71 | positive |
| DIABLO | AC004000.1 | 0.475916163 | 5.51E-64 | positive |
| BRAF | AC004000.1 | 0.437149921 | 3.73E-53 | positive |
| MPG | AC004000.1 | -0.427784581 | 9.67E-51 | negative |
| ATRX | AC004000.1 | 0.623098999 | 1.06E-120 | positive |
| RNF31 | AC004000.1 | 0.448583698 | 3.32E-56 | positive |
| BACH2 | LINC00402 | 0.576663466 | 1.22E-99 | positive |
| OTULIN | AC010531.3 | 0.403931696 | 6.29E-45 | positive |
| DIABLO | AC010531.3 | 0.689208647 | 1.22E-157 | positive |
| BRAF | AC010531.3 | 0.579526571 | 7.65E-101 | positive |
| TSC1 | AC005014.4 | 0.452694014 | 2.49E-57 | positive |
| MAPK8 | AC005014.4 | 0.406999429 | 1.19E-45 | positive |
| CYLD | AC005014.4 | 0.409763799 | 2.63E-46 | positive |
| DIABLO | AC005014.4 | 0.677314413 | 2.81E-150 | positive |
| CFLAR | AC005014.4 | 0.522793561 | 4.35E-79 | positive |
| BRAF | AC005014.4 | 0.639102722 | 8.19E-129 | positive |
| ATRX | AC005014.4 | 0.583103323 | 2.32E-102 | positive |
| RNF31 | AC005014.4 | 0.466661009 | 2.85E-61 | positive |
| FASLG | AC007991.2 | 0.413145227 | 4.06E-47 | positive |
| DIABLO | LRRC2-AS1 | 0.420425092 | 6.75E-49 | positive |
| DIABLO | AL683813.1 | 0.558586145 | 2.54E-92 | positive |
| BRAF | AL683813.1 | 0.509142108 | 1.91E-74 | positive |
| ATRX | AL683813.1 | 0.484773703 | 1.16E-66 | positive |
| TSC1 | NUTM2A-AS1 | 0.482859181 | 4.46E-66 | positive |
| MAPK8 | NUTM2A-AS1 | 0.561069944 | 2.67E-93 | positive |
| IPMK | NUTM2A-AS1 | 0.536793062 | 4.48E-84 | positive |
| OTULIN | NUTM2A-AS1 | 0.551433294 | 1.51E-89 | positive |
| CYLD | NUTM2A-AS1 | 0.516893349 | 4.69E-77 | positive |
| MAP3K7 | NUTM2A-AS1 | 0.533375485 | 7.77E-83 | positive |
| DIABLO | NUTM2A-AS1 | 0.647127612 | 4.58E-133 | positive |
| CFLAR | NUTM2A-AS1 | 0.518882613 | 9.77E-78 | positive |
| BRAF | NUTM2A-AS1 | 0.72266728 | 2.07E-180 | positive |
| SIRT1 | NUTM2A-AS1 | 0.435612891 | 9.41E-53 | positive |
| ATRX | NUTM2A-AS1 | 0.624132725 | 3.27E-121 | positive |
| OTULIN | AC025211.1 | 0.422869293 | 1.67E-49 | positive |
| DIABLO | AC025211.1 | 0.759299889 | 1.57E-209 | positive |
| BRAF | AC025211.1 | 0.674500288 | 1.38E-148 | positive |
| OTULIN | FBXW7-AS1 | 0.45361409 | 1.39E-57 | positive |
| DIABLO | FBXW7-AS1 | 0.651699989 | 1.51E-135 | positive |
| BRAF | FBXW7-AS1 | 0.576056396 | 2.18E-99 | positive |
| OTULIN | AL592546.3 | 0.444288643 | 4.80E-55 | positive |
| CYLD | AL592546.3 | 0.410003237 | 2.31E-46 | positive |
| DIABLO | AL592546.3 | 0.773351421 | 3.93E-222 | positive |
| BRAF | AL592546.3 | 0.675468178 | 3.63E-149 | positive |
| STAT3 | AC004596.1 | 0.442439424 | 1.50E-54 | positive |
| CYLD | AC060766.7 | 0.502478022 | 2.97E-72 | positive |
| CFLAR | AC060766.7 | 0.468129527 | 1.07E-61 | positive |
| BRAF | AC060766.7 | 0.488582655 | 7.74E-68 | positive |
| ATRX | AC060766.7 | 0.572594228 | 5.93E-98 | positive |
| OTULIN | AC092903.2 | 0.422015937 | 2.72E-49 | positive |
| DIABLO | AC092903.2 | 0.70219021 | 4.27E-166 | positive |
| BRAF | AC092903.2 | 0.604717235 | 6.00E-112 | positive |
| ATRX | AC092903.2 | 0.465151817 | 7.76E-61 | positive |
| MAPK8 | AC068790.6 | 0.419030626 | 1.49E-48 | positive |
| OTULIN | AC068790.6 | 0.479325271 | 5.25E-65 | positive |
| CYLD | AC068790.6 | 0.415814626 | 9.15E-48 | positive |
| MAP3K7 | AC068790.6 | 0.415067436 | 1.39E-47 | positive |
| DIABLO | AC068790.6 | 0.740792489 | 3.35E-194 | positive |
| BRAF | AC068790.6 | 0.677994356 | 1.09E-150 | positive |
| ATRX | AC068790.6 | 0.426123427 | 2.54E-50 | positive |
| TSC1 | NCBP2-AS1 | 0.432166102 | 7.34E-52 | positive |
| OTULIN | NCBP2-AS1 | 0.411503138 | 1.01E-46 | positive |
| DIABLO | NCBP2-AS1 | 0.453098357 | 1.93E-57 | positive |
| BRAF | NCBP2-AS1 | 0.424326268 | 7.20E-50 | positive |
| CYLD | ALG13-AS1 | 0.404940947 | 3.65E-45 | positive |
| DIABLO | ALG13-AS1 | 0.646219699 | 1.41E-132 | positive |
| CFLAR | ALG13-AS1 | 0.40870723 | 4.70E-46 | positive |
| BRAF | ALG13-AS1 | 0.62152223 | 6.29E-120 | positive |
| ATRX | ALG13-AS1 | 0.514764083 | 2.48E-76 | positive |
| MAPK8 | ACBD3-AS1 | 0.439286309 | 1.03E-53 | positive |
| IPMK | ACBD3-AS1 | 0.427175973 | 1.38E-50 | positive |
| OTULIN | ACBD3-AS1 | 0.432652402 | 5.50E-52 | positive |
| CYLD | ACBD3-AS1 | 0.419752817 | 9.89E-49 | positive |
| DIABLO | ACBD3-AS1 | 0.728166456 | 1.78E-184 | positive |
| CFLAR | ACBD3-AS1 | 0.425493387 | 3.67E-50 | positive |
| BRAF | ACBD3-AS1 | 0.659279457 | 9.38E-140 | positive |
| ATRX | ACBD3-AS1 | 0.446382077 | 1.31E-55 | positive |
| DIABLO | AL590101.1 | 0.597895473 | 7.64E-109 | positive |
| BRAF | AL590101.1 | 0.536249276 | 7.07E-84 | positive |
| DIABLO | AC105345.2 | 0.685471193 | 2.73E-155 | positive |
| BRAF | AC105345.2 | 0.588898611 | 7.34E-105 | positive |
| OTULIN | AC011933.2 | 0.450217489 | 1.19E-56 | positive |
| CYLD | AC011933.2 | 0.435214382 | 1.19E-52 | positive |
| DIABLO | AC011933.2 | 0.782348084 | 1.09E-230 | positive |
| BRAF | AC011933.2 | 0.675668714 | 2.75E-149 | positive |
| ATRX | AC011933.2 | 0.403303653 | 8.81E-45 | positive |
| STUB1 | SNHG32 | 0.519198893 | 7.61E-78 | positive |
| MPG | SNHG32 | 0.476737708 | 3.13E-64 | positive |
| CYLD | AC074366.1 | 0.40547137 | 2.74E-45 | positive |
| DIABLO | AC074366.1 | 0.731281515 | 7.99E-187 | positive |
| CFLAR | AC074366.1 | 0.425087625 | 4.64E-50 | positive |
| BRAF | AC074366.1 | 0.590482505 | 1.49E-105 | positive |
| ATRX | AC074366.1 | 0.458666308 | 5.41E-59 | positive |
| OTULIN | RHOQ-AS1 | 0.402178494 | 1.61E-44 | positive |
| DIABLO | RHOQ-AS1 | 0.643176996 | 5.89E-131 | positive |
| BRAF | RHOQ-AS1 | 0.589078186 | 6.13E-105 | positive |
| OTULIN | AL513185.1 | 0.407475469 | 9.21E-46 | positive |
| CYLD | AL513185.1 | 0.416020895 | 8.15E-48 | positive |
| DIABLO | AL513185.1 | 0.745399818 | 6.81E-198 | positive |
| CFLAR | AL513185.1 | 0.435954205 | 7.67E-53 | positive |
| BRAF | AL513185.1 | 0.661064212 | 9.19E-141 | positive |
| ATRX | AL513185.1 | 0.472691675 | 4.97E-63 | positive |
| OTULIN | AC068397.1 | 0.422554709 | 2.00E-49 | positive |
| CYLD | AC068397.1 | 0.402368249 | 1.46E-44 | positive |
| DIABLO | AC068397.1 | 0.767159549 | 1.80E-216 | positive |
| BRAF | AC068397.1 | 0.644216535 | 1.65E-131 | positive |
| ATRX | AC068397.1 | 0.429475109 | 3.59E-51 | positive |
| MAPK8 | AC105036.3 | 0.404733191 | 4.08E-45 | positive |
| OTULIN | AC105036.3 | 0.44541732 | 2.39E-55 | positive |
| CYLD | AC105036.3 | 0.457070081 | 1.52E-58 | positive |
| DIABLO | AC105036.3 | 0.694322507 | 6.43E-161 | positive |
| CFLAR | AC105036.3 | 0.414685495 | 1.72E-47 | positive |
| BRAF | AC105036.3 | 0.648416506 | 9.25E-134 | positive |
| ATRX | AC105036.3 | 0.524799238 | 8.67E-80 | positive |
| DIABLO | AC138028.2 | 0.611416034 | 4.52E-115 | positive |
| BRAF | AC138028.2 | 0.45246387 | 2.88E-57 | positive |
| CYLD | AC112250.2 | 0.464941002 | 8.92E-61 | positive |
| DIABLO | AC112250.2 | 0.625295554 | 8.70E-122 | positive |
| CFLAR | AC112250.2 | 0.434870462 | 1.47E-52 | positive |
| BRAF | AC112250.2 | 0.602828177 | 4.42E-111 | positive |
| ATRX | AC112250.2 | 0.512527289 | 1.41E-75 | positive |
| TSC1 | MIRLET7A1HG | 0.484939786 | 1.03E-66 | positive |
| DIABLO | MIRLET7A1HG | 0.594875594 | 1.71E-107 | positive |
| BRAF | MIRLET7A1HG | 0.525735004 | 4.07E-80 | positive |
| ATRX | MIRLET7A1HG | 0.402120389 | 1.66E-44 | positive |
| TSC1 | AP000766.1 | 0.457005055 | 1.58E-58 | positive |
| MAPK8 | AP000766.1 | 0.486483399 | 3.46E-67 | positive |
| IPMK | AP000766.1 | 0.479624316 | 4.27E-65 | positive |
| OTULIN | AP000766.1 | 0.455146879 | 5.22E-58 | positive |
| CYLD | AP000766.1 | 0.564766582 | 8.98E-95 | positive |
| MAP3K7 | AP000766.1 | 0.468067464 | 1.12E-61 | positive |
| DIABLO | AP000766.1 | 0.696850259 | 1.46E-162 | positive |
| CFLAR | AP000766.1 | 0.561718173 | 1.48E-93 | positive |
| BRAF | AP000766.1 | 0.791810392 | 3.87E-240 | positive |
| STUB1 | AP000766.1 | -0.407538538 | 8.90E-46 | negative |
| SIRT1 | AP000766.1 | 0.419477031 | 1.16E-48 | positive |
| MPG | AP000766.1 | -0.426762761 | 1.75E-50 | negative |
| ATRX | AP000766.1 | 0.712326805 | 5.03E-173 | positive |
| MAPK8 | AC132219.1 | 0.418598612 | 1.90E-48 | positive |
| IPMK | AC132219.1 | 0.446745667 | 1.05E-55 | positive |
| OTULIN | AC132219.1 | 0.433353279 | 3.63E-52 | positive |
| CYLD | AC132219.1 | 0.444031999 | 5.62E-55 | positive |
| DIABLO | AC132219.1 | 0.747039635 | 3.16E-199 | positive |
| CFLAR | AC132219.1 | 0.410897537 | 1.41E-46 | positive |
| BRAF | AC132219.1 | 0.696848047 | 1.46E-162 | positive |
| ATRX | AC132219.1 | 0.511025705 | 4.50E-75 | positive |
| OTULIN | LIMS1-AS1 | 0.467981812 | 1.18E-61 | positive |
| CYLD | LIMS1-AS1 | 0.505439994 | 3.20E-73 | positive |
| DIABLO | LIMS1-AS1 | 0.723716762 | 3.53E-181 | positive |
| CFLAR | LIMS1-AS1 | 0.505083357 | 4.19E-73 | positive |
| BRAF | LIMS1-AS1 | 0.688334975 | 4.34E-157 | positive |
| ATRX | LIMS1-AS1 | 0.495360966 | 5.76E-70 | positive |
| OTULIN | AC064852.1 | 0.454035921 | 1.06E-57 | positive |
| DIABLO | AC064852.1 | 0.785556307 | 7.73E-234 | positive |
| BRAF | AC064852.1 | 0.675614994 | 2.96E-149 | positive |
| TSC1 | AL445222.1 | 0.414832328 | 1.59E-47 | positive |
| DIABLO | AL445222.1 | 0.490562885 | 1.87E-68 | positive |
| BRAF | AL445222.1 | 0.441653186 | 2.42E-54 | positive |
| DIABLO | DISC1-IT1 | 0.652484954 | 5.62E-136 | positive |
| BRAF | DISC1-IT1 | 0.569931731 | 7.31E-97 | positive |
| ATRX | DISC1-IT1 | 0.428897063 | 5.04E-51 | positive |
| TSC1 | CTBP1-AS | 0.513696706 | 5.70E-76 | positive |
| DIABLO | CTBP1-AS | 0.505169892 | 3.92E-73 | positive |
| CFLAR | CTBP1-AS | 0.453668503 | 1.34E-57 | positive |
| BRAF | CTBP1-AS | 0.466911175 | 2.41E-61 | positive |
| ATRX | CTBP1-AS | 0.441277316 | 3.05E-54 | positive |
| RNF31 | CTBP1-AS | 0.574337753 | 1.13E-98 | positive |
| MAPK8 | AC073641.1 | 0.422704873 | 1.83E-49 | positive |
| IPMK | AC073641.1 | 0.431579248 | 1.04E-51 | positive |
| OTULIN | AC073641.1 | 0.440467558 | 5.00E-54 | positive |
| MAP3K7 | AC073641.1 | 0.444751732 | 3.61E-55 | positive |
| DIABLO | AC073641.1 | 0.582271319 | 5.25E-102 | positive |
| BRAF | AC073641.1 | 0.552216121 | 7.56E-90 | positive |
| MAPK8 | AC008121.2 | 0.403790369 | 6.78E-45 | positive |
| OTULIN | AC008121.2 | 0.442338693 | 1.59E-54 | positive |
| MAP3K7 | AC008121.2 | 0.405335012 | 2.95E-45 | positive |
| DIABLO | AC008121.2 | 0.645622979 | 2.94E-132 | positive |
| BRAF | AC008121.2 | 0.572835916 | 4.71E-98 | positive |
| DIABLO | AC012640.1 | 0.411426506 | 1.05E-46 | positive |
| BCL2L11 | AC010536.3 | 0.457040718 | 1.55E-58 | positive |
| OTULIN | AL512353.1 | 0.402308923 | 1.50E-44 | positive |
| CYLD | AL512353.1 | 0.418223685 | 2.35E-48 | positive |
| DIABLO | AL512353.1 | 0.435141233 | 1.25E-52 | positive |
| BRAF | AL512353.1 | 0.480042382 | 3.19E-65 | positive |
| ATRX | AL512353.1 | 0.509411049 | 1.56E-74 | positive |
| OTULIN | AC104170.1 | 0.410327632 | 1.93E-46 | positive |
| CYLD | AC104170.1 | 0.462377825 | 4.82E-60 | positive |
| DIABLO | AC104170.1 | 0.785630633 | 6.53E-234 | positive |
| CFLAR | AC104170.1 | 0.420828616 | 5.36E-49 | positive |
| BRAF | AC104170.1 | 0.683271696 | 6.37E-154 | positive |
| ATRX | AC104170.1 | 0.520475951 | 2.76E-78 | positive |
| TSC1 | Z98885.3 | 0.401667838 | 2.12E-44 | positive |
| OTULIN | Z98885.3 | 0.441346509 | 2.92E-54 | positive |
| CYLD | Z98885.3 | 0.419717703 | 1.01E-48 | positive |
| DIABLO | Z98885.3 | 0.80026638 | 5.20E-249 | positive |
| CFLAR | Z98885.3 | 0.410150232 | 2.13E-46 | positive |
| BRAF | Z98885.3 | 0.683597068 | 4.01E-154 | positive |
| ATRX | Z98885.3 | 0.458610185 | 5.61E-59 | positive |
| MAPK8 | AC100821.2 | 0.433689407 | 2.97E-52 | positive |
| IPMK | AC100821.2 | 0.43893145 | 1.27E-53 | positive |
| OTULIN | AC100821.2 | 0.466807292 | 2.59E-61 | positive |
| CYLD | AC100821.2 | 0.452174548 | 3.46E-57 | positive |
| MAP3K7 | AC100821.2 | 0.425851794 | 2.98E-50 | positive |
| DIABLO | AC100821.2 | 0.77608461 | 1.09E-224 | positive |
| CFLAR | AC100821.2 | 0.417031968 | 4.61E-48 | positive |
| BRAF | AC100821.2 | 0.717495005 | 1.13E-176 | positive |
| ATRX | AC100821.2 | 0.515154797 | 1.83E-76 | positive |
| CYLD | AC004917.1 | 0.423297711 | 1.30E-49 | positive |
| DIABLO | AC004917.1 | 0.667882737 | 1.11E-144 | positive |
| BRAF | AC004917.1 | 0.599245729 | 1.88E-109 | positive |
| TSC1 | AC011465.1 | 0.442450504 | 1.49E-54 | positive |
| DIABLO | AC011465.1 | 0.629952745 | 4.07E-124 | positive |
| CFLAR | AC011465.1 | 0.532403954 | 1.74E-82 | positive |
| BRAF | AC011465.1 | 0.601976896 | 1.08E-110 | positive |
| MPG | AC011465.1 | -0.402855181 | 1.12E-44 | negative |
| ATRX | AC011465.1 | 0.621174568 | 9.30E-120 | positive |
| RNF31 | AC011465.1 | 0.483858645 | 2.21E-66 | positive |
| CYLD | AL355581.1 | 0.458630406 | 5.54E-59 | positive |
| DIABLO | AL355581.1 | 0.66851637 | 4.73E-145 | positive |
| BRAF | AL355581.1 | 0.594486248 | 2.55E-107 | positive |
| ATRX | AL355581.1 | 0.438729946 | 1.44E-53 | positive |
| DIABLO | Z98200.1 | 0.53767193 | 2.14E-84 | positive |
| BRAF | Z98200.1 | 0.531651348 | 3.24E-82 | positive |
| ATRX | Z98200.1 | 0.470605999 | 2.03E-62 | positive |
| BACH2 | AC011407.1 | 0.500412381 | 1.39E-71 | positive |
| KLF9 | AC011407.1 | 0.456414963 | 2.31E-58 | positive |
| KLF9 | AC008808.2 | 0.521121211 | 1.65E-78 | positive |
| OTULIN | AC078950.1 | 0.422097929 | 2.60E-49 | positive |
| DIABLO | AC078950.1 | 0.763724107 | 2.10E-213 | positive |
| BRAF | AC078950.1 | 0.614581429 | 1.42E-116 | positive |
| OTULIN | AC135279.3 | 0.414009115 | 2.51E-47 | positive |
| CYLD | AC135279.3 | 0.444523456 | 4.15E-55 | positive |
| DIABLO | AC135279.3 | 0.667073189 | 3.27E-144 | positive |
| BRAF | AC135279.3 | 0.586228815 | 1.06E-103 | positive |
| ATRX | AC135279.3 | 0.426576291 | 1.96E-50 | positive |
| DIABLO | AC012676.3 | 0.672249254 | 3.01E-147 | positive |
| BRAF | AC012676.3 | 0.571172171 | 2.27E-97 | positive |
| DIABLO | CHN2-AS1 | 0.502989134 | 2.03E-72 | positive |
| BRAF | CHN2-AS1 | 0.439462561 | 9.22E-54 | positive |
| DIABLO | AC012409.1 | 0.592873713 | 1.32E-106 | positive |
| CFLAR | AC012409.1 | 0.441954225 | 2.02E-54 | positive |
| BRAF | AC012409.1 | 0.573071542 | 3.77E-98 | positive |
| ATRX | AC012409.1 | 0.470958975 | 1.60E-62 | positive |
| OTULIN | AC126182.3 | 0.420557064 | 6.26E-49 | positive |
| CYLD | AC126182.3 | 0.41802607 | 2.63E-48 | positive |
| DIABLO | AC126182.3 | 0.729958437 | 8.01E-186 | positive |
| BRAF | AC126182.3 | 0.628321289 | 2.69E-123 | positive |
| TSC1 | AL137003.1 | 0.431084441 | 1.39E-51 | positive |
| MAPK8 | AL137003.1 | 0.401601603 | 2.19E-44 | positive |
| OTULIN | AL137003.1 | 0.434070423 | 2.37E-52 | positive |
| MAP3K7 | AL137003.1 | 0.497370185 | 1.32E-70 | positive |
| DIABLO | AL137003.1 | 0.48142006 | 1.22E-65 | positive |
| BRAF | AL137003.1 | 0.560436442 | 4.75E-93 | positive |
| ATRX | AL137003.1 | 0.402792952 | 1.16E-44 | positive |
| BCL2 | LINC02812 | 0.465273346 | 7.16E-61 | positive |
| BACH2 | LINC02812 | 0.415633721 | 1.01E-47 | positive |
| CD40 | LINC02812 | 0.415172061 | 1.31E-47 | positive |
| OTULIN | AL033397.2 | 0.480937897 | 1.71E-65 | positive |
| DIABLO | AL033397.2 | 0.45908455 | 4.13E-59 | positive |
| BRAF | AL033397.2 | 0.50931957 | 1.67E-74 | positive |
| ATRX | AL033397.2 | 0.402100114 | 1.68E-44 | positive |
| DIABLO | AC007292.2 | 0.467971616 | 1.19E-61 | positive |
| SIRT3 | WAC-AS1 | 0.426237008 | 2.38E-50 | positive |
| TARDBP | WAC-AS1 | 0.508309758 | 3.61E-74 | positive |
| DIABLO | LINC00893 | 0.692914543 | 5.21E-160 | positive |
| BRAF | LINC00893 | 0.602989688 | 3.73E-111 | positive |
| ATRX | LINC00893 | 0.480683588 | 2.04E-65 | positive |
| DIABLO | AL160171.1 | 0.417997601 | 2.68E-48 | positive |
| AXL | AL139220.2 | 0.400512901 | 3.92E-44 | positive |
| ATRX | AL139220.2 | 0.40819632 | 6.21E-46 | positive |
| KLF9 | AL139220.2 | 0.548610032 | 1.80E-88 | positive |
| DIABLO | RRS1-AS1 | 0.66388294 | 2.27E-142 | positive |
| BRAF | RRS1-AS1 | 0.560533248 | 4.35E-93 | positive |
| CYLD | LINC01359 | 0.403701269 | 7.12E-45 | positive |
| DIABLO | LINC01359 | 0.720188522 | 1.31E-178 | positive |
| BRAF | LINC01359 | 0.644715373 | 8.97E-132 | positive |
| ATRX | LINC01359 | 0.46934038 | 4.76E-62 | positive |
| DIABLO | AC005540.1 | 0.663989773 | 1.97E-142 | positive |
| BRAF | AC005540.1 | 0.543117818 | 2.09E-86 | positive |
| OTULIN | AL050309.1 | 0.441711508 | 2.34E-54 | positive |
| DIABLO | AL050309.1 | 0.737631684 | 1.03E-191 | positive |
| BRAF | AL050309.1 | 0.663720507 | 2.81E-142 | positive |
| OTULIN | AL161757.2 | 0.495555349 | 4.99E-70 | positive |
| CYLD | AL161757.2 | 0.467673059 | 1.45E-61 | positive |
| DIABLO | AL161757.2 | 0.734089437 | 5.72E-189 | positive |
| BRAF | AL161757.2 | 0.685881645 | 1.51E-155 | positive |
| ATRX | AL161757.2 | 0.437185582 | 3.66E-53 | positive |
| DIABLO | AC011287.2 | 0.439493447 | 9.05E-54 | positive |
| DIABLO | FAR1-IT1 | 0.547840723 | 3.52E-88 | positive |
| BRAF | FAR1-IT1 | 0.409916619 | 2.42E-46 | positive |
| OTULIN | AL512506.1 | 0.420937244 | 5.04E-49 | positive |
| DIABLO | AL512506.1 | 0.684894156 | 6.26E-155 | positive |
| BRAF | AL512506.1 | 0.611573295 | 3.81E-115 | positive |
| ATRX | AL512506.1 | 0.400202856 | 4.63E-44 | positive |
| OTULIN | AC092376.3 | 0.413439328 | 3.45E-47 | positive |
| CYLD | AC092376.3 | 0.503462103 | 1.42E-72 | positive |
| MAP3K7 | AC092376.3 | 0.435985509 | 7.52E-53 | positive |
| DIABLO | AC092376.3 | 0.428493493 | 6.39E-51 | positive |
| BRAF | AC092376.3 | 0.56332432 | 3.39E-94 | positive |
| BACH2 | AC092376.3 | 0.439601831 | 8.47E-54 | positive |
| ATRX | AC092376.3 | 0.481680742 | 1.02E-65 | positive |
| KLF9 | AC092376.3 | 0.464314644 | 1.35E-60 | positive |
| CYLD | AL442071.2 | 0.424032954 | 8.53E-50 | positive |
| DIABLO | AL442071.2 | 0.720133056 | 1.44E-178 | positive |
| BRAF | AL442071.2 | 0.605202433 | 3.58E-112 | positive |
| CYLD | AC005332.2 | 0.449098776 | 2.41E-56 | positive |
| DIABLO | AC005332.2 | 0.487534379 | 1.64E-67 | positive |
| CFLAR | AC005332.2 | 0.47344602 | 2.98E-63 | positive |
| BRAF | AC005332.2 | 0.504856367 | 4.97E-73 | positive |
| ATRX | AC005332.2 | 0.471227957 | 1.34E-62 | positive |
| CD40 | AC096734.1 | 0.617003748 | 9.81E-118 | positive |
| TSC1 | AC055855.1 | 0.435217393 | 1.19E-52 | positive |
| DIABLO | AC055855.1 | 0.498331297 | 6.49E-71 | positive |
| BRAF | AC055855.1 | 0.492452359 | 4.78E-69 | positive |
| ATRX | AC055855.1 | 0.483535663 | 2.77E-66 | positive |
| RNF31 | AC055855.1 | 0.403500166 | 7.93E-45 | positive |
| TSC1 | AC021078.1 | 0.472927407 | 4.23E-63 | positive |
| MAPK8 | AC021078.1 | 0.423793671 | 9.79E-50 | positive |
| IPMK | AC021078.1 | 0.47949424 | 4.67E-65 | positive |
| OTULIN | AC021078.1 | 0.512014942 | 2.10E-75 | positive |
| CYLD | AC021078.1 | 0.554953731 | 6.64E-91 | positive |
| MAP3K7 | AC021078.1 | 0.430456925 | 2.02E-51 | positive |
| DIABLO | AC021078.1 | 0.712065341 | 7.66E-173 | positive |
| CFLAR | AC021078.1 | 0.492472781 | 4.71E-69 | positive |
| BRAF | AC021078.1 | 0.680250463 | 4.60E-152 | positive |
| ATRX | AC021078.1 | 0.552076389 | 8.55E-90 | positive |
| RNF31 | AC021078.1 | 0.41716072 | 4.29E-48 | positive |
| DIABLO | KCNIP2-AS1 | 0.586040748 | 1.27E-103 | positive |
| BRAF | KCNIP2-AS1 | 0.494919923 | 7.95E-70 | positive |
| BACH2 | Z95118.2 | 0.458990205 | 4.39E-59 | positive |
| CYLD | AC024337.2 | 0.429645007 | 3.25E-51 | positive |
| CFLAR | AC024337.2 | 0.427021434 | 1.51E-50 | positive |
| ATRX | AC024337.2 | 0.428215895 | 7.52E-51 | positive |
| KLF9 | AC024337.2 | 0.506415122 | 1.53E-73 | positive |
| OTULIN | AC009682.1 | 0.452436875 | 2.93E-57 | positive |
| DIABLO | AC009682.1 | 0.715855629 | 1.65E-175 | positive |
| BRAF | AC009682.1 | 0.628465433 | 2.28E-123 | positive |
| MAPK8 | AP005136.3 | 0.463706963 | 2.01E-60 | positive |
| IPMK | AP005136.3 | 0.415609636 | 1.03E-47 | positive |
| MAP3K7 | AP005136.3 | 0.409351289 | 3.30E-46 | positive |
| DIABLO | AP005136.3 | 0.564477697 | 1.17E-94 | positive |
| BRAF | AP005136.3 | 0.563926296 | 1.95E-94 | positive |
| ATRX | AP005136.3 | 0.518275711 | 1.58E-77 | positive |
| OTULIN | AIRN | 0.438167646 | 2.02E-53 | positive |
| CYLD | AIRN | 0.426167754 | 2.48E-50 | positive |
| DIABLO | AIRN | 0.806793926 | 3.71E-256 | positive |
| BRAF | AIRN | 0.68323426 | 6.72E-154 | positive |
| OTULIN | AC004943.3 | 0.410081417 | 2.21E-46 | positive |
| DIABLO | AC004943.3 | 0.636516812 | 1.80E-127 | positive |
| BRAF | AC004943.3 | 0.56375544 | 2.28E-94 | positive |
| CD40 | AC021594.2 | 0.598619886 | 3.60E-109 | positive |
| TSC1 | AC068620.1 | 0.402362083 | 1.46E-44 | positive |
| MAP3K7 | AC068620.1 | 0.401653495 | 2.13E-44 | positive |
| BRAF | AC068620.1 | 0.424862715 | 5.28E-50 | positive |
| ATRX | AC068620.1 | 0.425117669 | 4.56E-50 | positive |
| DIABLO | CRTC3-AS1 | 0.529653753 | 1.67E-81 | positive |
| BRAF | CRTC3-AS1 | 0.48639971 | 3.67E-67 | positive |
| ATRX | CRTC3-AS1 | 0.424523505 | 6.43E-50 | positive |
| DIABLO | LINC02649 | 0.58271517 | 3.40E-102 | positive |
| BRAF | LINC02649 | 0.51200821 | 2.11E-75 | positive |
| TSC1 | OCIAD1-AS1 | 0.427535602 | 1.12E-50 | positive |
| MAPK8 | OCIAD1-AS1 | 0.493011043 | 3.19E-69 | positive |
| IPMK | OCIAD1-AS1 | 0.466803367 | 2.59E-61 | positive |
| OTULIN | OCIAD1-AS1 | 0.45115322 | 6.61E-57 | positive |
| CYLD | OCIAD1-AS1 | 0.444270376 | 4.86E-55 | positive |
| MAP3K7 | OCIAD1-AS1 | 0.485556223 | 6.67E-67 | positive |
| DIABLO | OCIAD1-AS1 | 0.650761549 | 4.93E-135 | positive |
| CFLAR | OCIAD1-AS1 | 0.414406322 | 2.01E-47 | positive |
| BRAF | OCIAD1-AS1 | 0.666301116 | 9.17E-144 | positive |
| ATRX | OCIAD1-AS1 | 0.56157502 | 1.68E-93 | positive |
| OTULIN | AC006504.1 | 0.492952246 | 3.33E-69 | positive |
| DIABLO | AC006504.1 | 0.40684967 | 1.29E-45 | positive |
| BRAF | AC006504.1 | 0.456952617 | 1.64E-58 | positive |
| ATRX | AC006504.1 | 0.466179673 | 3.93E-61 | positive |
| CYLD | CLMAT3 | 0.454139251 | 9.93E-58 | positive |
| DIABLO | CLMAT3 | 0.659102377 | 1.18E-139 | positive |
| CFLAR | CLMAT3 | 0.400260144 | 4.49E-44 | positive |
| BRAF | CLMAT3 | 0.607486378 | 3.13E-113 | positive |
| OTULIN | AL096678.1 | 0.443654505 | 7.10E-55 | positive |
| MAP3K7 | AL096678.1 | 0.414937773 | 1.49E-47 | positive |
| DIABLO | AL096678.1 | 0.663957602 | 2.05E-142 | positive |
| BRAF | AL096678.1 | 0.57334225 | 2.91E-98 | positive |
| TSC1 | AL358115.1 | 0.408131156 | 6.44E-46 | positive |
| MAPK8 | AL358115.1 | 0.41010868 | 2.18E-46 | positive |
| OTULIN | AL358115.1 | 0.523323954 | 2.84E-79 | positive |
| CYLD | AL358115.1 | 0.402038835 | 1.74E-44 | positive |
| MAP3K7 | AL358115.1 | 0.406946443 | 1.23E-45 | positive |
| DIABLO | AL358115.1 | 0.764661194 | 3.10E-214 | positive |
| BRAF | AL358115.1 | 0.68481482 | 7.01E-155 | positive |
| ATRX | AL358115.1 | 0.406528038 | 1.54E-45 | positive |
| DIABLO | MIS18A-AS1 | 0.607044568 | 5.03E-113 | positive |
| BRAF | MIS18A-AS1 | 0.523953625 | 1.71E-79 | positive |
| ATRX | MIS18A-AS1 | 0.452822114 | 2.30E-57 | positive |
| OTULIN | AC005803.1 | 0.407177602 | 1.08E-45 | positive |
| CYLD | AC005803.1 | 0.426288973 | 2.31E-50 | positive |
| DIABLO | AC005803.1 | 0.787225151 | 1.69E-235 | positive |
| CFLAR | AC005803.1 | 0.410753829 | 1.53E-46 | positive |
| BRAF | AC005803.1 | 0.665234922 | 3.79E-143 | positive |
| STUB1 | U47924.2 | 0.44574886 | 1.95E-55 | positive |
| BRAF | LINC00997 | 0.442477015 | 1.46E-54 | positive |
| ATRX | LINC00997 | 0.558817185 | 2.06E-92 | positive |
| ATRX | AP003392.4 | 0.418133359 | 2.48E-48 | positive |
| DIABLO | AC012358.2 | 0.570052999 | 6.53E-97 | positive |
| BRAF | AC012358.2 | 0.527729648 | 8.06E-81 | positive |
| ATRX | AC012358.2 | 0.444491492 | 4.24E-55 | positive |
| OTULIN | TCF4-AS2 | 0.419207906 | 1.35E-48 | positive |
| CYLD | TCF4-AS2 | 0.405439262 | 2.78E-45 | positive |
| DIABLO | TCF4-AS2 | 0.666382613 | 8.23E-144 | positive |
| BRAF | TCF4-AS2 | 0.615322557 | 6.29E-117 | positive |
| CYLD | AC103591.4 | 0.547322155 | 5.53E-88 | positive |
| DIABLO | AC103591.4 | 0.420040577 | 8.40E-49 | positive |
| CFLAR | AC103591.4 | 0.501182968 | 7.82E-72 | positive |
| BRAF | AC103591.4 | 0.524422676 | 1.17E-79 | positive |
| AXL | AC103591.4 | 0.407197142 | 1.07E-45 | positive |
| ATRX | AC103591.4 | 0.597881423 | 7.75E-109 | positive |
| KLF9 | AC103591.4 | 0.597542072 | 1.10E-108 | positive |
| ATRX | ZEB1-AS1 | 0.44612776 | 1.54E-55 | positive |
| TSC1 | AC138207.4 | 0.416642916 | 5.74E-48 | positive |
| IPMK | AC138207.4 | 0.424078015 | 8.31E-50 | positive |
| OTULIN | AC138207.4 | 0.471030132 | 1.53E-62 | positive |
| CYLD | AC138207.4 | 0.569201781 | 1.45E-96 | positive |
| MAP3K7 | AC138207.4 | 0.419103983 | 1.43E-48 | positive |
| DIABLO | AC138207.4 | 0.70504965 | 5.08E-168 | positive |
| CFLAR | AC138207.4 | 0.495139583 | 6.77E-70 | positive |
| BRAF | AC138207.4 | 0.695263056 | 1.58E-161 | positive |
| ATRX | AC138207.4 | 0.577615906 | 4.86E-100 | positive |
| RNF31 | AC138207.4 | 0.434145292 | 2.26E-52 | positive |
| CD40 | MIR155HG | 0.697189897 | 8.72E-163 | positive |
| OTULIN | AC009996.1 | 0.415849943 | 8.97E-48 | positive |
| DIABLO | AC009996.1 | 0.739591792 | 2.98E-193 | positive |
| CFLAR | AC009996.1 | 0.414644991 | 1.76E-47 | positive |
| BRAF | AC009996.1 | 0.621638976 | 5.51E-120 | positive |
| ATRX | AC009996.1 | 0.440790298 | 4.11E-54 | positive |
| DIABLO | VWA8-AS1 | 0.460385311 | 1.77E-59 | positive |
| BRAF | VWA8-AS1 | 0.47445993 | 1.49E-63 | positive |
| ATRX | VWA8-AS1 | 0.404544908 | 4.52E-45 | positive |
| ATRX | AC104938.1 | 0.452419527 | 2.96E-57 | positive |
| DIABLO | AP000356.1 | 0.447108233 | 8.35E-56 | positive |
| ATRX | AP000356.1 | 0.415649221 | 1.00E-47 | positive |
| TSC1 | COX10-AS1 | 0.570202469 | 5.67E-97 | positive |
| MAPK8 | COX10-AS1 | 0.519382596 | 6.58E-78 | positive |
| IPMK | COX10-AS1 | 0.51810224 | 1.81E-77 | positive |
| OTULIN | COX10-AS1 | 0.596822751 | 2.32E-108 | positive |
| CYLD | COX10-AS1 | 0.563676295 | 2.45E-94 | positive |
| USP22 | COX10-AS1 | 0.408181696 | 6.26E-46 | positive |
| MAP3K7 | COX10-AS1 | 0.622111358 | 3.24E-120 | positive |
| DIABLO | COX10-AS1 | 0.511370509 | 3.45E-75 | positive |
| CFLAR | COX10-AS1 | 0.469770447 | 3.57E-62 | positive |
| BRAF | COX10-AS1 | 0.660731801 | 1.42E-140 | positive |
| SIRT1 | COX10-AS1 | 0.428395374 | 6.77E-51 | positive |
| ATRX | COX10-AS1 | 0.645102223 | 5.58E-132 | positive |
| RNF31 | COX10-AS1 | 0.40992166 | 2.41E-46 | positive |
| BCL2L11 | COX10-AS1 | 0.466305636 | 3.61E-61 | positive |
| CYLD | ITPKB-AS1 | 0.403871502 | 6.49E-45 | positive |
| DIABLO | ITPKB-AS1 | 0.679107466 | 2.29E-151 | positive |
| BRAF | ITPKB-AS1 | 0.58694557 | 5.17E-104 | positive |
| ATRX | ITPKB-AS1 | 0.417977499 | 2.71E-48 | positive |
| CYLD | AC093726.2 | 0.434565865 | 1.76E-52 | positive |
| DIABLO | AC093726.2 | 0.648933911 | 4.85E-134 | positive |
| CFLAR | AC093726.2 | 0.435276672 | 1.15E-52 | positive |
| BRAF | AC093726.2 | 0.588278145 | 1.37E-104 | positive |
| ATRX | AC093726.2 | 0.41222108 | 6.78E-47 | positive |
| TSC1 | AC092301.1 | 0.400462648 | 4.03E-44 | positive |
| DIABLO | AC092301.1 | 0.629275191 | 8.93E-124 | positive |
| CFLAR | AC092301.1 | 0.48979761 | 3.24E-68 | positive |
| BRAF | AC092301.1 | 0.568582647 | 2.59E-96 | positive |
| ATRX | AC092301.1 | 0.540823137 | 1.48E-85 | positive |
| RNF31 | AC092301.1 | 0.501077658 | 8.46E-72 | positive |
| OTULIN | PRMT5-AS1 | 0.400670102 | 3.61E-44 | positive |
| DIABLO | PRMT5-AS1 | 0.669428123 | 1.38E-145 | positive |
| BRAF | PRMT5-AS1 | 0.521955627 | 8.50E-79 | positive |
| RNF31 | PRMT5-AS1 | 0.439683242 | 8.06E-54 | positive |
| DIABLO | AC055811.3 | 0.692211553 | 1.48E-159 | positive |
| CFLAR | AC055811.3 | 0.406096226 | 1.95E-45 | positive |
| BRAF | AC055811.3 | 0.583867213 | 1.09E-102 | positive |
| ATRX | AC055811.3 | 0.421353713 | 3.97E-49 | positive |
| DIABLO | AC090018.2 | 0.502530398 | 2.86E-72 | positive |
| BRAF | AC090018.2 | 0.418200323 | 2.39E-48 | positive |
| TSC1 | AC024361.1 | 0.404434328 | 4.79E-45 | positive |
| DIABLO | AC024361.1 | 0.467826425 | 1.31E-61 | positive |
| BRAF | AC024361.1 | 0.419823258 | 9.51E-49 | positive |
| DIABLO | AC246817.1 | 0.543613627 | 1.37E-86 | positive |
| BRAF | AC246817.1 | 0.464730406 | 1.03E-60 | positive |
| MAPK8 | RORA-AS1 | 0.431956021 | 8.32E-52 | positive |
| IPMK | RORA-AS1 | 0.403616245 | 7.45E-45 | positive |
| OTULIN | RORA-AS1 | 0.448695926 | 3.10E-56 | positive |
| CYLD | RORA-AS1 | 0.464529877 | 1.17E-60 | positive |
| DIABLO | RORA-AS1 | 0.785805248 | 4.38E-234 | positive |
| CFLAR | RORA-AS1 | 0.434920384 | 1.42E-52 | positive |
| BRAF | RORA-AS1 | 0.702082618 | 5.04E-166 | positive |
| ATRX | RORA-AS1 | 0.457053171 | 1.53E-58 | positive |
| RNF31 | AC008105.1 | 0.440620038 | 4.56E-54 | positive |
| DIABLO | AC105137.2 | 0.441322419 | 2.97E-54 | positive |
| TSC1 | AL662884.5 | 0.4359021 | 7.91E-53 | positive |
| CYLD | AL662884.5 | 0.40871958 | 4.67E-46 | positive |
| DIABLO | AL662884.5 | 0.627778565 | 5.03E-123 | positive |
| CFLAR | AL662884.5 | 0.509219565 | 1.80E-74 | positive |
| BRAF | AL662884.5 | 0.579098064 | 1.16E-100 | positive |
| ATRX | AL662884.5 | 0.510121119 | 9.03E-75 | positive |
| KLF9 | AC004554.1 | 0.451155158 | 6.60E-57 | positive |
| DIABLO | AP002892.1 | 0.671768783 | 5.80E-147 | positive |
| BRAF | AP002892.1 | 0.602550435 | 5.92E-111 | positive |
| TSC1 | SP2-AS1 | 0.413524251 | 3.29E-47 | positive |
| MAPK8 | SP2-AS1 | 0.439407513 | 9.53E-54 | positive |
| MAP3K7 | SP2-AS1 | 0.477003026 | 2.61E-64 | positive |
| DIABLO | SP2-AS1 | 0.528979658 | 2.91E-81 | positive |
| BRAF | SP2-AS1 | 0.577441632 | 5.75E-100 | positive |
| ATRX | SP2-AS1 | 0.45476208 | 6.67E-58 | positive |
| TSC1 | AC010618.3 | 0.486096504 | 4.55E-67 | positive |
| MAPK8 | AC010618.3 | 0.42436945 | 7.03E-50 | positive |
| CYLD | AC010618.3 | 0.461095838 | 1.12E-59 | positive |
| DIABLO | AC010618.3 | 0.6688361 | 3.08E-145 | positive |
| CFLAR | AC010618.3 | 0.518357188 | 1.48E-77 | positive |
| BRAF | AC010618.3 | 0.628210309 | 3.06E-123 | positive |
| ATRX | AC010618.3 | 0.606651103 | 7.66E-113 | positive |
| RNF31 | AC010618.3 | 0.511997244 | 2.13E-75 | positive |
| FADD | AL021392.1 | 0.41015958 | 2.12E-46 | positive |
| TSC1 | AL157838.1 | 0.451305929 | 6.00E-57 | positive |
| MAPK8 | AL157838.1 | 0.409974823 | 2.34E-46 | positive |
| IPMK | AL157838.1 | 0.407376863 | 9.72E-46 | positive |
| OTULIN | AL157838.1 | 0.466116554 | 4.09E-61 | positive |
| CYLD | AL157838.1 | 0.440768677 | 4.16E-54 | positive |
| MAP3K7 | AL157838.1 | 0.443828231 | 6.38E-55 | positive |
| DIABLO | AL157838.1 | 0.731702235 | 3.83E-187 | positive |
| CFLAR | AL157838.1 | 0.496513327 | 2.47E-70 | positive |
| BRAF | AL157838.1 | 0.679876049 | 7.80E-152 | positive |
| ATRX | AL157838.1 | 0.51400564 | 4.48E-76 | positive |
| RNF31 | AL157838.1 | 0.400777005 | 3.41E-44 | positive |
| OTULIN | AC012370.1 | 0.464127871 | 1.53E-60 | positive |
| DIABLO | AC012370.1 | 0.592642785 | 1.67E-106 | positive |
| BRAF | AC012370.1 | 0.506802268 | 1.14E-73 | positive |
| DIABLO | C2-AS1 | 0.475427912 | 7.70E-64 | positive |
| AXL | AP001363.2 | 0.407337585 | 9.93E-46 | positive |
| KLF9 | AP001363.2 | 0.428250961 | 7.36E-51 | positive |
| TSC1 | ZNF32-AS1 | 0.43662914 | 5.11E-53 | positive |
| DIABLO | ZNF32-AS1 | 0.409929293 | 2.40E-46 | positive |
| OTULIN | AL929236.1 | 0.442085708 | 1.86E-54 | positive |
| CYLD | AL929236.1 | 0.407677654 | 8.25E-46 | positive |
| MAP3K7 | AL929236.1 | 0.416503772 | 6.21E-48 | positive |
| DIABLO | AL929236.1 | 0.604842012 | 5.25E-112 | positive |
| CFLAR | AL929236.1 | 0.404304042 | 5.14E-45 | positive |
| BRAF | AL929236.1 | 0.596400991 | 3.58E-108 | positive |
| ATRX | AL929236.1 | 0.517477865 | 2.96E-77 | positive |
| DIABLO | AC092868.2 | 0.588644545 | 9.47E-105 | positive |
| BRAF | AC092868.2 | 0.432922566 | 4.69E-52 | positive |
| DIABLO | AC019330.1 | 0.401330959 | 2.54E-44 | positive |
| OTULIN | AC073569.3 | 0.470736995 | 1.86E-62 | positive |
| CYLD | AC073569.3 | 0.41546723 | 1.11E-47 | positive |
| DIABLO | AC073569.3 | 0.742869796 | 7.42E-196 | positive |
| BRAF | AC073569.3 | 0.659835523 | 4.55E-140 | positive |
| OTULIN | AP000779.1 | 0.445674311 | 2.04E-55 | positive |
| CYLD | AP000779.1 | 0.406200518 | 1.84E-45 | positive |
| DIABLO | AP000779.1 | 0.753695417 | 9.57E-205 | positive |
| BRAF | AP000779.1 | 0.6760766 | 1.56E-149 | positive |
| OTULIN | AC005632.3 | 0.466168498 | 3.96E-61 | positive |
| CYLD | AC005632.3 | 0.474073956 | 1.94E-63 | positive |
| DIABLO | AC005632.3 | 0.738910332 | 1.02E-192 | positive |
| CFLAR | AC005632.3 | 0.427235159 | 1.33E-50 | positive |
| BRAF | AC005632.3 | 0.666971167 | 3.75E-144 | positive |
| ATRX | AC005632.3 | 0.426262441 | 2.35E-50 | positive |
| TARDBP | AC009065.8 | 0.466964691 | 2.33E-61 | positive |
| MPG | AC113410.4 | 0.402075992 | 1.70E-44 | positive |
| DIABLO | NPSR1-AS1 | 0.443423923 | 8.18E-55 | positive |
| BRAF | NPSR1-AS1 | 0.411678314 | 9.16E-47 | positive |
| DIABLO | LINC02795 | 0.595080804 | 1.39E-107 | positive |
| BRAF | LINC02795 | 0.517426789 | 3.08E-77 | positive |
| OTULIN | AC079385.1 | 0.41997486 | 8.72E-49 | positive |
| CYLD | AC079385.1 | 0.416970946 | 4.77E-48 | positive |
| DIABLO | AC079385.1 | 0.73243849 | 1.05E-187 | positive |
| BRAF | AC079385.1 | 0.592699254 | 1.58E-106 | positive |
| RNF31 | AC079385.1 | 0.407318967 | 1.00E-45 | positive |
| TSC1 | CCDC18-AS1 | 0.461600163 | 8.02E-60 | positive |
| CYLD | CCDC18-AS1 | 0.424092287 | 8.25E-50 | positive |
| DIABLO | CCDC18-AS1 | 0.564537741 | 1.11E-94 | positive |
| CFLAR | CCDC18-AS1 | 0.4408545 | 3.95E-54 | positive |
| BRAF | CCDC18-AS1 | 0.54036434 | 2.19E-85 | positive |
| ATRX | CCDC18-AS1 | 0.406235949 | 1.81E-45 | positive |
| DIABLO | MIR222HG | 0.608518261 | 1.03E-113 | positive |
| BRAF | MIR222HG | 0.52705713 | 1.39E-80 | positive |
| DIABLO | MAILR | 0.40660008 | 1.48E-45 | positive |
| TSC1 | AL021937.1 | 0.433341744 | 3.65E-52 | positive |
| DIABLO | AL021937.1 | 0.438335988 | 1.82E-53 | positive |
| BRAF | AL021937.1 | 0.462069644 | 5.90E-60 | positive |
| CYLD | AC060234.2 | 0.478789025 | 7.61E-65 | positive |
| DIABLO | AC060234.2 | 0.452126372 | 3.57E-57 | positive |
| BRAF | AC060234.2 | 0.412262945 | 6.63E-47 | positive |
| BCL2 | AC060234.2 | 0.470602453 | 2.04E-62 | positive |
| BACH2 | AC060234.2 | 0.48931247 | 4.59E-68 | positive |
| TSC1 | AL022328.1 | 0.542073678 | 5.11E-86 | positive |
| CFLAR | AL022328.1 | 0.432998813 | 4.48E-52 | positive |
| ATRX | AL022328.1 | 0.476093135 | 4.88E-64 | positive |
| RNF31 | AL022328.1 | 0.524428354 | 1.17E-79 | positive |
| TSC1 | AC007406.4 | 0.445587155 | 2.15E-55 | positive |
| MAPK8 | AC007406.4 | 0.435476836 | 1.02E-52 | positive |
| OTULIN | AC007406.4 | 0.411459238 | 1.03E-46 | positive |
| CYLD | AC007406.4 | 0.514721279 | 2.57E-76 | positive |
| MAP3K7 | AC007406.4 | 0.428222925 | 7.49E-51 | positive |
| DIABLO | AC007406.4 | 0.422985224 | 1.56E-49 | positive |
| CFLAR | AC007406.4 | 0.476447137 | 3.83E-64 | positive |
| BRAF | AC007406.4 | 0.586811447 | 5.91E-104 | positive |
| BCL2 | AC007406.4 | 0.439259664 | 1.04E-53 | positive |
| SIRT1 | AC007406.4 | 0.400807376 | 3.35E-44 | positive |
| BACH2 | AC007406.4 | 0.401935598 | 1.84E-44 | positive |
| ATRX | AC007406.4 | 0.627489261 | 7.03E-123 | positive |
| BCL2L11 | AC007406.4 | 0.417481004 | 3.58E-48 | positive |
| DIABLO | AC090220.1 | 0.68495578 | 5.73E-155 | positive |
| BRAF | AC090220.1 | 0.541517947 | 8.21E-86 | positive |
| BCL2L11 | AP003721.1 | 0.441506015 | 2.65E-54 | positive |
| MAPK8 | AC010761.3 | 0.438353868 | 1.81E-53 | positive |
| IPMK | AC010761.3 | 0.431223596 | 1.28E-51 | positive |
| OTULIN | AC010761.3 | 0.430299273 | 2.21E-51 | positive |
| CYLD | AC010761.3 | 0.446104464 | 1.56E-55 | positive |
| DIABLO | AC010761.3 | 0.751442598 | 7.39E-203 | positive |
| CFLAR | AC010761.3 | 0.45489281 | 6.14E-58 | positive |
| BRAF | AC010761.3 | 0.714443059 | 1.65E-174 | positive |
| ATRX | AC010761.3 | 0.533554574 | 6.69E-83 | positive |
| MAPK8 | AC007938.2 | 0.409319092 | 3.36E-46 | positive |
| IPMK | AC007938.2 | 0.412071913 | 7.37E-47 | positive |
| OTULIN | AC007938.2 | 0.46062479 | 1.52E-59 | positive |
| CYLD | AC007938.2 | 0.409367893 | 3.27E-46 | positive |
| DIABLO | AC007938.2 | 0.788801695 | 4.42E-237 | positive |
| BRAF | AC007938.2 | 0.718539319 | 2.02E-177 | positive |
| ATRX | AC007938.2 | 0.416866667 | 5.06E-48 | positive |
| GATA3 | AC103746.1 | 0.541753057 | 6.72E-86 | positive |
| CFLAR | AL023882.1 | 0.437532276 | 2.97E-53 | positive |
| ATRX | AL023882.1 | 0.531923343 | 2.58E-82 | positive |
| RNF31 | AL023882.1 | 0.409404537 | 3.21E-46 | positive |
| TSC1 | NARF-AS1 | 0.435077569 | 1.30E-52 | positive |
| DIABLO | NARF-AS1 | 0.465281749 | 7.12E-61 | positive |
| CFLAR | NARF-AS1 | 0.428506253 | 6.34E-51 | positive |
| BRAF | NARF-AS1 | 0.404185058 | 5.48E-45 | positive |
| ATRX | NARF-AS1 | 0.506184691 | 1.82E-73 | positive |
| RNF31 | NARF-AS1 | 0.443764178 | 6.63E-55 | positive |
| KLF9 | AC010478.1 | 0.506165617 | 1.85E-73 | positive |
| OTULIN | ST7-AS2 | 0.464035351 | 1.62E-60 | positive |
| CYLD | ST7-AS2 | 0.420677812 | 5.84E-49 | positive |
| DIABLO | ST7-AS2 | 0.774465379 | 3.60E-223 | positive |
| BRAF | ST7-AS2 | 0.686004898 | 1.27E-155 | positive |
| DIABLO | PLAC4 | 0.504006342 | 9.43E-73 | positive |
| AXL | LINC01711 | 0.554593189 | 9.16E-91 | positive |
| OTULIN | ETV5-AS1 | 0.422887613 | 1.65E-49 | positive |
| CYLD | ETV5-AS1 | 0.415909623 | 8.67E-48 | positive |
| DIABLO | ETV5-AS1 | 0.654328472 | 5.43E-137 | positive |
| BRAF | ETV5-AS1 | 0.636196374 | 2.64E-127 | positive |
| ATRX | ETV5-AS1 | 0.451810096 | 4.36E-57 | positive |
| DIABLO | AC016747.4 | 0.51005629 | 9.49E-75 | positive |
| BRAF | AC016747.4 | 0.482465337 | 5.88E-66 | positive |
| ATRX | AC016747.4 | 0.492302908 | 5.33E-69 | positive |
| DIABLO | AC090236.2 | 0.704405806 | 1.38E-167 | positive |
| BRAF | AC090236.2 | 0.592407503 | 2.13E-106 | positive |
| ATRX | AC090236.2 | 0.447949932 | 4.94E-56 | positive |
| OTULIN | AC092168.2 | 0.448861204 | 2.79E-56 | positive |
| DIABLO | AC092168.2 | 0.764211313 | 7.78E-214 | positive |
| BRAF | AC092168.2 | 0.650775788 | 4.84E-135 | positive |
| CYLD | AC027097.1 | 0.402376851 | 1.45E-44 | positive |
| DIABLO | AC027097.1 | 0.545638301 | 2.38E-87 | positive |
| CFLAR | AC027097.1 | 0.456140368 | 2.76E-58 | positive |
| BRAF | AC027097.1 | 0.52043743 | 2.85E-78 | positive |
| ATRX | AC027097.1 | 0.580673267 | 2.51E-101 | positive |
| TNF | AC074290.1 | 0.400560107 | 3.82E-44 | positive |
| BCL2 | AC074290.1 | 0.415559963 | 1.05E-47 | positive |
| OTULIN | AC027514.1 | 0.461507864 | 8.52E-60 | positive |
| DIABLO | AC027514.1 | 0.732667791 | 7.03E-188 | positive |
| BRAF | AC027514.1 | 0.63739847 | 6.31E-128 | positive |
| MAPK8 | AC007620.3 | 0.41943408 | 1.19E-48 | positive |
| IPMK | AC007620.3 | 0.411857078 | 8.30E-47 | positive |
| OTULIN | AC007620.3 | 0.461860515 | 6.77E-60 | positive |
| CYLD | AC007620.3 | 0.498796742 | 4.60E-71 | positive |
| MAP3K7 | AC007620.3 | 0.408705183 | 4.70E-46 | positive |
| DIABLO | AC007620.3 | 0.719663551 | 3.14E-178 | positive |
| CFLAR | AC007620.3 | 0.453256272 | 1.74E-57 | positive |
| BRAF | AC007620.3 | 0.684888556 | 6.31E-155 | positive |
| ATRX | AC007620.3 | 0.520734323 | 2.25E-78 | positive |
| CYLD | AC103858.1 | 0.4353615 | 1.09E-52 | positive |
| DIABLO | AP002985.1 | 0.411746893 | 8.82E-47 | positive |
| BRAF | AP002985.1 | 0.423956064 | 8.92E-50 | positive |
| ATRX | AP002985.1 | 0.404784599 | 3.97E-45 | positive |
| DIABLO | AL022323.1 | 0.729341016 | 2.34E-185 | positive |
| BRAF | AL022323.1 | 0.589195447 | 5.45E-105 | positive |
| GATA3 | Z98745.2 | 0.430908557 | 1.55E-51 | positive |
| CFLAR | LINC02256 | 0.413275067 | 3.78E-47 | positive |
| ATRX | LINC02256 | 0.450092646 | 1.29E-56 | positive |
| KLF9 | LINC02256 | 0.570833196 | 3.13E-97 | positive |
| DIABLO | AL110114.1 | 0.532010323 | 2.41E-82 | positive |
| BRAF | AL110114.1 | 0.525072479 | 6.95E-80 | positive |
| ATRX | AL110114.1 | 0.435130971 | 1.26E-52 | positive |
| TSC1 | Z94721.3 | 0.44017923 | 5.96E-54 | positive |
| MAPK8 | Z94721.3 | 0.424670116 | 5.91E-50 | positive |
| IPMK | Z94721.3 | 0.436071921 | 7.14E-53 | positive |
| OTULIN | Z94721.3 | 0.45644735 | 2.27E-58 | positive |
| MAP3K7 | Z94721.3 | 0.430698839 | 1.75E-51 | positive |
| DIABLO | Z94721.3 | 0.65614551 | 5.33E-138 | positive |
| CFLAR | Z94721.3 | 0.430949543 | 1.51E-51 | positive |
| BRAF | Z94721.3 | 0.620364086 | 2.31E-119 | positive |
| ATRX | Z94721.3 | 0.567124066 | 1.01E-95 | positive |
| TSC1 | AC125494.1 | 0.480133006 | 3.00E-65 | positive |
| RNF31 | AC125494.1 | 0.465953976 | 4.56E-61 | positive |
| OTULIN | AC013640.1 | 0.441926322 | 2.05E-54 | positive |
| DIABLO | AC013640.1 | 0.728746801 | 6.54E-185 | positive |
| BRAF | AC013640.1 | 0.624687967 | 1.74E-121 | positive |
| DIABLO | PHKA1-AS1 | 0.584387819 | 6.55E-103 | positive |
| BRAF | PHKA1-AS1 | 0.510107282 | 9.12E-75 | positive |
| TSC1 | AC068790.3 | 0.413360966 | 3.60E-47 | positive |
| MAPK8 | AC068790.3 | 0.424071884 | 8.34E-50 | positive |
| OTULIN | AC068790.3 | 0.484526466 | 1.38E-66 | positive |
| CYLD | AC068790.3 | 0.436224901 | 6.52E-53 | positive |
| MAP3K7 | AC068790.3 | 0.416176601 | 7.46E-48 | positive |
| DIABLO | AC068790.3 | 0.783981735 | 2.76E-232 | positive |
| CFLAR | AC068790.3 | 0.417645175 | 3.26E-48 | positive |
| BRAF | AC068790.3 | 0.701394593 | 1.45E-165 | positive |
| ATRX | AC068790.3 | 0.482753105 | 4.81E-66 | positive |
| DIABLO | AP003352.1 | 0.495455523 | 5.37E-70 | positive |
| OTULIN | AC110813.1 | 0.426564179 | 1.97E-50 | positive |
| DIABLO | AC110813.1 | 0.74341336 | 2.72E-196 | positive |
| BRAF | AC110813.1 | 0.643707361 | 3.08E-131 | positive |
| SPATA2 | AL357033.4 | 0.408813158 | 4.43E-46 | positive |
| TSC1 | AC004830.2 | 0.402992668 | 1.04E-44 | positive |
| MAPK8 | AC004830.2 | 0.412192691 | 6.89E-47 | positive |
| DIABLO | AC004830.2 | 0.503853255 | 1.06E-72 | positive |
| BRAF | AC004830.2 | 0.499367296 | 3.01E-71 | positive |
| ATRX | AC004830.2 | 0.429377905 | 3.80E-51 | positive |
| TSC1 | AL139021.2 | 0.44733593 | 7.25E-56 | positive |
| CYLD | AL139021.2 | 0.429695815 | 3.16E-51 | positive |
| DIABLO | AL139021.2 | 0.525992243 | 3.30E-80 | positive |
| BRAF | AL139021.2 | 0.508774876 | 2.53E-74 | positive |
| ATRX | AL139021.2 | 0.469987904 | 3.08E-62 | positive |
| TSC1 | HCG18 | 0.503669138 | 1.22E-72 | positive |
| MAPK8 | HCG18 | 0.52340793 | 2.66E-79 | positive |
| IPMK | HCG18 | 0.451412467 | 5.61E-57 | positive |
| OTULIN | HCG18 | 0.477748111 | 1.56E-64 | positive |
| CYLD | HCG18 | 0.415441124 | 1.13E-47 | positive |
| MAP3K7 | HCG18 | 0.546146992 | 1.53E-87 | positive |
| DIABLO | HCG18 | 0.678489345 | 5.45E-151 | positive |
| CFLAR | HCG18 | 0.524484643 | 1.12E-79 | positive |
| BRAF | HCG18 | 0.67716628 | 3.45E-150 | positive |
| ATRX | HCG18 | 0.614041837 | 2.57E-116 | positive |
| DIABLO | AC004775.1 | 0.447310426 | 7.37E-56 | positive |
| BRAF | AC004775.1 | 0.400990158 | 3.04E-44 | positive |
| MAPK8 | AC092338.1 | 0.408565228 | 5.08E-46 | positive |
| OTULIN | AC092338.1 | 0.463110056 | 2.98E-60 | positive |
| CYLD | AC092338.1 | 0.43666513 | 5.00E-53 | positive |
| DIABLO | AC092338.1 | 0.803452879 | 1.82E-252 | positive |
| CFLAR | AC092338.1 | 0.419442203 | 1.18E-48 | positive |
| BRAF | AC092338.1 | 0.70233588 | 3.41E-166 | positive |
| ATRX | AC092338.1 | 0.427451254 | 1.17E-50 | positive |
| MAPK8 | AC108463.2 | 0.40981402 | 2.56E-46 | positive |
| OTULIN | AC108463.2 | 0.428827206 | 5.25E-51 | positive |
| CYLD | AC108463.2 | 0.496331904 | 2.83E-70 | positive |
| MAP3K7 | AC108463.2 | 0.426315186 | 2.28E-50 | positive |
| DIABLO | AC108463.2 | 0.581996983 | 6.87E-102 | positive |
| CFLAR | AC108463.2 | 0.497789284 | 9.68E-71 | positive |
| BRAF | AC108463.2 | 0.606298381 | 1.12E-112 | positive |
| ATRX | AC108463.2 | 0.404564101 | 4.47E-45 | positive |
| DIABLO | AL033543.1 | 0.734392989 | 3.34E-189 | positive |
| BRAF | AL033543.1 | 0.612437568 | 1.49E-115 | positive |
| ATRX | AL033543.1 | 0.404947354 | 3.63E-45 | positive |
| DIABLO | AC115282.2 | 0.544336831 | 7.33E-87 | positive |
| BRAF | AC115282.2 | 0.519827167 | 4.63E-78 | positive |
| DIABLO | IPO9-AS1 | 0.43360489 | 3.12E-52 | positive |
| MAPK8 | AC036103.1 | 0.41462609 | 1.78E-47 | positive |
| OTULIN | AC036103.1 | 0.483435629 | 2.98E-66 | positive |
| CYLD | AC036103.1 | 0.440066725 | 6.38E-54 | positive |
| DIABLO | AC036103.1 | 0.746748952 | 5.46E-199 | positive |
| CFLAR | AC036103.1 | 0.415738368 | 9.54E-48 | positive |
| BRAF | AC036103.1 | 0.678169133 | 8.52E-151 | positive |
| ATRX | AC036103.1 | 0.425961519 | 2.80E-50 | positive |
| CYLD | TMSB15B-AS1 | 0.448383828 | 3.77E-56 | positive |
| DIABLO | TMSB15B-AS1 | 0.605076042 | 4.10E-112 | positive |
| CFLAR | TMSB15B-AS1 | 0.476359742 | 4.06E-64 | positive |
| BRAF | TMSB15B-AS1 | 0.582923803 | 2.77E-102 | positive |
| ATRX | TMSB15B-AS1 | 0.48705581 | 2.30E-67 | positive |
| MAPK8 | AC114781.2 | 0.421000043 | 4.86E-49 | positive |
| CYLD | AC114781.2 | 0.403090599 | 9.88E-45 | positive |
| DIABLO | AC114781.2 | 0.64928745 | 3.12E-134 | positive |
| CFLAR | AC114781.2 | 0.405557223 | 2.61E-45 | positive |
| BRAF | AC114781.2 | 0.625469768 | 7.13E-122 | positive |
| ATRX | AC114781.2 | 0.425870781 | 2.95E-50 | positive |
| BACH2 | LINC02731 | 0.409945225 | 2.38E-46 | positive |
| ATRX | LINC01290 | 0.451131383 | 6.70E-57 | positive |
| DIABLO | AC068205.2 | 0.401810926 | 1.96E-44 | positive |
| OTULIN | MIR181A2HG | 0.422826832 | 1.71E-49 | positive |
| DIABLO | MIR181A2HG | 0.656034181 | 6.15E-138 | positive |
| BRAF | MIR181A2HG | 0.581440422 | 1.19E-101 | positive |
| DIABLO | AL135936.1 | 0.450912905 | 7.69E-57 | positive |
| OTULIN | AL390066.2 | 0.512693235 | 1.24E-75 | positive |
| CYLD | AL390066.2 | 0.514267611 | 3.65E-76 | positive |
| DIABLO | AL390066.2 | 0.654926461 | 2.53E-137 | positive |
| CFLAR | AL390066.2 | 0.473253316 | 3.39E-63 | positive |
| BRAF | AL390066.2 | 0.649701488 | 1.86E-134 | positive |
| ATRX | AL390066.2 | 0.474932733 | 1.08E-63 | positive |
| KLF9 | LINC00924 | 0.50960202 | 1.34E-74 | positive |
| TSC1 | AL031670.1 | 0.410991693 | 1.34E-46 | positive |
| MAPK8 | AL031670.1 | 0.455911241 | 3.20E-58 | positive |
| IPMK | AL031670.1 | 0.423930048 | 9.05E-50 | positive |
| OTULIN | AL031670.1 | 0.537857292 | 1.83E-84 | positive |
| CYLD | AL031670.1 | 0.505770435 | 2.49E-73 | positive |
| MAP3K7 | AL031670.1 | 0.438398478 | 1.76E-53 | positive |
| DIABLO | AL031670.1 | 0.745920649 | 2.58E-198 | positive |
| CFLAR | AL031670.1 | 0.48642925 | 3.59E-67 | positive |
| BRAF | AL031670.1 | 0.746229136 | 1.45E-198 | positive |
| ATRX | AL031670.1 | 0.558886799 | 1.94E-92 | positive |
| RNF31 | AL031670.1 | 0.43155264 | 1.06E-51 | positive |
| MAPK8 | RSF1-IT1 | 0.407606401 | 8.57E-46 | positive |
| OTULIN | RSF1-IT1 | 0.480991519 | 1.65E-65 | positive |
| CYLD | RSF1-IT1 | 0.423515505 | 1.15E-49 | positive |
| MAP3K7 | RSF1-IT1 | 0.402604448 | 1.28E-44 | positive |
| DIABLO | RSF1-IT1 | 0.760175746 | 2.72E-210 | positive |
| BRAF | RSF1-IT1 | 0.689072837 | 1.48E-157 | positive |
| ATRX | RSF1-IT1 | 0.408530198 | 5.18E-46 | positive |
| TSC1 | AP001107.4 | 0.451968354 | 3.95E-57 | positive |
| DIABLO | AP001107.4 | 0.507610126 | 6.16E-74 | positive |
| BRAF | AP001107.4 | 0.48122903 | 1.40E-65 | positive |
| DIABLO | AC005837.4 | 0.495943015 | 3.76E-70 | positive |
| BRAF | AC005837.4 | 0.493693214 | 1.94E-69 | positive |
| ATRX | AC005837.4 | 0.476931565 | 2.74E-64 | positive |
| KLF9 | AC005837.4 | 0.412421832 | 6.07E-47 | positive |
| DIABLO | AC107959.2 | 0.555003655 | 6.35E-91 | positive |
| BRAF | AC107959.2 | 0.500329097 | 1.48E-71 | positive |
| MAPK8 | ZRANB2-AS1 | 0.420354582 | 7.03E-49 | positive |
| OTULIN | ZRANB2-AS1 | 0.412435737 | 6.02E-47 | positive |
| CYLD | ZRANB2-AS1 | 0.435682104 | 9.03E-53 | positive |
| DIABLO | ZRANB2-AS1 | 0.748221966 | 3.41E-200 | positive |
| CFLAR | ZRANB2-AS1 | 0.431565102 | 1.05E-51 | positive |
| BRAF | ZRANB2-AS1 | 0.649149872 | 3.71E-134 | positive |
| ATRX | ZRANB2-AS1 | 0.447740467 | 5.63E-56 | positive |
| OTULIN | AC130651.1 | 0.465097514 | 8.04E-61 | positive |
| CYLD | AC130651.1 | 0.446617363 | 1.13E-55 | positive |
| DIABLO | AC130651.1 | 0.773618724 | 2.22E-222 | positive |
| BRAF | AC130651.1 | 0.696341197 | 3.13E-162 | positive |
| ATRX | AC130651.1 | 0.425918658 | 2.87E-50 | positive |
| DIABLO | AC127496.1 | 0.487431633 | 1.76E-67 | positive |
| DIABLO | AC084024.1 | 0.602171799 | 8.82E-111 | positive |
| BRAF | AC084024.1 | 0.539713445 | 3.81E-85 | positive |
| ATRX | AC084024.1 | 0.539078287 | 6.53E-85 | positive |
| RNF31 | AC084024.1 | 0.408407256 | 5.54E-46 | positive |
| CYLD | AC026329.1 | 0.421383029 | 3.91E-49 | positive |
| DIABLO | AC026329.1 | 0.599974712 | 8.80E-110 | positive |
| BRAF | AC026329.1 | 0.598695204 | 3.33E-109 | positive |
| DIABLO | AL138720.1 | 0.405891135 | 2.18E-45 | positive |
| MAPK8 | AC007336.1 | 0.405429303 | 2.80E-45 | positive |
| IPMK | AC007336.1 | 0.416552082 | 6.04E-48 | positive |
| OTULIN | AC007336.1 | 0.506929535 | 1.03E-73 | positive |
| CYLD | AC007336.1 | 0.471480279 | 1.13E-62 | positive |
| MAP3K7 | AC007336.1 | 0.414936996 | 1.50E-47 | positive |
| DIABLO | AC007336.1 | 0.763308749 | 4.89E-213 | positive |
| BRAF | AC007336.1 | 0.726924679 | 1.50E-183 | positive |
| ATRX | AC007336.1 | 0.456884708 | 1.71E-58 | positive |
| OTULIN | AC022540.1 | 0.458539067 | 5.88E-59 | positive |
| CYLD | AC022540.1 | 0.400205861 | 4.62E-44 | positive |
| DIABLO | AC022540.1 | 0.744697516 | 2.52E-197 | positive |
| BRAF | AC022540.1 | 0.641479118 | 4.65E-130 | positive |
| DIABLO | AC012291.2 | 0.713066396 | 1.53E-173 | positive |
| BRAF | AC012291.2 | 0.604725959 | 5.94E-112 | positive |
| OTULIN | AL391863.1 | 0.430414297 | 2.07E-51 | positive |
| CYLD | AL391863.1 | 0.414100539 | 2.39E-47 | positive |
| DIABLO | AL391863.1 | 0.820466676 | 4.72E-272 | positive |
| BRAF | AL391863.1 | 0.67785821 | 1.32E-150 | positive |
| MAPK8 | AC024270.3 | 0.408791345 | 4.49E-46 | positive |
| OTULIN | AC024270.3 | 0.426523061 | 2.02E-50 | positive |
| CYLD | AC024270.3 | 0.430436804 | 2.04E-51 | positive |
| DIABLO | AC024270.3 | 0.801240354 | 4.64E-250 | positive |
| CFLAR | AC024270.3 | 0.425550805 | 3.55E-50 | positive |
| BRAF | AC024270.3 | 0.694855521 | 2.90E-161 | positive |
| ATRX | AC024270.3 | 0.449996477 | 1.37E-56 | positive |
| MAPK8 | AC087683.2 | 0.406475113 | 1.59E-45 | positive |
| OTULIN | AC087683.2 | 0.482846066 | 4.50E-66 | positive |
| CYLD | AC087683.2 | 0.428796543 | 5.35E-51 | positive |
| MAP3K7 | AC087683.2 | 0.425791434 | 3.09E-50 | positive |
| DIABLO | AC087683.2 | 0.756722069 | 2.58E-207 | positive |
| BRAF | AC087683.2 | 0.675924965 | 1.93E-149 | positive |
| DIABLO | AC009120.4 | 0.443125161 | 9.83E-55 | positive |
| BRAF | AC009120.4 | 0.408869567 | 4.30E-46 | positive |
| TSC1 | AC092802.2 | 0.409183573 | 3.62E-46 | positive |
| MAPK8 | AC092802.2 | 0.423115955 | 1.45E-49 | positive |
| OTULIN | AC092802.2 | 0.448881876 | 2.76E-56 | positive |
| CYLD | AC092802.2 | 0.441862308 | 2.13E-54 | positive |
| DIABLO | AC092802.2 | 0.688652983 | 2.73E-157 | positive |
| CFLAR | AC092802.2 | 0.438810722 | 1.37E-53 | positive |
| BRAF | AC092802.2 | 0.637504555 | 5.56E-128 | positive |
| ATRX | AC092802.2 | 0.503449256 | 1.43E-72 | positive |
| DIABLO | AC008883.1 | 0.711292068 | 2.65E-172 | positive |
| BRAF | AC008883.1 | 0.62730009 | 8.74E-123 | positive |
| MAPK8 | AC022558.3 | 0.425272563 | 4.17E-50 | positive |
| OTULIN | AC022558.3 | 0.47465944 | 1.30E-63 | positive |
| CYLD | AC022558.3 | 0.456201662 | 2.65E-58 | positive |
| MAP3K7 | AC022558.3 | 0.428854679 | 5.17E-51 | positive |
| DIABLO | AC022558.3 | 0.669604722 | 1.09E-145 | positive |
| CFLAR | AC022558.3 | 0.477174596 | 2.32E-64 | positive |
| BRAF | AC022558.3 | 0.639233386 | 7.00E-129 | positive |
| ATRX | AC022558.3 | 0.502787576 | 2.36E-72 | positive |
| DIABLO | AP000873.4 | 0.593394421 | 7.79E-107 | positive |
| BRAF | AP000873.4 | 0.53239507 | 1.75E-82 | positive |
| ATRX | AP000873.4 | 0.458476858 | 6.12E-59 | positive |
| DIABLO | INTS6-AS1 | 0.470916729 | 1.65E-62 | positive |
| BRAF | INTS6-AS1 | 0.429179946 | 4.27E-51 | positive |
| DIABLO | AC013286.1 | 0.613355073 | 5.45E-116 | positive |
| BRAF | AC013286.1 | 0.547647254 | 4.17E-88 | positive |
| OTULIN | AL353768.1 | 0.404658266 | 4.25E-45 | positive |
| CYLD | AL353768.1 | 0.449598806 | 1.76E-56 | positive |
| DIABLO | AL353768.1 | 0.652934835 | 3.18E-136 | positive |
| BRAF | AL353768.1 | 0.618944576 | 1.13E-118 | positive |
| MAPK8 | AF129075.1 | 0.444881437 | 3.33E-55 | positive |
| IPMK | AF129075.1 | 0.423801558 | 9.75E-50 | positive |
| OTULIN | AF129075.1 | 0.461171123 | 1.06E-59 | positive |
| CYLD | AF129075.1 | 0.443521857 | 7.70E-55 | positive |
| MAP3K7 | AF129075.1 | 0.425515748 | 3.62E-50 | positive |
| DIABLO | AF129075.1 | 0.761795286 | 1.05E-211 | positive |
| CFLAR | AF129075.1 | 0.403803647 | 6.73E-45 | positive |
| BRAF | AF129075.1 | 0.676907574 | 4.94E-150 | positive |
| ATRX | AF129075.1 | 0.43271796 | 5.29E-52 | positive |
| DIABLO | AL356124.2 | 0.61343979 | 4.97E-116 | positive |
| BRAF | AL356124.2 | 0.584616989 | 5.22E-103 | positive |
| TSC1 | AL162586.1 | 0.502298604 | 3.40E-72 | positive |
| OTULIN | STEAP3-AS1 | 0.416013833 | 8.18E-48 | positive |
| DIABLO | STEAP3-AS1 | 0.435528969 | 9.89E-53 | positive |
| BRAF | STEAP3-AS1 | 0.459240758 | 3.73E-59 | positive |
| DIABLO | AC016644.1 | 0.768233893 | 1.94E-217 | positive |
| BRAF | AC016644.1 | 0.618368663 | 2.15E-118 | positive |
| OTULIN | AC016831.4 | 0.412332944 | 6.37E-47 | positive |
| DIABLO | AC016831.4 | 0.790408574 | 1.04E-238 | positive |
| BRAF | AC016831.4 | 0.668880537 | 2.90E-145 | positive |
| CYLD | AL691447.2 | 0.423515572 | 1.15E-49 | positive |
| AXL | AL691447.2 | 0.438428135 | 1.73E-53 | positive |
| KLF9 | AL691447.2 | 0.51855585 | 1.27E-77 | positive |
| DIABLO | AC007497.1 | 0.476038028 | 5.07E-64 | positive |
| BRAF | AC007497.1 | 0.430786326 | 1.66E-51 | positive |
| DIABLO | IGBP1-AS1 | 0.476831575 | 2.94E-64 | positive |
| BRAF | IGBP1-AS1 | 0.423427619 | 1.21E-49 | positive |
| DIABLO | TSC22D1-AS1 | 0.491499986 | 9.52E-69 | positive |
| BRAF | TSC22D1-AS1 | 0.433894835 | 2.63E-52 | positive |
| ATRX | AC005972.3 | 0.42870571 | 5.64E-51 | positive |
| OTULIN | AC098851.1 | 0.468349179 | 9.25E-62 | positive |
| CYLD | AC098851.1 | 0.447547244 | 6.35E-56 | positive |
| DIABLO | AC098851.1 | 0.679688948 | 1.01E-151 | positive |
| CFLAR | AC098851.1 | 0.431749128 | 9.40E-52 | positive |
| BRAF | AC098851.1 | 0.611159937 | 5.96E-115 | positive |
| MAPK8 | AL592148.3 | 0.476588185 | 3.47E-64 | positive |
| IPMK | AL592148.3 | 0.415416362 | 1.14E-47 | positive |
| OTULIN | AL592148.3 | 0.506548301 | 1.38E-73 | positive |
| CYLD | AL592148.3 | 0.514002306 | 4.49E-76 | positive |
| MAP3K7 | AL592148.3 | 0.470968565 | 1.59E-62 | positive |
| DIABLO | AL592148.3 | 0.663451446 | 4.01E-142 | positive |
| CFLAR | AL592148.3 | 0.433122973 | 4.16E-52 | positive |
| BRAF | AL592148.3 | 0.691211314 | 6.45E-159 | positive |
| ATRX | AL592148.3 | 0.549197604 | 1.08E-88 | positive |
| DIABLO | AL121972.1 | 0.43425577 | 2.12E-52 | positive |
| OTULIN | BMP8B-AS1 | 0.410932407 | 1.38E-46 | positive |
| DIABLO | BMP8B-AS1 | 0.629705343 | 5.42E-124 | positive |
| BRAF | BMP8B-AS1 | 0.550941041 | 2.33E-89 | positive |
| DIABLO | AC004839.2 | 0.469223813 | 5.15E-62 | positive |
| BRAF | AC004839.2 | 0.483400904 | 3.05E-66 | positive |
| ATRX | AC004839.2 | 0.429696823 | 3.15E-51 | positive |
| DIABLO | AC008680.1 | 0.64424249 | 1.60E-131 | positive |
| BRAF | AC008680.1 | 0.555817436 | 3.07E-91 | positive |
| CYLD | AL109767.1 | 0.412531506 | 5.71E-47 | positive |
| DIABLO | AL109767.1 | 0.729979911 | 7.72E-186 | positive |
| BRAF | AL109767.1 | 0.593541881 | 6.70E-107 | positive |
| FASLG | LINC00892 | 0.4579821 | 8.43E-59 | positive |
| DIABLO | AC022306.3 | 0.465891037 | 4.76E-61 | positive |
| BRAF | AC022306.3 | 0.447300849 | 7.41E-56 | positive |
| MAPK8 | AC131568.1 | 0.435065945 | 1.31E-52 | positive |
| IPMK | AC131568.1 | 0.434140899 | 2.27E-52 | positive |
| OTULIN | AC131568.1 | 0.490395362 | 2.11E-68 | positive |
| CYLD | AC131568.1 | 0.523105145 | 3.39E-79 | positive |
| DIABLO | AC131568.1 | 0.801691947 | 1.51E-250 | positive |
| CFLAR | AC131568.1 | 0.536774053 | 4.55E-84 | positive |
| BRAF | AC131568.1 | 0.74346007 | 2.50E-196 | positive |
| ATRX | AC131568.1 | 0.578329934 | 2.44E-100 | positive |
| DIABLO | AC007014.1 | 0.718429842 | 2.42E-177 | positive |
| CFLAR | AC007014.1 | 0.408528338 | 5.18E-46 | positive |
| BRAF | AC007014.1 | 0.573413691 | 2.72E-98 | positive |
| ATRX | AC007014.1 | 0.433755389 | 2.86E-52 | positive |
| TRIM11 | AC245140.2 | 0.400688353 | 3.57E-44 | positive |
| MAP3K7 | AC010207.1 | 0.429718466 | 3.11E-51 | positive |
| DIABLO | AC010207.1 | 0.51885124 | 1.00E-77 | positive |
| BRAF | AC010207.1 | 0.544326441 | 7.40E-87 | positive |
| ATRX | AC010207.1 | 0.433784387 | 2.81E-52 | positive |
| BCL2 | LINC01366 | 0.405832662 | 2.25E-45 | positive |
| RNF31 | AC016738.1 | 0.499960139 | 1.94E-71 | positive |
| DIABLO | AC073912.1 | 0.578583343 | 1.91E-100 | positive |
| BRAF | AC073912.1 | 0.499508382 | 2.72E-71 | positive |
| DIABLO | AC006348.1 | 0.638706831 | 1.32E-128 | positive |
| BRAF | AC006348.1 | 0.504420505 | 6.90E-73 | positive |
| DIABLO | AC114550.2 | 0.425049379 | 4.74E-50 | positive |
| TSC1 | AC117490.2 | 0.400064783 | 4.98E-44 | positive |
| OTULIN | AC117490.2 | 0.461104027 | 1.11E-59 | positive |
| CYLD | AC117490.2 | 0.453592832 | 1.41E-57 | positive |
| DIABLO | AC117490.2 | 0.538851969 | 7.90E-85 | positive |
| BRAF | AC117490.2 | 0.539507046 | 4.54E-85 | positive |
| ATRX | AC117490.2 | 0.559006445 | 1.74E-92 | positive |
| RNF31 | AC117490.2 | 0.418862907 | 1.64E-48 | positive |
| OTULIN | GLYCTK-AS1 | 0.403738164 | 6.98E-45 | positive |
| CYLD | GLYCTK-AS1 | 0.402073377 | 1.71E-44 | positive |
| DIABLO | GLYCTK-AS1 | 0.795361143 | 8.19E-244 | positive |
| CFLAR | GLYCTK-AS1 | 0.405679626 | 2.44E-45 | positive |
| BRAF | GLYCTK-AS1 | 0.66298026 | 7.45E-142 | positive |
| OTULIN | AC090948.4 | 0.420814211 | 5.41E-49 | positive |
| CYLD | AC090948.4 | 0.444817423 | 3.46E-55 | positive |
| DIABLO | AC090948.4 | 0.751652099 | 4.94E-203 | positive |
| CFLAR | AC090948.4 | 0.407838255 | 7.56E-46 | positive |
| BRAF | AC090948.4 | 0.643909668 | 2.41E-131 | positive |
| DIABLO | AP001094.3 | 0.606378795 | 1.02E-112 | positive |
| BRAF | AP001094.3 | 0.514683485 | 2.64E-76 | positive |
| DIABLO | AC004951.1 | 0.463882647 | 1.79E-60 | positive |
| CFLAR | AC004951.1 | 0.430644976 | 1.80E-51 | positive |
| BRAF | AC004951.1 | 0.4281128 | 7.98E-51 | positive |
| MPG | AC004951.1 | -0.431066105 | 1.41E-51 | negative |
| ATRX | AC004951.1 | 0.647416855 | 3.20E-133 | positive |
| RNF31 | AC004951.1 | 0.486509889 | 3.39E-67 | positive |
| DIABLO | AC004691.1 | 0.639741481 | 3.80E-129 | positive |
| BRAF | AC004691.1 | 0.585278164 | 2.71E-103 | positive |
| DIABLO | AC025576.3 | 0.74315368 | 4.40E-196 | positive |
| BRAF | AC025576.3 | 0.610338373 | 1.45E-114 | positive |
| OTULIN | AL034405.1 | 0.438831614 | 1.35E-53 | positive |
| CYLD | AL034405.1 | 0.402524176 | 1.34E-44 | positive |
| DIABLO | AL034405.1 | 0.76942364 | 1.61E-218 | positive |
| BRAF | AL034405.1 | 0.680372749 | 3.87E-152 | positive |
| ATRX | AL034405.1 | 0.418015188 | 2.65E-48 | positive |
| DIABLO | AC004490.1 | 0.482642703 | 5.19E-66 | positive |
| DIABLO | AC135050.4 | 0.572929369 | 4.31E-98 | positive |
| BRAF | AC135050.4 | 0.469276809 | 4.97E-62 | positive |
| DIABLO | AL049830.3 | 0.60516008 | 3.75E-112 | positive |
| BRAF | AL049830.3 | 0.577472622 | 5.58E-100 | positive |
| ATRX | AL049830.3 | 0.411813792 | 8.50E-47 | positive |
| SPATA2 | CTC-338M12.4 | 0.431477738 | 1.10E-51 | positive |
| DIABLO | AC008649.2 | 0.733216951 | 2.67E-188 | positive |
| BRAF | AC008649.2 | 0.584038231 | 9.24E-103 | positive |
| DIABLO | AC093151.2 | 0.500485125 | 1.31E-71 | positive |
| DIABLO | AC000068.2 | 0.451245516 | 6.23E-57 | positive |
| BRAF | AC000068.2 | 0.403336886 | 8.66E-45 | positive |
| DIABLO | AC010327.7 | 0.420870865 | 5.23E-49 | positive |
| OTULIN | AC007448.3 | 0.437706964 | 2.67E-53 | positive |
| CYLD | AC007448.3 | 0.429818847 | 2.94E-51 | positive |
| DIABLO | AC007448.3 | 0.767907809 | 3.82E-217 | positive |
| BRAF | AC007448.3 | 0.677765518 | 1.50E-150 | positive |
| ATRX | AC007448.3 | 0.40468904 | 4.18E-45 | positive |
| DIABLO | AL139288.1 | 0.434697449 | 1.63E-52 | positive |
| BRAF | AL139288.1 | 0.414004752 | 2.52E-47 | positive |
| ATRX | AL139288.1 | 0.49805845 | 7.94E-71 | positive |
| DIABLO | AC008982.2 | 0.408122995 | 6.47E-46 | positive |
| MPG | AC008982.2 | -0.418962418 | 1.55E-48 | negative |
| ATRX | AC008982.2 | 0.402609775 | 1.28E-44 | positive |
| OTULIN | AC138207.7 | 0.405279984 | 3.04E-45 | positive |
| CYLD | AC138207.7 | 0.448039949 | 4.67E-56 | positive |
| DIABLO | AC138207.7 | 0.606238738 | 1.19E-112 | positive |
| CFLAR | AC138207.7 | 0.41075869 | 1.52E-46 | positive |
| BRAF | AC138207.7 | 0.569397483 | 1.21E-96 | positive |
| ATRX | AC138207.7 | 0.530791277 | 6.57E-82 | positive |
| TSC1 | AL021878.4 | 0.501992989 | 4.27E-72 | positive |
| DIABLO | AL021878.4 | 0.450538474 | 9.74E-57 | positive |
| BRAF | AL021878.4 | 0.420564793 | 6.23E-49 | positive |
| ATRX | AL021878.4 | 0.400739804 | 3.48E-44 | positive |
| RNF31 | AL021878.4 | 0.426555743 | 1.98E-50 | positive |
| TSC1 | SSBP3-AS1 | 0.53943492 | 4.82E-85 | positive |
| CYLD | SSBP3-AS1 | 0.423552646 | 1.13E-49 | positive |
| DIABLO | SSBP3-AS1 | 0.598067634 | 6.39E-109 | positive |
| CFLAR | SSBP3-AS1 | 0.491822285 | 7.54E-69 | positive |
| BRAF | SSBP3-AS1 | 0.581464109 | 1.16E-101 | positive |
| ATRX | SSBP3-AS1 | 0.542207559 | 4.56E-86 | positive |
| RNF31 | SSBP3-AS1 | 0.485121262 | 9.07E-67 | positive |
| DIABLO | AC004054.1 | 0.55830539 | 3.27E-92 | positive |
| BRAF | AC004054.1 | 0.497350733 | 1.34E-70 | positive |
| DIABLO | TPM1-AS | 0.584842971 | 4.17E-103 | positive |
| BRAF | TPM1-AS | 0.510650636 | 6.01E-75 | positive |
| DIABLO | AC025171.4 | 0.528174839 | 5.61E-81 | positive |
| CFLAR | AC025171.4 | 0.459095232 | 4.10E-59 | positive |
| BRAF | AC025171.4 | 0.49524942 | 6.25E-70 | positive |
| ATRX | AC025171.4 | 0.482661411 | 5.13E-66 | positive |
| DIABLO | AC004466.3 | 0.603354806 | 2.54E-111 | positive |
| BRAF | AC004466.3 | 0.542167346 | 4.72E-86 | positive |
| RNF31 | AC004466.3 | 0.410978321 | 1.35E-46 | positive |
| DIABLO | AC068631.1 | 0.418844143 | 1.66E-48 | positive |
| TSC1 | DNAJC9-AS1 | 0.401994907 | 1.78E-44 | positive |
| MAPK8 | DNAJC9-AS1 | 0.413797423 | 2.82E-47 | positive |
| DIABLO | DNAJC9-AS1 | 0.539709787 | 3.82E-85 | positive |
| BRAF | DNAJC9-AS1 | 0.473113145 | 3.73E-63 | positive |
| DIABLO | ARHGAP26-AS1 | 0.729708529 | 1.24E-185 | positive |
| BRAF | ARHGAP26-AS1 | 0.597653474 | 9.81E-109 | positive |
| DIABLO | AC138028.1 | 0.614204089 | 2.15E-116 | positive |
| BRAF | AC138028.1 | 0.444971519 | 3.15E-55 | positive |
| DIABLO | AL365271.1 | 0.704672761 | 9.14E-168 | positive |
| BRAF | AL365271.1 | 0.494248387 | 1.30E-69 | positive |
| DIABLO | AC116025.1 | 0.430994962 | 1.47E-51 | positive |
| KLF9 | AC000050.2 | 0.492649258 | 4.15E-69 | positive |
| CYLD | POT1-AS1 | 0.400554299 | 3.84E-44 | positive |
| DIABLO | POT1-AS1 | 0.545500667 | 2.68E-87 | positive |
| BRAF | POT1-AS1 | 0.566513749 | 1.78E-95 | positive |
| ATRX | POT1-AS1 | 0.450486517 | 1.01E-56 | positive |
| OTULIN | AC026202.2 | 0.424701073 | 5.80E-50 | positive |
| CYLD | AC026202.2 | 0.478243433 | 1.11E-64 | positive |
| DIABLO | AC026202.2 | 0.75866179 | 5.57E-209 | positive |
| CFLAR | AC026202.2 | 0.44658288 | 1.16E-55 | positive |
| BRAF | AC026202.2 | 0.660061076 | 3.40E-140 | positive |
| MAPK8 | AL359697.1 | 0.43277054 | 5.13E-52 | positive |
| DIABLO | AL359697.1 | 0.735349192 | 6.12E-190 | positive |
| BRAF | AL359697.1 | 0.625138827 | 1.04E-121 | positive |
| ATRX | AL359697.1 | 0.465625446 | 5.67E-61 | positive |
| CYLD | SAP30-DT | 0.429712781 | 3.12E-51 | positive |
| KLF9 | SAP30-DT | 0.458089558 | 7.86E-59 | positive |
| PANX1 | AC108488.3 | 0.409336182 | 3.33E-46 | positive |
| OTULIN | AC018628.2 | 0.448476894 | 3.55E-56 | positive |
| CYLD | AC018628.2 | 0.43202714 | 7.97E-52 | positive |
| DIABLO | AC018628.2 | 0.799627714 | 2.52E-248 | positive |
| BRAF | AC018628.2 | 0.711318587 | 2.54E-172 | positive |
| ATRX | AC018628.2 | 0.478726278 | 7.95E-65 | positive |
| OTULIN | AC020900.1 | 0.475694911 | 6.41E-64 | positive |
| CYLD | AC020900.1 | 0.424762525 | 5.60E-50 | positive |
| DIABLO | AC020900.1 | 0.749928277 | 1.34E-201 | positive |
| BRAF | AC020900.1 | 0.673946524 | 2.95E-148 | positive |
| TSC1 | AL358472.5 | 0.41131796 | 1.12E-46 | positive |
| DIABLO | AL358472.5 | 0.572827067 | 4.75E-98 | positive |
| BRAF | AL358472.5 | 0.521742399 | 1.01E-78 | positive |
| ATRX | AL358472.5 | 0.418442752 | 2.08E-48 | positive |
| RNF31 | AL358472.5 | 0.438855166 | 1.33E-53 | positive |
| ATRX | TMCO1-AS1 | 0.466412947 | 3.36E-61 | positive |
| DIABLO | AL035420.1 | 0.451713059 | 4.64E-57 | positive |
| ATRX | AL035420.1 | 0.407122183 | 1.12E-45 | positive |
| DIABLO | AC018552.2 | 0.506010682 | 2.08E-73 | positive |
| BRAF | AC018552.2 | 0.483465572 | 2.91E-66 | positive |
| HDAC9 | SERPINB9P1 | 0.552827056 | 4.40E-90 | positive |
| OTULIN | AC020891.3 | 0.434689959 | 1.63E-52 | positive |
| DIABLO | AC020891.3 | 0.606692227 | 7.33E-113 | positive |
| BRAF | AC020891.3 | 0.544630582 | 5.69E-87 | positive |
| ATRX | AC020891.3 | 0.431550348 | 1.06E-51 | positive |
| DIABLO | BX284613.2 | 0.583518135 | 1.54E-102 | positive |
| BRAF | BX284613.2 | 0.565028971 | 7.05E-95 | positive |
| DIABLO | AC016597.2 | 0.707877198 | 6.02E-170 | positive |
| BRAF | AC016597.2 | 0.605354891 | 3.05E-112 | positive |
| KLF9 | AC110491.1 | 0.432304253 | 6.77E-52 | positive |
| MAPK8 | NUTM2B-AS1 | 0.468057349 | 1.12E-61 | positive |
| IPMK | NUTM2B-AS1 | 0.445133754 | 2.85E-55 | positive |
| OTULIN | NUTM2B-AS1 | 0.466568684 | 3.03E-61 | positive |
| CYLD | NUTM2B-AS1 | 0.435355309 | 1.10E-52 | positive |
| MAP3K7 | NUTM2B-AS1 | 0.433172541 | 4.04E-52 | positive |
| DIABLO | NUTM2B-AS1 | 0.69226597 | 1.36E-159 | positive |
| CFLAR | NUTM2B-AS1 | 0.464147614 | 1.51E-60 | positive |
| BRAF | NUTM2B-AS1 | 0.691714931 | 3.07E-159 | positive |
| ATRX | NUTM2B-AS1 | 0.535952395 | 9.06E-84 | positive |
| OTULIN | AL008721.2 | 0.464774793 | 9.96E-61 | positive |
| CYLD | AL008721.2 | 0.430696661 | 1.75E-51 | positive |
| MAP3K7 | AL008721.2 | 0.416131445 | 7.66E-48 | positive |
| DIABLO | AL008721.2 | 0.62320933 | 9.34E-121 | positive |
| CFLAR | AL008721.2 | 0.422351473 | 2.24E-49 | positive |
| BRAF | AL008721.2 | 0.58489332 | 3.97E-103 | positive |
| ATRX | AL008721.2 | 0.484827348 | 1.12E-66 | positive |
| KLF9 | SENCR | 0.476443538 | 3.84E-64 | positive |
| OTULIN | AC018410.1 | 0.410588672 | 1.67E-46 | positive |
| CYLD | AC018410.1 | 0.468379658 | 9.07E-62 | positive |
| DIABLO | AC018410.1 | 0.746295307 | 1.28E-198 | positive |
| CFLAR | AC018410.1 | 0.45524483 | 4.90E-58 | positive |
| BRAF | AC018410.1 | 0.63497748 | 1.12E-126 | positive |
| ATRX | AC018410.1 | 0.453808247 | 1.23E-57 | positive |
| MAPK8 | STARD4-AS1 | 0.448705289 | 3.08E-56 | positive |
| OTULIN | STARD4-AS1 | 0.524320151 | 1.28E-79 | positive |
| CYLD | STARD4-AS1 | 0.526217642 | 2.75E-80 | positive |
| MAP3K7 | STARD4-AS1 | 0.451419352 | 5.58E-57 | positive |
| DIABLO | STARD4-AS1 | 0.729436881 | 1.98E-185 | positive |
| CFLAR | STARD4-AS1 | 0.457902052 | 8.87E-59 | positive |
| BRAF | STARD4-AS1 | 0.69941192 | 3.01E-164 | positive |
| ATRX | STARD4-AS1 | 0.483292426 | 3.29E-66 | positive |
| OTULIN | CASC15 | 0.447578362 | 6.23E-56 | positive |
| CYLD | CASC15 | 0.408291884 | 5.90E-46 | positive |
| MAP3K7 | CASC15 | 0.401314267 | 2.56E-44 | positive |
| DIABLO | CASC15 | 0.579992659 | 4.86E-101 | positive |
| BRAF | CASC15 | 0.621610971 | 5.69E-120 | positive |
| ATRX | CASC15 | 0.466147826 | 4.01E-61 | positive |
| MAPK8 | AC004908.3 | 0.404040017 | 5.93E-45 | positive |
| DIABLO | AC004908.3 | 0.541549039 | 8.00E-86 | positive |
| CFLAR | AC004908.3 | 0.427613987 | 1.07E-50 | positive |
| BRAF | AC004908.3 | 0.561237891 | 2.29E-93 | positive |
| STUB1 | AC004908.3 | -0.417330151 | 3.90E-48 | negative |
| ATRX | AC004908.3 | 0.598312184 | 4.96E-109 | positive |
| DIABLO | DARS-AS1 | 0.42689436 | 1.63E-50 | positive |
| MAPK8 | AL158163.2 | 0.422102577 | 2.59E-49 | positive |
| IPMK | AL158163.2 | 0.422172835 | 2.49E-49 | positive |
| CYLD | AL158163.2 | 0.458858008 | 4.78E-59 | positive |
| DIABLO | AL158163.2 | 0.651307957 | 2.48E-135 | positive |
| CFLAR | AL158163.2 | 0.459300507 | 3.59E-59 | positive |
| BRAF | AL158163.2 | 0.641214168 | 6.41E-130 | positive |
| ATRX | AL158163.2 | 0.545049562 | 3.96E-87 | positive |
| KLF9 | LINC02106 | 0.517188904 | 3.72E-77 | positive |
| SPATA2 | ELF3-AS1 | 0.420404451 | 6.83E-49 | positive |
| CYLD | AC067945.2 | 0.440292182 | 5.57E-54 | positive |
| DIABLO | AC067945.2 | 0.49498292 | 7.59E-70 | positive |
| CFLAR | AC067945.2 | 0.430129257 | 2.45E-51 | positive |
| BRAF | AC067945.2 | 0.488401858 | 8.81E-68 | positive |
| DIABLO | AC008635.1 | 0.564043782 | 1.75E-94 | positive |
| BRAF | AC008635.1 | 0.428563352 | 6.13E-51 | positive |
| RNF31 | AC008635.1 | 0.475910806 | 5.53E-64 | positive |
| TSC1 | AC129510.1 | 0.552256906 | 7.29E-90 | positive |
| RNF31 | AC129510.1 | 0.425613294 | 3.42E-50 | positive |
| DIABLO | AC002057.2 | 0.600044419 | 8.18E-110 | positive |
| BRAF | AC002057.2 | 0.519905096 | 4.35E-78 | positive |
| TSC1 | CASK-AS1 | 0.40984272 | 2.52E-46 | positive |
| DIABLO | CASK-AS1 | 0.465149733 | 7.77E-61 | positive |
| BRAF | CASK-AS1 | 0.46724064 | 1.94E-61 | positive |
| ATRX | CASK-AS1 | 0.41016898 | 2.11E-46 | positive |
| OTULIN | Z97652.1 | 0.419392423 | 1.21E-48 | positive |
| DIABLO | Z97652.1 | 0.702073766 | 5.11E-166 | positive |
| BRAF | Z97652.1 | 0.613966742 | 2.79E-116 | positive |
| ATRX | Z97652.1 | 0.41707802 | 4.50E-48 | positive |
| DIABLO | AC106028.2 | 0.585728069 | 1.74E-103 | positive |
| CFLAR | AC106028.2 | 0.420536956 | 6.33E-49 | positive |
| BRAF | AC106028.2 | 0.540143591 | 2.65E-85 | positive |
| ATRX | AC106028.2 | 0.580740499 | 2.35E-101 | positive |
| RNF31 | AC106028.2 | 0.457684335 | 1.02E-58 | positive |
| DIABLO | AC005280.2 | 0.499965762 | 1.93E-71 | positive |
| BRAF | AC005280.2 | 0.415084465 | 1.38E-47 | positive |
| DIABLO | AC011773.4 | 0.477720989 | 1.59E-64 | positive |
| BRAF | AC011773.4 | 0.43602534 | 7.35E-53 | positive |
| BCL2 | AL161781.2 | 0.449780134 | 1.57E-56 | positive |
| BACH2 | AL161781.2 | 0.455886471 | 3.25E-58 | positive |
| DIABLO | AC079949.1 | 0.535566798 | 1.25E-83 | positive |
| BRAF | AC079949.1 | 0.425588464 | 3.47E-50 | positive |
| MAPK8 | AC105339.2 | 0.448207835 | 4.21E-56 | positive |
| IPMK | AC105339.2 | 0.422747037 | 1.79E-49 | positive |
| OTULIN | AC105339.2 | 0.497675658 | 1.05E-70 | positive |
| MAP3K7 | AC105339.2 | 0.455576604 | 3.96E-58 | positive |
| DIABLO | AC105339.2 | 0.736263464 | 1.20E-190 | positive |
| BRAF | AC105339.2 | 0.643443594 | 4.26E-131 | positive |
| ATRX | AC105339.2 | 0.417174287 | 4.26E-48 | positive |
| DIABLO | WWOX-AS1 | 0.696223083 | 3.74E-162 | positive |
| BRAF | WWOX-AS1 | 0.605152804 | 3.78E-112 | positive |
| ATRX | WWOX-AS1 | 0.428555146 | 6.16E-51 | positive |
| OTULIN | AC005096.1 | 0.448562083 | 3.37E-56 | positive |
| CYLD | AC005096.1 | 0.423746516 | 1.01E-49 | positive |
| DIABLO | AC005096.1 | 0.817443178 | 2.01E-268 | positive |
| CFLAR | AC005096.1 | 0.406754641 | 1.36E-45 | positive |
| BRAF | AC005096.1 | 0.686464333 | 6.53E-156 | positive |
| ATRX | AC005096.1 | 0.428545521 | 6.20E-51 | positive |
| OTULIN | AC008625.1 | 0.426370916 | 2.20E-50 | positive |
| CYLD | AC008625.1 | 0.427374916 | 1.23E-50 | positive |
| DIABLO | AC008625.1 | 0.748714077 | 1.34E-200 | positive |
| BRAF | AC008625.1 | 0.66298558 | 7.40E-142 | positive |
| ATRX | AC008625.1 | 0.418123724 | 2.49E-48 | positive |
| DIABLO | AC068724.1 | 0.577906805 | 3.67E-100 | positive |
| BRAF | AC068724.1 | 0.451092259 | 6.87E-57 | positive |
| TSC1 | AC109992.2 | 0.42522654 | 4.28E-50 | positive |
| IPMK | AC109992.2 | 0.407354071 | 9.84E-46 | positive |
| CYLD | AC109992.2 | 0.476520099 | 3.64E-64 | positive |
| DIABLO | AC109992.2 | 0.596157991 | 4.59E-108 | positive |
| CFLAR | AC109992.2 | 0.468398019 | 8.96E-62 | positive |
| BRAF | AC109992.2 | 0.613577614 | 4.27E-116 | positive |
| MPG | AC109992.2 | -0.402200222 | 1.59E-44 | negative |
| ATRX | AC109992.2 | 0.655293545 | 1.59E-137 | positive |
| RNF31 | AC109992.2 | 0.468248808 | 9.89E-62 | positive |
| DIABLO | AL513164.1 | 0.70905948 | 9.28E-171 | positive |
| BRAF | AL513164.1 | 0.537966571 | 1.67E-84 | positive |
| OTULIN | AC004223.2 | 0.402963649 | 1.06E-44 | positive |
| CYLD | AC004223.2 | 0.409242202 | 3.50E-46 | positive |
| DIABLO | AC004223.2 | 0.755900499 | 1.30E-206 | positive |
| BRAF | AC004223.2 | 0.668056641 | 8.77E-145 | positive |
| OTULIN | AL451123.1 | 0.460750016 | 1.40E-59 | positive |
| CYLD | AL451123.1 | 0.473025992 | 3.96E-63 | positive |
| DIABLO | AL451123.1 | 0.711612706 | 1.58E-172 | positive |
| CFLAR | AL451123.1 | 0.472816929 | 4.56E-63 | positive |
| BRAF | AL451123.1 | 0.67890727 | 3.04E-151 | positive |
| ATRX | AL451123.1 | 0.420078966 | 8.22E-49 | positive |
| OTULIN | AC013652.1 | 0.405294281 | 3.01E-45 | positive |
| MAP3K7 | AC013652.1 | 0.411667875 | 9.21E-47 | positive |
| DIABLO | AC013652.1 | 0.477299547 | 2.13E-64 | positive |
| BRAF | AC013652.1 | 0.534657726 | 2.67E-83 | positive |
| ATRX | AC013652.1 | 0.477075811 | 2.48E-64 | positive |
| OTULIN | MYB-AS1 | 0.436185834 | 6.67E-53 | positive |
| DIABLO | MYB-AS1 | 0.665958204 | 1.45E-143 | positive |
| BRAF | MYB-AS1 | 0.570369189 | 4.85E-97 | positive |
| TSC1 | AL360020.1 | 0.482657154 | 5.14E-66 | positive |
| OTULIN | AL360020.1 | 0.408643883 | 4.86E-46 | positive |
| CYLD | AL360020.1 | 0.442848897 | 1.17E-54 | positive |
| DIABLO | AL360020.1 | 0.471853443 | 8.76E-63 | positive |
| CFLAR | AL360020.1 | 0.432135343 | 7.48E-52 | positive |
| BRAF | AL360020.1 | 0.567969067 | 4.60E-96 | positive |
| MPG | AL360020.1 | -0.410411704 | 1.84E-46 | negative |
| ATRX | AL360020.1 | 0.637155873 | 8.43E-128 | positive |
| RNF31 | AL360020.1 | 0.428452896 | 6.54E-51 | positive |
| OTULIN | INHBA-AS1 | 0.428734318 | 5.55E-51 | positive |
| CYLD | INHBA-AS1 | 0.422118156 | 2.57E-49 | positive |
| DIABLO | INHBA-AS1 | 0.590343323 | 1.72E-105 | positive |
| BRAF | INHBA-AS1 | 0.524689079 | 9.47E-80 | positive |
| CD40 | LINC02576 | 0.604342941 | 8.92E-112 | positive |
| KLF9 | AC015845.2 | 0.439243622 | 1.05E-53 | positive |
| MAPK8 | AC027544.2 | 0.430165434 | 2.39E-51 | positive |
| IPMK | AC027544.2 | 0.429040398 | 4.64E-51 | positive |
| OTULIN | AC027544.2 | 0.479754697 | 3.90E-65 | positive |
| CYLD | AC027544.2 | 0.45491512 | 6.05E-58 | positive |
| MAP3K7 | AC027544.2 | 0.401620552 | 2.17E-44 | positive |
| DIABLO | AC027544.2 | 0.776177649 | 8.91E-225 | positive |
| CFLAR | AC027544.2 | 0.417294814 | 3.98E-48 | positive |
| BRAF | AC027544.2 | 0.721665243 | 1.11E-179 | positive |
| ATRX | AC027544.2 | 0.486239975 | 4.11E-67 | positive |
| BACH2 | AC243960.1 | 0.562306778 | 8.61E-94 | positive |
| MAPK8 | MFF-DT | 0.455404002 | 4.42E-58 | positive |
| OTULIN | MFF-DT | 0.402182548 | 1.61E-44 | positive |
| CYLD | MFF-DT | 0.436166792 | 6.75E-53 | positive |
| MAP3K7 | MFF-DT | 0.483906345 | 2.14E-66 | positive |
| DIABLO | MFF-DT | 0.615404982 | 5.74E-117 | positive |
| CFLAR | MFF-DT | 0.413680139 | 3.02E-47 | positive |
| BRAF | MFF-DT | 0.63566516 | 4.97E-127 | positive |
| ATRX | MFF-DT | 0.514481855 | 3.09E-76 | positive |
| FASLG | LINC00996 | 0.478392251 | 1.00E-64 | positive |
| CYLD | LINC00996 | 0.40980928 | 2.57E-46 | positive |
| BCL2 | LINC00996 | 0.455643329 | 3.80E-58 | positive |
| CD40 | LINC00996 | 0.403977797 | 6.13E-45 | positive |
| MPG | RAB11B-AS1 | 0.418305214 | 2.25E-48 | positive |
| CYLD | AL137779.1 | 0.481789942 | 9.44E-66 | positive |
| DIABLO | AL137779.1 | 0.705573646 | 2.24E-168 | positive |
| CFLAR | AL137779.1 | 0.463266933 | 2.69E-60 | positive |
| BRAF | AL137779.1 | 0.632444457 | 2.22E-125 | positive |
| ATRX | AL137779.1 | 0.44083221 | 4.00E-54 | positive |
| DIABLO | AC100778.2 | 0.504030698 | 9.26E-73 | positive |
| BRAF | AC100778.2 | 0.415819089 | 9.12E-48 | positive |
| RNF31 | AC100778.2 | 0.423028149 | 1.52E-49 | positive |
| OTULIN | AC009269.5 | 0.443441543 | 8.09E-55 | positive |
| CYLD | AC009269.5 | 0.404079571 | 5.80E-45 | positive |
| DIABLO | AC009269.5 | 0.716660357 | 4.44E-176 | positive |
| BRAF | AC009269.5 | 0.654190738 | 6.47E-137 | positive |
| OTULIN | AC011247.1 | 0.410490059 | 1.76E-46 | positive |
| CYLD | AC011247.1 | 0.434136607 | 2.28E-52 | positive |
| DIABLO | AC011247.1 | 0.727295795 | 7.96E-184 | positive |
| CFLAR | AC011247.1 | 0.468507667 | 8.32E-62 | positive |
| BRAF | AC011247.1 | 0.652894521 | 3.35E-136 | positive |
| ATRX | AC011247.1 | 0.480749392 | 1.95E-65 | positive |
| HDAC9 | AC010247.1 | 0.410504566 | 1.75E-46 | positive |
| CD40 | AC010247.1 | 0.567856443 | 5.10E-96 | positive |
| TSC1 | C1orf220 | 0.424816841 | 5.43E-50 | positive |
| ATRX | C1orf220 | 0.400589203 | 3.77E-44 | positive |
| OTULIN | ATP11A-AS1 | 0.414451026 | 1.96E-47 | positive |
| DIABLO | ATP11A-AS1 | 0.77200403 | 6.95E-221 | positive |
| BRAF | ATP11A-AS1 | 0.63477279 | 1.43E-126 | positive |
| CYLD | AP000942.5 | 0.412342307 | 6.34E-47 | positive |
| DIABLO | AP000942.5 | 0.625281952 | 8.83E-122 | positive |
| BRAF | AP000942.5 | 0.524516317 | 1.09E-79 | positive |
| DIABLO | AC010551.2 | 0.599118315 | 2.15E-109 | positive |
| BRAF | AC010551.2 | 0.476042461 | 5.05E-64 | positive |
| OTULIN | AC000065.1 | 0.466400679 | 3.39E-61 | positive |
| CYLD | AC000065.1 | 0.404017789 | 6.00E-45 | positive |
| DIABLO | AC000065.1 | 0.738873891 | 1.09E-192 | positive |
| BRAF | AC000065.1 | 0.641811045 | 3.11E-130 | positive |
| DIABLO | AC016292.1 | 0.71149491 | 1.91E-172 | positive |
| BRAF | AC016292.1 | 0.543922423 | 1.05E-86 | positive |
| DIABLO | AC010359.3 | 0.703596321 | 4.86E-167 | positive |
| BRAF | AC010359.3 | 0.601622167 | 1.57E-110 | positive |
| KLF9 | AP001347.1 | 0.485667487 | 6.16E-67 | positive |
| DIABLO | LINC00698 | 0.47866979 | 8.27E-65 | positive |
| BRAF | LINC00698 | 0.443269346 | 9.00E-55 | positive |
| DIABLO | AC020916.1 | 0.491360901 | 1.05E-68 | positive |
| MAPK8 | AC007780.1 | 0.405583518 | 2.58E-45 | positive |
| OTULIN | AC007780.1 | 0.469030871 | 5.86E-62 | positive |
| CYLD | AC007780.1 | 0.46887406 | 6.51E-62 | positive |
| MAP3K7 | AC007780.1 | 0.415600877 | 1.03E-47 | positive |
| DIABLO | AC007780.1 | 0.803312818 | 2.59E-252 | positive |
| CFLAR | AC007780.1 | 0.430605733 | 1.85E-51 | positive |
| BRAF | AC007780.1 | 0.690774686 | 1.23E-158 | positive |
| ATRX | AC007780.1 | 0.440129433 | 6.15E-54 | positive |
| DIABLO | AL353708.3 | 0.483002761 | 4.03E-66 | positive |
| OTULIN | AC100763.1 | 0.459257158 | 3.69E-59 | positive |
| CYLD | AC100763.1 | 0.417615319 | 3.32E-48 | positive |
| DIABLO | AC100763.1 | 0.76289443 | 1.14E-212 | positive |
| BRAF | AC100763.1 | 0.66939026 | 1.46E-145 | positive |
| OTULIN | AC009268.2 | 0.417499153 | 3.55E-48 | positive |
| DIABLO | AC009268.2 | 0.57925486 | 9.96E-101 | positive |
| BRAF | AC009268.2 | 0.518691302 | 1.14E-77 | positive |
| DIABLO | AL138962.1 | 0.53755459 | 2.36E-84 | positive |
| BRAF | AL138962.1 | 0.462641631 | 4.06E-60 | positive |
| DIABLO | AL121820.3 | 0.45931111 | 3.56E-59 | positive |
| BRAF | AL121820.3 | 0.415551216 | 1.06E-47 | positive |
| BCL2 | AL121820.3 | 0.407348571 | 9.87E-46 | positive |
| BCL2L11 | AL121820.3 | 0.42193672 | 2.85E-49 | positive |
| OTULIN | AC005343.4 | 0.412594243 | 5.51E-47 | positive |
| CYLD | AC005343.4 | 0.411121516 | 1.25E-46 | positive |
| DIABLO | AC005343.4 | 0.827305054 | 1.61E-280 | positive |
| BRAF | AC005343.4 | 0.672173187 | 3.34E-147 | positive |
| ATRX | AC005343.4 | 0.419129439 | 1.41E-48 | positive |
| DIABLO | SCG5-AS1 | 0.504634897 | 5.87E-73 | positive |
| SIRT3 | MAPKAPK5-AS1 | 0.436286093 | 6.28E-53 | positive |
| MYC | MAPKAPK5-AS1 | 0.439068074 | 1.17E-53 | positive |
| MPG | MAPKAPK5-AS1 | 0.446922614 | 9.38E-56 | positive |
| ATRX | MAPKAPK5-AS1 | -0.429235224 | 4.14E-51 | negative |
| TARDBP | MAPKAPK5-AS1 | 0.418140805 | 2.47E-48 | positive |
| MAPK8 | AC004832.4 | 0.431307127 | 1.22E-51 | positive |
| IPMK | AC004832.4 | 0.411845463 | 8.35E-47 | positive |
| OTULIN | AC004832.4 | 0.48422269 | 1.71E-66 | positive |
| CYLD | AC004832.4 | 0.478356061 | 1.03E-64 | positive |
| MAP3K7 | AC004832.4 | 0.405507983 | 2.68E-45 | positive |
| DIABLO | AC004832.4 | 0.775603072 | 3.09E-224 | positive |
| CFLAR | AC004832.4 | 0.42567567 | 3.30E-50 | positive |
| BRAF | AC004832.4 | 0.72757047 | 4.97E-184 | positive |
| ATRX | AC004832.4 | 0.484083537 | 1.89E-66 | positive |
| DIABLO | HOXA-AS2 | 0.473346334 | 3.18E-63 | positive |
| BRAF | HOXA-AS2 | 0.461855596 | 6.79E-60 | positive |
| ATRX | HOXA-AS2 | 0.406829021 | 1.31E-45 | positive |
| DIABLO | AL596218.1 | 0.67178025 | 5.71E-147 | positive |
| BRAF | AL596218.1 | 0.582139232 | 5.98E-102 | positive |
| TSC1 | AC008735.4 | 0.458194014 | 7.35E-59 | positive |
| DIABLO | AC008735.4 | 0.486299418 | 3.94E-67 | positive |
| CFLAR | AC008735.4 | 0.458016989 | 8.24E-59 | positive |
| BRAF | AC008735.4 | 0.470188752 | 2.69E-62 | positive |
| ATRX | AC008735.4 | 0.457579212 | 1.09E-58 | positive |
| RNF31 | AC008735.4 | 0.40987386 | 2.48E-46 | positive |
| DIABLO | AC021321.1 | 0.463731661 | 1.98E-60 | positive |
| BRAF | AC021321.1 | 0.441886406 | 2.10E-54 | positive |
| MAPK8 | AC110611.1 | 0.451744405 | 4.55E-57 | positive |
| IPMK | AC110611.1 | 0.455139082 | 5.24E-58 | positive |
| OTULIN | AC110611.1 | 0.471466952 | 1.14E-62 | positive |
| CYLD | AC110611.1 | 0.480627426 | 2.12E-65 | positive |
| MAP3K7 | AC110611.1 | 0.43745912 | 3.10E-53 | positive |
| DIABLO | AC110611.1 | 0.754034608 | 4.95E-205 | positive |
| CFLAR | AC110611.1 | 0.470274464 | 2.54E-62 | positive |
| BRAF | AC110611.1 | 0.718538473 | 2.02E-177 | positive |
| ATRX | AC110611.1 | 0.548930557 | 1.36E-88 | positive |
| MAPK8 | AC124283.3 | 0.419296351 | 1.28E-48 | positive |
| IPMK | AC124283.3 | 0.40704734 | 1.16E-45 | positive |
| OTULIN | AC124283.3 | 0.485722764 | 5.93E-67 | positive |
| CYLD | AC124283.3 | 0.453717148 | 1.30E-57 | positive |
| DIABLO | AC124283.3 | 0.809975527 | 9.69E-260 | positive |
| CFLAR | AC124283.3 | 0.418814871 | 1.68E-48 | positive |
| BRAF | AC124283.3 | 0.716435098 | 6.42E-176 | positive |
| ATRX | AC124283.3 | 0.479183563 | 5.79E-65 | positive |
| TSC1 | AC018690.1 | 0.500692726 | 1.13E-71 | positive |
| MAPK8 | AC018690.1 | 0.429328486 | 3.92E-51 | positive |
| OTULIN | AC018690.1 | 0.448161135 | 4.33E-56 | positive |
| MAP3K7 | AC018690.1 | 0.437944109 | 2.31E-53 | positive |
| DIABLO | AC018690.1 | 0.49769541 | 1.04E-70 | positive |
| CFLAR | AC018690.1 | 0.412828173 | 4.84E-47 | positive |
| BRAF | AC018690.1 | 0.55669024 | 1.40E-91 | positive |
| ATRX | AC018690.1 | 0.530839414 | 6.32E-82 | positive |
| RNF31 | AC018690.1 | 0.538149106 | 1.43E-84 | positive |
| DIABLO | AL357315.1 | 0.552614199 | 5.31E-90 | positive |
| BRAF | AL357315.1 | 0.454313805 | 8.88E-58 | positive |
| MAPK8 | AC068189.2 | 0.407296671 | 1.02E-45 | positive |
| IPMK | AC068189.2 | 0.418709642 | 1.79E-48 | positive |
| OTULIN | AC068189.2 | 0.478492441 | 9.35E-65 | positive |
| CYLD | AC068189.2 | 0.423791564 | 9.81E-50 | positive |
| DIABLO | AC068189.2 | 0.80070176 | 1.77E-249 | positive |
| BRAF | AC068189.2 | 0.71661133 | 4.81E-176 | positive |
| ATRX | AC068189.2 | 0.465430134 | 6.45E-61 | positive |
| MAPK8 | AP000866.6 | 0.465872387 | 4.81E-61 | positive |
| IPMK | AP000866.6 | 0.454820492 | 6.43E-58 | positive |
| OTULIN | AP000866.6 | 0.499493775 | 2.74E-71 | positive |
| CYLD | AP000866.6 | 0.454377884 | 8.53E-58 | positive |
| MAP3K7 | AP000866.6 | 0.434665269 | 1.66E-52 | positive |
| DIABLO | AP000866.6 | 0.799181404 | 7.55E-248 | positive |
| CFLAR | AP000866.6 | 0.443633369 | 7.19E-55 | positive |
| BRAF | AP000866.6 | 0.754484322 | 2.07E-205 | positive |
| ATRX | AP000866.6 | 0.510658926 | 5.97E-75 | positive |
| DIABLO | AC032044.1 | 0.451699174 | 4.68E-57 | positive |
| RNF31 | AC032044.1 | 0.501328911 | 7.01E-72 | positive |
| GATA3 | AL035603.1 | 0.550256591 | 4.25E-89 | positive |
| OTULIN | AL513188.1 | 0.445404998 | 2.41E-55 | positive |
| CYLD | AL513188.1 | 0.417349225 | 3.86E-48 | positive |
| DIABLO | AL513188.1 | 0.754952553 | 8.29E-206 | positive |
| BRAF | AL513188.1 | 0.673877705 | 3.25E-148 | positive |
| ATRX | AL513188.1 | 0.420884518 | 5.19E-49 | positive |
| DIABLO | LINC01655 | 0.575989918 | 2.32E-99 | positive |
| BRAF | LINC01655 | 0.513074879 | 9.23E-76 | positive |
| DIABLO | AC020910.2 | 0.684579805 | 9.82E-155 | positive |
| BRAF | AC020910.2 | 0.60314556 | 3.16E-111 | positive |
| ATRX | AC020910.2 | 0.487362021 | 1.85E-67 | positive |
| OTULIN | AL390774.2 | 0.421771109 | 3.13E-49 | positive |
| DIABLO | AL390774.2 | 0.780290854 | 1.07E-228 | positive |
| BRAF | AL390774.2 | 0.661233173 | 7.37E-141 | positive |
| MAPK8 | AC090181.2 | 0.403948953 | 6.23E-45 | positive |
| DIABLO | AC090181.2 | 0.73308734 | 3.36E-188 | positive |
| BRAF | AC090181.2 | 0.629410795 | 7.63E-124 | positive |
| DIABLO | LINC02257 | 0.474529608 | 1.42E-63 | positive |
| BRAF | LINC02257 | 0.42734002 | 1.25E-50 | positive |
| MAPK8 | AC078883.1 | 0.429293171 | 4.00E-51 | positive |
| IPMK | AC078883.1 | 0.424324106 | 7.21E-50 | positive |
| OTULIN | AC078883.1 | 0.469408338 | 4.55E-62 | positive |
| CYLD | AC078883.1 | 0.416197607 | 7.38E-48 | positive |
| MAP3K7 | AC078883.1 | 0.409501389 | 3.04E-46 | positive |
| DIABLO | AC078883.1 | 0.718205944 | 3.50E-177 | positive |
| CFLAR | AC078883.1 | 0.437038391 | 3.99E-53 | positive |
| BRAF | AC078883.1 | 0.655257812 | 1.66E-137 | positive |
| ATRX | AC078883.1 | 0.451804978 | 4.38E-57 | positive |
| CYLD | NR2F2-AS1 | 0.440909905 | 3.82E-54 | positive |
| DIABLO | NR2F2-AS1 | 0.594879857 | 1.71E-107 | positive |
| CFLAR | NR2F2-AS1 | 0.517129675 | 3.89E-77 | positive |
| BRAF | NR2F2-AS1 | 0.555310296 | 4.83E-91 | positive |
| ATRX | NR2F2-AS1 | 0.515944378 | 9.87E-77 | positive |
| KLF9 | NR2F2-AS1 | 0.505443513 | 3.19E-73 | positive |
| DIABLO | AC002401.1 | 0.60245258 | 6.56E-111 | positive |
| BRAF | AC002401.1 | 0.530237519 | 1.04E-81 | positive |
| DIABLO | AC006547.1 | 0.409043914 | 3.91E-46 | positive |
| MPG | AC006547.1 | -0.41664429 | 5.74E-48 | negative |
| ATRX | AC006547.1 | 0.499661225 | 2.42E-71 | positive |
| DIABLO | AC025423.1 | 0.606747088 | 6.91E-113 | positive |
| BRAF | AC025423.1 | 0.50111339 | 8.23E-72 | positive |
| OTULIN | AC135893.1 | 0.40550352 | 2.69E-45 | positive |
| CYLD | AC135893.1 | 0.434891817 | 1.45E-52 | positive |
| DIABLO | AC135893.1 | 0.592141793 | 2.78E-106 | positive |
| BRAF | AC135893.1 | 0.557326055 | 7.92E-92 | positive |
| ATRX | AC135893.1 | 0.462768281 | 3.73E-60 | positive |
| TSC1 | AC009716.1 | 0.444211075 | 5.04E-55 | positive |
| MAPK8 | AC009716.1 | 0.420078753 | 8.22E-49 | positive |
| IPMK | AC009716.1 | 0.412842527 | 4.80E-47 | positive |
| OTULIN | AC009716.1 | 0.505976435 | 2.13E-73 | positive |
| CYLD | AC009716.1 | 0.507905351 | 4.92E-74 | positive |
| DIABLO | AC009716.1 | 0.633984114 | 3.63E-126 | positive |
| CFLAR | AC009716.1 | 0.467124188 | 2.10E-61 | positive |
| BRAF | AC009716.1 | 0.679630675 | 1.10E-151 | positive |
| ATRX | AC009716.1 | 0.571267074 | 2.08E-97 | positive |
| RNF31 | AC009716.1 | 0.439893023 | 7.10E-54 | positive |
| MAP3K7 | Z97989.1 | 0.439418997 | 9.46E-54 | positive |
| DIABLO | Z97989.1 | 0.413999756 | 2.52E-47 | positive |
| BRAF | Z97989.1 | 0.46043748 | 1.71E-59 | positive |
| BACH2 | Z97989.1 | 0.534831379 | 2.31E-83 | positive |
| DIABLO | AC126118.1 | 0.465945379 | 4.59E-61 | positive |
| BRAF | AC126118.1 | 0.432565297 | 5.80E-52 | positive |
| DIABLO | AC023355.1 | 0.526047141 | 3.16E-80 | positive |
| BRAF | AC023355.1 | 0.45958318 | 2.99E-59 | positive |
| RNF31 | AC023355.1 | 0.423470564 | 1.18E-49 | positive |
| KLF9 | AC087521.1 | 0.501873129 | 4.67E-72 | positive |
| OTULIN | AC020978.7 | 0.45887851 | 4.72E-59 | positive |
| CYLD | AC020978.7 | 0.481528055 | 1.13E-65 | positive |
| DIABLO | AC020978.7 | 0.776779462 | 2.41E-225 | positive |
| CFLAR | AC020978.7 | 0.441486957 | 2.68E-54 | positive |
| BRAF | AC020978.7 | 0.672368096 | 2.56E-147 | positive |
| ATRX | AC020978.7 | 0.533153211 | 9.34E-83 | positive |
| RNF31 | AC020978.7 | 0.49627932 | 2.94E-70 | positive |
| DIABLO | AC037459.4 | 0.596699568 | 2.63E-108 | positive |
| BRAF | AC037459.4 | 0.492678778 | 4.06E-69 | positive |
| MAPK8 | AC107032.3 | 0.417139531 | 4.34E-48 | positive |
| OTULIN | AC107032.3 | 0.408260161 | 6.00E-46 | positive |
| CYLD | AC107032.3 | 0.44821948 | 4.18E-56 | positive |
| DIABLO | AC107032.3 | 0.660807029 | 1.29E-140 | positive |
| BRAF | AC107032.3 | 0.571886134 | 1.16E-97 | positive |
| ATRX | AC107032.3 | 0.49028875 | 2.28E-68 | positive |
| BCL2 | AC119428.2 | 0.405701342 | 2.42E-45 | positive |
| DIABLO | AC058791.1 | 0.790345123 | 1.21E-238 | positive |
| BRAF | AC058791.1 | 0.664171376 | 1.55E-142 | positive |
| DIABLO | AL161663.2 | 0.423596263 | 1.10E-49 | positive |
| TSC1 | NDUFA6-DT | 0.48286461 | 4.45E-66 | positive |
| BRAF | NDUFA6-DT | 0.438919115 | 1.28E-53 | positive |
| ATRX | NDUFA6-DT | 0.481213215 | 1.41E-65 | positive |
| TSC1 | SMG7-AS1 | 0.45487771 | 6.20E-58 | positive |
| BRAF | SMG7-AS1 | 0.431897247 | 8.61E-52 | positive |
| ATRX | SMG7-AS1 | 0.409020283 | 3.96E-46 | positive |
| BCL2L11 | SMG7-AS1 | 0.418994378 | 1.52E-48 | positive |
| ATRX | AL121890.4 | 0.402468307 | 1.38E-44 | positive |
| DIABLO | AL136526.1 | 0.694425447 | 5.51E-161 | positive |
| BRAF | AL136526.1 | 0.548163174 | 2.66E-88 | positive |
| OTULIN | AP001429.1 | 0.454730988 | 6.81E-58 | positive |
| CYLD | AP001429.1 | 0.416118819 | 7.71E-48 | positive |
| DIABLO | AP001429.1 | 0.753348208 | 1.88E-204 | positive |
| BRAF | AP001429.1 | 0.680178058 | 5.10E-152 | positive |
| DIABLO | LINC02803 | 0.523959761 | 1.70E-79 | positive |
| BRAF | LINC02803 | 0.4296389 | 3.26E-51 | positive |
| ATRX | LINC02803 | 0.403024318 | 1.02E-44 | positive |
| RNF31 | LINC02803 | 0.401077889 | 2.90E-44 | positive |
| TSC1 | AC245060.7 | 0.437045606 | 3.98E-53 | positive |
| ATRX | AC245060.7 | 0.445789668 | 1.90E-55 | positive |
| OTULIN | AC092279.1 | 0.400787915 | 3.39E-44 | positive |
| CYLD | AC092279.1 | 0.418868189 | 1.63E-48 | positive |
| DIABLO | AC092279.1 | 0.674388741 | 1.61E-148 | positive |
| CFLAR | AC092279.1 | 0.479625007 | 4.26E-65 | positive |
| BRAF | AC092279.1 | 0.650399724 | 7.77E-135 | positive |
| ATRX | AC092279.1 | 0.616663831 | 1.43E-117 | positive |
| MAPK8 | AL359094.1 | 0.412723361 | 5.13E-47 | positive |
| OTULIN | AL359094.1 | 0.446472485 | 1.24E-55 | positive |
| CYLD | AL359094.1 | 0.440724405 | 4.28E-54 | positive |
| DIABLO | AL359094.1 | 0.806660162 | 5.23E-256 | positive |
| CFLAR | AL359094.1 | 0.432073162 | 7.76E-52 | positive |
| BRAF | AL359094.1 | 0.700091227 | 1.07E-164 | positive |
| ATRX | AL359094.1 | 0.49134865 | 1.06E-68 | positive |
| OTULIN | AC009318.2 | 0.40952966 | 2.99E-46 | positive |
| DIABLO | AC009318.2 | 0.564028172 | 1.77E-94 | positive |
| BRAF | AC009318.2 | 0.534692621 | 2.60E-83 | positive |
| TSC1 | AC137932.3 | 0.489189225 | 5.02E-68 | positive |
| OTULIN | AC137932.3 | 0.407830909 | 7.59E-46 | positive |
| DIABLO | AC137932.3 | 0.57882508 | 1.51E-100 | positive |
| BRAF | AC137932.3 | 0.486969563 | 2.45E-67 | positive |
| RNF31 | AC137932.3 | 0.475286125 | 8.48E-64 | positive |
| OTULIN | AC021092.1 | 0.418243272 | 2.33E-48 | positive |
| DIABLO | AC021092.1 | 0.636417761 | 2.03E-127 | positive |
| BRAF | AC021092.1 | 0.618996631 | 1.07E-118 | positive |
| ATRX | AC021092.1 | 0.462011005 | 6.13E-60 | positive |
| TSC1 | AL138921.1 | 0.460457027 | 1.69E-59 | positive |
| MAPK8 | AL138921.1 | 0.467994455 | 1.17E-61 | positive |
| IPMK | AL138921.1 | 0.437650061 | 2.76E-53 | positive |
| OTULIN | AL138921.1 | 0.523829444 | 1.89E-79 | positive |
| CYLD | AL138921.1 | 0.475811863 | 5.92E-64 | positive |
| MAP3K7 | AL138921.1 | 0.478620323 | 8.55E-65 | positive |
| DIABLO | AL138921.1 | 0.718768903 | 1.38E-177 | positive |
| CFLAR | AL138921.1 | 0.470022255 | 3.01E-62 | positive |
| BRAF | AL138921.1 | 0.674195527 | 2.10E-148 | positive |
| ATRX | AL138921.1 | 0.561456375 | 1.87E-93 | positive |
| RNF31 | AL138921.1 | 0.410816879 | 1.47E-46 | positive |
| OTULIN | AC074050.4 | 0.420126045 | 8.00E-49 | positive |
| DIABLO | AC074050.4 | 0.630894987 | 1.36E-124 | positive |
| BRAF | AC074050.4 | 0.502151015 | 3.79E-72 | positive |
| DIABLO | AC010999.2 | 0.482794861 | 4.67E-66 | positive |
| BRAF | AC010999.2 | 0.41503124 | 1.42E-47 | positive |
| DIABLO | LINC01210 | 0.447736375 | 5.65E-56 | positive |
| BRAF | LINC01210 | 0.402498697 | 1.36E-44 | positive |
| DIABLO | AP002340.1 | 0.446231411 | 1.44E-55 | positive |
| CFLAR | AP002340.1 | 0.446237529 | 1.44E-55 | positive |
| BRAF | AP002340.1 | 0.475576716 | 6.95E-64 | positive |
| ATRX | AP002340.1 | 0.45356291 | 1.43E-57 | positive |
| MAPK8 | LURAP1L-AS1 | 0.400026387 | 5.08E-44 | positive |
| OTULIN | LURAP1L-AS1 | 0.461305298 | 9.73E-60 | positive |
| CYLD | LURAP1L-AS1 | 0.418860901 | 1.64E-48 | positive |
| DIABLO | LURAP1L-AS1 | 0.7729358 | 9.55E-222 | positive |
| BRAF | LURAP1L-AS1 | 0.686609858 | 5.30E-156 | positive |
| ATRX | LURAP1L-AS1 | 0.401177069 | 2.75E-44 | positive |
| DIABLO | AC023830.1 | 0.646775481 | 7.08E-133 | positive |
| CFLAR | AC023830.1 | 0.441667972 | 2.40E-54 | positive |
| BRAF | AC023830.1 | 0.511409574 | 3.35E-75 | positive |
| ATRX | AC023830.1 | 0.495361963 | 5.75E-70 | positive |
| DIABLO | AC087620.1 | 0.55456146 | 9.42E-91 | positive |
| BRAF | AC087620.1 | 0.473763439 | 2.40E-63 | positive |
| OTULIN | MIR133A1HG | 0.464916709 | 9.07E-61 | positive |
| CYLD | MIR133A1HG | 0.453224185 | 1.78E-57 | positive |
| DIABLO | MIR133A1HG | 0.778895274 | 2.34E-227 | positive |
| BRAF | MIR133A1HG | 0.702291224 | 3.65E-166 | positive |
| ATRX | MIR133A1HG | 0.433279331 | 3.79E-52 | positive |
| OTULIN | AL358216.1 | 0.436984277 | 4.13E-53 | positive |
| CYLD | AL358216.1 | 0.404062537 | 5.86E-45 | positive |
| DIABLO | AL358216.1 | 0.755586686 | 2.40E-206 | positive |
| BRAF | AL358216.1 | 0.666811229 | 4.64E-144 | positive |
| TSC1 | AL360181.2 | 0.453682992 | 1.33E-57 | positive |
| RNF31 | AL360181.2 | 0.53926917 | 5.55E-85 | positive |
| ATRX | AC011444.1 | 0.403484397 | 8.00E-45 | positive |
| DIABLO | AC013549.1 | 0.507913468 | 4.89E-74 | positive |
| BRAF | AC013549.1 | 0.459148988 | 3.96E-59 | positive |
| ATRX | AC013549.1 | 0.516259059 | 7.71E-77 | positive |
| HDAC9 | LINC02542 | 0.560254196 | 5.60E-93 | positive |
| DIABLO | INTS9-AS1 | 0.700181176 | 9.30E-165 | positive |
| BRAF | INTS9-AS1 | 0.619098592 | 9.53E-119 | positive |
| ATRX | INTS9-AS1 | 0.411701116 | 9.04E-47 | positive |
| FASLG | AC116914.1 | 0.403680935 | 7.19E-45 | positive |
| EGFR | AC022509.1 | 0.416323735 | 6.87E-48 | positive |
| DIABLO | AC078942.1 | 0.451266326 | 6.15E-57 | positive |
| TSC1 | AL513327.2 | 0.474580558 | 1.37E-63 | positive |
| OTULIN | AL513327.2 | 0.460820175 | 1.34E-59 | positive |
| CYLD | AL513327.2 | 0.415560396 | 1.05E-47 | positive |
| DIABLO | AL513327.2 | 0.694321554 | 6.44E-161 | positive |
| CFLAR | AL513327.2 | 0.440474173 | 4.98E-54 | positive |
| BRAF | AL513327.2 | 0.635490065 | 6.11E-127 | positive |
| ATRX | AL513327.2 | 0.506178405 | 1.83E-73 | positive |
| DIABLO | SPON1-AS1 | 0.616959112 | 1.03E-117 | positive |
| BRAF | SPON1-AS1 | 0.568653524 | 2.42E-96 | positive |
| DIABLO | LINC02062 | 0.413922583 | 2.63E-47 | positive |
| TRAF2 | LINC02245 | 0.412479579 | 5.88E-47 | positive |
| CD40 | LINC02245 | 0.47537813 | 7.96E-64 | positive |
| MAPK8 | AL139274.2 | 0.402262027 | 1.54E-44 | positive |
| CYLD | AL139274.2 | 0.471821935 | 8.95E-63 | positive |
| MAP3K7 | AL139274.2 | 0.450793897 | 8.29E-57 | positive |
| DIABLO | AL139274.2 | 0.665101807 | 4.52E-143 | positive |
| CFLAR | AL139274.2 | 0.430440284 | 2.04E-51 | positive |
| BRAF | AL139274.2 | 0.660843367 | 1.23E-140 | positive |
| ATRX | AL139274.2 | 0.576396629 | 1.57E-99 | positive |
| TSC1 | AC138956.1 | 0.474144413 | 1.85E-63 | positive |
| MAPK8 | AC138956.1 | 0.420463883 | 6.60E-49 | positive |
| OTULIN | AC138956.1 | 0.449432015 | 1.95E-56 | positive |
| CYLD | AC138956.1 | 0.466524036 | 3.12E-61 | positive |
| DIABLO | AC138956.1 | 0.707680885 | 8.21E-170 | positive |
| CFLAR | AC138956.1 | 0.471630036 | 1.02E-62 | positive |
| BRAF | AC138956.1 | 0.682300727 | 2.54E-153 | positive |
| ATRX | AC138956.1 | 0.510526672 | 6.61E-75 | positive |
| RNF31 | AC138956.1 | 0.459216884 | 3.79E-59 | positive |
| DIABLO | AC024451.4 | 0.624258719 | 2.84E-121 | positive |
| BRAF | AC024451.4 | 0.520601386 | 2.50E-78 | positive |
| ATRX | AC024451.4 | 0.527431224 | 1.03E-80 | positive |
| MAPK8 | AC016949.1 | 0.40654178 | 1.53E-45 | positive |
| IPMK | AC016949.1 | 0.426074733 | 2.62E-50 | positive |
| OTULIN | AC016949.1 | 0.461143304 | 1.08E-59 | positive |
| CYLD | AC016949.1 | 0.476180644 | 4.59E-64 | positive |
| DIABLO | AC016949.1 | 0.769973256 | 5.08E-219 | positive |
| CFLAR | AC016949.1 | 0.489964295 | 2.88E-68 | positive |
| BRAF | AC016949.1 | 0.717601505 | 9.47E-177 | positive |
| ATRX | AC016949.1 | 0.640980942 | 8.50E-130 | positive |
| RNF31 | AC016949.1 | 0.41181964 | 8.47E-47 | positive |
| OTULIN | ITPRIP-AS1 | 0.400388407 | 4.19E-44 | positive |
| CYLD | ITPRIP-AS1 | 0.410306891 | 1.95E-46 | positive |
| DIABLO | ITPRIP-AS1 | 0.575340586 | 4.33E-99 | positive |
| BRAF | ITPRIP-AS1 | 0.549423554 | 8.83E-89 | positive |
| DIABLO | AC090970.1 | 0.620248978 | 2.63E-119 | positive |
| BRAF | AC090970.1 | 0.566107171 | 2.60E-95 | positive |
| ATRX | AC090970.1 | 0.493740501 | 1.88E-69 | positive |
| CYLD | AC002558.3 | 0.464343102 | 1.32E-60 | positive |
| DIABLO | AC002558.3 | 0.743593667 | 1.95E-196 | positive |
| CFLAR | AC002558.3 | 0.468226021 | 1.00E-61 | positive |
| BRAF | AC002558.3 | 0.677880575 | 1.28E-150 | positive |
| ATRX | AC002558.3 | 0.582574489 | 3.90E-102 | positive |
| ATRX | LINC00649 | 0.435454722 | 1.03E-52 | positive |
| DIABLO | AP001439.1 | 0.695194328 | 1.75E-161 | positive |
| BRAF | AP001439.1 | 0.540111439 | 2.72E-85 | positive |
| DIABLO | AL356740.2 | 0.523284115 | 2.93E-79 | positive |
| BRAF | AL356740.2 | 0.469310578 | 4.86E-62 | positive |
| OTULIN | AP003168.2 | 0.448646518 | 3.20E-56 | positive |
| CYLD | AP003168.2 | 0.439199838 | 1.08E-53 | positive |
| DIABLO | AP003168.2 | 0.790433292 | 9.86E-239 | positive |
| BRAF | AP003168.2 | 0.687783813 | 9.67E-157 | positive |
| ATRX | AP003168.2 | 0.411053525 | 1.29E-46 | positive |
| DIABLO | AL133330.1 | 0.525077199 | 6.93E-80 | positive |
| BRAF | AL133330.1 | 0.461268535 | 9.96E-60 | positive |
| DIABLO | AC010184.1 | 0.477851944 | 1.45E-64 | positive |
| BRAF | AC010184.1 | 0.437617068 | 2.82E-53 | positive |
| MAPK8 | AL162171.3 | 0.416055468 | 7.99E-48 | positive |
| OTULIN | AL162171.3 | 0.479871673 | 3.59E-65 | positive |
| CYLD | AL162171.3 | 0.456775146 | 1.83E-58 | positive |
| DIABLO | AL162171.3 | 0.801274236 | 4.26E-250 | positive |
| CFLAR | AL162171.3 | 0.42156039 | 3.53E-49 | positive |
| BRAF | AL162171.3 | 0.719825962 | 2.39E-178 | positive |
| ATRX | AL162171.3 | 0.488201324 | 1.02E-67 | positive |
| OTULIN | NFIA-AS1 | 0.450367923 | 1.08E-56 | positive |
| CYLD | NFIA-AS1 | 0.402573822 | 1.30E-44 | positive |
| DIABLO | NFIA-AS1 | 0.790468346 | 9.08E-239 | positive |
| BRAF | NFIA-AS1 | 0.663392038 | 4.33E-142 | positive |
| OTULIN | AC034229.4 | 0.433436815 | 3.45E-52 | positive |
| DIABLO | AC034229.4 | 0.559132167 | 1.55E-92 | positive |
| BRAF | AC034229.4 | 0.525519884 | 4.84E-80 | positive |
| DIABLO | AC131025.3 | 0.47793872 | 1.37E-64 | positive |
| CFLAR | AC131025.3 | 0.418271761 | 2.29E-48 | positive |
| BRAF | AC131025.3 | 0.407670038 | 8.28E-46 | positive |
| ATRX | AC131025.3 | 0.409713662 | 2.70E-46 | positive |
| RNF31 | AC131025.3 | 0.432406921 | 6.37E-52 | positive |
| DIABLO | AP001619.1 | 0.48164685 | 1.04E-65 | positive |
| TSC1 | AC022098.1 | 0.443293185 | 8.87E-55 | positive |
| DIABLO | AC022098.1 | 0.541891567 | 5.97E-86 | positive |
| BRAF | AC022098.1 | 0.466961096 | 2.34E-61 | positive |
| OTULIN | MSC-AS1 | 0.470623779 | 2.01E-62 | positive |
| CYLD | MSC-AS1 | 0.523809409 | 1.92E-79 | positive |
| MAP3K7 | MSC-AS1 | 0.417046965 | 4.57E-48 | positive |
| DIABLO | MSC-AS1 | 0.479613846 | 4.30E-65 | positive |
| BRAF | MSC-AS1 | 0.543999201 | 9.80E-87 | positive |
| ATRX | MSC-AS1 | 0.420576969 | 6.19E-49 | positive |
| MAPK8 | AC022973.5 | 0.412433741 | 6.03E-47 | positive |
| IPMK | AC022973.5 | 0.42575973 | 3.14E-50 | positive |
| OTULIN | AC022973.5 | 0.510503529 | 6.73E-75 | positive |
| CYLD | AC022973.5 | 0.484052786 | 1.93E-66 | positive |
| MAP3K7 | AC022973.5 | 0.417805573 | 2.98E-48 | positive |
| DIABLO | AC022973.5 | 0.678098271 | 9.41E-151 | positive |
| CFLAR | AC022973.5 | 0.428397367 | 6.76E-51 | positive |
| BRAF | AC022973.5 | 0.658445094 | 2.76E-139 | positive |
| ATRX | AC022973.5 | 0.44834552 | 3.86E-56 | positive |
| DIABLO | AL160286.3 | 0.432778781 | 5.11E-52 | positive |
| MAPK8 | C2orf27A | 0.53614653 | 7.70E-84 | positive |
| IPMK | C2orf27A | 0.478507402 | 9.25E-65 | positive |
| OTULIN | C2orf27A | 0.504638303 | 5.86E-73 | positive |
| CYLD | C2orf27A | 0.504836861 | 5.04E-73 | positive |
| MAP3K7 | C2orf27A | 0.526775344 | 1.75E-80 | positive |
| DIABLO | C2orf27A | 0.608685284 | 8.65E-114 | positive |
| CFLAR | C2orf27A | 0.455669506 | 3.73E-58 | positive |
| BRAF | C2orf27A | 0.674571105 | 1.25E-148 | positive |
| ATRX | C2orf27A | 0.506775884 | 1.16E-73 | positive |
| MAPK8 | AC116158.3 | 0.407407922 | 9.55E-46 | positive |
| OTULIN | AC116158.3 | 0.473510626 | 2.85E-63 | positive |
| CYLD | AC116158.3 | 0.556867937 | 1.20E-91 | positive |
| DIABLO | AC116158.3 | 0.802595543 | 1.57E-251 | positive |
| CFLAR | AC116158.3 | 0.507867502 | 5.06E-74 | positive |
| BRAF | AC116158.3 | 0.722787383 | 1.69E-180 | positive |
| ATRX | AC116158.3 | 0.511381125 | 3.42E-75 | positive |
| OTULIN | AL160396.2 | 0.442032553 | 1.92E-54 | positive |
| MAP3K7 | AL160396.2 | 0.416509977 | 6.19E-48 | positive |
| DIABLO | AL160396.2 | 0.661339494 | 6.41E-141 | positive |
| BRAF | AL160396.2 | 0.606942739 | 5.61E-113 | positive |
| DIABLO | AC005329.3 | 0.52231302 | 6.38E-79 | positive |
| RNF31 | AC005329.3 | 0.431060915 | 1.41E-51 | positive |
| TRAF2 | SNHG7 | 0.496758834 | 2.07E-70 | positive |
| STUB1 | SNHG7 | 0.47336683 | 3.14E-63 | positive |
| MPG | SNHG7 | 0.505245475 | 3.70E-73 | positive |
| OTULIN | AC040169.3 | 0.439270555 | 1.04E-53 | positive |
| DIABLO | AC040169.3 | 0.686765754 | 4.23E-156 | positive |
| BRAF | AC040169.3 | 0.575975646 | 2.36E-99 | positive |
| DIABLO | AC011471.2 | 0.580785225 | 2.25E-101 | positive |
| BRAF | AC011471.2 | 0.440541559 | 4.78E-54 | positive |
| MYC | VPS9D1-AS1 | 0.527190314 | 1.25E-80 | positive |
| PLK1 | VPS9D1-AS1 | 0.41596733 | 8.39E-48 | positive |
| CYLD | AC011472.4 | 0.444332572 | 4.67E-55 | positive |
| DIABLO | AC011472.4 | 0.705627801 | 2.06E-168 | positive |
| CFLAR | AC011472.4 | 0.431935217 | 8.42E-52 | positive |
| BRAF | AC011472.4 | 0.668762867 | 3.39E-145 | positive |
| ATRX | AC011472.4 | 0.505891164 | 2.27E-73 | positive |
| CYLD | AC020913.1 | 0.497202082 | 1.49E-70 | positive |
| DIABLO | AC020913.1 | 0.548971847 | 1.31E-88 | positive |
| CFLAR | AC020913.1 | 0.535907747 | 9.41E-84 | positive |
| BRAF | AC020913.1 | 0.542997761 | 2.32E-86 | positive |
| ATRX | AC020913.1 | 0.619675614 | 5.00E-119 | positive |
| RNF31 | AC020913.1 | 0.502381145 | 3.19E-72 | positive |
| DIABLO | AC012100.2 | 0.574141245 | 1.36E-98 | positive |
| CFLAR | AC012100.2 | 0.402450506 | 1.39E-44 | positive |
| BRAF | AC012100.2 | 0.488153429 | 1.05E-67 | positive |
| ATRX | AC012100.2 | 0.510900463 | 4.96E-75 | positive |
| CYLD | AC020634.2 | 0.42648301 | 2.06E-50 | positive |
| DIABLO | AC020634.2 | 0.465334537 | 6.88E-61 | positive |
| BRAF | AC020634.2 | 0.509187878 | 1.85E-74 | positive |
| KLF9 | AC020634.2 | 0.414934745 | 1.50E-47 | positive |
| TSC1 | AC009090.6 | 0.433386382 | 3.56E-52 | positive |
| MAPK8 | AC009090.6 | 0.467468019 | 1.67E-61 | positive |
| IPMK | AC009090.6 | 0.444663983 | 3.81E-55 | positive |
| OTULIN | AC009090.6 | 0.512118167 | 1.94E-75 | positive |
| CYLD | AC009090.6 | 0.477251006 | 2.20E-64 | positive |
| MAP3K7 | AC009090.6 | 0.438486395 | 1.67E-53 | positive |
| DIABLO | AC009090.6 | 0.774023639 | 9.31E-223 | positive |
| CFLAR | AC009090.6 | 0.442045235 | 1.91E-54 | positive |
| BRAF | AC009090.6 | 0.717088533 | 2.20E-176 | positive |
| ATRX | AC009090.6 | 0.475342718 | 8.16E-64 | positive |
| DIABLO | AL359844.1 | 0.401482839 | 2.34E-44 | positive |
| KLF9 | AL049796.1 | 0.444312364 | 4.73E-55 | positive |
| CYLD | AC005695.2 | 0.442491303 | 1.45E-54 | positive |
| DIABLO | AC005695.2 | 0.756194548 | 7.29E-207 | positive |
| CFLAR | AC005695.2 | 0.42730451 | 1.28E-50 | positive |
| BRAF | AC005695.2 | 0.67813326 | 8.96E-151 | positive |
| ATRX | AC005695.2 | 0.499293081 | 3.19E-71 | positive |
| CYLD | AC005264.1 | 0.439001383 | 1.22E-53 | positive |
| DIABLO | AC005264.1 | 0.530995424 | 5.56E-82 | positive |
| RNF31 | AC005264.1 | 0.481139437 | 1.49E-65 | positive |
| DIABLO | AL162724.2 | 0.664287167 | 1.33E-142 | positive |
| CFLAR | AL162724.2 | 0.405463359 | 2.75E-45 | positive |
| BRAF | AL162724.2 | 0.580463151 | 3.08E-101 | positive |
| DIABLO | PRNCR1 | 0.552775509 | 4.61E-90 | positive |
| BRAF | PRNCR1 | 0.498149592 | 7.42E-71 | positive |
| DIABLO | AC053527.2 | 0.434871315 | 1.47E-52 | positive |
| BRAF | AC053527.2 | 0.489843289 | 3.14E-68 | positive |
| ATRX | AC053527.2 | 0.430820298 | 1.63E-51 | positive |
| DIABLO | AC144548.1 | 0.444344117 | 4.64E-55 | positive |
| BRAF | AC144548.1 | 0.439301531 | 1.02E-53 | positive |
| RNF31 | AC144548.1 | 0.406619672 | 1.47E-45 | positive |
| DIABLO | PLCXD2-AS1 | 0.658502404 | 2.56E-139 | positive |
| BRAF | PLCXD2-AS1 | 0.57117139 | 2.28E-97 | positive |
| DIABLO | AC018648.1 | 0.525151737 | 6.52E-80 | positive |
| BRAF | AC018648.1 | 0.497877128 | 9.07E-71 | positive |
| ATRX | AC018648.1 | 0.413527005 | 3.28E-47 | positive |
| MAPK8 | C3orf35 | 0.431958816 | 8.30E-52 | positive |
| DIABLO | C3orf35 | 0.790076966 | 2.27E-238 | positive |
| CFLAR | C3orf35 | 0.420724124 | 5.69E-49 | positive |
| BRAF | C3orf35 | 0.684283697 | 1.50E-154 | positive |
| ATRX | C3orf35 | 0.463338259 | 2.57E-60 | positive |
| DIABLO | AC017071.1 | 0.669924579 | 7.08E-146 | positive |
| BRAF | AC017071.1 | 0.611908285 | 2.64E-115 | positive |
| CYLD | Z93930.3 | 0.407981329 | 6.99E-46 | positive |
| DIABLO | Z93930.3 | 0.643747066 | 2.94E-131 | positive |
| CFLAR | Z93930.3 | 0.431194693 | 1.30E-51 | positive |
| BRAF | Z93930.3 | 0.569686536 | 9.21E-97 | positive |
| ATRX | Z93930.3 | 0.505194373 | 3.85E-73 | positive |
| DIABLO | AC138123.2 | 0.498407076 | 6.13E-71 | positive |
| BRAF | AC138123.2 | 0.442846977 | 1.17E-54 | positive |
| IPMK | CLIP1-AS1 | 0.417168658 | 4.27E-48 | positive |
| OTULIN | CLIP1-AS1 | 0.470271413 | 2.55E-62 | positive |
| CYLD | CLIP1-AS1 | 0.499206105 | 3.40E-71 | positive |
| DIABLO | CLIP1-AS1 | 0.845505453 | 5.25E-305 | positive |
| CFLAR | CLIP1-AS1 | 0.445301306 | 2.57E-55 | positive |
| BRAF | CLIP1-AS1 | 0.707345169 | 1.39E-169 | positive |
| ATRX | CLIP1-AS1 | 0.51107605 | 4.33E-75 | positive |
| OTULIN | AC002542.6 | 0.420520998 | 6.39E-49 | positive |
| DIABLO | AC002542.6 | 0.720798793 | 4.74E-179 | positive |
| BRAF | AC002542.6 | 0.617483654 | 5.76E-118 | positive |
| ATRX | AC002542.6 | 0.424578095 | 6.23E-50 | positive |
| TSC1 | GARS1-DT | 0.41620673 | 7.34E-48 | positive |
| MAPK8 | GARS1-DT | 0.402568923 | 1.31E-44 | positive |
| OTULIN | GARS1-DT | 0.414114779 | 2.37E-47 | positive |
| CYLD | GARS1-DT | 0.429909997 | 2.78E-51 | positive |
| MAP3K7 | GARS1-DT | 0.417750594 | 3.08E-48 | positive |
| DIABLO | GARS1-DT | 0.669597292 | 1.10E-145 | positive |
| CFLAR | GARS1-DT | 0.491853786 | 7.37E-69 | positive |
| BRAF | GARS1-DT | 0.638558215 | 1.57E-128 | positive |
| ATRX | GARS1-DT | 0.526959468 | 1.51E-80 | positive |
| DIABLO | AC005828.2 | 0.603750929 | 1.67E-111 | positive |
| BRAF | AC005828.2 | 0.565841963 | 3.32E-95 | positive |
| DIABLO | AC011676.1 | 0.514766495 | 2.48E-76 | positive |
| BRAF | AC011676.1 | 0.4143707 | 2.05E-47 | positive |
| OTULIN | MMADHC-DT | 0.413092414 | 4.18E-47 | positive |
| DIABLO | MMADHC-DT | 0.644742812 | 8.67E-132 | positive |
| BRAF | MMADHC-DT | 0.587857223 | 2.08E-104 | positive |
| DIABLO | AC092535.4 | 0.547408062 | 5.13E-88 | positive |
| BRAF | AC092535.4 | 0.550613882 | 3.11E-89 | positive |
| OTULIN | AC135178.4 | 0.412525904 | 5.73E-47 | positive |
| DIABLO | AC135178.4 | 0.747210488 | 2.29E-199 | positive |
| BRAF | AC135178.4 | 0.585132295 | 3.13E-103 | positive |
| DIABLO | AL157371.2 | 0.40005045 | 5.02E-44 | positive |
| CYLD | AC005899.8 | 0.452678563 | 2.52E-57 | positive |
| DIABLO | AC005899.8 | 0.699372357 | 3.19E-164 | positive |
| CFLAR | AC005899.8 | 0.458845108 | 4.82E-59 | positive |
| BRAF | AC005899.8 | 0.614765762 | 1.16E-116 | positive |
| ATRX | AC005899.8 | 0.566239384 | 2.30E-95 | positive |
| OTULIN | AL109923.1 | 0.465322765 | 6.93E-61 | positive |
| CYLD | AL109923.1 | 0.426168136 | 2.48E-50 | positive |
| DIABLO | AL109923.1 | 0.752057636 | 2.27E-203 | positive |
| CFLAR | AL109923.1 | 0.40390944 | 6.36E-45 | positive |
| BRAF | AL109923.1 | 0.682567571 | 1.74E-153 | positive |
| ATRX | AL109923.1 | 0.447741569 | 5.63E-56 | positive |
| DIABLO | AC244517.7 | 0.481127022 | 1.50E-65 | positive |
| BRAF | AC244517.7 | 0.482561814 | 5.50E-66 | positive |
| MAPK8 | SEMA6A-AS1 | 0.422045961 | 2.67E-49 | positive |
| OTULIN | SEMA6A-AS1 | 0.436917558 | 4.30E-53 | positive |
| DIABLO | SEMA6A-AS1 | 0.749618356 | 2.41E-201 | positive |
| BRAF | SEMA6A-AS1 | 0.649970347 | 1.33E-134 | positive |
| ATRX | SEMA6A-AS1 | 0.409835002 | 2.53E-46 | positive |
| DIABLO | AL355297.3 | 0.48525428 | 8.26E-67 | positive |
| BRAF | AL355297.3 | 0.443434216 | 8.13E-55 | positive |
| TSC1 | AC018766.1 | 0.412862495 | 4.75E-47 | positive |
| DIABLO | AC018766.1 | 0.656404695 | 3.82E-138 | positive |
| BRAF | AC018766.1 | 0.561219718 | 2.33E-93 | positive |
| ATRX | AC018766.1 | 0.4290218 | 4.69E-51 | positive |
| TSC1 | AP002812.5 | 0.400084084 | 4.93E-44 | positive |
| CYLD | AP002812.5 | 0.402152471 | 1.63E-44 | positive |
| DIABLO | AP002812.5 | 0.698184098 | 1.94E-163 | positive |
| CFLAR | AP002812.5 | 0.484838008 | 1.11E-66 | positive |
| BRAF | AP002812.5 | 0.66525259 | 3.70E-143 | positive |
| ATRX | AP002812.5 | 0.597074296 | 1.79E-108 | positive |
| RNF31 | AP002812.5 | 0.425211664 | 4.32E-50 | positive |
| BACH2 | AC145098.1 | 0.401508092 | 2.31E-44 | positive |
| HDAC9 | AC145098.1 | 0.492122046 | 6.07E-69 | positive |
| TSC1 | AC022382.2 | 0.493023958 | 3.16E-69 | positive |
| MAPK8 | AC022382.2 | 0.433378842 | 3.57E-52 | positive |
| IPMK | AC022382.2 | 0.406372638 | 1.68E-45 | positive |
| OTULIN | AC022382.2 | 0.439289911 | 1.02E-53 | positive |
| CYLD | AC022382.2 | 0.513163523 | 8.62E-76 | positive |
| DIABLO | AC022382.2 | 0.75464665 | 1.51E-205 | positive |
| CFLAR | AC022382.2 | 0.541820781 | 6.34E-86 | positive |
| BRAF | AC022382.2 | 0.714632586 | 1.21E-174 | positive |
| ATRX | AC022382.2 | 0.511538594 | 3.03E-75 | positive |
| OTULIN | AC023483.1 | 0.403835168 | 6.62E-45 | positive |
| DIABLO | AC023483.1 | 0.751520084 | 6.37E-203 | positive |
| BRAF | AC023483.1 | 0.661818038 | 3.43E-141 | positive |
| ATRX | AC023483.1 | 0.432204397 | 7.18E-52 | positive |
| OTULIN | AP001992.2 | 0.433876732 | 2.66E-52 | positive |
| CYLD | AP001992.2 | 0.410551497 | 1.71E-46 | positive |
| DIABLO | AP001992.2 | 0.682781733 | 1.28E-153 | positive |
| CFLAR | AP001992.2 | 0.428226224 | 7.47E-51 | positive |
| BRAF | AP001992.2 | 0.632152845 | 3.12E-125 | positive |
| ATRX | AP001992.2 | 0.494546985 | 1.04E-69 | positive |
| IPMK | AC011346.1 | 0.416956765 | 4.81E-48 | positive |
| OTULIN | AC011346.1 | 0.444267278 | 4.86E-55 | positive |
| CYLD | AC011346.1 | 0.480320522 | 2.63E-65 | positive |
| DIABLO | AC011346.1 | 0.599838305 | 1.01E-109 | positive |
| BRAF | AC011346.1 | 0.612397076 | 1.55E-115 | positive |
| ATRX | AC011346.1 | 0.526955888 | 1.51E-80 | positive |
| OTULIN | DTD1-AS1 | 0.407778541 | 7.81E-46 | positive |
| DIABLO | DTD1-AS1 | 0.729846184 | 9.74E-186 | positive |
| BRAF | DTD1-AS1 | 0.618862571 | 1.24E-118 | positive |
| CD40 | LINC01871 | 0.67891528 | 3.00E-151 | positive |
| CYLD | AC025171.3 | 0.453922537 | 1.14E-57 | positive |
| DIABLO | AC025171.3 | 0.470802714 | 1.78E-62 | positive |
| CFLAR | AC025171.3 | 0.491952055 | 6.87E-69 | positive |
| BRAF | AC025171.3 | 0.502660914 | 2.59E-72 | positive |
| ATRX | AC025171.3 | 0.50469795 | 5.60E-73 | positive |
| OTULIN | ZNF346-IT1 | 0.43216789 | 7.34E-52 | positive |
| CYLD | ZNF346-IT1 | 0.464696952 | 1.05E-60 | positive |
| DIABLO | ZNF346-IT1 | 0.777259976 | 8.45E-226 | positive |
| CFLAR | ZNF346-IT1 | 0.473525838 | 2.82E-63 | positive |
| BRAF | ZNF346-IT1 | 0.701111691 | 2.24E-165 | positive |
| ATRX | ZNF346-IT1 | 0.55131864 | 1.67E-89 | positive |
| MYC | UBR5-AS1 | 0.481414229 | 1.23E-65 | positive |
| DIABLO | AC078852.1 | 0.685429683 | 2.90E-155 | positive |
| BRAF | AC078852.1 | 0.51903992 | 8.63E-78 | positive |
| IPMK | AC022150.4 | 0.405081494 | 3.38E-45 | positive |
| OTULIN | AC022150.4 | 0.410939016 | 1.38E-46 | positive |
| CYLD | AC022150.4 | 0.42774812 | 9.88E-51 | positive |
| DIABLO | AC022150.4 | 0.605492158 | 2.63E-112 | positive |
| CFLAR | AC022150.4 | 0.47089147 | 1.68E-62 | positive |
| BRAF | AC022150.4 | 0.593043178 | 1.11E-106 | positive |
| ATRX | AC022150.4 | 0.470587267 | 2.06E-62 | positive |
| CD40 | FAM167A-AS1 | 0.593121474 | 1.03E-106 | positive |
| OTULIN | AC008264.2 | 0.453433501 | 1.56E-57 | positive |
| DIABLO | AC008264.2 | 0.788029599 | 2.64E-236 | positive |
| BRAF | AC008264.2 | 0.681467704 | 8.26E-153 | positive |
| OTULIN | RUSC1-AS1 | 0.410719064 | 1.56E-46 | positive |
| DIABLO | RUSC1-AS1 | 0.560958162 | 2.95E-93 | positive |
| BRAF | RUSC1-AS1 | 0.444357666 | 4.60E-55 | positive |
| OTULIN | AC108102.1 | 0.418846883 | 1.65E-48 | positive |
| DIABLO | AC108102.1 | 0.774381107 | 4.32E-223 | positive |
| BRAF | AC108102.1 | 0.658164899 | 3.97E-139 | positive |
| CFLAR | AC008537.4 | 0.428104027 | 8.02E-51 | positive |
| ATRX | AC008537.4 | 0.43883756 | 1.35E-53 | positive |
| RNF31 | AC008537.4 | 0.465186061 | 7.59E-61 | positive |
| MAPK8 | AL049869.2 | 0.447616771 | 6.08E-56 | positive |
| IPMK | AL049869.2 | 0.431234865 | 1.27E-51 | positive |
| OTULIN | AL049869.2 | 0.496093797 | 3.37E-70 | positive |
| CYLD | AL049869.2 | 0.509138213 | 1.92E-74 | positive |
| MAP3K7 | AL049869.2 | 0.408213178 | 6.16E-46 | positive |
| DIABLO | AL049869.2 | 0.797271459 | 8.06E-246 | positive |
| CFLAR | AL049869.2 | 0.472908079 | 4.29E-63 | positive |
| BRAF | AL049869.2 | 0.7416412 | 7.10E-195 | positive |
| ATRX | AL049869.2 | 0.518917143 | 9.51E-78 | positive |
| MAPK8 | AC022272.1 | 0.402296907 | 1.51E-44 | positive |
| IPMK | AC022272.1 | 0.402292609 | 1.52E-44 | positive |
| OTULIN | AC022272.1 | 0.441842957 | 2.16E-54 | positive |
| CYLD | AC022272.1 | 0.401084983 | 2.89E-44 | positive |
| DIABLO | AC022272.1 | 0.709248767 | 6.88E-171 | positive |
| BRAF | AC022272.1 | 0.667655042 | 1.50E-144 | positive |
| ATRX | AC022272.1 | 0.433259991 | 3.84E-52 | positive |
| MYC | AC124798.1 | 0.413665252 | 3.04E-47 | positive |
| SPATA2 | AC124798.1 | 0.421281662 | 4.14E-49 | positive |
| TARDBP | AC124798.1 | 0.433575019 | 3.18E-52 | positive |
| CYLD | AC090515.5 | 0.45421509 | 9.46E-58 | positive |
| DIABLO | AC090515.5 | 0.703919145 | 2.95E-167 | positive |
| CFLAR | AC090515.5 | 0.474336315 | 1.62E-63 | positive |
| BRAF | AC090515.5 | 0.641716019 | 3.49E-130 | positive |
| ATRX | AC090515.5 | 0.52006214 | 3.84E-78 | positive |
| DIABLO | AC073140.2 | 0.410998308 | 1.33E-46 | positive |
| CYLD | BHLHE40-AS1 | 0.401125038 | 2.83E-44 | positive |
| OTULIN | AC055764.1 | 0.476380984 | 4.00E-64 | positive |
| CYLD | AC055764.1 | 0.529219981 | 2.39E-81 | positive |
| DIABLO | AC055764.1 | 0.700418533 | 6.47E-165 | positive |
| CFLAR | AC055764.1 | 0.482030789 | 7.97E-66 | positive |
| BRAF | AC055764.1 | 0.667464508 | 1.94E-144 | positive |
| ATRX | AC055764.1 | 0.586189162 | 1.10E-103 | positive |
| TSC1 | AL355574.1 | 0.564491706 | 1.16E-94 | positive |
| ATRX | AL355574.1 | 0.449224826 | 2.22E-56 | positive |
| TSC1 | AC133550.3 | 0.45225685 | 3.29E-57 | positive |
| DIABLO | AC133550.3 | 0.537363708 | 2.77E-84 | positive |
| BRAF | AC133550.3 | 0.491462093 | 9.78E-69 | positive |
| ATRX | AC133550.3 | 0.511208613 | 3.91E-75 | positive |
| RNF31 | AC133550.3 | 0.455460515 | 4.27E-58 | positive |
| MAPK8 | AC108058.1 | 0.405556023 | 2.61E-45 | positive |
| OTULIN | AC108058.1 | 0.410981583 | 1.35E-46 | positive |
| CYLD | AC108058.1 | 0.419400722 | 1.21E-48 | positive |
| DIABLO | AC108058.1 | 0.773692495 | 1.89E-222 | positive |
| CFLAR | AC108058.1 | 0.470818263 | 1.76E-62 | positive |
| BRAF | AC108058.1 | 0.667307572 | 2.39E-144 | positive |
| ATRX | AC108058.1 | 0.427262522 | 1.31E-50 | positive |
| OTULIN | AC004672.2 | 0.404389092 | 4.91E-45 | positive |
| DIABLO | AC004672.2 | 0.742347396 | 1.94E-195 | positive |
| BRAF | AC004672.2 | 0.642836762 | 8.92E-131 | positive |
| ATRX | AC004672.2 | 0.479548369 | 4.50E-65 | positive |
| DIABLO | AC002064.1 | 0.687478724 | 1.51E-156 | positive |
| CFLAR | AC002064.1 | 0.413396659 | 3.53E-47 | positive |
| BRAF | AC002064.1 | 0.614530651 | 1.50E-116 | positive |
| OTULIN | AC131571.1 | 0.454293218 | 9.00E-58 | positive |
| CYLD | AC131571.1 | 0.407331612 | 9.96E-46 | positive |
| DIABLO | AC131571.1 | 0.772318001 | 3.56E-221 | positive |
| BRAF | AC131571.1 | 0.671993159 | 4.27E-147 | positive |
| ATRX | AC131571.1 | 0.427133594 | 1.41E-50 | positive |
| OTULIN | AC106799.3 | 0.416751146 | 5.40E-48 | positive |
| MAPK8 | AP000813.1 | 0.436430355 | 5.76E-53 | positive |
| OTULIN | AP000813.1 | 0.422019281 | 2.71E-49 | positive |
| MAP3K7 | AP000813.1 | 0.449549511 | 1.81E-56 | positive |
| DIABLO | AP000813.1 | 0.502522767 | 2.87E-72 | positive |
| BRAF | AP000813.1 | 0.563798593 | 2.19E-94 | positive |
| ATRX | AP000813.1 | 0.588536354 | 1.06E-104 | positive |
| DIABLO | AC073314.1 | 0.582129704 | 6.04E-102 | positive |
| BRAF | AC073314.1 | 0.550050663 | 5.10E-89 | positive |
| CYLD | AC090948.3 | 0.478588976 | 8.74E-65 | positive |
| DIABLO | AC090948.3 | 0.636691511 | 1.47E-127 | positive |
| CFLAR | AC090948.3 | 0.472040332 | 7.72E-63 | positive |
| BRAF | AC090948.3 | 0.590637993 | 1.28E-105 | positive |
| DIABLO | AC027338.1 | 0.525375703 | 5.44E-80 | positive |
| BRAF | AC027338.1 | 0.483068042 | 3.85E-66 | positive |
| OTULIN | AC103703.1 | 0.424670585 | 5.90E-50 | positive |
| CYLD | AC103703.1 | 0.439820111 | 7.42E-54 | positive |
| DIABLO | AC103703.1 | 0.767159908 | 1.80E-216 | positive |
| CFLAR | AC103703.1 | 0.454544826 | 7.66E-58 | positive |
| BRAF | AC103703.1 | 0.688516642 | 3.33E-157 | positive |
| ATRX | AC103703.1 | 0.605866743 | 1.77E-112 | positive |
| OTULIN | AL136115.1 | 0.402837844 | 1.13E-44 | positive |
| CYLD | AL136115.1 | 0.421025221 | 4.79E-49 | positive |
| DIABLO | AL136115.1 | 0.779891852 | 2.60E-228 | positive |
| CFLAR | AL136115.1 | 0.457453739 | 1.18E-58 | positive |
| BRAF | AL136115.1 | 0.66938098 | 1.48E-145 | positive |
| ATRX | AL136115.1 | 0.443317371 | 8.73E-55 | positive |
| DIABLO | MESTIT1 | 0.77633811 | 6.29E-225 | positive |
| BRAF | MESTIT1 | 0.631232898 | 9.16E-125 | positive |
| TSC1 | KDM4A-AS1 | 0.506575542 | 1.35E-73 | positive |
| MAPK8 | KDM4A-AS1 | 0.439474309 | 9.15E-54 | positive |
| OTULIN | KDM4A-AS1 | 0.545372357 | 3.00E-87 | positive |
| CYLD | KDM4A-AS1 | 0.423965618 | 8.87E-50 | positive |
| MAP3K7 | KDM4A-AS1 | 0.417887668 | 2.85E-48 | positive |
| DIABLO | KDM4A-AS1 | 0.72354774 | 4.70E-181 | positive |
| BRAF | KDM4A-AS1 | 0.670084149 | 5.71E-146 | positive |
| ATRX | KDM4A-AS1 | 0.520989561 | 1.84E-78 | positive |
| RNF31 | KDM4A-AS1 | 0.462468529 | 4.54E-60 | positive |
| OTULIN | DOCK4-AS1 | 0.411834174 | 8.40E-47 | positive |
| DIABLO | DOCK4-AS1 | 0.678976398 | 2.76E-151 | positive |
| BRAF | DOCK4-AS1 | 0.614400799 | 1.73E-116 | positive |
| TSC1 | SEC62-AS1 | 0.41954484 | 1.11E-48 | positive |
| MAPK8 | SEC62-AS1 | 0.447885956 | 5.14E-56 | positive |
| IPMK | SEC62-AS1 | 0.413215483 | 3.90E-47 | positive |
| OTULIN | SEC62-AS1 | 0.456398793 | 2.34E-58 | positive |
| CYLD | SEC62-AS1 | 0.555152639 | 5.56E-91 | positive |
| MAP3K7 | SEC62-AS1 | 0.424838183 | 5.36E-50 | positive |
| DIABLO | SEC62-AS1 | 0.723988782 | 2.23E-181 | positive |
| CFLAR | SEC62-AS1 | 0.565511614 | 4.51E-95 | positive |
| BRAF | SEC62-AS1 | 0.714349218 | 1.92E-174 | positive |
| ATRX | SEC62-AS1 | 0.619699424 | 4.87E-119 | positive |
| RNF31 | SEC62-AS1 | 0.430677781 | 1.77E-51 | positive |
| CYLD | AC008149.1 | 0.428460546 | 6.51E-51 | positive |
| DIABLO | AC008149.1 | 0.65336149 | 1.85E-136 | positive |
| CFLAR | AC008149.1 | 0.404316964 | 5.11E-45 | positive |
| BRAF | AC008149.1 | 0.592798315 | 1.43E-106 | positive |
| ATRX | AC008149.1 | 0.434282392 | 2.09E-52 | positive |
| TSC1 | LINC02614 | 0.403320367 | 8.73E-45 | positive |
| OTULIN | LINC02614 | 0.454031844 | 1.06E-57 | positive |
| MAP3K7 | LINC02614 | 0.417203284 | 4.19E-48 | positive |
| DIABLO | LINC02614 | 0.714549181 | 1.39E-174 | positive |
| BRAF | LINC02614 | 0.63058357 | 1.95E-124 | positive |
| ATRX | LINC02614 | 0.432553442 | 5.84E-52 | positive |
| OTULIN | AL355304.1 | 0.458703448 | 5.28E-59 | positive |
| CYLD | AL355304.1 | 0.4945638 | 1.03E-69 | positive |
| MAP3K7 | AL355304.1 | 0.417468592 | 3.61E-48 | positive |
| DIABLO | AL355304.1 | 0.593099498 | 1.05E-106 | positive |
| CFLAR | AL355304.1 | 0.415377777 | 1.17E-47 | positive |
| BRAF | AL355304.1 | 0.540689521 | 1.66E-85 | positive |
| BACH2 | AL355304.1 | 0.412396074 | 6.15E-47 | positive |
| ATRX | AL355304.1 | 0.48212976 | 7.44E-66 | positive |
| OTULIN | AL158801.3 | 0.40620265 | 1.84E-45 | positive |
| DIABLO | AL158801.3 | 0.714401412 | 1.76E-174 | positive |
| BRAF | AL158801.3 | 0.603404931 | 2.41E-111 | positive |
| CYLD | RERG-IT1 | 0.436057376 | 7.21E-53 | positive |
| DIABLO | RERG-IT1 | 0.47730898 | 2.12E-64 | positive |
| CFLAR | RERG-IT1 | 0.444071564 | 5.49E-55 | positive |
| BRAF | RERG-IT1 | 0.486494197 | 3.43E-67 | positive |
| ATRX | RERG-IT1 | 0.468033472 | 1.14E-61 | positive |
| KLF9 | RERG-IT1 | 0.487364329 | 1.85E-67 | positive |
| OTULIN | Z82217.1 | 0.417298784 | 3.97E-48 | positive |
| CYLD | Z82217.1 | 0.411971361 | 7.79E-47 | positive |
| DIABLO | Z82217.1 | 0.752827828 | 5.13E-204 | positive |
| BRAF | Z82217.1 | 0.662365379 | 1.67E-141 | positive |
| FASLG | AC090912.2 | 0.450827822 | 8.11E-57 | positive |
| DIABLO | DPP9-AS1 | 0.442012136 | 1.95E-54 | positive |
| CFLAR | DPP9-AS1 | 0.489279209 | 4.70E-68 | positive |
| ATRX | DPP9-AS1 | 0.496580617 | 2.36E-70 | positive |
| RNF31 | DPP9-AS1 | 0.561020438 | 2.79E-93 | positive |
| DIABLO | AC074138.1 | 0.465834218 | 4.94E-61 | positive |
| CFLAR | AC074138.1 | 0.453677075 | 1.33E-57 | positive |
| BRAF | AC074138.1 | 0.518191285 | 1.69E-77 | positive |
| ATRX | AC074138.1 | 0.533510832 | 6.94E-83 | positive |
| RNF31 | AC074138.1 | 0.409105912 | 3.78E-46 | positive |
| DIABLO | AC024382.1 | 0.503220761 | 1.70E-72 | positive |
| BRAF | AC024382.1 | 0.438335376 | 1.83E-53 | positive |
| CYLD | LINC01798 | 0.401682704 | 2.10E-44 | positive |
| DIABLO | LINC01798 | 0.468959474 | 6.15E-62 | positive |
| BRAF | LINC01798 | 0.489875825 | 3.07E-68 | positive |
| ATRX | LINC01798 | 0.449481741 | 1.89E-56 | positive |
| KLF9 | LINC01798 | 0.449110116 | 2.39E-56 | positive |
| HDAC9 | AC007671.1 | 0.445628618 | 2.10E-55 | positive |
| DIABLO | AL355483.2 | 0.635033372 | 1.05E-126 | positive |
| BRAF | AL355483.2 | 0.44881738 | 2.87E-56 | positive |
| TSC1 | AC012443.2 | 0.435037399 | 1.33E-52 | positive |
| MAPK8 | AC012443.2 | 0.453386627 | 1.60E-57 | positive |
| OTULIN | AC012443.2 | 0.499946932 | 1.96E-71 | positive |
| CYLD | AC012443.2 | 0.454612388 | 7.34E-58 | positive |
| MAP3K7 | AC012443.2 | 0.425785834 | 3.10E-50 | positive |
| DIABLO | AC012443.2 | 0.756110108 | 8.61E-207 | positive |
| CFLAR | AC012443.2 | 0.466979112 | 2.31E-61 | positive |
| BRAF | AC012443.2 | 0.67194673 | 4.55E-147 | positive |
| ATRX | AC012443.2 | 0.453476639 | 1.51E-57 | positive |
| OTULIN | AC009041.3 | 0.424404645 | 6.89E-50 | positive |
| MAP3K7 | AC009041.3 | 0.436424541 | 5.78E-53 | positive |
| DIABLO | AC009041.3 | 0.413223098 | 3.89E-47 | positive |
| CYLD | AC232271.3 | 0.512626502 | 1.31E-75 | positive |
| DIABLO | AC232271.3 | 0.535326356 | 1.53E-83 | positive |
| BRAF | AC232271.3 | 0.521982816 | 8.31E-79 | positive |
| ATRX | AC232271.3 | 0.434015487 | 2.45E-52 | positive |
| CFLAR | AC093525.4 | 0.449621286 | 1.73E-56 | positive |
| BRAF | AC093525.4 | 0.419024203 | 1.50E-48 | positive |
| ATRX | AC093525.4 | 0.511879198 | 2.33E-75 | positive |
| OTULIN | GPC6-AS1 | 0.425273887 | 4.17E-50 | positive |
| CYLD | GPC6-AS1 | 0.408958 | 4.09E-46 | positive |
| DIABLO | GPC6-AS1 | 0.7084463 | 2.45E-170 | positive |
| BRAF | GPC6-AS1 | 0.654789635 | 3.02E-137 | positive |
| MAPK8 | AL391834.1 | 0.45220658 | 3.39E-57 | positive |
| IPMK | AL391834.1 | 0.426699542 | 1.82E-50 | positive |
| OTULIN | AL391834.1 | 0.463261576 | 2.70E-60 | positive |
| MAP3K7 | AL391834.1 | 0.419131584 | 1.41E-48 | positive |
| DIABLO | AL391834.1 | 0.679703715 | 9.93E-152 | positive |
| BRAF | AL391834.1 | 0.663511013 | 3.70E-142 | positive |
| ATRX | AL391834.1 | 0.443641204 | 7.16E-55 | positive |
| DIABLO | AC022405.1 | 0.53283311 | 1.22E-82 | positive |
| BRAF | AC022405.1 | 0.470904075 | 1.66E-62 | positive |
| ATRX | AC022405.1 | 0.479720981 | 3.99E-65 | positive |
| DIABLO | AC108159.1 | 0.652314961 | 6.97E-136 | positive |
| BRAF | AC108159.1 | 0.534853842 | 2.27E-83 | positive |
| OTULIN | AL139120.1 | 0.450562158 | 9.59E-57 | positive |
| CYLD | AL139120.1 | 0.414469799 | 1.94E-47 | positive |
| DIABLO | AL139120.1 | 0.756383409 | 5.03E-207 | positive |
| BRAF | AL139120.1 | 0.664822685 | 6.54E-143 | positive |
| ATRX | AL139120.1 | 0.43916149 | 1.11E-53 | positive |
| DIABLO | ERICH6-AS1 | 0.4103308 | 1.93E-46 | positive |
| ATRX | ERICH6-AS1 | 0.485790554 | 5.65E-67 | positive |
| BACH2 | AC006058.1 | 0.439534693 | 8.82E-54 | positive |
| ATRX | LIFR-AS1 | 0.455788262 | 3.46E-58 | positive |
| KLF9 | LIFR-AS1 | 0.400053872 | 5.01E-44 | positive |
| TSC1 | AC020915.2 | 0.489381804 | 4.37E-68 | positive |
| DIABLO | AC020915.2 | 0.548265462 | 2.43E-88 | positive |
| CFLAR | AC020915.2 | 0.437943965 | 2.31E-53 | positive |
| BRAF | AC020915.2 | 0.538794104 | 8.30E-85 | positive |
| ATRX | AC020915.2 | 0.486295046 | 3.95E-67 | positive |
| RNF31 | AC020915.2 | 0.441417638 | 2.80E-54 | positive |
| OTULIN | AC093388.1 | 0.44289509 | 1.13E-54 | positive |
| CYLD | AC093388.1 | 0.457809327 | 9.42E-59 | positive |
| MAP3K7 | AC093388.1 | 0.403184804 | 9.39E-45 | positive |
| DIABLO | AC093388.1 | 0.6291912 | 9.84E-124 | positive |
| CFLAR | AC093388.1 | 0.429653683 | 3.24E-51 | positive |
| BRAF | AC093388.1 | 0.603893069 | 1.44E-111 | positive |
| ATRX | AC093388.1 | 0.463044828 | 3.11E-60 | positive |
| TSC1 | BACE1-AS | 0.470383784 | 2.36E-62 | positive |
| KLF9 | AC006130.3 | 0.487643173 | 1.51E-67 | positive |
| OTULIN | AL137139.2 | 0.408345204 | 5.73E-46 | positive |
| DIABLO | AL137139.2 | 0.657544898 | 8.83E-139 | positive |
| BRAF | AL137139.2 | 0.551741066 | 1.15E-89 | positive |
| DIABLO | AL160163.1 | 0.54954049 | 7.97E-89 | positive |
| BRAF | AL160163.1 | 0.47679518 | 3.01E-64 | positive |
| ATRX | AL160163.1 | 0.442697089 | 1.28E-54 | positive |
| OTULIN | AC024896.1 | 0.403052572 | 1.01E-44 | positive |
| CYLD | AC024896.1 | 0.42324471 | 1.34E-49 | positive |
| DIABLO | AC024896.1 | 0.54657989 | 1.05E-87 | positive |
| CFLAR | AC024896.1 | 0.459284491 | 3.63E-59 | positive |
| BRAF | AC024896.1 | 0.533152607 | 9.34E-83 | positive |
| ATRX | AC024896.1 | 0.474450182 | 1.50E-63 | positive |
| DIABLO | AC004771.1 | 0.538020165 | 1.59E-84 | positive |
| BRAF | AC004771.1 | 0.448553345 | 3.39E-56 | positive |
| DIABLO | LINC01060 | 0.453428269 | 1.56E-57 | positive |
| MAPK8 | AC080162.1 | 0.421637318 | 3.38E-49 | positive |
| IPMK | AC080162.1 | 0.414833618 | 1.58E-47 | positive |
| OTULIN | AC080162.1 | 0.496754574 | 2.07E-70 | positive |
| CYLD | AC080162.1 | 0.534052341 | 4.43E-83 | positive |
| MAP3K7 | AC080162.1 | 0.400740178 | 3.48E-44 | positive |
| DIABLO | AC080162.1 | 0.844170971 | 4.18E-303 | positive |
| CFLAR | AC080162.1 | 0.515508774 | 1.39E-76 | positive |
| BRAF | AC080162.1 | 0.761239001 | 3.22E-211 | positive |
| ATRX | AC080162.1 | 0.573331161 | 2.94E-98 | positive |
| TSC1 | AC090198.1 | 0.436778913 | 4.67E-53 | positive |
| MAPK8 | AC090198.1 | 0.430208722 | 2.33E-51 | positive |
| OTULIN | AC090198.1 | 0.413109016 | 4.14E-47 | positive |
| MAP3K7 | AC090198.1 | 0.416893097 | 4.99E-48 | positive |
| DIABLO | AC090198.1 | 0.636874056 | 1.18E-127 | positive |
| CFLAR | AC090198.1 | 0.421574737 | 3.50E-49 | positive |
| BRAF | AC090198.1 | 0.633010116 | 1.14E-125 | positive |
| ATRX | AC090198.1 | 0.490523088 | 1.92E-68 | positive |
| IPMK | AL139353.2 | 0.401016408 | 3.00E-44 | positive |
| OTULIN | AL139353.2 | 0.506649525 | 1.28E-73 | positive |
| CYLD | AL139353.2 | 0.465337761 | 6.86E-61 | positive |
| MAP3K7 | AL139353.2 | 0.411975749 | 7.77E-47 | positive |
| DIABLO | AL139353.2 | 0.682779725 | 1.28E-153 | positive |
| BRAF | AL139353.2 | 0.672263133 | 2.96E-147 | positive |
| ATRX | AL139353.2 | 0.505783716 | 2.47E-73 | positive |
| DIABLO | AL161729.2 | 0.550144327 | 4.69E-89 | positive |
| BRAF | AL161729.2 | 0.477481014 | 1.88E-64 | positive |
| TSC1 | AL162727.2 | 0.414740856 | 1.67E-47 | positive |
| DIABLO | AL162727.2 | 0.427903081 | 9.02E-51 | positive |
| CYLD | AC005740.3 | 0.407915103 | 7.24E-46 | positive |
| DIABLO | AC005740.3 | 0.745971722 | 2.34E-198 | positive |
| BRAF | AC005740.3 | 0.652944597 | 3.14E-136 | positive |
| ATRX | AC005740.3 | 0.424551961 | 6.32E-50 | positive |
| OTULIN | AC083870.1 | 0.443874794 | 6.20E-55 | positive |
| CYLD | AC083870.1 | 0.418253202 | 2.32E-48 | positive |
| DIABLO | AC083870.1 | 0.703000086 | 1.22E-166 | positive |
| BRAF | AC083870.1 | 0.658019544 | 4.79E-139 | positive |
| CYLD | AC090617.4 | 0.519675333 | 5.22E-78 | positive |
| DIABLO | AC090617.4 | 0.678489451 | 5.45E-151 | positive |
| CFLAR | AC090617.4 | 0.536503666 | 5.71E-84 | positive |
| BRAF | AC090617.4 | 0.63746714 | 5.81E-128 | positive |
| ATRX | AC090617.4 | 0.619805426 | 4.33E-119 | positive |
| RNF31 | AC090617.4 | 0.464137561 | 1.52E-60 | positive |
| MAPK8 | AC068888.1 | 0.426248095 | 2.37E-50 | positive |
| DIABLO | AC068888.1 | 0.678436832 | 5.86E-151 | positive |
| BRAF | AC068888.1 | 0.581523338 | 1.09E-101 | positive |
| ATRX | AC068888.1 | 0.40726071 | 1.04E-45 | positive |
| DIABLO | LINC01585 | 0.574730484 | 7.76E-99 | positive |
| BRAF | LINC01585 | 0.466444881 | 3.29E-61 | positive |
| ATRX | LINC01585 | 0.419754905 | 9.88E-49 | positive |
| TSC1 | AC068790.7 | 0.477602059 | 1.73E-64 | positive |
| MAPK8 | AC068790.7 | 0.425358407 | 3.97E-50 | positive |
| OTULIN | AC068790.7 | 0.439032461 | 1.20E-53 | positive |
| CYLD | AC068790.7 | 0.466136701 | 4.04E-61 | positive |
| MAP3K7 | AC068790.7 | 0.41267759 | 5.26E-47 | positive |
| DIABLO | AC068790.7 | 0.746924648 | 3.93E-199 | positive |
| CFLAR | AC068790.7 | 0.504561489 | 6.21E-73 | positive |
| BRAF | AC068790.7 | 0.683434933 | 5.05E-154 | positive |
| ATRX | AC068790.7 | 0.596522778 | 3.15E-108 | positive |
| OTULIN | NEAT1 | 0.416367714 | 6.70E-48 | positive |
| DIABLO | NEAT1 | 0.709018568 | 9.91E-171 | positive |
| BRAF | NEAT1 | 0.603605865 | 1.95E-111 | positive |
| TSC1 | AC109347.1 | 0.407417607 | 9.50E-46 | positive |
| OTULIN | AC109347.1 | 0.40302399 | 1.02E-44 | positive |
| CYLD | AC109347.1 | 0.454635039 | 7.24E-58 | positive |
| MAP3K7 | AC109347.1 | 0.467940982 | 1.22E-61 | positive |
| DIABLO | AC109347.1 | 0.461451065 | 8.84E-60 | positive |
| BRAF | AC109347.1 | 0.523971536 | 1.69E-79 | positive |
| ATRX | AC109347.1 | 0.424802306 | 5.47E-50 | positive |
| MAPK8 | AC093752.2 | 0.412009871 | 7.62E-47 | positive |
| OTULIN | AC093752.2 | 0.404897431 | 3.73E-45 | positive |
| CYLD | AC093752.2 | 0.427149243 | 1.40E-50 | positive |
| DIABLO | AC093752.2 | 0.736453623 | 8.52E-191 | positive |
| CFLAR | AC093752.2 | 0.418973584 | 1.54E-48 | positive |
| BRAF | AC093752.2 | 0.667529293 | 1.78E-144 | positive |
| ATRX | AC093752.2 | 0.446052057 | 1.61E-55 | positive |
| IPMK | AC072039.2 | 0.400179919 | 4.68E-44 | positive |
| OTULIN | AC072039.2 | 0.418357665 | 2.18E-48 | positive |
| DIABLO | AC072039.2 | 0.707401007 | 1.28E-169 | positive |
| BRAF | AC072039.2 | 0.613953356 | 2.83E-116 | positive |
| ATRX | AC072039.2 | 0.459746913 | 2.69E-59 | positive |
| STUB1 | AC009061.1 | 0.518802671 | 1.04E-77 | positive |
| TSC1 | AL031282.2 | 0.501559564 | 5.90E-72 | positive |
| ATRX | AL031282.2 | 0.506132504 | 1.89E-73 | positive |
| RNF31 | AL031282.2 | 0.413116889 | 4.12E-47 | positive |
| DIABLO | AC008474.1 | 0.751327942 | 9.21E-203 | positive |
| BRAF | AC008474.1 | 0.603720728 | 1.72E-111 | positive |
| ATRX | AC008474.1 | 0.42319328 | 1.38E-49 | positive |
| ATRX | AC060766.6 | 0.494015283 | 1.54E-69 | positive |
| OTULIN | AC119800.1 | 0.411067101 | 1.28E-46 | positive |
| DIABLO | AC119800.1 | 0.63438109 | 2.27E-126 | positive |
| BRAF | AC119800.1 | 0.567072343 | 1.06E-95 | positive |
| TSC1 | AC073896.2 | 0.418091943 | 2.54E-48 | positive |
| DIABLO | AC073896.2 | 0.452699372 | 2.48E-57 | positive |
| RNF31 | AC073896.2 | 0.416318779 | 6.89E-48 | positive |
| DIABLO | LINC02033 | 0.409396473 | 3.22E-46 | positive |
| CFLAR | LINC02033 | 0.418774496 | 1.72E-48 | positive |
| OTULIN | TH2LCRR | 0.474204236 | 1.78E-63 | positive |
| CYLD | TH2LCRR | 0.433563372 | 3.20E-52 | positive |
| DIABLO | TH2LCRR | 0.793064879 | 1.99E-241 | positive |
| BRAF | TH2LCRR | 0.689429858 | 8.80E-158 | positive |
| ATRX | TH2LCRR | 0.407365521 | 9.78E-46 | positive |
| MAPK8 | AL050343.2 | 0.485768627 | 5.74E-67 | positive |
| IPMK | AL050343.2 | 0.45889751 | 4.66E-59 | positive |
| OTULIN | AL050343.2 | 0.53544523 | 1.39E-83 | positive |
| CYLD | AL050343.2 | 0.531416051 | 3.93E-82 | positive |
| MAP3K7 | AL050343.2 | 0.445455414 | 2.33E-55 | positive |
| DIABLO | AL050343.2 | 0.766059409 | 1.75E-215 | positive |
| CFLAR | AL050343.2 | 0.509520819 | 1.43E-74 | positive |
| BRAF | AL050343.2 | 0.750867184 | 2.23E-202 | positive |
| ATRX | AL050343.2 | 0.545127999 | 3.70E-87 | positive |
| OTULIN | AC110769.2 | 0.466443808 | 3.29E-61 | positive |
| CYLD | AC110769.2 | 0.469146496 | 5.43E-62 | positive |
| DIABLO | AC110769.2 | 0.750771389 | 2.67E-202 | positive |
| CFLAR | AC110769.2 | 0.427256901 | 1.32E-50 | positive |
| BRAF | AC110769.2 | 0.653778731 | 1.09E-136 | positive |
| ATRX | AC110769.2 | 0.489335744 | 4.52E-68 | positive |
| SIRT3 | CEBPA-DT | 0.449790056 | 1.56E-56 | positive |
| SPATA2 | CEBPA-DT | 0.480165779 | 2.93E-65 | positive |
| HAT1 | AC026401.3 | 0.401748187 | 2.03E-44 | positive |
| TARDBP | AC026401.3 | 0.524401894 | 1.19E-79 | positive |
| MAPK8 | RNF32-AS1 | 0.425022824 | 4.82E-50 | positive |
| DIABLO | RNF32-AS1 | 0.595027405 | 1.47E-107 | positive |
| BRAF | RNF32-AS1 | 0.600704996 | 4.10E-110 | positive |
| CYLD | AL031595.3 | 0.442553618 | 1.40E-54 | positive |
| DIABLO | AL031595.3 | 0.777155311 | 1.06E-225 | positive |
| CFLAR | AL031595.3 | 0.441401535 | 2.83E-54 | positive |
| BRAF | AL031595.3 | 0.643598739 | 3.52E-131 | positive |
| ATRX | AL031595.3 | 0.476461373 | 3.79E-64 | positive |
| CYLD | AL117336.1 | 0.486349031 | 3.80E-67 | positive |
| DIABLO | AL117336.1 | 0.565267898 | 5.65E-95 | positive |
| BRAF | AL117336.1 | 0.588690837 | 9.04E-105 | positive |
| CYLD | AL031710.1 | 0.435978864 | 7.55E-53 | positive |
| DIABLO | AL031710.1 | 0.631388443 | 7.63E-125 | positive |
| CFLAR | AL031710.1 | 0.448675902 | 3.14E-56 | positive |
| BRAF | AL031710.1 | 0.557123216 | 9.50E-92 | positive |
| ATRX | AL031710.1 | 0.478459342 | 9.56E-65 | positive |
| KLF9 | FGF14-AS2 | 0.422247874 | 2.38E-49 | positive |
| DIABLO | AC118658.1 | 0.579927867 | 5.18E-101 | positive |
| BRAF | AC118658.1 | 0.513605756 | 6.12E-76 | positive |
| AXL | AC245041.1 | 0.461601521 | 8.02E-60 | positive |
| MAPK8 | AC007622.2 | 0.433263631 | 3.83E-52 | positive |
| IPMK | AC007622.2 | 0.419634339 | 1.06E-48 | positive |
| OTULIN | AC007622.2 | 0.502391045 | 3.17E-72 | positive |
| CYLD | AC007622.2 | 0.450442719 | 1.03E-56 | positive |
| DIABLO | AC007622.2 | 0.83946602 | 1.53E-296 | positive |
| BRAF | AC007622.2 | 0.715883748 | 1.58E-175 | positive |
| ATRX | AC007622.2 | 0.432000922 | 8.10E-52 | positive |
| OTULIN | AC009032.1 | 0.460924586 | 1.25E-59 | positive |
| DIABLO | AC009032.1 | 0.722354675 | 3.51E-180 | positive |
| BRAF | AC009032.1 | 0.638581617 | 1.53E-128 | positive |
| MAPK8 | AC021851.1 | 0.465603337 | 5.75E-61 | positive |
| IPMK | AC021851.1 | 0.439744407 | 7.77E-54 | positive |
| OTULIN | AC021851.1 | 0.479258766 | 5.50E-65 | positive |
| MAP3K7 | AC021851.1 | 0.491988556 | 6.69E-69 | positive |
| DIABLO | AC021851.1 | 0.686635713 | 5.10E-156 | positive |
| BRAF | AC021851.1 | 0.644637432 | 9.87E-132 | positive |
| ATRX | AC021851.1 | 0.480277016 | 2.71E-65 | positive |
| TSC1 | AC112484.5 | 0.441932788 | 2.04E-54 | positive |
| ATRX | AC112484.5 | 0.455355766 | 4.56E-58 | positive |
| MAPK8 | AL158166.2 | 0.416644565 | 5.74E-48 | positive |
| OTULIN | AL158166.2 | 0.463506933 | 2.30E-60 | positive |
| DIABLO | AL158166.2 | 0.69067683 | 1.42E-158 | positive |
| BRAF | AL158166.2 | 0.620667545 | 1.64E-119 | positive |
| CYLD | SETBP1-DT | 0.401908317 | 1.86E-44 | positive |
| BACH2 | SETBP1-DT | 0.461252466 | 1.01E-59 | positive |
| ATRX | SETBP1-DT | 0.403950236 | 6.22E-45 | positive |
| KLF9 | SETBP1-DT | 0.591020989 | 8.66E-106 | positive |
| TSC1 | AL359921.1 | 0.496413504 | 2.66E-70 | positive |
| MAPK8 | AL359921.1 | 0.406540008 | 1.53E-45 | positive |
| OTULIN | AL359921.1 | 0.476809005 | 2.98E-64 | positive |
| CYLD | AL359921.1 | 0.483528213 | 2.79E-66 | positive |
| DIABLO | AL359921.1 | 0.583552061 | 1.49E-102 | positive |
| CFLAR | AL359921.1 | 0.495212493 | 6.42E-70 | positive |
| BRAF | AL359921.1 | 0.618184392 | 2.64E-118 | positive |
| ATRX | AL359921.1 | 0.555052563 | 6.08E-91 | positive |
| RNF31 | AL359921.1 | 0.522498885 | 5.50E-79 | positive |
| OTULIN | AC026470.2 | 0.490284281 | 2.29E-68 | positive |
| CYLD | AC026470.2 | 0.469734037 | 3.66E-62 | positive |
| DIABLO | AC026470.2 | 0.743710308 | 1.57E-196 | positive |
| BRAF | AC026470.2 | 0.661406778 | 5.87E-141 | positive |
| CD40 | LINC01754 | 0.43635612 | 6.02E-53 | positive |
| BCL2L11 | LINC01754 | 0.403044329 | 1.01E-44 | positive |
| DIABLO | AL589743.3 | 0.533116121 | 9.63E-83 | positive |
| BRAF | AL589743.3 | 0.48309751 | 3.78E-66 | positive |
| DIABLO | AC131159.2 | 0.446540224 | 1.19E-55 | positive |
| CYLD | AL096870.10 | 0.430545783 | 1.91E-51 | positive |
| DIABLO | AL096870.10 | 0.680555649 | 2.99E-152 | positive |
| CFLAR | AL096870.10 | 0.426446578 | 2.11E-50 | positive |
| BRAF | AL096870.10 | 0.635690607 | 4.82E-127 | positive |
| ATRX | AL096870.10 | 0.459040419 | 4.25E-59 | positive |
| DIABLO | ISM1-AS1 | 0.563616633 | 2.59E-94 | positive |
| BRAF | ISM1-AS1 | 0.571372198 | 1.88E-97 | positive |
| ATRX | ISM1-AS1 | 0.458805063 | 4.95E-59 | positive |
| CYLD | RPL34-DT | 0.411569053 | 9.73E-47 | positive |
| DIABLO | RPL34-DT | 0.543471946 | 1.54E-86 | positive |
| CFLAR | RPL34-DT | 0.408897436 | 4.23E-46 | positive |
| BRAF | RPL34-DT | 0.543982574 | 9.94E-87 | positive |
| ATRX | RPL34-DT | 0.463857061 | 1.82E-60 | positive |
| DIABLO | PSD2-AS1 | 0.531702703 | 3.10E-82 | positive |
| MAPK8 | AL731563.3 | 0.407853028 | 7.49E-46 | positive |
| OTULIN | AL731563.3 | 0.454203257 | 9.53E-58 | positive |
| MAP3K7 | AL731563.3 | 0.411203771 | 1.19E-46 | positive |
| DIABLO | AL731563.3 | 0.662539522 | 1.33E-141 | positive |
| CFLAR | AL731563.3 | 0.431243019 | 1.27E-51 | positive |
| BRAF | AL731563.3 | 0.59547426 | 9.27E-108 | positive |
| ATRX | AL731563.3 | 0.519003781 | 8.88E-78 | positive |
| SIRT3 | AC008966.1 | 0.424937406 | 5.06E-50 | positive |
| DIABLO | AC087257.1 | 0.707270381 | 1.57E-169 | positive |
| BRAF | AC087257.1 | 0.602439073 | 6.66E-111 | positive |
| ATRX | AC087257.1 | 0.446250466 | 1.42E-55 | positive |
| CYLD | AL139317.5 | 0.463227472 | 2.76E-60 | positive |
| DIABLO | AL139317.5 | 0.693336229 | 2.79E-160 | positive |
| BRAF | AL139317.5 | 0.63697296 | 1.05E-127 | positive |
| ATRX | AL139317.5 | 0.441161703 | 3.27E-54 | positive |
| BCL2L11 | AC016168.1 | 0.4473008 | 7.41E-56 | positive |
| DIABLO | AC007637.1 | 0.418019898 | 2.64E-48 | positive |
| CFLAR | AC007637.1 | 0.470518949 | 2.16E-62 | positive |
| BRAF | AC007637.1 | 0.440559803 | 4.73E-54 | positive |
| ATRX | AC007637.1 | 0.420913376 | 5.11E-49 | positive |
| KLF9 | AC007637.1 | 0.468523503 | 8.23E-62 | positive |
| CYLD | AP002336.1 | 0.417392903 | 3.76E-48 | positive |
| DIABLO | AP002336.1 | 0.621808797 | 4.55E-120 | positive |
| BRAF | AP002336.1 | 0.533379592 | 7.74E-83 | positive |
| ATRX | AP002336.1 | 0.460951386 | 1.23E-59 | positive |
| OTULIN | AC010168.1 | 0.495734176 | 4.38E-70 | positive |
| CYLD | AC010168.1 | 0.407014706 | 1.18E-45 | positive |
| DIABLO | AC010168.1 | 0.491025624 | 1.34E-68 | positive |
| BRAF | AC010168.1 | 0.523193734 | 3.15E-79 | positive |
| ATRX | AC010168.1 | 0.520384745 | 2.97E-78 | positive |
| DIABLO | OSBPL10-AS1 | 0.755814886 | 1.54E-206 | positive |
| BRAF | OSBPL10-AS1 | 0.60050923 | 5.04E-110 | positive |
| CYLD | IRAG1-AS1 | 0.447119329 | 8.30E-56 | positive |
| DIABLO | IRAG1-AS1 | 0.683883956 | 2.66E-154 | positive |
| CFLAR | IRAG1-AS1 | 0.413474501 | 3.38E-47 | positive |
| BRAF | IRAG1-AS1 | 0.616553801 | 1.61E-117 | positive |
| ATRX | IRAG1-AS1 | 0.401053371 | 2.94E-44 | positive |
| OTULIN | AC084876.1 | 0.471801077 | 9.08E-63 | positive |
| CYLD | AC084876.1 | 0.498961033 | 4.07E-71 | positive |
| MAP3K7 | AC084876.1 | 0.404087153 | 5.78E-45 | positive |
| DIABLO | AC084876.1 | 0.643864778 | 2.54E-131 | positive |
| CFLAR | AC084876.1 | 0.438853263 | 1.33E-53 | positive |
| BRAF | AC084876.1 | 0.616292091 | 2.16E-117 | positive |
| ATRX | AC084876.1 | 0.454193817 | 9.59E-58 | positive |
| CYLD | AC112721.1 | 0.407131679 | 1.11E-45 | positive |
| DIABLO | AC107072.1 | 0.730802019 | 1.85E-186 | positive |
| BRAF | AC107072.1 | 0.587312145 | 3.59E-104 | positive |
| DIABLO | AC064836.1 | 0.583722341 | 1.26E-102 | positive |
| BRAF | AC064836.1 | 0.510697536 | 5.80E-75 | positive |
| BACH2 | ST3GAL5-AS1 | 0.41056255 | 1.70E-46 | positive |
| DIABLO | AC022215.2 | 0.673268503 | 7.48E-148 | positive |
| BRAF | AC022215.2 | 0.581181306 | 1.53E-101 | positive |
| OTULIN | DIAPH2-AS1 | 0.447937025 | 4.98E-56 | positive |
| CYLD | DIAPH2-AS1 | 0.411688842 | 9.10E-47 | positive |
| DIABLO | DIAPH2-AS1 | 0.757568979 | 4.86E-208 | positive |
| BRAF | DIAPH2-AS1 | 0.675764565 | 2.41E-149 | positive |
| ATRX | DIAPH2-AS1 | 0.444284476 | 4.81E-55 | positive |
| OTULIN | AC145146.1 | 0.401594231 | 2.20E-44 | positive |
| DIABLO | AC145146.1 | 0.778587588 | 4.61E-227 | positive |
| BRAF | AC145146.1 | 0.629568921 | 6.35E-124 | positive |
| CYLD | AC012085.2 | 0.400620621 | 3.70E-44 | positive |
| DIABLO | AC012085.2 | 0.703631893 | 4.60E-167 | positive |
| BRAF | AC012085.2 | 0.612780394 | 1.02E-115 | positive |
| TRAF2 | C9orf163 | 0.416448792 | 6.41E-48 | positive |
| DIABLO | AL137025.1 | 0.549373534 | 9.23E-89 | positive |
| BRAF | AL137025.1 | 0.44915951 | 2.32E-56 | positive |
| ATRX | AL137025.1 | 0.426758669 | 1.76E-50 | positive |
| CYLD | AC025180.1 | 0.426068908 | 2.63E-50 | positive |
| DIABLO | AC025180.1 | 0.728935542 | 4.72E-185 | positive |
| BRAF | AC025180.1 | 0.656672968 | 2.71E-138 | positive |
| ATRX | AC025180.1 | 0.412686859 | 5.24E-47 | positive |
| DIABLO | AP000864.1 | 0.409576865 | 2.92E-46 | positive |
| DIABLO | AC008758.2 | 0.583036596 | 2.48E-102 | positive |
| BRAF | AC008758.2 | 0.467812427 | 1.32E-61 | positive |
| ATRX | AC145285.2 | 0.490927834 | 1.44E-68 | positive |
| KLF9 | HLX-AS1 | 0.456133229 | 2.77E-58 | positive |
| TSC1 | AC087289.1 | 0.464042986 | 1.61E-60 | positive |
| DIABLO | AC087289.1 | 0.523470191 | 2.53E-79 | positive |
| BRAF | AC087289.1 | 0.473600722 | 2.68E-63 | positive |
| ATRX | AC087289.1 | 0.429879561 | 2.83E-51 | positive |
| RNF31 | AC087289.1 | 0.404531463 | 4.55E-45 | positive |
| TSC1 | AL021707.2 | 0.429224738 | 4.16E-51 | positive |
| DIABLO | AL021707.2 | 0.406229967 | 1.81E-45 | positive |
| BRAF | AL021707.2 | 0.476480303 | 3.74E-64 | positive |
| ATRX | AL021707.2 | 0.589666811 | 3.39E-105 | positive |
| RNF31 | AL021707.2 | 0.486032649 | 4.76E-67 | positive |
| TSC1 | AC096586.2 | 0.421158594 | 4.44E-49 | positive |
| MAPK8 | AC096586.2 | 0.468157116 | 1.05E-61 | positive |
| IPMK | AC096586.2 | 0.402000471 | 1.77E-44 | positive |
| OTULIN | AC096586.2 | 0.504663282 | 5.75E-73 | positive |
| CYLD | AC096586.2 | 0.473503119 | 2.86E-63 | positive |
| MAP3K7 | AC096586.2 | 0.436855243 | 4.46E-53 | positive |
| DIABLO | AC096586.2 | 0.698139292 | 2.08E-163 | positive |
| CFLAR | AC096586.2 | 0.442646207 | 1.32E-54 | positive |
| BRAF | AC096586.2 | 0.667138858 | 3.00E-144 | positive |
| ATRX | AC096586.2 | 0.494470798 | 1.10E-69 | positive |
| GATA3 | AL445584.2 | 0.457318494 | 1.29E-58 | positive |
| OTULIN | AC002347.2 | 0.42004044 | 8.40E-49 | positive |
| DIABLO | AC002347.2 | 0.628987087 | 1.25E-123 | positive |
| BRAF | AC002347.2 | 0.554474687 | 1.02E-90 | positive |
| ATRX | AC002347.2 | 0.4051898 | 3.19E-45 | positive |
| TSC1 | AL139099.2 | 0.472482115 | 5.73E-63 | positive |
| ATRX | AL139099.2 | 0.402447055 | 1.40E-44 | positive |
| RNF31 | AL139099.2 | 0.4422161 | 1.72E-54 | positive |
| OTULIN | TNK2-AS1 | 0.408996587 | 4.01E-46 | positive |
| CYLD | TNK2-AS1 | 0.525639762 | 4.40E-80 | positive |
| DIABLO | TNK2-AS1 | 0.476171724 | 4.62E-64 | positive |
| BRAF | TNK2-AS1 | 0.426320893 | 2.27E-50 | positive |
| CYLD | AP000654.1 | 0.43007555 | 2.52E-51 | positive |
| DIABLO | AP000654.1 | 0.691454976 | 4.51E-159 | positive |
| CFLAR | AP000654.1 | 0.410649613 | 1.62E-46 | positive |
| BRAF | AP000654.1 | 0.650483678 | 6.99E-135 | positive |
| ATRX | AP000654.1 | 0.496967634 | 1.77E-70 | positive |
| CYLD | AC010333.2 | 0.41947622 | 1.16E-48 | positive |
| DIABLO | AC010333.2 | 0.643391848 | 4.53E-131 | positive |
| CFLAR | AC010333.2 | 0.431439525 | 1.13E-51 | positive |
| BRAF | AC010333.2 | 0.540112771 | 2.72E-85 | positive |
| ATRX | AC010333.2 | 0.447951389 | 4.94E-56 | positive |
| BACH2 | LINC02397 | 0.625892278 | 4.40E-122 | positive |
| TSC1 | THUMPD3-AS1 | 0.43051969 | 1.94E-51 | positive |
| MAP3K7 | THUMPD3-AS1 | 0.441337201 | 2.94E-54 | positive |
| DIABLO | THUMPD3-AS1 | 0.574031388 | 1.51E-98 | positive |
| BRAF | THUMPD3-AS1 | 0.502690222 | 2.53E-72 | positive |
| DIABLO | AC011466.2 | 0.632236915 | 2.83E-125 | positive |
| BRAF | AC011466.2 | 0.491621227 | 8.72E-69 | positive |
| CFLAR | AC068491.1 | 0.44075773 | 4.19E-54 | positive |
| ATRX | AC068491.1 | 0.462908176 | 3.40E-60 | positive |
| OTULIN | AC005072.1 | 0.44411357 | 5.35E-55 | positive |
| CYLD | AC005072.1 | 0.453263894 | 1.73E-57 | positive |
| DIABLO | AC005072.1 | 0.80608226 | 2.30E-255 | positive |
| CFLAR | AC005072.1 | 0.475932103 | 5.45E-64 | positive |
| BRAF | AC005072.1 | 0.698738747 | 8.37E-164 | positive |
| ATRX | AC005072.1 | 0.539755987 | 3.68E-85 | positive |
| DIABLO | AP001011.1 | 0.620340189 | 2.38E-119 | positive |
| BRAF | AP001011.1 | 0.497100869 | 1.61E-70 | positive |
| MAPK8 | KCNQ1OT1 | 0.415693849 | 9.79E-48 | positive |
| OTULIN | KCNQ1OT1 | 0.413534459 | 3.27E-47 | positive |
| DIABLO | KCNQ1OT1 | 0.72847587 | 1.04E-184 | positive |
| CFLAR | KCNQ1OT1 | 0.410665229 | 1.60E-46 | positive |
| BRAF | KCNQ1OT1 | 0.647689296 | 2.28E-133 | positive |
| ATRX | KCNQ1OT1 | 0.504939867 | 4.67E-73 | positive |
| DIABLO | AL357793.1 | 0.586884649 | 5.50E-104 | positive |
| BRAF | AL357793.1 | 0.516634288 | 5.74E-77 | positive |
| TSC1 | AC104335.1 | 0.409451738 | 3.12E-46 | positive |
| OTULIN | AC104335.1 | 0.407044341 | 1.16E-45 | positive |
| DIABLO | AC104335.1 | 0.618088825 | 2.94E-118 | positive |
| CFLAR | AC104335.1 | 0.407137954 | 1.11E-45 | positive |
| BRAF | AC104335.1 | 0.586592506 | 7.36E-104 | positive |
| ATRX | AC104335.1 | 0.491982756 | 6.72E-69 | positive |
| DIABLO | AC137561.1 | 0.529943056 | 1.32E-81 | positive |
| BRAF | AC137561.1 | 0.473123885 | 3.70E-63 | positive |
| DIABLO | AC100791.3 | 0.633079526 | 1.05E-125 | positive |
| BRAF | AC100791.3 | 0.580079151 | 4.47E-101 | positive |
| ATRX | AC100791.3 | 0.424322524 | 7.22E-50 | positive |
| DIABLO | AC026355.3 | 0.585190374 | 2.96E-103 | positive |
| BRAF | AC026355.3 | 0.582865127 | 2.93E-102 | positive |
| MAPK8 | AC004039.1 | 0.415352664 | 1.18E-47 | positive |
| IPMK | AC004039.1 | 0.411473273 | 1.03E-46 | positive |
| OTULIN | AC004039.1 | 0.452762762 | 2.38E-57 | positive |
| CYLD | AC004039.1 | 0.494056485 | 1.49E-69 | positive |
| DIABLO | AC004039.1 | 0.784186727 | 1.74E-232 | positive |
| BRAF | AC004039.1 | 0.70315198 | 9.67E-167 | positive |
| ATRX | AC004039.1 | 0.454818538 | 6.43E-58 | positive |
| CYLD | AC027279.1 | 0.40762469 | 8.49E-46 | positive |
| DIABLO | AC027279.1 | 0.712936019 | 1.89E-173 | positive |
| BRAF | AC027279.1 | 0.664459688 | 1.06E-142 | positive |
| ATRX | AC027279.1 | 0.514191722 | 3.88E-76 | positive |
| STUB1 | SNHG21 | 0.407839182 | 7.55E-46 | positive |
| MAPK8 | AL031668.1 | 0.406944087 | 1.23E-45 | positive |
| OTULIN | AL031668.1 | 0.460690806 | 1.45E-59 | positive |
| CYLD | AL031668.1 | 0.412965822 | 4.49E-47 | positive |
| DIABLO | AL031668.1 | 0.820942704 | 1.25E-272 | positive |
| BRAF | AL031668.1 | 0.70143321 | 1.37E-165 | positive |
| ATRX | AL031668.1 | 0.427498496 | 1.14E-50 | positive |
| DIABLO | AC002128.2 | 0.703628663 | 4.62E-167 | positive |
| CFLAR | AC002128.2 | 0.441778114 | 2.25E-54 | positive |
| BRAF | AC002128.2 | 0.574290955 | 1.18E-98 | positive |
| ATRX | AC002128.2 | 0.457036985 | 1.55E-58 | positive |
| MAPK8 | AC021739.2 | 0.457878682 | 9.01E-59 | positive |
| DIABLO | AC021739.2 | 0.646727113 | 7.52E-133 | positive |
| CFLAR | AC021739.2 | 0.492498162 | 4.62E-69 | positive |
| BRAF | AC021739.2 | 0.596368402 | 3.70E-108 | positive |
| ATRX | AC021739.2 | 0.450577252 | 9.50E-57 | positive |
| CFLAR | AC026150.1 | 0.422304233 | 2.31E-49 | positive |
| ATRX | AC026150.1 | 0.495452078 | 5.39E-70 | positive |
| KLF9 | AC026150.1 | 0.474584338 | 1.37E-63 | positive |
| TARDBP | AC019069.1 | 0.416941281 | 4.86E-48 | positive |
| OTULIN | EGOT | 0.481596774 | 1.08E-65 | positive |
| CYLD | EGOT | 0.439251437 | 1.05E-53 | positive |
| DIABLO | EGOT | 0.644371193 | 1.37E-131 | positive |
| BRAF | EGOT | 0.601769742 | 1.35E-110 | positive |
| DIABLO | AC068768.1 | 0.498295509 | 6.66E-71 | positive |
| OTULIN | AL731577.1 | 0.401017549 | 3.00E-44 | positive |
| CYLD | AL731577.1 | 0.410491756 | 1.76E-46 | positive |
| DIABLO | AL731577.1 | 0.743546442 | 2.13E-196 | positive |
| BRAF | AL731577.1 | 0.656488784 | 3.43E-138 | positive |
| ATRX | AL731577.1 | 0.411182791 | 1.20E-46 | positive |
| MAPK8 | AC125437.1 | 0.41976283 | 9.84E-49 | positive |
| OTULIN | AC125437.1 | 0.454529589 | 7.74E-58 | positive |
| MAP3K7 | AC125437.1 | 0.410204953 | 2.06E-46 | positive |
| DIABLO | AC125437.1 | 0.492796283 | 3.73E-69 | positive |
| BRAF | AC125437.1 | 0.483772803 | 2.35E-66 | positive |
| CYLD | LINC02694 | 0.438727643 | 1.44E-53 | positive |
| DIABLO | LINC02694 | 0.65915779 | 1.10E-139 | positive |
| BRAF | LINC02694 | 0.583577328 | 1.46E-102 | positive |
| ATRX | LINC02694 | 0.405153217 | 3.25E-45 | positive |
| DIABLO | DELEC1 | 0.439824698 | 7.40E-54 | positive |
| BRAF | DELEC1 | 0.412712444 | 5.16E-47 | positive |
| DIABLO | SYNJ2-IT1 | 0.789979362 | 2.85E-238 | positive |
| BRAF | SYNJ2-IT1 | 0.613893362 | 3.02E-116 | positive |
| DIABLO | LIX1-AS1 | 0.411127645 | 1.24E-46 | positive |
| DIABLO | AC107464.1 | 0.631898273 | 4.21E-125 | positive |
| BRAF | AC107464.1 | 0.456002066 | 3.02E-58 | positive |
| RNF31 | AC107464.1 | 0.411796511 | 8.58E-47 | positive |
| DIABLO | AC013643.2 | 0.567159495 | 9.77E-96 | positive |
| BRAF | AC013643.2 | 0.528336214 | 4.92E-81 | positive |
| KLF9 | AC012101.2 | 0.580834178 | 2.14E-101 | positive |
| DIABLO | AL645768.1 | 0.598403683 | 4.51E-109 | positive |
| BRAF | AL645768.1 | 0.518576342 | 1.24E-77 | positive |
| MAPK8 | LINC00216 | 0.435382774 | 1.08E-52 | positive |
| IPMK | LINC00216 | 0.417205509 | 4.18E-48 | positive |
| OTULIN | LINC00216 | 0.494364237 | 1.19E-69 | positive |
| CYLD | LINC00216 | 0.488766378 | 6.79E-68 | positive |
| MAP3K7 | LINC00216 | 0.407555619 | 8.81E-46 | positive |
| DIABLO | LINC00216 | 0.797654171 | 3.17E-246 | positive |
| CFLAR | LINC00216 | 0.430572139 | 1.88E-51 | positive |
| BRAF | LINC00216 | 0.720619929 | 6.39E-179 | positive |
| ATRX | LINC00216 | 0.472393797 | 6.08E-63 | positive |
| AXL | MANCR | 0.465184077 | 7.60E-61 | positive |
| DIABLO | AC018809.1 | 0.544643005 | 5.63E-87 | positive |
| BRAF | AC018809.1 | 0.472411793 | 6.00E-63 | positive |
| DIABLO | AL353801.1 | 0.659793735 | 4.81E-140 | positive |
| BRAF | AL353801.1 | 0.580887454 | 2.03E-101 | positive |
| ATRX | AC067930.9 | 0.427339644 | 1.25E-50 | positive |
| DIABLO | AC093799.1 | 0.762153952 | 5.09E-212 | positive |
| CFLAR | AC093799.1 | 0.409264495 | 3.46E-46 | positive |
| BRAF | AC093799.1 | 0.618360499 | 2.17E-118 | positive |
| ATRX | AC093799.1 | 0.422798591 | 1.74E-49 | positive |
| DIABLO | AC008429.1 | 0.421030876 | 4.78E-49 | positive |
| OTULIN | AC097658.3 | 0.453470845 | 1.52E-57 | positive |
| CYLD | AC097658.3 | 0.413550163 | 3.24E-47 | positive |
| DIABLO | AC097658.3 | 0.789007259 | 2.74E-237 | positive |
| BRAF | AC097658.3 | 0.680394913 | 3.76E-152 | positive |
| TSC1 | AC139887.2 | 0.468004642 | 1.16E-61 | positive |
| DIABLO | AC139887.2 | 0.463030448 | 3.14E-60 | positive |
| CFLAR | AC139887.2 | 0.425727886 | 3.20E-50 | positive |
| BRAF | AC139887.2 | 0.466945455 | 2.36E-61 | positive |
| ATRX | AC139887.2 | 0.487711621 | 1.44E-67 | positive |
| RNF31 | AC139887.2 | 0.443978085 | 5.81E-55 | positive |
| MYC | AC124067.4 | 0.497415966 | 1.27E-70 | positive |
| SPATA2 | AC124067.4 | 0.498274798 | 6.76E-71 | positive |
| OTULIN | AC096708.3 | 0.451418384 | 5.59E-57 | positive |
| CYLD | AC096708.3 | 0.439500631 | 9.01E-54 | positive |
| DIABLO | AC096708.3 | 0.710560048 | 8.53E-172 | positive |
| BRAF | AC096708.3 | 0.662942529 | 7.83E-142 | positive |
| ATRX | AC096708.3 | 0.441132158 | 3.33E-54 | positive |
| OTULIN | AC103591.3 | 0.430275294 | 2.24E-51 | positive |
| DIABLO | AC103591.3 | 0.616739315 | 1.31E-117 | positive |
| BRAF | AC103591.3 | 0.548768017 | 1.57E-88 | positive |
| OTULIN | MIR17HG | 0.452280382 | 3.24E-57 | positive |
| MAP3K7 | MIR17HG | 0.411479224 | 1.02E-46 | positive |
| DIABLO | MIR17HG | 0.691438184 | 4.62E-159 | positive |
| BRAF | MIR17HG | 0.575876553 | 2.59E-99 | positive |
| DIABLO | TPT1-AS1 | 0.540884855 | 1.41E-85 | positive |
| BRAF | TPT1-AS1 | 0.46570305 | 5.39E-61 | positive |
| DIABLO | AC087742.1 | 0.463056571 | 3.09E-60 | positive |
| SPATA2 | AL118506.1 | 0.476995912 | 2.62E-64 | positive |
| OTULIN | ITCH-AS1 | 0.414649468 | 1.76E-47 | positive |
| DIABLO | ITCH-AS1 | 0.737024886 | 3.06E-191 | positive |
| BRAF | ITCH-AS1 | 0.621175486 | 9.29E-120 | positive |
| ATRX | ITCH-AS1 | 0.424331321 | 7.18E-50 | positive |
| DIABLO | AL157400.2 | 0.449964378 | 1.40E-56 | positive |
| OTULIN | PDXP-DT | 0.403422445 | 8.27E-45 | positive |
| DIABLO | PDXP-DT | 0.553047822 | 3.62E-90 | positive |
| BRAF | PDXP-DT | 0.440822375 | 4.03E-54 | positive |
| ATRX | LINC01967 | 0.415406593 | 1.15E-47 | positive |
| CYLD | MALAT1 | 0.412698827 | 5.20E-47 | positive |
| DIABLO | MALAT1 | 0.714997133 | 6.70E-175 | positive |
| CFLAR | MALAT1 | 0.48013208 | 3.00E-65 | positive |
| BRAF | MALAT1 | 0.638765383 | 1.23E-128 | positive |
| ATRX | MALAT1 | 0.578197106 | 2.77E-100 | positive |
| RNF31 | MALAT1 | 0.404583427 | 4.42E-45 | positive |
| TSC1 | AC015871.3 | 0.424224394 | 7.64E-50 | positive |
| MAPK8 | AC015871.3 | 0.462180849 | 5.49E-60 | positive |
| OTULIN | AC015871.3 | 0.472859356 | 4.43E-63 | positive |
| CYLD | AC015871.3 | 0.488124749 | 1.07E-67 | positive |
| MAP3K7 | AC015871.3 | 0.462012236 | 6.13E-60 | positive |
| DIABLO | AC015871.3 | 0.688471389 | 3.56E-157 | positive |
| CFLAR | AC015871.3 | 0.492809835 | 3.69E-69 | positive |
| BRAF | AC015871.3 | 0.685526228 | 2.52E-155 | positive |
| ATRX | AC015871.3 | 0.46911488 | 5.54E-62 | positive |
| IPMK | AL450992.2 | 0.41092714 | 1.39E-46 | positive |
| CYLD | AL450992.2 | 0.438935775 | 1.27E-53 | positive |
| DIABLO | AL450992.2 | 0.75586779 | 1.38E-206 | positive |
| CFLAR | AL450992.2 | 0.457026089 | 1.56E-58 | positive |
| BRAF | AL450992.2 | 0.664591709 | 8.88E-143 | positive |
| ATRX | AL450992.2 | 0.564525212 | 1.12E-94 | positive |
| RNF31 | AL450992.2 | 0.427381291 | 1.22E-50 | positive |
| DIABLO | AL137246.1 | 0.533904806 | 5.00E-83 | positive |
| BRAF | AL137246.1 | 0.511479338 | 3.17E-75 | positive |
| RNF31 | AC013356.2 | 0.404360909 | 4.99E-45 | positive |
| CYLD | AC012184.4 | 0.408916062 | 4.19E-46 | positive |
| DIABLO | AC012184.4 | 0.763512667 | 3.23E-213 | positive |
| BRAF | AC012184.4 | 0.602401654 | 6.92E-111 | positive |
| ATRX | AC012184.4 | 0.469412196 | 4.54E-62 | positive |
| TSC1 | AP001033.2 | 0.40784131 | 7.54E-46 | positive |
| MAPK8 | AP001033.2 | 0.465036816 | 8.37E-61 | positive |
| IPMK | AP001033.2 | 0.47079769 | 1.79E-62 | positive |
| OTULIN | AP001033.2 | 0.483225825 | 3.45E-66 | positive |
| CYLD | AP001033.2 | 0.519760515 | 4.88E-78 | positive |
| MAP3K7 | AP001033.2 | 0.452398347 | 3.00E-57 | positive |
| DIABLO | AP001033.2 | 0.741675172 | 6.67E-195 | positive |
| CFLAR | AP001033.2 | 0.496808146 | 1.99E-70 | positive |
| BRAF | AP001033.2 | 0.752377151 | 1.22E-203 | positive |
| ATRX | AP001033.2 | 0.566775787 | 1.40E-95 | positive |
| TSC1 | LINC02604 | 0.410927174 | 1.39E-46 | positive |
| RNF31 | LINC02604 | 0.422250111 | 2.38E-49 | positive |
| DIABLO | AP001178.3 | 0.52641577 | 2.34E-80 | positive |
| BRAF | AP001178.3 | 0.401198833 | 2.72E-44 | positive |
| OTULIN | AC040934.1 | 0.421915228 | 2.88E-49 | positive |
| DIABLO | AC040934.1 | 0.649461445 | 2.51E-134 | positive |
| CFLAR | AC040934.1 | 0.432776875 | 5.11E-52 | positive |
| BRAF | AC040934.1 | 0.634734146 | 1.50E-126 | positive |
| ATRX | AC040934.1 | 0.557165887 | 9.14E-92 | positive |
| BCL2 | ITPKB-IT1 | 0.44667055 | 1.10E-55 | positive |
| BCL2L11 | ITPKB-IT1 | 0.474002905 | 2.04E-63 | positive |
| BCL2 | AL353648.1 | 0.407060821 | 1.15E-45 | positive |
| BCL2L11 | AL353648.1 | 0.41498912 | 1.45E-47 | positive |
| DIABLO | AL513218.2 | 0.400683737 | 3.58E-44 | positive |
| DIABLO | AL158832.1 | 0.517386562 | 3.18E-77 | positive |
| BRAF | AL158832.1 | 0.469505227 | 4.26E-62 | positive |
| OTULIN | MYLK-AS1 | 0.494573797 | 1.02E-69 | positive |
| CYLD | MYLK-AS1 | 0.407540793 | 8.89E-46 | positive |
| DIABLO | MYLK-AS1 | 0.700207028 | 8.94E-165 | positive |
| BRAF | MYLK-AS1 | 0.613365415 | 5.39E-116 | positive |
| ATRX | MYLK-AS1 | 0.409078119 | 3.83E-46 | positive |
| TSC1 | XPC-AS1 | 0.583896737 | 1.06E-102 | positive |
| MAPK8 | XPC-AS1 | 0.449624278 | 1.73E-56 | positive |
| OTULIN | XPC-AS1 | 0.454577347 | 7.51E-58 | positive |
| CYLD | XPC-AS1 | 0.546323724 | 1.32E-87 | positive |
| MAP3K7 | XPC-AS1 | 0.444425426 | 4.41E-55 | positive |
| DIABLO | XPC-AS1 | 0.695418938 | 1.25E-161 | positive |
| CFLAR | XPC-AS1 | 0.59987595 | 9.76E-110 | positive |
| BRAF | XPC-AS1 | 0.682484902 | 1.95E-153 | positive |
| ATRX | XPC-AS1 | 0.56077863 | 3.48E-93 | positive |
| RNF31 | XPC-AS1 | 0.50080946 | 1.03E-71 | positive |
| DIABLO | AL136419.2 | 0.490575214 | 1.85E-68 | positive |
| DIABLO | AC007271.1 | 0.447384095 | 7.03E-56 | positive |
| BRAF | AC007271.1 | 0.432793684 | 5.06E-52 | positive |
| MAPK8 | PTPRG-AS1 | 0.416725157 | 5.48E-48 | positive |
| OTULIN | PTPRG-AS1 | 0.497288827 | 1.40E-70 | positive |
| CYLD | PTPRG-AS1 | 0.418320606 | 2.23E-48 | positive |
| MAP3K7 | PTPRG-AS1 | 0.428724788 | 5.58E-51 | positive |
| DIABLO | PTPRG-AS1 | 0.761880636 | 8.84E-212 | positive |
| BRAF | PTPRG-AS1 | 0.70072719 | 4.04E-165 | positive |
| ATRX | PTPRG-AS1 | 0.409026176 | 3.94E-46 | positive |
| TSC1 | AL592166.1 | 0.443631679 | 7.20E-55 | positive |
| OTULIN | AL592166.1 | 0.439871767 | 7.19E-54 | positive |
| DIABLO | AL592166.1 | 0.581605205 | 1.01E-101 | positive |
| BRAF | AL592166.1 | 0.4912402 | 1.15E-68 | positive |
| ATRX | AL592166.1 | 0.439217027 | 1.07E-53 | positive |
| RNF31 | AL592166.1 | 0.432039606 | 7.92E-52 | positive |
| DIABLO | LINC02157 | 0.505908725 | 2.24E-73 | positive |
| CFLAR | LINC02157 | 0.463507728 | 2.30E-60 | positive |
| BRAF | LINC02157 | 0.462120742 | 5.71E-60 | positive |
| ATRX | LINC02157 | 0.464568953 | 1.14E-60 | positive |
| KLF9 | LINC02157 | 0.416590566 | 5.91E-48 | positive |
| KLF9 | PART1 | 0.469928816 | 3.21E-62 | positive |
| OTULIN | AL157369.1 | 0.400299937 | 4.39E-44 | positive |
| DIABLO | AL157369.1 | 0.626987868 | 1.25E-122 | positive |
| BRAF | AL157369.1 | 0.547585337 | 4.40E-88 | positive |
| RIPK3 | AC009065.5 | 0.401609708 | 2.18E-44 | positive |
| CYLD | DIRC3 | 0.419113189 | 1.42E-48 | positive |
| DIABLO | DIRC3 | 0.453545218 | 1.45E-57 | positive |
| BRAF | DIRC3 | 0.496903853 | 1.86E-70 | positive |
| DIABLO | AC104461.1 | 0.423318396 | 1.29E-49 | positive |
| BRAF | LINC02584 | 0.400524224 | 3.90E-44 | positive |
| CD40 | AC095057.4 | 0.563025298 | 4.46E-94 | positive |
| BCL2L11 | AC095057.4 | 0.415654317 | 1.00E-47 | positive |
| CYLD | L3MBTL2-AS1 | 0.43342821 | 3.47E-52 | positive |
| DIABLO | L3MBTL2-AS1 | 0.429824347 | 2.93E-51 | positive |
| BRAF | L3MBTL2-AS1 | 0.447986182 | 4.83E-56 | positive |
| BACH2 | L3MBTL2-AS1 | 0.489151791 | 5.15E-68 | positive |
| ATRX | L3MBTL2-AS1 | 0.442964709 | 1.08E-54 | positive |
| DIABLO | LINC00626 | 0.456832375 | 1.77E-58 | positive |
| OTULIN | AC025030.2 | 0.447440316 | 6.79E-56 | positive |
| DIABLO | AC025030.2 | 0.757821568 | 2.95E-208 | positive |
| BRAF | AC025030.2 | 0.656484876 | 3.45E-138 | positive |
| DIABLO | AC026765.3 | 0.43335495 | 3.62E-52 | positive |
| TSC1 | AL359878.1 | 0.489070015 | 5.46E-68 | positive |
| MAPK8 | AL359878.1 | 0.400350929 | 4.28E-44 | positive |
| DIABLO | AL359878.1 | 0.592587984 | 1.77E-106 | positive |
| CFLAR | AL359878.1 | 0.450267531 | 1.15E-56 | positive |
| BRAF | AL359878.1 | 0.580581889 | 2.74E-101 | positive |
| ATRX | AL359878.1 | 0.554765857 | 7.85E-91 | positive |
| RNF31 | AL359878.1 | 0.402499 | 1.36E-44 | positive |
| DIABLO | ABCA9-AS1 | 0.403475813 | 8.03E-45 | positive |
| MAPK8 | AC012467.1 | 0.405829801 | 2.25E-45 | positive |
| DIABLO | AC012467.1 | 0.57727792 | 6.74E-100 | positive |
| BRAF | AC012467.1 | 0.573004019 | 4.02E-98 | positive |
| DIABLO | AC013564.1 | 0.668856063 | 2.99E-145 | positive |
| BRAF | AC013564.1 | 0.531654795 | 3.23E-82 | positive |
| DIABLO | AL360091.1 | 0.534218162 | 3.85E-83 | positive |
| BRAF | AL360091.1 | 0.504645653 | 5.83E-73 | positive |
| ATRX | AL360091.1 | 0.492761433 | 3.82E-69 | positive |
| SPATA2 | LINC01006 | 0.404731375 | 4.08E-45 | positive |
| MAPK8 | CASC2 | 0.459088136 | 4.12E-59 | positive |
| IPMK | CASC2 | 0.402040129 | 1.74E-44 | positive |
| DIABLO | CASC2 | 0.514367243 | 3.38E-76 | positive |
| BRAF | CASC2 | 0.560202953 | 5.87E-93 | positive |
| TSC1 | AC011479.2 | 0.480210901 | 2.84E-65 | positive |
| DIABLO | AC011479.2 | 0.551746052 | 1.15E-89 | positive |
| CFLAR | AC011479.2 | 0.481775931 | 9.53E-66 | positive |
| BRAF | AC011479.2 | 0.545063822 | 3.92E-87 | positive |
| ATRX | AC011479.2 | 0.539269553 | 5.55E-85 | positive |
| RNF31 | AC011479.2 | 0.455124745 | 5.29E-58 | positive |
| OTULIN | AL109824.1 | 0.422381301 | 2.21E-49 | positive |
| DIABLO | AL109824.1 | 0.75967372 | 7.43E-210 | positive |
| BRAF | AL109824.1 | 0.670939502 | 1.79E-146 | positive |
| ATRX | AL109824.1 | 0.434323298 | 2.04E-52 | positive |
| DIABLO | AC097500.1 | 0.649724862 | 1.81E-134 | positive |
| BRAF | AC097500.1 | 0.586116312 | 1.18E-103 | positive |
| DIABLO | AC003101.2 | 0.435437354 | 1.05E-52 | positive |
| MAP3K7 | AL442067.3 | 0.412154069 | 7.04E-47 | positive |
| DIABLO | AC012676.4 | 0.620235522 | 2.67E-119 | positive |
| BRAF | AC012676.4 | 0.420847866 | 5.30E-49 | positive |
| DIABLO | AC006449.3 | 0.41218654 | 6.91E-47 | positive |
| BRAF | AC006449.3 | 0.440294858 | 5.56E-54 | positive |
| CYLD | ADAMTS9-AS1 | 0.432158822 | 7.38E-52 | positive |
| DIABLO | ADAMTS9-AS1 | 0.564920709 | 7.79E-95 | positive |
| BRAF | ADAMTS9-AS1 | 0.566143724 | 2.51E-95 | positive |
| KLF9 | ADAMTS9-AS1 | 0.470913616 | 1.65E-62 | positive |
| DIABLO | AL138999.1 | 0.49752605 | 1.18E-70 | positive |
| DIABLO | AC000061.1 | 0.434953214 | 1.40E-52 | positive |
| OTULIN | AP001160.4 | 0.413779541 | 2.85E-47 | positive |
| CYLD | AP001160.4 | 0.401891498 | 1.88E-44 | positive |
| DIABLO | AP001160.4 | 0.710531401 | 8.93E-172 | positive |
| CFLAR | AP001160.4 | 0.410327214 | 1.93E-46 | positive |
| BRAF | AP001160.4 | 0.625028519 | 1.18E-121 | positive |
| ATRX | AP001160.4 | 0.432067912 | 7.78E-52 | positive |
| GATA3 | SNHG31 | 0.536598323 | 5.27E-84 | positive |
| DIABLO | AC124319.1 | 0.424865873 | 5.27E-50 | positive |
| TSC1 | AC067852.3 | 0.445045645 | 3.01E-55 | positive |
| CYLD | AC067852.3 | 0.400593517 | 3.76E-44 | positive |
| DIABLO | AC067852.3 | 0.667725224 | 1.37E-144 | positive |
| CFLAR | AC067852.3 | 0.50503772 | 4.33E-73 | positive |
| BRAF | AC067852.3 | 0.61824434 | 2.47E-118 | positive |
| ATRX | AC067852.3 | 0.53255058 | 1.54E-82 | positive |
| RNF31 | AC067852.3 | 0.470985893 | 1.57E-62 | positive |
| DIABLO | CFTR-AS1 | 0.593440564 | 7.43E-107 | positive |
| BRAF | CFTR-AS1 | 0.498360618 | 6.35E-71 | positive |
| DIABLO | LINC01990 | 0.440485104 | 4.95E-54 | positive |
| BRAF | LINC01990 | 0.445211069 | 2.71E-55 | positive |
| ATRX | LINC01990 | 0.47797384 | 1.34E-64 | positive |
| KLF9 | LINC01990 | 0.466732983 | 2.72E-61 | positive |
| TSC1 | AL513314.2 | 0.426558664 | 1.98E-50 | positive |
| ATRX | AL513314.2 | 0.586862239 | 5.62E-104 | positive |
| RNF31 | AL513314.2 | 0.426600272 | 1.93E-50 | positive |
| MAPK8 | AC079142.1 | 0.429054146 | 4.60E-51 | positive |
| OTULIN | AC079142.1 | 0.485395688 | 7.47E-67 | positive |
| MAP3K7 | AC079142.1 | 0.425238888 | 4.25E-50 | positive |
| DIABLO | AC079142.1 | 0.662594087 | 1.24E-141 | positive |
| BRAF | AC079142.1 | 0.603859677 | 1.49E-111 | positive |
| KLF9 | AL109741.1 | 0.615877132 | 3.41E-117 | positive |
| KLF9 | AC090877.2 | 0.432403859 | 6.38E-52 | positive |
| DIABLO | MIR583HG | 0.605229848 | 3.48E-112 | positive |
| BRAF | MIR583HG | 0.477452642 | 1.92E-64 | positive |
| TSC1 | AL354733.3 | 0.476965644 | 2.68E-64 | positive |
| MAPK8 | AL354733.3 | 0.416576702 | 5.96E-48 | positive |
| OTULIN | AL354733.3 | 0.495326469 | 5.90E-70 | positive |
| CYLD | AL354733.3 | 0.49191532 | 7.05E-69 | positive |
| MAP3K7 | AL354733.3 | 0.410631565 | 1.63E-46 | positive |
| DIABLO | AL354733.3 | 0.666979374 | 3.71E-144 | positive |
| CFLAR | AL354733.3 | 0.468515873 | 8.28E-62 | positive |
| BRAF | AL354733.3 | 0.653729756 | 1.16E-136 | positive |
| ATRX | AL354733.3 | 0.52993786 | 1.33E-81 | positive |
| RNF31 | AL354733.3 | 0.439300581 | 1.02E-53 | positive |
| MAPK8 | LANCL1-AS1 | 0.426493746 | 2.05E-50 | positive |
| IPMK | LANCL1-AS1 | 0.412105957 | 7.23E-47 | positive |
| OTULIN | LANCL1-AS1 | 0.420225556 | 7.56E-49 | positive |
| CYLD | LANCL1-AS1 | 0.480384471 | 2.52E-65 | positive |
| DIABLO | LANCL1-AS1 | 0.69901526 | 5.50E-164 | positive |
| CFLAR | LANCL1-AS1 | 0.419850641 | 9.36E-49 | positive |
| BRAF | LANCL1-AS1 | 0.709897529 | 2.45E-171 | positive |
| ATRX | LANCL1-AS1 | 0.499759185 | 2.25E-71 | positive |
| MAP3K7 | LINC02175 | 0.409331073 | 3.34E-46 | positive |
| DIABLO | LINC02175 | 0.439930605 | 6.94E-54 | positive |
| BRAF | LINC02175 | 0.443336436 | 8.63E-55 | positive |
| OTULIN | AC007878.1 | 0.4498615 | 1.49E-56 | positive |
| CYLD | AC007878.1 | 0.426871951 | 1.65E-50 | positive |
| DIABLO | AC007878.1 | 0.774386284 | 4.27E-223 | positive |
| BRAF | AC007878.1 | 0.689934756 | 4.20E-158 | positive |
| ATRX | AC007878.1 | 0.402161663 | 1.63E-44 | positive |
| DIABLO | SKAP1-AS1 | 0.517772131 | 2.35E-77 | positive |
| BRAF | SKAP1-AS1 | 0.425183332 | 4.39E-50 | positive |
| ATRX | SKAP1-AS1 | 0.456286741 | 2.51E-58 | positive |
| DIABLO | AP003550.1 | 0.600659224 | 4.31E-110 | positive |
| BRAF | AP003550.1 | 0.48898536 | 5.81E-68 | positive |
| DIABLO | LINC00852 | 0.564891835 | 8.00E-95 | positive |
| BRAF | LINC00852 | 0.482822446 | 4.58E-66 | positive |
| DIABLO | AC008937.1 | 0.465762001 | 5.18E-61 | positive |
| DIABLO | AL713852.1 | 0.676775516 | 5.94E-150 | positive |
| BRAF | AL713852.1 | 0.561693009 | 1.51E-93 | positive |
| TSC1 | AC107375.1 | 0.440390572 | 5.24E-54 | positive |
| BCL2 | LINC00487 | 0.401104828 | 2.86E-44 | positive |
| BCL2L11 | LINC00487 | 0.454793622 | 6.54E-58 | positive |
| DIABLO | AL512283.2 | 0.478447087 | 9.64E-65 | positive |
| OTULIN | ALKBH3-AS1 | 0.482417431 | 6.08E-66 | positive |
| CYLD | ALKBH3-AS1 | 0.434781094 | 1.55E-52 | positive |
| DIABLO | ALKBH3-AS1 | 0.735589489 | 3.99E-190 | positive |
| BRAF | ALKBH3-AS1 | 0.654567561 | 4.00E-137 | positive |
| ATRX | ALKBH3-AS1 | 0.416020723 | 8.15E-48 | positive |
| OTULIN | GHRLOS | 0.405436359 | 2.79E-45 | positive |
| CYLD | GHRLOS | 0.456488494 | 2.21E-58 | positive |
| DIABLO | GHRLOS | 0.738244485 | 3.41E-192 | positive |
| CFLAR | GHRLOS | 0.520968667 | 1.87E-78 | positive |
| BRAF | GHRLOS | 0.677395315 | 2.51E-150 | positive |
| ATRX | GHRLOS | 0.547526601 | 4.63E-88 | positive |
| DIABLO | DGCR11 | 0.405143528 | 3.27E-45 | positive |
| BRAF | DGCR11 | 0.401990174 | 1.78E-44 | positive |
| PANX1 | LINC01615 | 0.424351141 | 7.10E-50 | positive |
| HDAC9 | LINC02099 | 0.541890276 | 5.98E-86 | positive |
| CYLD | AC144521.1 | 0.4023657 | 1.46E-44 | positive |
| DIABLO | AC144521.1 | 0.666450895 | 7.51E-144 | positive |
| BRAF | AC144521.1 | 0.622670251 | 1.72E-120 | positive |
| DIABLO | AP001893.1 | 0.492586388 | 4.34E-69 | positive |
| BRAF | AP001893.1 | 0.492615764 | 4.25E-69 | positive |
| TSC1 | LINC00339 | 0.438604661 | 1.55E-53 | positive |
| DIABLO | LINC00339 | 0.480331256 | 2.61E-65 | positive |
| BRAF | LINC00339 | 0.440652537 | 4.47E-54 | positive |
| DIABLO | AC092902.4 | 0.628960526 | 1.29E-123 | positive |
| BRAF | AC092902.4 | 0.575376991 | 4.18E-99 | positive |
| ATRX | AC092902.4 | 0.461400194 | 9.14E-60 | positive |
| CD40 | AL354833.2 | 0.685294146 | 3.52E-155 | positive |
| DIABLO | AC004846.2 | 0.520131572 | 3.63E-78 | positive |
| BRAF | AC004846.2 | 0.450949275 | 7.51E-57 | positive |
| ATRX | AC004846.2 | 0.414550348 | 1.86E-47 | positive |
| TSC1 | AC009120.3 | 0.447185591 | 7.96E-56 | positive |
| CYLD | AC009120.3 | 0.471728827 | 9.53E-63 | positive |
| DIABLO | AC009120.3 | 0.571838985 | 1.21E-97 | positive |
| CFLAR | AC009120.3 | 0.51151863 | 3.08E-75 | positive |
| BRAF | AC009120.3 | 0.559828235 | 8.25E-93 | positive |
| ATRX | AC009120.3 | 0.554122026 | 1.39E-90 | positive |
| RNF31 | AC009120.3 | 0.464792949 | 9.84E-61 | positive |
| MAPK8 | AL049780.2 | 0.408817659 | 4.42E-46 | positive |
| OTULIN | AL049780.2 | 0.454620309 | 7.30E-58 | positive |
| CYLD | AL049780.2 | 0.414983916 | 1.46E-47 | positive |
| DIABLO | AL049780.2 | 0.721200074 | 2.43E-179 | positive |
| BRAF | AL049780.2 | 0.649237284 | 3.33E-134 | positive |
| ATRX | AL049780.2 | 0.430138677 | 2.43E-51 | positive |
| RIPK3 | HOXA11-AS | 0.426847634 | 1.67E-50 | positive |
| STUB1 | HOXA11-AS | 0.447445252 | 6.77E-56 | positive |
| MPG | HOXA11-AS | 0.492578746 | 4.36E-69 | positive |
| OTULIN | AL139041.1 | 0.427137439 | 1.41E-50 | positive |
| DIABLO | AL139041.1 | 0.659316506 | 8.94E-140 | positive |
| BRAF | AL139041.1 | 0.606655332 | 7.62E-113 | positive |
| DIABLO | LINC02828 | 0.410299536 | 1.96E-46 | positive |
| DIABLO | AP000873.3 | 0.576502315 | 1.42E-99 | positive |
| BRAF | AP000873.3 | 0.547166734 | 6.33E-88 | positive |
| DIABLO | AC021422.1 | 0.569024118 | 1.71E-96 | positive |
| BRAF | AC021422.1 | 0.466358351 | 3.49E-61 | positive |
| AXL | AL133482.1 | 0.419619018 | 1.07E-48 | positive |
| OTULIN | AC110603.1 | 0.414365654 | 2.06E-47 | positive |
| CYLD | AC110603.1 | 0.422747585 | 1.79E-49 | positive |
| DIABLO | AC110603.1 | 0.68963741 | 6.50E-158 | positive |
| BRAF | AC110603.1 | 0.640310929 | 1.91E-129 | positive |
| BCL2 | LINC02202 | 0.463864066 | 1.82E-60 | positive |
| BCL2L11 | LINC02202 | 0.43164229 | 1.00E-51 | positive |
| DIABLO | KIRREL1-IT1 | 0.608492995 | 1.06E-113 | positive |
| BRAF | KIRREL1-IT1 | 0.559732697 | 9.00E-93 | positive |
| ATRX | KIRREL1-IT1 | 0.474946529 | 1.07E-63 | positive |
| TSC1 | AC009107.2 | 0.464819775 | 9.66E-61 | positive |
| CYLD | AC009107.2 | 0.427864547 | 9.23E-51 | positive |
| DIABLO | AC009107.2 | 0.478373985 | 1.01E-64 | positive |
| BRAF | AC009107.2 | 0.473827187 | 2.30E-63 | positive |
| DIABLO | AJ239328.1 | 0.517737452 | 2.41E-77 | positive |
| DIABLO | AC119403.1 | 0.455616273 | 3.86E-58 | positive |
| BRAF | AC119403.1 | 0.435067359 | 1.30E-52 | positive |
| RNF31 | AC073548.2 | 0.412896724 | 4.66E-47 | positive |
| MAPK8 | AL049840.6 | 0.419963681 | 8.78E-49 | positive |
| OTULIN | AL049840.6 | 0.514536911 | 2.96E-76 | positive |
| CYLD | AL049840.6 | 0.495526891 | 5.10E-70 | positive |
| MAP3K7 | AL049840.6 | 0.415468435 | 1.11E-47 | positive |
| DIABLO | AL049840.6 | 0.77206809 | 6.06E-221 | positive |
| CFLAR | AL049840.6 | 0.443849709 | 6.29E-55 | positive |
| BRAF | AL049840.6 | 0.720511136 | 7.66E-179 | positive |
| ATRX | AL049840.6 | 0.443123881 | 9.84E-55 | positive |
| TSC1 | AC096992.2 | 0.410640945 | 1.62E-46 | positive |
| OTULIN | AC096992.2 | 0.45414791 | 9.87E-58 | positive |
| CYLD | AC096992.2 | 0.494315679 | 1.23E-69 | positive |
| MAP3K7 | AC096992.2 | 0.425925904 | 2.85E-50 | positive |
| DIABLO | AC096992.2 | 0.475850761 | 5.76E-64 | positive |
| BRAF | AC096992.2 | 0.559386924 | 1.23E-92 | positive |
| ATRX | AC096992.2 | 0.540281931 | 2.35E-85 | positive |
| DIABLO | AC010201.3 | 0.70774224 | 7.45E-170 | positive |
| BRAF | AC010201.3 | 0.512091827 | 1.98E-75 | positive |
| OTULIN | MED4-AS1 | 0.448442595 | 3.63E-56 | positive |
| DIABLO | MED4-AS1 | 0.697244248 | 8.04E-163 | positive |
| BRAF | MED4-AS1 | 0.571100019 | 2.43E-97 | positive |
| DIABLO | Z69666.1 | 0.541704567 | 7.00E-86 | positive |
| BRAF | Z69666.1 | 0.449629295 | 1.73E-56 | positive |
| ATRX | Z69666.1 | 0.51263785 | 1.30E-75 | positive |
| DIABLO | AC010260.1 | 0.658725023 | 1.92E-139 | positive |
| BRAF | AC010260.1 | 0.617050829 | 9.31E-118 | positive |
| ATRX | AC010260.1 | 0.452561946 | 2.71E-57 | positive |
| DIABLO | AC022154.1 | 0.566682469 | 1.52E-95 | positive |
| BRAF | AC022154.1 | 0.517838007 | 2.23E-77 | positive |
| ATRX | AC022154.1 | 0.453548595 | 1.45E-57 | positive |
| TSC1 | AC068790.4 | 0.42206324 | 2.65E-49 | positive |
| CYLD | AC068790.4 | 0.429238653 | 4.13E-51 | positive |
| DIABLO | AC068790.4 | 0.738843299 | 1.16E-192 | positive |
| CFLAR | AC068790.4 | 0.483514574 | 2.82E-66 | positive |
| BRAF | AC068790.4 | 0.66819024 | 7.33E-145 | positive |
| ATRX | AC068790.4 | 0.542167208 | 4.72E-86 | positive |
| OTULIN | AC099845.1 | 0.40375278 | 6.92E-45 | positive |
| DIABLO | AC099845.1 | 0.714215851 | 2.38E-174 | positive |
| BRAF | AC099845.1 | 0.57032395 | 5.06E-97 | positive |
| DIABLO | AC083806.3 | 0.552929731 | 4.02E-90 | positive |
| BRAF | AC083806.3 | 0.482246733 | 6.86E-66 | positive |
| ATRX | AC083806.3 | 0.442241782 | 1.69E-54 | positive |
| DIABLO | LINC02433 | 0.500975447 | 9.13E-72 | positive |
| BRAF | LINC02433 | 0.467034502 | 2.22E-61 | positive |
| DIABLO | AC048341.2 | 0.606372168 | 1.03E-112 | positive |
| CFLAR | AC048341.2 | 0.40672417 | 1.39E-45 | positive |
| BRAF | AC048341.2 | 0.558140893 | 3.80E-92 | positive |
| ATRX | AC048341.2 | 0.443107858 | 9.94E-55 | positive |
| DIABLO | AC079600.3 | 0.504321912 | 7.44E-73 | positive |
| BRAF | AC079600.3 | 0.41034144 | 1.92E-46 | positive |
| OTULIN | LINC02788 | 0.439518619 | 8.91E-54 | positive |
| DIABLO | LINC02788 | 0.661700634 | 4.00E-141 | positive |
| BRAF | LINC02788 | 0.631609115 | 5.90E-125 | positive |
| ATRX | LINC02788 | 0.411113767 | 1.25E-46 | positive |
| TSC1 | Z99127.3 | 0.400305967 | 4.38E-44 | positive |
| MAPK8 | Z99127.3 | 0.409172085 | 3.64E-46 | positive |
| IPMK | Z99127.3 | 0.409055839 | 3.88E-46 | positive |
| OTULIN | Z99127.3 | 0.452400448 | 3.00E-57 | positive |
| MAP3K7 | Z99127.3 | 0.420892865 | 5.17E-49 | positive |
| DIABLO | Z99127.3 | 0.628081883 | 3.55E-123 | positive |
| BRAF | Z99127.3 | 0.628440615 | 2.34E-123 | positive |
| ATRX | Z99127.3 | 0.413794643 | 2.83E-47 | positive |
| TLR3 | AC083837.1 | 0.414290523 | 2.15E-47 | positive |
| CFLAR | AC083837.1 | 0.46493477 | 8.96E-61 | positive |
| OTULIN | AC027627.1 | 0.405236739 | 3.11E-45 | positive |
| CYLD | AC027627.1 | 0.418056219 | 2.59E-48 | positive |
| DIABLO | AC027627.1 | 0.69018696 | 2.91E-158 | positive |
| BRAF | AC027627.1 | 0.610447498 | 1.29E-114 | positive |
| ATRX | AC027627.1 | 0.43583808 | 8.22E-53 | positive |
| DIABLO | AL135999.2 | 0.515813339 | 1.09E-76 | positive |
| RNF31 | AL135999.2 | 0.429425483 | 3.70E-51 | positive |
| DIABLO | NSMCE1-DT | 0.515730553 | 1.17E-76 | positive |
| BRAF | NSMCE1-DT | 0.432469538 | 6.13E-52 | positive |
| ATRX | NSMCE1-DT | 0.401788857 | 1.99E-44 | positive |
| DIABLO | SAMD12-AS1 | 0.58571481 | 1.76E-103 | positive |
| BRAF | SAMD12-AS1 | 0.555927201 | 2.78E-91 | positive |
| DIABLO | ASTN2-AS1 | 0.726796065 | 1.88E-183 | positive |
| BRAF | ASTN2-AS1 | 0.622826746 | 1.44E-120 | positive |
| MAPK8 | AC022137.3 | 0.421716465 | 3.23E-49 | positive |
| OTULIN | AC022137.3 | 0.486238352 | 4.11E-67 | positive |
| CYLD | AC022137.3 | 0.409320777 | 3.36E-46 | positive |
| DIABLO | AC022137.3 | 0.763777472 | 1.88E-213 | positive |
| CFLAR | AC022137.3 | 0.40873965 | 4.62E-46 | positive |
| BRAF | AC022137.3 | 0.696493878 | 2.49E-162 | positive |
| ATRX | AC022137.3 | 0.441691756 | 2.37E-54 | positive |
| DIABLO | AP005059.1 | 0.516525419 | 6.26E-77 | positive |
| BRAF | AP005059.1 | 0.43901697 | 1.21E-53 | positive |
| DIABLO | AL158212.2 | 0.562077909 | 1.06E-93 | positive |
| BRAF | AL158212.2 | 0.436853613 | 4.46E-53 | positive |
| MAPK8 | AC103739.1 | 0.403808401 | 6.72E-45 | positive |
| CYLD | AC103739.1 | 0.429300053 | 3.98E-51 | positive |
| DIABLO | AC103739.1 | 0.73152326 | 5.24E-187 | positive |
| CFLAR | AC103739.1 | 0.445988744 | 1.68E-55 | positive |
| BRAF | AC103739.1 | 0.658508634 | 2.54E-139 | positive |
| ATRX | AC103739.1 | 0.47587805 | 5.65E-64 | positive |
| DIABLO | AL024497.2 | 0.499389156 | 2.97E-71 | positive |
| BRAF | AL024497.2 | 0.500840192 | 1.01E-71 | positive |
| OTULIN | AC020978.3 | 0.464430982 | 1.25E-60 | positive |
| CYLD | AC020978.3 | 0.451872674 | 4.19E-57 | positive |
| DIABLO | AC020978.3 | 0.758883467 | 3.59E-209 | positive |
| BRAF | AC020978.3 | 0.68348078 | 4.73E-154 | positive |
| ATRX | AC020978.3 | 0.463649217 | 2.09E-60 | positive |
| MAPK8 | AP000787.1 | 0.402524577 | 1.34E-44 | positive |
| OTULIN | AP000787.1 | 0.408921475 | 4.18E-46 | positive |
| CYLD | AP000787.1 | 0.459079952 | 4.14E-59 | positive |
| DIABLO | AP000787.1 | 0.665151927 | 4.23E-143 | positive |
| CFLAR | AP000787.1 | 0.406054152 | 2.00E-45 | positive |
| BRAF | AP000787.1 | 0.650492406 | 6.91E-135 | positive |
| ATRX | AP000787.1 | 0.491538068 | 9.26E-69 | positive |
| TSC1 | AC008543.1 | 0.433250041 | 3.86E-52 | positive |
| OTULIN | AC008543.1 | 0.421561465 | 3.53E-49 | positive |
| MAP3K7 | AC008543.1 | 0.410377599 | 1.88E-46 | positive |
| DIABLO | AC008543.1 | 0.69299005 | 4.66E-160 | positive |
| BRAF | AC008543.1 | 0.596832129 | 2.29E-108 | positive |
| ATRX | AC008543.1 | 0.432039445 | 7.92E-52 | positive |
| TSC1 | AL512770.1 | 0.416828425 | 5.17E-48 | positive |
| DIABLO | AL512770.1 | 0.405324636 | 2.96E-45 | positive |
| RNF31 | AL512770.1 | 0.508697503 | 2.69E-74 | positive |
| DIABLO | CARMN | 0.41223399 | 6.73E-47 | positive |
| CFLAR | CARMN | 0.407536543 | 8.91E-46 | positive |
| BRAF | CARMN | 0.427749825 | 9.87E-51 | positive |
| KLF9 | CARMN | 0.560185728 | 5.96E-93 | positive |
| CDKN2A | AL449423.1 | 0.545900756 | 1.90E-87 | positive |
| DIABLO | AC022819.1 | 0.640147476 | 2.33E-129 | positive |
| CFLAR | AC022819.1 | 0.434570669 | 1.76E-52 | positive |
| BRAF | AC022819.1 | 0.569628558 | 9.72E-97 | positive |
| ATRX | AC022819.1 | 0.56273258 | 5.83E-94 | positive |
| RNF31 | AC022819.1 | 0.482140022 | 7.39E-66 | positive |
| TSC1 | AL031320.2 | 0.425602725 | 3.44E-50 | positive |
| MAPK8 | AL031320.2 | 0.448651724 | 3.19E-56 | positive |
| OTULIN | AL031320.2 | 0.409191598 | 3.60E-46 | positive |
| CYLD | AL031320.2 | 0.412979414 | 4.45E-47 | positive |
| DIABLO | AL031320.2 | 0.749259295 | 4.77E-201 | positive |
| CFLAR | AL031320.2 | 0.481808207 | 9.32E-66 | positive |
| BRAF | AL031320.2 | 0.69789766 | 2.99E-163 | positive |
| ATRX | AL031320.2 | 0.53168208 | 3.15E-82 | positive |
| KLF9 | AC005180.1 | 0.568637418 | 2.46E-96 | positive |
| DIABLO | AC127526.1 | 0.584636005 | 5.12E-103 | positive |
| BRAF | AC127526.1 | 0.531135773 | 4.95E-82 | positive |
| ATRX | AC127526.1 | 0.46019047 | 2.01E-59 | positive |
| CYLD | AL365318.1 | 0.405364843 | 2.90E-45 | positive |
| DIABLO | AL365318.1 | 0.71179852 | 1.18E-172 | positive |
| BRAF | AL365318.1 | 0.612072435 | 2.21E-115 | positive |
| ATRX | AL365318.1 | 0.431238442 | 1.27E-51 | positive |
| DIABLO | AL034428.1 | 0.598587666 | 3.73E-109 | positive |
| BRAF | AL034428.1 | 0.524892645 | 8.04E-80 | positive |
| MYC | FAM222A-AS1 | 0.641187848 | 6.62E-130 | positive |
| MAPK8 | AL158834.2 | 0.411339057 | 1.10E-46 | positive |
| OTULIN | AL158834.2 | 0.443067064 | 1.02E-54 | positive |
| CYLD | AL158834.2 | 0.403858073 | 6.54E-45 | positive |
| MAP3K7 | AL158834.2 | 0.404321999 | 5.09E-45 | positive |
| DIABLO | AL158834.2 | 0.746027734 | 2.11E-198 | positive |
| BRAF | AL158834.2 | 0.633964026 | 3.72E-126 | positive |
| RNF31 | AL132780.2 | 0.457530167 | 1.13E-58 | positive |
| MAP3K7 | LMCD1-AS1 | 0.406723954 | 1.39E-45 | positive |
| DIABLO | LMCD1-AS1 | 0.522956358 | 3.81E-79 | positive |
| BRAF | LMCD1-AS1 | 0.55592247 | 2.79E-91 | positive |
| ATRX | LMCD1-AS1 | 0.480519904 | 2.29E-65 | positive |
| DIABLO | AL360270.3 | 0.416051734 | 8.01E-48 | positive |
| SPATA2 | AC010761.4 | 0.4251052 | 4.59E-50 | positive |
| TSC1 | AC091057.1 | 0.481547273 | 1.12E-65 | positive |
| DNMT1 | AC091057.1 | 0.414351184 | 2.07E-47 | positive |
| TARDBP | AC091057.1 | 0.424659368 | 5.94E-50 | positive |
| HDAC9 | NAALADL2-AS2 | 0.475525196 | 7.20E-64 | positive |
| DIABLO | AC008771.1 | 0.736128088 | 1.53E-190 | positive |
| BRAF | AC008771.1 | 0.632632286 | 1.78E-125 | positive |
| ATRX | AC008771.1 | 0.461914626 | 6.53E-60 | positive |
| DIABLO | AC083900.1 | 0.483014126 | 4.00E-66 | positive |
| BRAF | AC083900.1 | 0.417664751 | 3.23E-48 | positive |
| TSC1 | AL590652.1 | 0.419489065 | 1.15E-48 | positive |
| MAP3K7 | AL590652.1 | 0.412320533 | 6.42E-47 | positive |
| DIABLO | AL590652.1 | 0.454940869 | 5.95E-58 | positive |
| BRAF | AL590652.1 | 0.490230052 | 2.38E-68 | positive |
| ATRX | AL590652.1 | 0.519149934 | 7.91E-78 | positive |
| EGFR | AC006460.1 | 0.461459781 | 8.79E-60 | positive |
| CYLD | LRRK2-DT | 0.52629201 | 2.59E-80 | positive |
| CFLAR | LRRK2-DT | 0.408925079 | 4.17E-46 | positive |
| BRAF | LRRK2-DT | 0.419110703 | 1.42E-48 | positive |
| BCL2 | LRRK2-DT | 0.43083188 | 1.62E-51 | positive |
| BACH2 | LRRK2-DT | 0.57360976 | 2.26E-98 | positive |
| KLF9 | LRRK2-DT | 0.483034963 | 3.94E-66 | positive |
| OTULIN | AC016747.3 | 0.425388327 | 3.90E-50 | positive |
| CYLD | AC016747.3 | 0.491108536 | 1.26E-68 | positive |
| DIABLO | AC016747.3 | 0.787467602 | 9.67E-236 | positive |
| CFLAR | AC016747.3 | 0.5155451 | 1.35E-76 | positive |
| BRAF | AC016747.3 | 0.720263378 | 1.16E-178 | positive |
| ATRX | AC016747.3 | 0.606716856 | 7.14E-113 | positive |
| RNF31 | AC016747.3 | 0.429495201 | 3.55E-51 | positive |
| DIABLO | AL451074.5 | 0.4084164 | 5.51E-46 | positive |
| OTULIN | ABALON | 0.431235845 | 1.27E-51 | positive |
| DIABLO | ABALON | 0.771711297 | 1.29E-220 | positive |
| BRAF | ABALON | 0.641743515 | 3.37E-130 | positive |
| BCL2 | AC015819.1 | 0.461889879 | 6.64E-60 | positive |
| CD40 | AC015819.1 | 0.487579981 | 1.58E-67 | positive |
| BCL2L11 | AC015819.1 | 0.499376 | 3.00E-71 | positive |
| PANX1 | AP000759.1 | 0.468986313 | 6.04E-62 | positive |
| CYLD | AP000759.1 | 0.417234828 | 4.12E-48 | positive |
| ATRX | AP000759.1 | 0.418628104 | 1.87E-48 | positive |
| DIABLO | AC092617.1 | 0.508608014 | 2.88E-74 | positive |
| DIABLO | AC009948.2 | 0.536341094 | 6.54E-84 | positive |
| BRAF | AC009948.2 | 0.505364822 | 3.39E-73 | positive |
| DIABLO | AL358072.1 | 0.468829461 | 6.71E-62 | positive |
| BRAF | AL358072.1 | 0.441699162 | 2.36E-54 | positive |
| DIABLO | LINC02595 | 0.562108685 | 1.03E-93 | positive |
| BRAF | LINC02595 | 0.496855087 | 1.93E-70 | positive |
| ATRX | LINC02595 | 0.474943868 | 1.07E-63 | positive |
| TSC1 | AL080276.2 | 0.425169377 | 4.43E-50 | positive |
| MAPK8 | AL080276.2 | 0.464000156 | 1.66E-60 | positive |
| IPMK | AL080276.2 | 0.405603772 | 2.55E-45 | positive |
| OTULIN | AL080276.2 | 0.552362005 | 6.64E-90 | positive |
| CYLD | AL080276.2 | 0.46338033 | 2.50E-60 | positive |
| MAP3K7 | AL080276.2 | 0.492093782 | 6.20E-69 | positive |
| DIABLO | AL080276.2 | 0.769763269 | 7.89E-219 | positive |
| CFLAR | AL080276.2 | 0.434290055 | 2.08E-52 | positive |
| BRAF | AL080276.2 | 0.72298046 | 1.22E-180 | positive |
| ATRX | AL080276.2 | 0.481091512 | 1.54E-65 | positive |
| DIABLO | FARSA-AS1 | 0.590670685 | 1.23E-105 | positive |
| BRAF | FARSA-AS1 | 0.491214956 | 1.17E-68 | positive |
| ATRX | FARSA-AS1 | 0.446394041 | 1.30E-55 | positive |
| RNF31 | FARSA-AS1 | 0.409083978 | 3.82E-46 | positive |
| MAPK8 | AL109936.9 | 0.425713719 | 3.23E-50 | positive |
| OTULIN | AL109936.9 | 0.468618057 | 7.73E-62 | positive |
| CYLD | AL109936.9 | 0.440552607 | 4.75E-54 | positive |
| DIABLO | AL109936.9 | 0.732505141 | 9.36E-188 | positive |
| BRAF | AL109936.9 | 0.68237139 | 2.29E-153 | positive |
| ATRX | AL109936.9 | 0.465899733 | 4.73E-61 | positive |
| OTULIN | AL031667.3 | 0.493732706 | 1.89E-69 | positive |
| CYLD | AL031667.3 | 0.401020618 | 2.99E-44 | positive |
| MAP3K7 | AL031667.3 | 0.42801614 | 8.45E-51 | positive |
| DIABLO | AL031667.3 | 0.686351964 | 7.68E-156 | positive |
| BRAF | AL031667.3 | 0.666484449 | 7.18E-144 | positive |
| ATRX | AL031667.3 | 0.493671418 | 1.97E-69 | positive |
| DIABLO | AC133106.1 | 0.575299102 | 4.50E-99 | positive |
| BRAF | AC133106.1 | 0.547293341 | 5.67E-88 | positive |
| OTULIN | AC020661.2 | 0.458982743 | 4.41E-59 | positive |
| CYLD | AC020661.2 | 0.418520297 | 1.99E-48 | positive |
| DIABLO | AC020661.2 | 0.746564959 | 7.71E-199 | positive |
| BRAF | AC020661.2 | 0.662548065 | 1.32E-141 | positive |
| ATRX | AC020661.2 | 0.452291282 | 3.22E-57 | positive |
| IPMK | AC083799.1 | 0.40830203 | 5.86E-46 | positive |
| OTULIN | AC083799.1 | 0.430630415 | 1.82E-51 | positive |
| MAP3K7 | AC083799.1 | 0.466718102 | 2.75E-61 | positive |
| HAT1 | AC083799.1 | 0.406373022 | 1.68E-45 | positive |
| TARDBP | AC083799.1 | 0.425507266 | 3.64E-50 | positive |
| MAPK8 | AC131159.1 | 0.488438124 | 8.59E-68 | positive |
| IPMK | AC131159.1 | 0.41600517 | 8.22E-48 | positive |
| OTULIN | AC131159.1 | 0.515930435 | 9.98E-77 | positive |
| CYLD | AC131159.1 | 0.429248981 | 4.10E-51 | positive |
| MAP3K7 | AC131159.1 | 0.401627303 | 2.16E-44 | positive |
| DIABLO | AC131159.1 | 0.70498975 | 5.58E-168 | positive |
| CFLAR | AC131159.1 | 0.407511481 | 9.03E-46 | positive |
| BRAF | AC131159.1 | 0.627298291 | 8.75E-123 | positive |
| ATRX | AC131159.1 | 0.529103009 | 2.63E-81 | positive |
| OTULIN | AC069542.1 | 0.483237326 | 3.42E-66 | positive |
| CYLD | AC069542.1 | 0.426074805 | 2.62E-50 | positive |
| DIABLO | AC069542.1 | 0.7481386 | 3.99E-200 | positive |
| BRAF | AC069542.1 | 0.657294999 | 1.22E-138 | positive |
| OTULIN | AL031710.2 | 0.439418308 | 9.47E-54 | positive |
| DIABLO | AL031710.2 | 0.589966989 | 2.51E-105 | positive |
| BRAF | AL031710.2 | 0.452115863 | 3.59E-57 | positive |
| DIABLO | AC015871.6 | 0.694055083 | 9.58E-161 | positive |
| BRAF | AC015871.6 | 0.612544118 | 1.32E-115 | positive |
| ATRX | AC015871.6 | 0.443178425 | 9.51E-55 | positive |
| DIABLO | AL158166.1 | 0.432576741 | 5.76E-52 | positive |
| BRAF | AL158166.1 | 0.412097468 | 7.26E-47 | positive |
| OTULIN | AL121845.4 | 0.452917376 | 2.16E-57 | positive |
| DIABLO | AL121845.4 | 0.742027301 | 3.50E-195 | positive |
| CFLAR | AL121845.4 | 0.433345723 | 3.64E-52 | positive |
| BRAF | AL121845.4 | 0.640490832 | 1.54E-129 | positive |
| ATRX | AL121845.4 | 0.45432273 | 8.83E-58 | positive |
| DIABLO | AC008760.1 | 0.41389918 | 2.67E-47 | positive |
| DIABLO | AC023825.2 | 0.592991503 | 1.17E-106 | positive |
| BRAF | AC023825.2 | 0.441694216 | 2.36E-54 | positive |
| MPG | AC115102.1 | -0.432678398 | 5.42E-52 | negative |
| ATRX | AC115102.1 | 0.497933802 | 8.70E-71 | positive |
| CD40 | LINC01353 | 0.652844963 | 3.57E-136 | positive |
| TSC1 | AC010168.2 | 0.5060419 | 2.03E-73 | positive |
| OTULIN | AC010168.2 | 0.435673235 | 9.07E-53 | positive |
| CYLD | AC010168.2 | 0.411373964 | 1.08E-46 | positive |
| MAP3K7 | AC010168.2 | 0.429789935 | 2.99E-51 | positive |
| DIABLO | AC010168.2 | 0.601273329 | 2.26E-110 | positive |
| BRAF | AC010168.2 | 0.544863217 | 4.66E-87 | positive |
| ATRX | AC010168.2 | 0.469950171 | 3.16E-62 | positive |
| MAPK8 | AC104316.1 | 0.409435261 | 3.15E-46 | positive |
| IPMK | AC104316.1 | 0.403121192 | 9.72E-45 | positive |
| OTULIN | AC104316.1 | 0.468516634 | 8.27E-62 | positive |
| CYLD | AC104316.1 | 0.450058318 | 1.32E-56 | positive |
| DIABLO | AC104316.1 | 0.794970938 | 2.09E-243 | positive |
| BRAF | AC104316.1 | 0.713911343 | 3.90E-174 | positive |
| ATRX | AC104316.1 | 0.423933715 | 9.04E-50 | positive |
| CYLD | AC112721.2 | 0.460895854 | 1.27E-59 | positive |
| DIABLO | AC087893.2 | 0.704429837 | 1.33E-167 | positive |
| BRAF | AC087893.2 | 0.602604112 | 5.60E-111 | positive |
| DIABLO | AC005050.3 | 0.471622524 | 1.02E-62 | positive |
| BRAF | AC005050.3 | 0.428937196 | 4.93E-51 | positive |
| DIABLO | AC130814.1 | 0.578845714 | 1.48E-100 | positive |
| BRAF | AC130814.1 | 0.547871627 | 3.43E-88 | positive |
| ATRX | AC130814.1 | 0.604933553 | 4.77E-112 | positive |
| RNF31 | AC130814.1 | 0.407999712 | 6.92E-46 | positive |
| MPG | AC012615.1 | 0.403242073 | 9.11E-45 | positive |
| DIABLO | MIR646HG | 0.437674797 | 2.72E-53 | positive |
| BRAF | MIR646HG | 0.424858224 | 5.30E-50 | positive |
| AXL | FAM225A | 0.412291034 | 6.52E-47 | positive |
| BCL2L11 | AC016168.2 | 0.448541228 | 3.41E-56 | positive |
| FADD | AP002336.2 | 0.522251734 | 6.71E-79 | positive |
| TNF | AP002336.2 | 0.40372037 | 7.04E-45 | positive |
| OTULIN | AP002336.2 | 0.484192635 | 1.75E-66 | positive |
| CYLD | AP002336.2 | 0.418822011 | 1.68E-48 | positive |
| DIABLO | AP002336.2 | 0.515507277 | 1.39E-76 | positive |
| BRAF | AP002336.2 | 0.499008629 | 3.93E-71 | positive |
| ATRX | AP002336.2 | 0.41302798 | 4.33E-47 | positive |
| MAPK8 | AC008966.2 | 0.429318546 | 3.94E-51 | positive |
| IPMK | AC008966.2 | 0.404640766 | 4.29E-45 | positive |
| OTULIN | AC008966.2 | 0.439700063 | 7.98E-54 | positive |
| CYLD | AC008966.2 | 0.442082261 | 1.86E-54 | positive |
| DIABLO | AC008966.2 | 0.726436428 | 3.47E-183 | positive |
| CFLAR | AC008966.2 | 0.439868825 | 7.20E-54 | positive |
| BRAF | AC008966.2 | 0.661071267 | 9.10E-141 | positive |
| ATRX | AC008966.2 | 0.474206856 | 1.77E-63 | positive |
| MAPK8 | ANKRD10-IT1 | 0.423524784 | 1.14E-49 | positive |
| OTULIN | ANKRD10-IT1 | 0.446274735 | 1.40E-55 | positive |
| MAP3K7 | ANKRD10-IT1 | 0.474756732 | 1.22E-63 | positive |
| DIABLO | ANKRD10-IT1 | 0.644269801 | 1.55E-131 | positive |
| CFLAR | ANKRD10-IT1 | 0.40976267 | 2.63E-46 | positive |
| BRAF | ANKRD10-IT1 | 0.60150109 | 1.78E-110 | positive |
| ATRX | ANKRD10-IT1 | 0.403460428 | 8.10E-45 | positive |
| DIABLO | AC010524.1 | 0.523265551 | 2.98E-79 | positive |
| BRAF | AC010524.1 | 0.461303849 | 9.74E-60 | positive |
| DIABLO | AC110760.1 | 0.688095066 | 6.16E-157 | positive |
| BRAF | AC110760.1 | 0.588275754 | 1.37E-104 | positive |
| CYLD | AC018653.4 | 0.401928322 | 1.84E-44 | positive |
| DIABLO | AC018653.4 | 0.599868576 | 9.83E-110 | positive |
| BRAF | AC018653.4 | 0.492005345 | 6.61E-69 | positive |
| ATRX | AC018653.4 | 0.469019368 | 5.91E-62 | positive |
| RNF31 | AC018653.4 | 0.454389889 | 8.46E-58 | positive |
| MAPK8 | AC069547.2 | 0.449826579 | 1.52E-56 | positive |
| IPMK | AC069547.2 | 0.461379944 | 9.27E-60 | positive |
| OTULIN | AC069547.2 | 0.439457892 | 9.24E-54 | positive |
| CYLD | AC069547.2 | 0.503745331 | 1.15E-72 | positive |
| DIABLO | AC069547.2 | 0.69424282 | 7.24E-161 | positive |
| CFLAR | AC069547.2 | 0.459107816 | 4.07E-59 | positive |
| BRAF | AC069547.2 | 0.659173459 | 1.08E-139 | positive |
| ATRX | AC069547.2 | 0.495325032 | 5.91E-70 | positive |
| CYLD | AC027796.5 | 0.401920774 | 1.85E-44 | positive |
| DIABLO | AC027796.5 | 0.62071205 | 1.56E-119 | positive |
| BRAF | AC027796.5 | 0.526415925 | 2.34E-80 | positive |
| ATRX | AC027796.5 | 0.491249795 | 1.14E-68 | positive |
| RNF31 | AC027796.5 | 0.425027057 | 4.80E-50 | positive |
| OTULIN | AC243773.2 | 0.416602236 | 5.88E-48 | positive |
| DIABLO | AC243773.2 | 0.763576731 | 2.84E-213 | positive |
| BRAF | AC243773.2 | 0.66091393 | 1.12E-140 | positive |
| CFLAR | DTX2P1-UPK3BP1-PMS2P11 | 0.420388037 | 6.89E-49 | positive |
| ATRX | DTX2P1-UPK3BP1-PMS2P11 | 0.492181496 | 5.82E-69 | positive |
| RNF31 | DTX2P1-UPK3BP1-PMS2P11 | 0.436455611 | 5.67E-53 | positive |
| DIABLO | AL031716.1 | 0.474164858 | 1.82E-63 | positive |
| BRAF | AL031716.1 | 0.446870943 | 9.69E-56 | positive |
| DIABLO | NUCB1-AS1 | 0.501860584 | 4.71E-72 | positive |
| BRAF | NUCB1-AS1 | 0.478440866 | 9.68E-65 | positive |
| ATRX | NUCB1-AS1 | 0.450602201 | 9.35E-57 | positive |
| CYLD | AL592295.3 | 0.498847543 | 4.43E-71 | positive |
| DIABLO | AL592295.3 | 0.679837311 | 8.23E-152 | positive |
| CFLAR | AL592295.3 | 0.549720873 | 6.81E-89 | positive |
| BRAF | AL592295.3 | 0.649165908 | 3.63E-134 | positive |
| ATRX | AL592295.3 | 0.594941226 | 1.60E-107 | positive |
| OTULIN | AC211433.1 | 0.466841138 | 2.53E-61 | positive |
| CYLD | AC211433.1 | 0.455862492 | 3.30E-58 | positive |
| DIABLO | AC211433.1 | 0.813673079 | 5.44E-264 | positive |
| CFLAR | AC211433.1 | 0.445190796 | 2.75E-55 | positive |
| BRAF | AC211433.1 | 0.725824113 | 9.88E-183 | positive |
| ATRX | AC211433.1 | 0.5088809 | 2.34E-74 | positive |
| DIABLO | AC022784.5 | 0.424765575 | 5.59E-50 | positive |
| BRAF | AC022784.5 | 0.425879698 | 2.93E-50 | positive |
| BNIP3 | AC103718.1 | 0.463833004 | 1.85E-60 | positive |
| DIABLO | AL133297.1 | 0.468063313 | 1.12E-61 | positive |
| DIABLO | AC021037.1 | 0.530433067 | 8.82E-82 | positive |
| BRAF | AC021037.1 | 0.509847088 | 1.11E-74 | positive |
| ATRX | AC021037.1 | 0.420012763 | 8.54E-49 | positive |
| RNF31 | AC087289.2 | 0.525690267 | 4.22E-80 | positive |
| DIABLO | AL021707.1 | 0.669785579 | 8.55E-146 | positive |
| BRAF | AL021707.1 | 0.576058997 | 2.17E-99 | positive |
| CYLD | AC010333.1 | 0.45965887 | 2.84E-59 | positive |
| DIABLO | AC010333.1 | 0.677781193 | 1.46E-150 | positive |
| CFLAR | AC010333.1 | 0.455059567 | 5.52E-58 | positive |
| BRAF | AC010333.1 | 0.614252212 | 2.04E-116 | positive |
| ATRX | AC010333.1 | 0.447525757 | 6.44E-56 | positive |
| DIABLO | AC097634.3 | 0.634742507 | 1.48E-126 | positive |
| CFLAR | AC097634.3 | 0.413124509 | 4.11E-47 | positive |
| BRAF | AC097634.3 | 0.558394667 | 3.02E-92 | positive |
| DIABLO | AC068792.1 | 0.471194107 | 1.37E-62 | positive |
| BRAF | AC068792.1 | 0.491804536 | 7.64E-69 | positive |
| ATRX | AC068792.1 | 0.474217741 | 1.76E-63 | positive |
| DIABLO | SPRY4-AS1 | 0.431146462 | 1.34E-51 | positive |
| DIABLO | PTPRJ-AS1 | 0.689544904 | 7.44E-158 | positive |
| BRAF | PTPRJ-AS1 | 0.554951574 | 6.65E-91 | positive |
| OTULIN | SND1-IT1 | 0.41528252 | 1.23E-47 | positive |
| CYLD | SND1-IT1 | 0.401323515 | 2.55E-44 | positive |
| DIABLO | SND1-IT1 | 0.768069894 | 2.72E-217 | positive |
| CFLAR | SND1-IT1 | 0.438881137 | 1.31E-53 | positive |
| BRAF | SND1-IT1 | 0.694030025 | 9.94E-161 | positive |
| ATRX | SND1-IT1 | 0.526206873 | 2.78E-80 | positive |
| DIABLO | AC008870.3 | 0.501686486 | 5.37E-72 | positive |
| BRAF | AC008870.3 | 0.436421254 | 5.79E-53 | positive |
| OTULIN | AL137782.1 | 0.412314597 | 6.44E-47 | positive |
| DIABLO | AL137782.1 | 0.712925783 | 1.92E-173 | positive |
| BRAF | AL137782.1 | 0.60571281 | 2.08E-112 | positive |
| TNFSF10 | AC068473.4 | 0.404187089 | 5.48E-45 | positive |
| OTULIN | HMGA2-AS1 | 0.44566119 | 2.05E-55 | positive |
| CYLD | HMGA2-AS1 | 0.424700247 | 5.80E-50 | positive |
| DIABLO | HMGA2-AS1 | 0.676043786 | 1.64E-149 | positive |
| BRAF | HMGA2-AS1 | 0.607984811 | 1.84E-113 | positive |
| ATRX | HMGA2-AS1 | 0.405838501 | 2.24E-45 | positive |
| DIABLO | ZFHX2-AS1 | 0.626948243 | 1.31E-122 | positive |
| BRAF | ZFHX2-AS1 | 0.485076939 | 9.36E-67 | positive |
| RNF31 | ZFHX2-AS1 | 0.49477972 | 8.80E-70 | positive |
| TSC1 | AL135791.1 | 0.406978902 | 1.21E-45 | positive |
| CYLD | AL512603.2 | 0.419280606 | 1.29E-48 | positive |
| DIABLO | AL512603.2 | 0.525052628 | 7.06E-80 | positive |
| BRAF | AL512603.2 | 0.538336919 | 1.22E-84 | positive |
| ATRX | AL512603.2 | 0.416749843 | 5.41E-48 | positive |
| ATRX | LINC02367 | 0.436670262 | 4.99E-53 | positive |
| DIABLO | AC005387.2 | 0.432230583 | 7.07E-52 | positive |
| RNF31 | AC005387.2 | 0.44608276 | 1.58E-55 | positive |
| DIABLO | AL390957.1 | 0.445885407 | 1.79E-55 | positive |
| BRAF | AL390957.1 | 0.448387663 | 3.76E-56 | positive |
| TSC1 | LAMTOR5-AS1 | 0.524431629 | 1.17E-79 | positive |
| MAPK8 | LAMTOR5-AS1 | 0.50943834 | 1.52E-74 | positive |
| IPMK | LAMTOR5-AS1 | 0.430499146 | 1.97E-51 | positive |
| OTULIN | LAMTOR5-AS1 | 0.527351117 | 1.10E-80 | positive |
| CYLD | LAMTOR5-AS1 | 0.428571033 | 6.11E-51 | positive |
| MAP3K7 | LAMTOR5-AS1 | 0.494959008 | 7.72E-70 | positive |
| DIABLO | LAMTOR5-AS1 | 0.703132001 | 9.98E-167 | positive |
| CFLAR | LAMTOR5-AS1 | 0.446295598 | 1.39E-55 | positive |
| BRAF | LAMTOR5-AS1 | 0.693757396 | 1.49E-160 | positive |
| ATRX | LAMTOR5-AS1 | 0.56086499 | 3.21E-93 | positive |
| DIABLO | AL355483.1 | 0.604435894 | 8.08E-112 | positive |
| BRAF | AL355483.1 | 0.454184349 | 9.65E-58 | positive |
| TSC1 | AC090510.2 | 0.416996093 | 4.71E-48 | positive |
| OTULIN | AC090510.2 | 0.414674502 | 1.73E-47 | positive |
| DIABLO | AC090510.2 | 0.673253587 | 7.64E-148 | positive |
| CFLAR | AC090510.2 | 0.41435555 | 2.07E-47 | positive |
| BRAF | AC090510.2 | 0.598684321 | 3.37E-109 | positive |
| ATRX | AC090510.2 | 0.431242678 | 1.27E-51 | positive |
| DIABLO | AC005746.3 | 0.602103538 | 9.47E-111 | positive |
| BRAF | AC005746.3 | 0.471576711 | 1.06E-62 | positive |
| CYLD | AC068389.3 | 0.401854624 | 1.92E-44 | positive |
| DIABLO | AC068389.3 | 0.741860074 | 4.75E-195 | positive |
| BRAF | AC068389.3 | 0.651232666 | 2.73E-135 | positive |
| ATRX | AC068389.3 | 0.413098083 | 4.17E-47 | positive |
| DIABLO | AL132639.3 | 0.420191728 | 7.71E-49 | positive |
| OTULIN | AL023584.2 | 0.422139817 | 2.53E-49 | positive |
| CYLD | AL023584.2 | 0.407485392 | 9.16E-46 | positive |
| DIABLO | AL023584.2 | 0.701837967 | 7.34E-166 | positive |
| BRAF | AL023584.2 | 0.619949716 | 3.68E-119 | positive |
| ATRX | AL023584.2 | 0.419141247 | 1.40E-48 | positive |
| CYLD | LINC01197 | 0.418620015 | 1.88E-48 | positive |
| CFLAR | LINC01197 | 0.439742685 | 7.78E-54 | positive |
| BACH2 | LINC01197 | 0.417858584 | 2.89E-48 | positive |
| KLF9 | LINC01197 | 0.568739467 | 2.24E-96 | positive |
| DIABLO | AC084398.2 | 0.568410578 | 3.04E-96 | positive |
| BRAF | AC084398.2 | 0.458459968 | 6.19E-59 | positive |
| DIABLO | AL109935.1 | 0.670209304 | 4.82E-146 | positive |
| BRAF | AL109935.1 | 0.556537571 | 1.61E-91 | positive |
| ATRX | AL109935.1 | 0.460804144 | 1.35E-59 | positive |
| OTULIN | AC090589.2 | 0.444084199 | 5.45E-55 | positive |
| DIABLO | AC090589.2 | 0.739475158 | 3.68E-193 | positive |
| BRAF | AC090589.2 | 0.609907776 | 2.31E-114 | positive |
| DIABLO | AC009908.1 | 0.403307635 | 8.79E-45 | positive |
| TSC1 | AL365330.1 | 0.444078209 | 5.47E-55 | positive |
| RNF31 | AL365330.1 | 0.445892751 | 1.78E-55 | positive |
| TSC1 | PABPC4-AS1 | 0.405198687 | 3.17E-45 | positive |
| MAPK8 | PABPC4-AS1 | 0.436387408 | 5.91E-53 | positive |
| IPMK | PABPC4-AS1 | 0.423732803 | 1.01E-49 | positive |
| OTULIN | PABPC4-AS1 | 0.487504442 | 1.67E-67 | positive |
| CYLD | PABPC4-AS1 | 0.421666516 | 3.32E-49 | positive |
| DIABLO | PABPC4-AS1 | 0.772662197 | 1.71E-221 | positive |
| BRAF | PABPC4-AS1 | 0.687531674 | 1.39E-156 | positive |
| ATRX | PABPC4-AS1 | 0.435183933 | 1.22E-52 | positive |
| OTULIN | AC244093.5 | 0.465406264 | 6.56E-61 | positive |
| CYLD | AC244093.5 | 0.419156416 | 1.39E-48 | positive |
| DIABLO | AC244093.5 | 0.778729734 | 3.37E-227 | positive |
| CFLAR | AC244093.5 | 0.406032716 | 2.02E-45 | positive |
| BRAF | AC244093.5 | 0.681526836 | 7.59E-153 | positive |
| ATRX | AC244093.5 | 0.452787201 | 2.35E-57 | positive |
| ATRX | AL117329.1 | 0.440937616 | 3.75E-54 | positive |
| RNF31 | AL117329.1 | 0.442071612 | 1.88E-54 | positive |
| DIABLO | AC234917.1 | 0.589076648 | 6.14E-105 | positive |
| BRAF | AC234917.1 | 0.503868166 | 1.05E-72 | positive |
| TSC1 | AC020915.1 | 0.414262967 | 2.18E-47 | positive |
| DIABLO | AC020915.1 | 0.647620237 | 2.49E-133 | positive |
| CFLAR | AC020915.1 | 0.485360902 | 7.66E-67 | positive |
| BRAF | AC020915.1 | 0.565383326 | 5.08E-95 | positive |
| ATRX | AC020915.1 | 0.536892874 | 4.12E-84 | positive |
| RNF31 | AC020915.1 | 0.429919901 | 2.77E-51 | positive |
| ATRX | AC087500.1 | 0.401390658 | 2.46E-44 | positive |
| RNF31 | AC087500.1 | 0.442089483 | 1.86E-54 | positive |
| TSC1 | AC060780.1 | 0.419335817 | 1.25E-48 | positive |
| CYLD | MIR29B2CHG | 0.419144308 | 1.40E-48 | positive |
| DIABLO | MIR29B2CHG | 0.744457544 | 3.93E-197 | positive |
| CFLAR | MIR29B2CHG | 0.457698824 | 1.01E-58 | positive |
| BRAF | MIR29B2CHG | 0.615950084 | 3.15E-117 | positive |
| DIABLO | AC018695.2 | 0.432099112 | 7.64E-52 | positive |
| BRAF | AC018695.2 | 0.400369111 | 4.23E-44 | positive |
| TSC1 | AL355488.1 | 0.459986868 | 2.30E-59 | positive |
| MAPK8 | AL355488.1 | 0.466152068 | 4.00E-61 | positive |
| IPMK | AL355488.1 | 0.405523956 | 2.66E-45 | positive |
| OTULIN | AL355488.1 | 0.53756001 | 2.35E-84 | positive |
| MAP3K7 | AL355488.1 | 0.463405534 | 2.46E-60 | positive |
| DIABLO | AL355488.1 | 0.65879876 | 1.75E-139 | positive |
| BRAF | AL355488.1 | 0.647512937 | 2.84E-133 | positive |
| ATRX | AL355488.1 | 0.474058195 | 1.96E-63 | positive |
| RNF31 | AL355488.1 | 0.401027649 | 2.98E-44 | positive |
| BRAF | AC139718.1 | 0.409060315 | 3.87E-46 | positive |
| TSC1 | PKD1P6-NPIPP1 | 0.400018232 | 5.10E-44 | positive |
| OTULIN | AL139286.2 | 0.406078145 | 1.97E-45 | positive |
| CYLD | AL139286.2 | 0.428333046 | 7.02E-51 | positive |
| DIABLO | AL139286.2 | 0.77514129 | 8.40E-224 | positive |
| CFLAR | AL139286.2 | 0.421607672 | 3.44E-49 | positive |
| BRAF | AL139286.2 | 0.693611849 | 1.85E-160 | positive |
| ATRX | AL139286.2 | 0.505841984 | 2.36E-73 | positive |
| DIABLO | AC107031.1 | 0.639099333 | 8.22E-129 | positive |
| BRAF | AC107031.1 | 0.591198989 | 7.23E-106 | positive |
| DIABLO | RPS6KA2-AS1 | 0.693919137 | 1.17E-160 | positive |
| BRAF | RPS6KA2-AS1 | 0.538869344 | 7.79E-85 | positive |
| OTULIN | AC012157.2 | 0.423550713 | 1.13E-49 | positive |
| CYLD | AC012157.2 | 0.427007068 | 1.52E-50 | positive |
| DIABLO | AC012157.2 | 0.821185749 | 6.32E-273 | positive |
| BRAF | AC012157.2 | 0.67439589 | 1.59E-148 | positive |
| DIABLO | AL358472.2 | 0.434665189 | 1.66E-52 | positive |
| DIABLO | LINC02886 | 0.515913797 | 1.01E-76 | positive |
| BRAF | LINC02886 | 0.450268913 | 1.15E-56 | positive |
| ATRX | LINC02886 | 0.427102124 | 1.44E-50 | positive |
| DNMT1 | CENATAC-DT | 0.488165624 | 1.04E-67 | positive |
| CYLD | AC027682.5 | 0.427242563 | 1.33E-50 | positive |
| DIABLO | AC027682.5 | 0.566261394 | 2.25E-95 | positive |
| BRAF | AC027682.5 | 0.457102896 | 1.49E-58 | positive |
| RNF31 | AC027682.5 | 0.401134932 | 2.82E-44 | positive |
| DIABLO | AL607028.1 | 0.691356666 | 5.21E-159 | positive |
| BRAF | AL607028.1 | 0.576592355 | 1.30E-99 | positive |
| BRAF | ARHGEF35-AS1 | 0.458854146 | 4.79E-59 | positive |
| ATRX | ARHGEF35-AS1 | 0.415995417 | 8.26E-48 | positive |
| STUB1 | AL590714.1 | 0.426141692 | 2.52E-50 | positive |
| MPG | AL590714.1 | 0.400071585 | 4.96E-44 | positive |
| DIABLO | AC005776.2 | 0.459521076 | 3.11E-59 | positive |
| CFLAR | AC005776.2 | 0.455264575 | 4.84E-58 | positive |
| BRAF | AC005776.2 | 0.46062656 | 1.51E-59 | positive |
| ATRX | AC005776.2 | 0.61863015 | 1.61E-118 | positive |
| RNF31 | AC005776.2 | 0.475737032 | 6.23E-64 | positive |
| MAPK8 | AC063965.2 | 0.401130885 | 2.82E-44 | positive |
| OTULIN | AC063965.2 | 0.476239452 | 4.41E-64 | positive |
| CYLD | AC063965.2 | 0.429817074 | 2.94E-51 | positive |
| DIABLO | AC063965.2 | 0.762727446 | 1.59E-212 | positive |
| BRAF | AC063965.2 | 0.678559474 | 4.94E-151 | positive |
| OTULIN | AC010542.5 | 0.411432896 | 1.05E-46 | positive |
| DIABLO | AC010542.5 | 0.64046589 | 1.58E-129 | positive |
| BRAF | AC010542.5 | 0.522139746 | 7.33E-79 | positive |
| OTULIN | AC110769.1 | 0.40246336 | 1.38E-44 | positive |
| CYLD | AC110769.1 | 0.419242494 | 1.32E-48 | positive |
| DIABLO | AC110769.1 | 0.712356324 | 4.80E-173 | positive |
| BRAF | AC110769.1 | 0.626172165 | 3.19E-122 | positive |
| KLF9 | HID1-AS1 | 0.514919514 | 2.20E-76 | positive |
| DIABLO | AC068888.2 | 0.66372974 | 2.78E-142 | positive |
| BRAF | AC068888.2 | 0.513317657 | 7.65E-76 | positive |
| BCL2 | AC009061.2 | 0.407433688 | 9.42E-46 | positive |
| OTULIN | AC109347.2 | 0.402788804 | 1.16E-44 | positive |
| BCL2 | AC109347.2 | 0.407005864 | 1.19E-45 | positive |
| CYLD | AC110048.2 | 0.40513126 | 3.29E-45 | positive |
| DIABLO | AC110048.2 | 0.738998185 | 8.74E-193 | positive |
| CFLAR | AC110048.2 | 0.436494112 | 5.54E-53 | positive |
| BRAF | AC110048.2 | 0.625972623 | 4.01E-122 | positive |
| ATRX | AC110048.2 | 0.455882004 | 3.26E-58 | positive |
| DIABLO | AC010280.3 | 0.510682802 | 5.86E-75 | positive |
| BRAF | AC010280.3 | 0.477338271 | 2.07E-64 | positive |
| TSC1 | Z80897.1 | 0.432816975 | 4.99E-52 | positive |
| CYLD | Z80897.1 | 0.441079703 | 3.44E-54 | positive |
| DIABLO | Z80897.1 | 0.471029386 | 1.53E-62 | positive |
| BRAF | Z80897.1 | 0.474024416 | 2.01E-63 | positive |
| DIABLO | AC078852.2 | 0.445139954 | 2.84E-55 | positive |
| DIABLO | AC243965.2 | 0.420384613 | 6.91E-49 | positive |
| BRAF | AC243965.2 | 0.494479338 | 1.10E-69 | positive |
| ATRX | AC243965.2 | 0.487548419 | 1.62E-67 | positive |
| RNF31 | AC243965.2 | 0.408122038 | 6.47E-46 | positive |
| TSC1 | FAM111A-DT | 0.419097301 | 1.44E-48 | positive |
| CASP8 | FAM111A-DT | 0.430778856 | 1.67E-51 | positive |
| MAP3K7 | FAM111A-DT | 0.401732881 | 2.05E-44 | positive |
| SIRT1 | FAM111A-DT | 0.413830975 | 2.77E-47 | positive |
| DIABLO | AC015853.3 | 0.745062767 | 1.28E-197 | positive |
| CFLAR | AC015853.3 | 0.428933378 | 4.94E-51 | positive |
| BRAF | AC015853.3 | 0.590139445 | 2.11E-105 | positive |
| ATRX | AC015853.3 | 0.452211469 | 3.38E-57 | positive |
| RNF31 | AC015853.3 | 0.407092908 | 1.13E-45 | positive |
| MAPK8 | AC073349.4 | 0.402012562 | 1.76E-44 | positive |
| IPMK | AC073349.4 | 0.408648875 | 4.85E-46 | positive |
| OTULIN | AC073349.4 | 0.456482457 | 2.21E-58 | positive |
| CYLD | AC073349.4 | 0.447282223 | 7.50E-56 | positive |
| MAP3K7 | AC073349.4 | 0.402766849 | 1.18E-44 | positive |
| DIABLO | AC073349.4 | 0.797810008 | 2.17E-246 | positive |
| CFLAR | AC073349.4 | 0.410555915 | 1.70E-46 | positive |
| BRAF | AC073349.4 | 0.723023115 | 1.14E-180 | positive |
| ATRX | AC073349.4 | 0.478597531 | 8.69E-65 | positive |
| FASLG | AL021978.1 | 0.55253625 | 5.69E-90 | positive |
| CYLD | AL021978.1 | 0.441620161 | 2.47E-54 | positive |
| TSC1 | AC245060.6 | 0.576717563 | 1.16E-99 | positive |
| OTULIN | AC245060.6 | 0.419734748 | 1.00E-48 | positive |
| CYLD | AC245060.6 | 0.469150489 | 5.41E-62 | positive |
| DIABLO | AC245060.6 | 0.488351563 | 9.13E-68 | positive |
| CFLAR | AC245060.6 | 0.454507539 | 7.85E-58 | positive |
| BRAF | AC245060.6 | 0.577004232 | 8.77E-100 | positive |
| ATRX | AC245060.6 | 0.600606195 | 4.55E-110 | positive |
| RNF31 | AC245060.6 | 0.509174867 | 1.86E-74 | positive |
| OTULIN | AL355312.2 | 0.417630402 | 3.29E-48 | positive |
| CYLD | AL355312.2 | 0.423698344 | 1.03E-49 | positive |
| MAP3K7 | AL355312.2 | 0.431387077 | 1.16E-51 | positive |
| DIABLO | AL355312.2 | 0.571966112 | 1.07E-97 | positive |
| BRAF | AL355312.2 | 0.552956306 | 3.92E-90 | positive |
| ATRX | AL355312.2 | 0.497435753 | 1.26E-70 | positive |
| MAPK8 | MAMDC2-AS1 | 0.40754582 | 8.86E-46 | positive |
| OTULIN | MAMDC2-AS1 | 0.400588427 | 3.77E-44 | positive |
| CYLD | MAMDC2-AS1 | 0.428652118 | 5.82E-51 | positive |
| DIABLO | MAMDC2-AS1 | 0.699340361 | 3.35E-164 | positive |
| CFLAR | MAMDC2-AS1 | 0.421617443 | 3.42E-49 | positive |
| BRAF | MAMDC2-AS1 | 0.65073961 | 5.07E-135 | positive |
| ATRX | MAMDC2-AS1 | 0.487457619 | 1.73E-67 | positive |
| SPATA2 | AL162595.1 | 0.445527293 | 2.23E-55 | positive |
| OTULIN | AC055764.2 | 0.451400115 | 5.65E-57 | positive |
| CYLD | AC055764.2 | 0.458798485 | 4.97E-59 | positive |
| DIABLO | AC055764.2 | 0.6035621 | 2.04E-111 | positive |
| BRAF | AC055764.2 | 0.571852144 | 1.20E-97 | positive |
| ATRX | AC055764.2 | 0.477056006 | 2.52E-64 | positive |
| DIABLO | RBFADN | 0.461063704 | 1.14E-59 | positive |
| BRAF | RBFADN | 0.413623741 | 3.11E-47 | positive |
| TSC1 | OGFR-AS1 | 0.424438355 | 6.75E-50 | positive |
| TSC1 | AL139011.1 | 0.494832589 | 8.47E-70 | positive |
| DIABLO | AL139011.1 | 0.409264404 | 3.46E-46 | positive |
| CFLAR | AL139011.1 | 0.409086901 | 3.82E-46 | positive |
| BRAF | AL139011.1 | 0.451333749 | 5.89E-57 | positive |
| ATRX | AL139011.1 | 0.472821873 | 4.55E-63 | positive |
| RNF31 | AL139011.1 | 0.488677812 | 7.23E-68 | positive |
| DIABLO | AC092436.3 | 0.72929987 | 2.51E-185 | positive |
| CFLAR | AC092436.3 | 0.449404528 | 1.99E-56 | positive |
| BRAF | AC092436.3 | 0.646826906 | 6.65E-133 | positive |
| ATRX | AC092436.3 | 0.476041144 | 5.06E-64 | positive |
| DIABLO | AC090541.1 | 0.544178337 | 8.40E-87 | positive |
| BRAF | AC090541.1 | 0.461373803 | 9.30E-60 | positive |
| DIABLO | AC069234.5 | 0.475660768 | 6.56E-64 | positive |
| RNF31 | AP001610.2 | 0.428173226 | 7.71E-51 | positive |
| DDX58 | AP001610.2 | 0.575292083 | 4.54E-99 | positive |
| OTULIN | AC022960.2 | 0.437942772 | 2.31E-53 | positive |
| CYLD | AC022960.2 | 0.420775963 | 5.53E-49 | positive |
| DIABLO | AC022960.2 | 0.752533825 | 9.06E-204 | positive |
| BRAF | AC022960.2 | 0.664143175 | 1.61E-142 | positive |
| DIABLO | AC020931.1 | 0.525651054 | 4.36E-80 | positive |
| CFLAR | AC020931.1 | 0.420259086 | 7.42E-49 | positive |
| BRAF | AC020931.1 | 0.415503012 | 1.09E-47 | positive |
| ATRX | AC020931.1 | 0.415204104 | 1.29E-47 | positive |
| RNF31 | AC020931.1 | 0.472480574 | 5.73E-63 | positive |
| OTULIN | AL136115.2 | 0.408193544 | 6.22E-46 | positive |
| CYLD | AL136115.2 | 0.42024892 | 7.46E-49 | positive |
| DIABLO | AL136115.2 | 0.724517911 | 9.11E-182 | positive |
| BRAF | AL136115.2 | 0.650053959 | 1.20E-134 | positive |
| DIABLO | FP325332.1 | 0.75179039 | 3.79E-203 | positive |
| CFLAR | FP325332.1 | 0.416004851 | 8.22E-48 | positive |
| BRAF | FP325332.1 | 0.633644491 | 5.41E-126 | positive |
| ATRX | FP325332.1 | 0.474069077 | 1.95E-63 | positive |
| DIABLO | AC092127.1 | 0.742487065 | 1.50E-195 | positive |
| BRAF | AC092127.1 | 0.57362893 | 2.22E-98 | positive |
| DIABLO | LINC02883 | 0.48257756 | 5.44E-66 | positive |
| CFLAR | LINC02883 | 0.414093666 | 2.39E-47 | positive |
| BRAF | LINC02883 | 0.402852752 | 1.12E-44 | positive |
| OTULIN | AC009269.2 | 0.417317725 | 3.93E-48 | positive |
| DIABLO | AC009269.2 | 0.711609799 | 1.59E-172 | positive |
| BRAF | AC009269.2 | 0.624542672 | 2.05E-121 | positive |
| OTULIN | AC002064.2 | 0.426612527 | 1.92E-50 | positive |
| CYLD | AC002064.2 | 0.414822624 | 1.59E-47 | positive |
| DIABLO | AC002064.2 | 0.696805157 | 1.56E-162 | positive |
| BRAF | AC002064.2 | 0.650348001 | 8.29E-135 | positive |
| MAPK8 | SBF2-AS1 | 0.41326959 | 3.79E-47 | positive |
| DIABLO | SBF2-AS1 | 0.448636905 | 3.22E-56 | positive |
| BRAF | SBF2-AS1 | 0.406968587 | 1.21E-45 | positive |
| IPMK | AP000240.1 | 0.401038896 | 2.96E-44 | positive |
| DIABLO | AP000240.1 | 0.589410465 | 4.39E-105 | positive |
| CFLAR | AP000240.1 | 0.440718501 | 4.29E-54 | positive |
| BRAF | AP000240.1 | 0.574411985 | 1.05E-98 | positive |
| MPG | AP000240.1 | -0.410196645 | 2.07E-46 | negative |
| ATRX | AP000240.1 | 0.630406837 | 2.40E-124 | positive |
| OTULIN | AC004672.1 | 0.465693385 | 5.42E-61 | positive |
| CYLD | AC004672.1 | 0.459792479 | 2.61E-59 | positive |
| DIABLO | AC004672.1 | 0.846685215 | 1.06E-306 | positive |
| BRAF | AC004672.1 | 0.706474463 | 5.47E-169 | positive |
| ATRX | AC004672.1 | 0.43771275 | 2.66E-53 | positive |
| MAPK8 | AC092718.5 | 0.439717286 | 7.90E-54 | positive |
| OTULIN | AC092718.5 | 0.40974108 | 2.66E-46 | positive |
| DIABLO | AC092718.5 | 0.563089207 | 4.20E-94 | positive |
| BRAF | AC092718.5 | 0.539887142 | 3.29E-85 | positive |
| MAPK8 | AL132656.2 | 0.427376087 | 1.23E-50 | positive |
| CYLD | AL132656.2 | 0.400199054 | 4.64E-44 | positive |
| DIABLO | AL132656.2 | 0.567071322 | 1.06E-95 | positive |
| CFLAR | AL132656.2 | 0.444152852 | 5.22E-55 | positive |
| BRAF | AL132656.2 | 0.575027252 | 5.84E-99 | positive |
| ATRX | AL132656.2 | 0.540774077 | 1.55E-85 | positive |
| DIABLO | AC098679.5 | 0.519095506 | 8.26E-78 | positive |
| BRAF | AC098679.5 | 0.462921937 | 3.37E-60 | positive |
| ATRX | AC098679.5 | 0.498744789 | 4.78E-71 | positive |
| DIABLO | LINC00677 | 0.428198081 | 7.60E-51 | positive |
| RNF31 | LINC00677 | 0.406435702 | 1.62E-45 | positive |
| CD40 | TP73-AS3 | 0.522594603 | 5.10E-79 | positive |
| DIABLO | AC025539.1 | 0.409694202 | 2.73E-46 | positive |
| KLF9 | MIR1-1HG-AS1 | 0.626809196 | 1.54E-122 | positive |
| DIABLO | SRI-AS1 | 0.703668535 | 4.35E-167 | positive |
| BRAF | SRI-AS1 | 0.600020329 | 8.39E-110 | positive |
| DIABLO | AC104785.1 | 0.463813466 | 1.88E-60 | positive |
| BRAF | AC104785.1 | 0.42517495 | 4.41E-50 | positive |
| CD40 | AC133961.1 | 0.63585618 | 3.96E-127 | positive |
| SIRT3 | FOXD2-AS1 | 0.427610491 | 1.07E-50 | positive |
| TSC1 | AC104109.4 | 0.41961142 | 1.07E-48 | positive |
| MAPK8 | AC104109.4 | 0.432268814 | 6.91E-52 | positive |
| IPMK | AC104109.4 | 0.412270995 | 6.60E-47 | positive |
| OTULIN | AC104109.4 | 0.469064278 | 5.73E-62 | positive |
| CYLD | AC104109.4 | 0.497020037 | 1.71E-70 | positive |
| MAP3K7 | AC104109.4 | 0.423312097 | 1.29E-49 | positive |
| DIABLO | AC104109.4 | 0.739030764 | 8.24E-193 | positive |
| CFLAR | AC104109.4 | 0.496175083 | 3.17E-70 | positive |
| BRAF | AC104109.4 | 0.689493941 | 8.01E-158 | positive |
| ATRX | AC104109.4 | 0.495412319 | 5.54E-70 | positive |
| RNF31 | AC104109.4 | 0.420125795 | 8.00E-49 | positive |
| OTULIN | AC008149.2 | 0.406607087 | 1.48E-45 | positive |
| CYLD | AC008149.2 | 0.47325292 | 3.39E-63 | positive |
| DIABLO | AC008149.2 | 0.719857994 | 2.27E-178 | positive |
| CFLAR | AC008149.2 | 0.501758251 | 5.09E-72 | positive |
| BRAF | AC008149.2 | 0.653374267 | 1.82E-136 | positive |
| ATRX | AC008149.2 | 0.517096047 | 4.00E-77 | positive |
| TSC1 | RNF139-AS1 | 0.4694387 | 4.46E-62 | positive |
| MAPK8 | RNF139-AS1 | 0.426128818 | 2.54E-50 | positive |
| OTULIN | RNF139-AS1 | 0.428474512 | 6.46E-51 | positive |
| MAP3K7 | RNF139-AS1 | 0.435204926 | 1.20E-52 | positive |
| DIABLO | RNF139-AS1 | 0.600129822 | 7.49E-110 | positive |
| BRAF | RNF139-AS1 | 0.56520508 | 5.99E-95 | positive |
| RNF31 | RNF139-AS1 | 0.405902355 | 2.17E-45 | positive |
| KLF9 | AC135012.3 | 0.60425687 | 9.77E-112 | positive |
| MAPK8 | AL109614.1 | 0.418641702 | 1.86E-48 | positive |
| IPMK | AL109614.1 | 0.411202135 | 1.19E-46 | positive |
| OTULIN | AL109614.1 | 0.487350905 | 1.87E-67 | positive |
| CYLD | AL109614.1 | 0.40313543 | 9.65E-45 | positive |
| MAP3K7 | AL109614.1 | 0.43614304 | 6.85E-53 | positive |
| DIABLO | AL109614.1 | 0.763608574 | 2.66E-213 | positive |
| BRAF | AL109614.1 | 0.686996475 | 3.03E-156 | positive |
| BACH2 | LINC01352 | 0.446711687 | 1.07E-55 | positive |
| KLF9 | LINC01352 | 0.441314878 | 2.98E-54 | positive |
| CYLD | LIX1L-AS1 | 0.40057907 | 3.79E-44 | positive |
| DIABLO | LIX1L-AS1 | 0.459616547 | 2.92E-59 | positive |
| CFLAR | LIX1L-AS1 | 0.423496193 | 1.16E-49 | positive |
| BRAF | LIX1L-AS1 | 0.412614103 | 5.45E-47 | positive |
| ATRX | LIX1L-AS1 | 0.505025396 | 4.37E-73 | positive |
| DIABLO | FMR1-IT1 | 0.691962634 | 2.13E-159 | positive |
| BRAF | FMR1-IT1 | 0.616291094 | 2.16E-117 | positive |
| ATRX | FMR1-IT1 | 0.431663644 | 9.89E-52 | positive |
| TSC1 | LAMC1-AS1 | 0.46770212 | 1.43E-61 | positive |
| MAPK8 | LAMC1-AS1 | 0.464503069 | 1.19E-60 | positive |
| OTULIN | LAMC1-AS1 | 0.509762713 | 1.19E-74 | positive |
| CYLD | LAMC1-AS1 | 0.504360197 | 7.23E-73 | positive |
| MAP3K7 | LAMC1-AS1 | 0.479072846 | 6.25E-65 | positive |
| DIABLO | LAMC1-AS1 | 0.702355819 | 3.31E-166 | positive |
| CFLAR | LAMC1-AS1 | 0.490896915 | 1.47E-68 | positive |
| BRAF | LAMC1-AS1 | 0.718561684 | 1.94E-177 | positive |
| ATRX | LAMC1-AS1 | 0.573022352 | 3.95E-98 | positive |
| RNF31 | LAMC1-AS1 | 0.427164591 | 1.39E-50 | positive |
| OTULIN | LINC00598 | 0.458306359 | 6.83E-59 | positive |
| CYLD | LINC00598 | 0.411971464 | 7.79E-47 | positive |
| DIABLO | LINC00598 | 0.685711759 | 1.93E-155 | positive |
| BRAF | LINC00598 | 0.622237058 | 2.81E-120 | positive |
| ATRX | LINC00598 | 0.40838741 | 5.60E-46 | positive |
| CYLD | AC092810.3 | 0.40955301 | 2.95E-46 | positive |
| DIABLO | AC092810.3 | 0.496140258 | 3.25E-70 | positive |
| BRAF | AC092810.3 | 0.453793239 | 1.24E-57 | positive |
| DIABLO | AC084125.3 | 0.669265112 | 1.73E-145 | positive |
| BRAF | AC084125.3 | 0.428000859 | 8.52E-51 | positive |
| DIABLO | AC092171.1 | 0.759613634 | 8.37E-210 | positive |
| BRAF | AC092171.1 | 0.607681525 | 2.54E-113 | positive |
| ATRX | AC092171.1 | 0.402588649 | 1.29E-44 | positive |
| DIABLO | AL135786.2 | 0.44066906 | 4.42E-54 | positive |
| DIABLO | AC092757.2 | 0.447136078 | 8.21E-56 | positive |
| CFLAR | AC092757.2 | 0.407260569 | 1.04E-45 | positive |
| BRAF | AC092757.2 | 0.459298176 | 3.59E-59 | positive |
| ATRX | AC092757.2 | 0.454315933 | 8.87E-58 | positive |
| DIABLO | AL031651.2 | 0.489910537 | 2.99E-68 | positive |
| BRAF | AL031651.2 | 0.523992903 | 1.66E-79 | positive |
| DIABLO | AP001619.2 | 0.515007024 | 2.05E-76 | positive |
| BRAF | AP001619.2 | 0.454797517 | 6.52E-58 | positive |
| OTULIN | AC018693.1 | 0.415960977 | 8.42E-48 | positive |
| CYLD | AC018693.1 | 0.421236917 | 4.25E-49 | positive |
| DIABLO | AC018693.1 | 0.725976067 | 7.62E-183 | positive |
| BRAF | AC018693.1 | 0.621613942 | 5.67E-120 | positive |
| ATRX | AC018693.1 | 0.502444865 | 3.05E-72 | positive |
| MAPK8 | AL359715.2 | 0.401462696 | 2.36E-44 | positive |
| OTULIN | AL359715.2 | 0.442118305 | 1.82E-54 | positive |
| MAP3K7 | AL359715.2 | 0.447865478 | 5.21E-56 | positive |
| DIABLO | AL359715.2 | 0.633777797 | 4.63E-126 | positive |
| BRAF | AL359715.2 | 0.617195241 | 7.93E-118 | positive |
| TSC1 | AC097641.2 | 0.481863945 | 8.96E-66 | positive |
| OTULIN | AC097641.2 | 0.430049636 | 2.56E-51 | positive |
| MAP3K7 | AC097641.2 | 0.404309292 | 5.13E-45 | positive |
| DIABLO | AC097641.2 | 0.626165019 | 3.22E-122 | positive |
| CFLAR | AC097641.2 | 0.423079983 | 1.48E-49 | positive |
| BRAF | AC097641.2 | 0.552374791 | 6.57E-90 | positive |
| ATRX | AC097641.2 | 0.476971113 | 2.67E-64 | positive |
| RNF31 | AC097641.2 | 0.439419499 | 9.46E-54 | positive |
| DIABLO | Z94721.1 | 0.403033707 | 1.02E-44 | positive |
| BRAF | Z94721.1 | 0.427314766 | 1.27E-50 | positive |
| ATRX | Z94721.1 | 0.510477607 | 6.86E-75 | positive |
| RNF31 | Z94721.1 | 0.406176155 | 1.87E-45 | positive |
| TSC1 | AC011462.4 | 0.411641992 | 9.34E-47 | positive |
| RNF31 | AC011462.4 | 0.440738445 | 4.24E-54 | positive |
| DIABLO | AL050320.1 | 0.607420619 | 3.36E-113 | positive |
| BRAF | AL050320.1 | 0.576591401 | 1.30E-99 | positive |
| ATRX | AL050320.1 | 0.429411518 | 3.73E-51 | positive |
| DIABLO | AC061975.7 | 0.494706359 | 9.29E-70 | positive |
| BRAF | AC061975.7 | 0.470158827 | 2.75E-62 | positive |
| OTULIN | AC024588.1 | 0.565118573 | 6.49E-95 | positive |
| DIABLO | AC024588.1 | 0.692953025 | 4.92E-160 | positive |
| BRAF | AC024588.1 | 0.607684423 | 2.53E-113 | positive |
| ATRX | AC024588.1 | 0.422938783 | 1.60E-49 | positive |
| MAPK8 | AC099501.1 | 0.404488001 | 4.66E-45 | positive |
| IPMK | AC099501.1 | 0.403463831 | 8.09E-45 | positive |
| OTULIN | AC099501.1 | 0.452734705 | 2.43E-57 | positive |
| CYLD | AC099501.1 | 0.432441624 | 6.24E-52 | positive |
| DIABLO | AC099501.1 | 0.771956384 | 7.69E-221 | positive |
| BRAF | AC099501.1 | 0.697245587 | 8.02E-163 | positive |
| ATRX | AC099501.1 | 0.406347138 | 1.70E-45 | positive |
| ATRX | AC012313.3 | 0.459087866 | 4.12E-59 | positive |
| BACH2 | LINC01781 | 0.457600077 | 1.08E-58 | positive |
| DIABLO | AL162734.1 | 0.641518104 | 4.43E-130 | positive |
| BRAF | AL162734.1 | 0.584810275 | 4.31E-103 | positive |
| ATRX | AL162734.1 | 0.520159043 | 3.55E-78 | positive |
| DIABLO | AC020913.2 | 0.429037667 | 4.64E-51 | positive |
| CYLD | AL121933.2 | 0.436503322 | 5.51E-53 | positive |
| DIABLO | AL121933.2 | 0.537396616 | 2.70E-84 | positive |
| CFLAR | AL121933.2 | 0.421304936 | 4.09E-49 | positive |
| BRAF | AL121933.2 | 0.497285279 | 1.40E-70 | positive |
| OTULIN | MRTFA-AS1 | 0.442112563 | 1.83E-54 | positive |
| CYLD | MRTFA-AS1 | 0.429929429 | 2.75E-51 | positive |
| DIABLO | MRTFA-AS1 | 0.747593792 | 1.11E-199 | positive |
| CFLAR | MRTFA-AS1 | 0.404111199 | 5.71E-45 | positive |
| BRAF | MRTFA-AS1 | 0.65728641 | 1.23E-138 | positive |
| ATRX | MRTFA-AS1 | 0.422855703 | 1.68E-49 | positive |
| TSC1 | AC004067.1 | 0.431094101 | 1.38E-51 | positive |
| CYLD | AC004067.1 | 0.455638379 | 3.81E-58 | positive |
| DIABLO | AC004067.1 | 0.564264231 | 1.43E-94 | positive |
| CFLAR | AC004067.1 | 0.4135176 | 3.30E-47 | positive |
| BRAF | AC004067.1 | 0.550799637 | 2.64E-89 | positive |
| ATRX | AC004067.1 | 0.422753782 | 1.78E-49 | positive |
| DIABLO | AC026624.1 | 0.645557625 | 3.18E-132 | positive |
| BRAF | AC026624.1 | 0.539071059 | 6.57E-85 | positive |
| ATRX | AC026624.1 | 0.429627791 | 3.28E-51 | positive |
| KLF9 | AC027807.2 | 0.424971533 | 4.96E-50 | positive |
| OTULIN | AF178030.1 | 0.464940958 | 8.92E-61 | positive |
| CYLD | AF178030.1 | 0.414179965 | 2.28E-47 | positive |
| DIABLO | AF178030.1 | 0.677800151 | 1.43E-150 | positive |
| BRAF | AF178030.1 | 0.625863616 | 4.54E-122 | positive |
| DIABLO | AL162724.1 | 0.703097312 | 1.05E-166 | positive |
| CFLAR | AL162724.1 | 0.443508517 | 7.77E-55 | positive |
| BRAF | AL162724.1 | 0.599609688 | 1.29E-109 | positive |
| ATRX | AL162724.1 | 0.426683425 | 1.84E-50 | positive |
| TSC1 | AL590369.1 | 0.431440049 | 1.13E-51 | positive |
| RNF31 | AL590369.1 | 0.410098402 | 2.19E-46 | positive |
| CYLD | AP001528.1 | 0.461795055 | 7.06E-60 | positive |
| DIABLO | AP001528.1 | 0.532005893 | 2.41E-82 | positive |
| CFLAR | AP001528.1 | 0.464735139 | 1.02E-60 | positive |
| BRAF | AP001528.1 | 0.58325864 | 1.99E-102 | positive |
| ATRX | AP001528.1 | 0.474973746 | 1.05E-63 | positive |
| KLF9 | AP001528.1 | 0.566291249 | 2.19E-95 | positive |
| MAPK8 | AC121493.1 | 0.438314872 | 1.85E-53 | positive |
| DIABLO | AC121493.1 | 0.587856368 | 2.08E-104 | positive |
| CFLAR | AC121493.1 | 0.44367631 | 7.00E-55 | positive |
| BRAF | AC121493.1 | 0.623046612 | 1.12E-120 | positive |
| ATRX | AC121493.1 | 0.473678379 | 2.54E-63 | positive |
| TSC1 | AL049840.3 | 0.445563469 | 2.18E-55 | positive |
| TSC1 | AL021707.8 | 0.426291579 | 2.31E-50 | positive |
| CYLD | AL021707.8 | 0.483669272 | 2.53E-66 | positive |
| DIABLO | AL021707.8 | 0.585975671 | 1.36E-103 | positive |
| CFLAR | AL021707.8 | 0.511249458 | 3.79E-75 | positive |
| BRAF | AL021707.8 | 0.537367134 | 2.76E-84 | positive |
| ATRX | AL021707.8 | 0.403261693 | 9.01E-45 | positive |
| OTULIN | LINC00456 | 0.427040946 | 1.49E-50 | positive |
| DIABLO | LINC00456 | 0.764178908 | 8.31E-214 | positive |
| BRAF | LINC00456 | 0.647228026 | 4.05E-133 | positive |
| MAPK8 | AC138393.3 | 0.440865925 | 3.92E-54 | positive |
| IPMK | AC138393.3 | 0.432559916 | 5.81E-52 | positive |
| OTULIN | AC138393.3 | 0.491941592 | 6.92E-69 | positive |
| CYLD | AC138393.3 | 0.444901227 | 3.29E-55 | positive |
| MAP3K7 | AC138393.3 | 0.452708486 | 2.47E-57 | positive |
| DIABLO | AC138393.3 | 0.737904274 | 6.30E-192 | positive |
| CFLAR | AC138393.3 | 0.479361436 | 5.12E-65 | positive |
| BRAF | AC138393.3 | 0.68507181 | 4.85E-155 | positive |
| ATRX | AC138393.3 | 0.513600243 | 6.14E-76 | positive |
| DIABLO | AC016831.6 | 0.786205965 | 1.75E-234 | positive |
| BRAF | AC016831.6 | 0.646179798 | 1.48E-132 | positive |
| MAPK8 | ZNF451-AS1 | 0.449476624 | 1.90E-56 | positive |
| IPMK | ZNF451-AS1 | 0.419152572 | 1.39E-48 | positive |
| OTULIN | ZNF451-AS1 | 0.51383023 | 5.14E-76 | positive |
| CYLD | ZNF451-AS1 | 0.474386839 | 1.57E-63 | positive |
| MAP3K7 | ZNF451-AS1 | 0.4416127 | 2.49E-54 | positive |
| DIABLO | ZNF451-AS1 | 0.79806548 | 1.16E-246 | positive |
| CFLAR | ZNF451-AS1 | 0.437412905 | 3.19E-53 | positive |
| BRAF | ZNF451-AS1 | 0.735175049 | 8.34E-190 | positive |
| ATRX | ZNF451-AS1 | 0.488017153 | 1.16E-67 | positive |
| OTULIN | GTF3C2-AS1 | 0.420526998 | 6.37E-49 | positive |
| CYLD | GTF3C2-AS1 | 0.41772704 | 3.12E-48 | positive |
| DIABLO | GTF3C2-AS1 | 0.778265691 | 9.35E-227 | positive |
| CFLAR | GTF3C2-AS1 | 0.439204996 | 1.08E-53 | positive |
| BRAF | GTF3C2-AS1 | 0.678306189 | 7.04E-151 | positive |
| ATRX | GTF3C2-AS1 | 0.560305393 | 5.35E-93 | positive |
| RNF31 | GTF3C2-AS1 | 0.439702517 | 7.97E-54 | positive |
| DIABLO | AL078604.2 | 0.446310376 | 1.37E-55 | positive |
| DIABLO | AC011676.2 | 0.746079534 | 1.91E-198 | positive |
| BRAF | AC011676.2 | 0.604747423 | 5.81E-112 | positive |
| TSC1 | AC025287.3 | 0.511135214 | 4.14E-75 | positive |
| MAPK8 | AC025287.3 | 0.417005778 | 4.68E-48 | positive |
| OTULIN | AC025287.3 | 0.508824965 | 2.44E-74 | positive |
| CYLD | AC025287.3 | 0.544424386 | 6.80E-87 | positive |
| MAP3K7 | AC025287.3 | 0.431082794 | 1.39E-51 | positive |
| DIABLO | AC025287.3 | 0.68542977 | 2.90E-155 | positive |
| CFLAR | AC025287.3 | 0.521298228 | 1.44E-78 | positive |
| BRAF | AC025287.3 | 0.650847718 | 4.43E-135 | positive |
| ATRX | AC025287.3 | 0.494299523 | 1.25E-69 | positive |
| RNF31 | AC025287.3 | 0.450623311 | 9.23E-57 | positive |
| OTULIN | AC005828.1 | 0.460371065 | 1.79E-59 | positive |
| CYLD | AC005828.1 | 0.406053825 | 2.00E-45 | positive |
| DIABLO | AC005828.1 | 0.6782239 | 7.90E-151 | positive |
| BRAF | AC005828.1 | 0.613415783 | 5.10E-116 | positive |
| ATRX | AC093582.1 | 0.407947821 | 7.12E-46 | positive |
| RNF31 | AC093582.1 | 0.421491762 | 3.67E-49 | positive |
| TSC1 | GAS8-AS1 | 0.419615052 | 1.07E-48 | positive |
| DIABLO | GAS8-AS1 | 0.640534308 | 1.46E-129 | positive |
| BRAF | GAS8-AS1 | 0.528724571 | 3.58E-81 | positive |
| ATRX | GAS8-AS1 | 0.405787422 | 2.31E-45 | positive |
| RNF31 | GAS8-AS1 | 0.499744975 | 2.28E-71 | positive |
| DIABLO | AC013472.2 | 0.52960333 | 1.74E-81 | positive |
| BRAF | AC013472.2 | 0.431715948 | 9.59E-52 | positive |
| OTULIN | JARID2-AS1 | 0.431061193 | 1.41E-51 | positive |
| DIABLO | JARID2-AS1 | 0.707895837 | 5.85E-170 | positive |
| BRAF | JARID2-AS1 | 0.589310599 | 4.85E-105 | positive |
| TSC1 | AC048341.1 | 0.428653244 | 5.82E-51 | positive |
| MAPK8 | AC048341.1 | 0.456757234 | 1.86E-58 | positive |
| OTULIN | AC048341.1 | 0.419416271 | 1.20E-48 | positive |
| CYLD | AC048341.1 | 0.499498126 | 2.74E-71 | positive |
| MAP3K7 | AC048341.1 | 0.408332383 | 5.77E-46 | positive |
| DIABLO | AC048341.1 | 0.718863164 | 1.18E-177 | positive |
| CFLAR | AC048341.1 | 0.51524149 | 1.71E-76 | positive |
| BRAF | AC048341.1 | 0.664346308 | 1.23E-142 | positive |
| ATRX | AC048341.1 | 0.546710573 | 9.41E-88 | positive |
| RNF31 | AC048341.1 | 0.407660602 | 8.32E-46 | positive |
| CFLAR | AC139795.2 | 0.461652004 | 7.76E-60 | positive |
| ATRX | AC139795.2 | 0.529799925 | 1.48E-81 | positive |
| RNF31 | AL135999.1 | 0.587619954 | 2.64E-104 | positive |
| MAPK8 | AC099811.5 | 0.406845341 | 1.30E-45 | positive |
| OTULIN | AC099811.5 | 0.458700068 | 5.30E-59 | positive |
| CYLD | AC099811.5 | 0.466930522 | 2.38E-61 | positive |
| DIABLO | AC099811.5 | 0.787673618 | 6.01E-236 | positive |
| CFLAR | AC099811.5 | 0.415299621 | 1.22E-47 | positive |
| BRAF | AC099811.5 | 0.710540009 | 8.81E-172 | positive |
| ATRX | AC099811.5 | 0.447343261 | 7.22E-56 | positive |
| DIABLO | CXXC5-AS1 | 0.490521175 | 1.93E-68 | positive |
| BRAF | CXXC5-AS1 | 0.403530665 | 7.80E-45 | positive |
| OTULIN | AL121906.1 | 0.429493825 | 3.55E-51 | positive |
| DIABLO | AL121906.1 | 0.594530259 | 2.44E-107 | positive |
| BRAF | AL121906.1 | 0.489183561 | 5.04E-68 | positive |
| KLF9 | AC005180.2 | 0.530428284 | 8.86E-82 | positive |
| DIABLO | L34079.2 | 0.586768973 | 6.17E-104 | positive |
| BRAF | L34079.2 | 0.482045938 | 7.89E-66 | positive |
| MAPK8 | LINC01572 | 0.412248271 | 6.68E-47 | positive |
| IPMK | LINC01572 | 0.416141368 | 7.61E-48 | positive |
| OTULIN | LINC01572 | 0.501334251 | 6.98E-72 | positive |
| CYLD | LINC01572 | 0.420517282 | 6.40E-49 | positive |
| MAP3K7 | LINC01572 | 0.433322934 | 3.69E-52 | positive |
| DIABLO | LINC01572 | 0.734329684 | 3.74E-189 | positive |
| BRAF | LINC01572 | 0.67134121 | 1.04E-146 | positive |
| ATRX | LINC01572 | 0.444520283 | 4.16E-55 | positive |
| DIABLO | AC012368.2 | 0.461827888 | 6.91E-60 | positive |
| BRAF | AC012368.2 | 0.418058575 | 2.59E-48 | positive |
| OTULIN | AC006566.1 | 0.431297 | 1.23E-51 | positive |
| CYLD | AC006566.1 | 0.421690154 | 3.28E-49 | positive |
| DIABLO | AC006566.1 | 0.691986947 | 2.06E-159 | positive |
| BRAF | AC006566.1 | 0.636372527 | 2.14E-127 | positive |
| ATRX | AC006566.1 | 0.447924111 | 5.02E-56 | positive |
| OTULIN | LARS2-AS1 | 0.422978839 | 1.57E-49 | positive |
| DIABLO | LARS2-AS1 | 0.771803724 | 1.06E-220 | positive |
| BRAF | LARS2-AS1 | 0.653615763 | 1.34E-136 | positive |
| DIABLO | NHS-AS1 | 0.579984027 | 4.90E-101 | positive |
| BRAF | NHS-AS1 | 0.513432289 | 7.00E-76 | positive |
| TSC1 | TRAPPC12-AS1 | 0.403648639 | 7.32E-45 | positive |
| TSC1 | AC112907.3 | 0.44304262 | 1.03E-54 | positive |
| DIABLO | AC112907.3 | 0.494192647 | 1.35E-69 | positive |
| BRAF | AC112907.3 | 0.454128625 | 1.00E-57 | positive |
| TSC1 | AC005332.5 | 0.419246902 | 1.32E-48 | positive |
| MAPK8 | AC103739.2 | 0.426897141 | 1.62E-50 | positive |
| IPMK | AC103739.2 | 0.417490694 | 3.56E-48 | positive |
| OTULIN | AC103739.2 | 0.444342981 | 4.64E-55 | positive |
| CYLD | AC103739.2 | 0.457465567 | 1.18E-58 | positive |
| DIABLO | AC103739.2 | 0.769928097 | 5.58E-219 | positive |
| CFLAR | AC103739.2 | 0.471961722 | 8.14E-63 | positive |
| BRAF | AC103739.2 | 0.704278938 | 1.69E-167 | positive |
| ATRX | AC103739.2 | 0.489131244 | 5.23E-68 | positive |
| MAPK8 | AC007390.1 | 0.466790833 | 2.62E-61 | positive |
| IPMK | AC007390.1 | 0.442088762 | 1.86E-54 | positive |
| OTULIN | AC007390.1 | 0.506001142 | 2.09E-73 | positive |
| CYLD | AC007390.1 | 0.492721249 | 3.93E-69 | positive |
| MAP3K7 | AC007390.1 | 0.425778419 | 3.11E-50 | positive |
| DIABLO | AC007390.1 | 0.802947823 | 6.50E-252 | positive |
| CFLAR | AC007390.1 | 0.454658719 | 7.13E-58 | positive |
| BRAF | AC007390.1 | 0.754600237 | 1.65E-205 | positive |
| ATRX | AC007390.1 | 0.529417257 | 2.03E-81 | positive |
| BACH2 | AL157895.1 | 0.436482887 | 5.58E-53 | positive |
| MAPK8 | AC007881.4 | 0.449658492 | 1.69E-56 | positive |
| IPMK | AC007881.4 | 0.411443701 | 1.04E-46 | positive |
| OTULIN | AC007881.4 | 0.464076979 | 1.58E-60 | positive |
| CYLD | AC007881.4 | 0.458799995 | 4.96E-59 | positive |
| DIABLO | AC007881.4 | 0.758418862 | 9.03E-209 | positive |
| CFLAR | AC007881.4 | 0.461862915 | 6.76E-60 | positive |
| BRAF | AC007881.4 | 0.709869814 | 2.56E-171 | positive |
| ATRX | AC007881.4 | 0.469586245 | 4.04E-62 | positive |
| CYLD | AC018529.2 | 0.409705395 | 2.72E-46 | positive |
| DIABLO | AC018529.2 | 0.738484083 | 2.21E-192 | positive |
| CFLAR | AC018529.2 | 0.417324738 | 3.91E-48 | positive |
| BRAF | AC018529.2 | 0.595542549 | 8.65E-108 | positive |
| OTULIN | AL355613.1 | 0.452128483 | 3.56E-57 | positive |
| DIABLO | AL355613.1 | 0.714212772 | 2.40E-174 | positive |
| BRAF | AL355613.1 | 0.631379924 | 7.71E-125 | positive |
| OTULIN | AC006460.2 | 0.464977275 | 8.71E-61 | positive |
| CYLD | AC006460.2 | 0.413540012 | 3.26E-47 | positive |
| MAP3K7 | AC006460.2 | 0.402265352 | 1.54E-44 | positive |
| DIABLO | AC006460.2 | 0.503129626 | 1.82E-72 | positive |
| CFLAR | AC006460.2 | 0.423826949 | 9.61E-50 | positive |
| BRAF | AC006460.2 | 0.539891842 | 3.28E-85 | positive |
| ATRX | AC006460.2 | 0.448963233 | 2.62E-56 | positive |
| OTULIN | AF127577.4 | 0.429450196 | 3.65E-51 | positive |
| CYLD | AF127577.4 | 0.415579899 | 1.04E-47 | positive |
| MAP3K7 | AF127577.4 | 0.410819183 | 1.47E-46 | positive |
| BRAF | AF127577.4 | 0.458290987 | 6.90E-59 | positive |
| MAPK8 | AC087752.4 | 0.405323929 | 2.96E-45 | positive |
| DIABLO | AC087752.4 | 0.545381805 | 2.98E-87 | positive |
| BRAF | AC087752.4 | 0.494861216 | 8.29E-70 | positive |
| TSC1 | AL132780.1 | 0.418829443 | 1.67E-48 | positive |
| DIABLO | AL132780.1 | 0.567118057 | 1.02E-95 | positive |
| BRAF | AL132780.1 | 0.544242694 | 7.95E-87 | positive |
| DIABLO | AC079915.1 | 0.722695281 | 1.98E-180 | positive |
| BRAF | AC079915.1 | 0.642476352 | 1.38E-130 | positive |
| ATRX | AC079915.1 | 0.459323116 | 3.54E-59 | positive |
| OTULIN | MAL2-AS1 | 0.424432461 | 6.78E-50 | positive |
| DIABLO | MAL2-AS1 | 0.765166585 | 1.10E-214 | positive |
| BRAF | MAL2-AS1 | 0.648252599 | 1.13E-133 | positive |
| OTULIN | AC009948.1 | 0.452039537 | 3.77E-57 | positive |
| DIABLO | AC009948.1 | 0.702742626 | 1.82E-166 | positive |
| BRAF | AC009948.1 | 0.66078301 | 1.33E-140 | positive |
| ATRX | AC009948.1 | 0.404206067 | 5.42E-45 | positive |
| DIABLO | AC015908.4 | 0.509707033 | 1.24E-74 | positive |
| MAPK8 | AC018647.3 | 0.424183388 | 7.82E-50 | positive |
| IPMK | AC018647.3 | 0.419588567 | 1.09E-48 | positive |
| OTULIN | AC018647.3 | 0.433114522 | 4.18E-52 | positive |
| CYLD | AC018647.3 | 0.437520276 | 2.99E-53 | positive |
| DIABLO | AC018647.3 | 0.702719715 | 1.89E-166 | positive |
| CFLAR | AC018647.3 | 0.402841606 | 1.13E-44 | positive |
| BRAF | AC018647.3 | 0.643607031 | 3.49E-131 | positive |
| ATRX | AC018647.3 | 0.489941949 | 2.92E-68 | positive |
| MAPK8 | AC093675.2 | 0.411176515 | 1.21E-46 | positive |
| OTULIN | AC093675.2 | 0.475579924 | 6.94E-64 | positive |
| CYLD | AC093675.2 | 0.440973137 | 3.67E-54 | positive |
| DIABLO | AC093675.2 | 0.820336019 | 6.79E-272 | positive |
| CFLAR | AC093675.2 | 0.401349554 | 2.51E-44 | positive |
| BRAF | AC093675.2 | 0.716338574 | 7.52E-176 | positive |
| ATRX | AC093675.2 | 0.432134129 | 7.48E-52 | positive |
| TSC1 | AC007066.2 | 0.580107881 | 4.35E-101 | positive |
| MAPK8 | AC007066.2 | 0.437082782 | 3.89E-53 | positive |
| OTULIN | AC007066.2 | 0.430306263 | 2.20E-51 | positive |
| MAP3K7 | AC007066.2 | 0.501101097 | 8.31E-72 | positive |
| DIABLO | AC007066.2 | 0.536110666 | 7.94E-84 | positive |
| BRAF | AC007066.2 | 0.547608559 | 4.31E-88 | positive |
| DIABLO | AOAH-IT1 | 0.575059321 | 5.67E-99 | positive |
| BRAF | AOAH-IT1 | 0.530854888 | 6.24E-82 | positive |
| SIRT3 | AC135050.6 | 0.434407976 | 1.93E-52 | positive |
| TARDBP | AC135050.6 | 0.450059746 | 1.32E-56 | positive |
| DIABLO | AL512643.2 | 0.433188159 | 4.00E-52 | positive |
| OTULIN | AL020997.2 | 0.436958323 | 4.19E-53 | positive |
| CYLD | AL020997.2 | 0.424912548 | 5.13E-50 | positive |
| DIABLO | AL020997.2 | 0.813155136 | 2.17E-263 | positive |
| BRAF | AL020997.2 | 0.668471432 | 5.02E-145 | positive |
| TSC1 | AL391684.1 | 0.533455612 | 7.27E-83 | positive |
| MAPK8 | AL391684.1 | 0.422378297 | 2.21E-49 | positive |
| OTULIN | AL391684.1 | 0.445999365 | 1.67E-55 | positive |
| MAP3K7 | AL391684.1 | 0.464656355 | 1.08E-60 | positive |
| DIABLO | AL391684.1 | 0.567259613 | 8.90E-96 | positive |
| BRAF | AL391684.1 | 0.609745492 | 2.76E-114 | positive |
| ATRX | AL391684.1 | 0.466561572 | 3.05E-61 | positive |
| DIABLO | AL138999.2 | 0.637452752 | 5.91E-128 | positive |
| BRAF | AL138999.2 | 0.475598889 | 6.85E-64 | positive |
| OTULIN | Z99289.3 | 0.433310571 | 3.72E-52 | positive |
| CYLD | Z99289.3 | 0.407580429 | 8.70E-46 | positive |
| DIABLO | Z99289.3 | 0.688830876 | 2.11E-157 | positive |
| BRAF | Z99289.3 | 0.653034678 | 2.81E-136 | positive |
| TSC1 | AC078846.1 | 0.459786686 | 2.62E-59 | positive |
| OTULIN | AC078846.1 | 0.443105144 | 9.95E-55 | positive |
| MAP3K7 | AC078846.1 | 0.426494426 | 2.05E-50 | positive |
| DIABLO | AC078846.1 | 0.713988303 | 3.45E-174 | positive |
| BRAF | AC078846.1 | 0.695780823 | 7.26E-162 | positive |
| ATRX | AC078846.1 | 0.457312685 | 1.30E-58 | positive |
| DIABLO | LINC02456 | 0.49404776 | 1.50E-69 | positive |
| BRAF | LINC02456 | 0.427360183 | 1.24E-50 | positive |
| OTULIN | AC073593.2 | 0.502343235 | 3.29E-72 | positive |
| CYLD | AC073593.2 | 0.449098797 | 2.41E-56 | positive |
| DIABLO | AC073593.2 | 0.754545628 | 1.83E-205 | positive |
| BRAF | AC073593.2 | 0.663116187 | 6.23E-142 | positive |
| ATRX | AC073593.2 | 0.466472458 | 3.23E-61 | positive |
| TSC1 | AP001001.1 | 0.43221551 | 7.13E-52 | positive |
| MAPK8 | AP001001.1 | 0.497458627 | 1.23E-70 | positive |
| IPMK | AP001001.1 | 0.477234974 | 2.23E-64 | positive |
| OTULIN | AP001001.1 | 0.52161618 | 1.11E-78 | positive |
| CYLD | AP001001.1 | 0.502846953 | 2.25E-72 | positive |
| MAP3K7 | AP001001.1 | 0.455401693 | 4.43E-58 | positive |
| DIABLO | AP001001.1 | 0.762610872 | 2.02E-212 | positive |
| CFLAR | AP001001.1 | 0.488316088 | 9.37E-68 | positive |
| BRAF | AP001001.1 | 0.767084222 | 2.11E-216 | positive |
| ATRX | AP001001.1 | 0.575073953 | 5.59E-99 | positive |
| DIABLO | AL021918.3 | 0.407599446 | 8.61E-46 | positive |
| TSC1 | ZBTB40-IT1 | 0.564189444 | 1.53E-94 | positive |
| MAPK8 | ZBTB40-IT1 | 0.408253356 | 6.02E-46 | positive |
| OTULIN | ZBTB40-IT1 | 0.418948282 | 1.56E-48 | positive |
| CYLD | ZBTB40-IT1 | 0.464021088 | 1.64E-60 | positive |
| DIABLO | ZBTB40-IT1 | 0.566198132 | 2.39E-95 | positive |
| CFLAR | ZBTB40-IT1 | 0.495021803 | 7.38E-70 | positive |
| BRAF | ZBTB40-IT1 | 0.610251936 | 1.60E-114 | positive |
| ATRX | ZBTB40-IT1 | 0.545377154 | 2.99E-87 | positive |
| RNF31 | ZBTB40-IT1 | 0.49926654 | 3.25E-71 | positive |
| MAPK8 | AC008119.1 | 0.440158202 | 6.04E-54 | positive |
| IPMK | AC008119.1 | 0.41535753 | 1.18E-47 | positive |
| OTULIN | AC008119.1 | 0.52396395 | 1.70E-79 | positive |
| CYLD | AC008119.1 | 0.467805804 | 1.33E-61 | positive |
| MAP3K7 | AC008119.1 | 0.411646344 | 9.32E-47 | positive |
| DIABLO | AC008119.1 | 0.823569043 | 7.55E-276 | positive |
| BRAF | AC008119.1 | 0.726929611 | 1.49E-183 | positive |
| ATRX | AC008119.1 | 0.42334713 | 1.27E-49 | positive |
| TSC1 | AL132989.1 | 0.597299942 | 1.41E-108 | positive |
| MAPK8 | AL132989.1 | 0.445547324 | 2.20E-55 | positive |
| OTULIN | AL132989.1 | 0.471427999 | 1.17E-62 | positive |
| CYLD | AL132989.1 | 0.56258501 | 6.68E-94 | positive |
| MAP3K7 | AL132989.1 | 0.459393871 | 3.38E-59 | positive |
| DIABLO | AL132989.1 | 0.645188577 | 5.01E-132 | positive |
| CFLAR | AL132989.1 | 0.593911757 | 4.59E-107 | positive |
| BRAF | AL132989.1 | 0.667847402 | 1.16E-144 | positive |
| ATRX | AL132989.1 | 0.603377752 | 2.48E-111 | positive |
| RNF31 | AL132989.1 | 0.521188509 | 1.57E-78 | positive |
| TSC1 | AC025766.1 | 0.436699147 | 4.90E-53 | positive |
| OTULIN | AC025766.1 | 0.426836815 | 1.68E-50 | positive |
| CYLD | AC025766.1 | 0.432838731 | 4.93E-52 | positive |
| MAP3K7 | AC025766.1 | 0.413852155 | 2.74E-47 | positive |
| DIABLO | AC025766.1 | 0.581427418 | 1.20E-101 | positive |
| BRAF | AC025766.1 | 0.566913308 | 1.23E-95 | positive |
| ATRX | AC025766.1 | 0.406476616 | 1.59E-45 | positive |
| OTULIN | AL513190.1 | 0.404776801 | 3.98E-45 | positive |
| BCL2 | AL513190.1 | 0.419594166 | 1.08E-48 | positive |
| CD40 | AL513190.1 | 0.489353692 | 4.46E-68 | positive |
| BCL2L11 | AL513190.1 | 0.475264949 | 8.60E-64 | positive |
| OTULIN | AC009432.2 | 0.457455575 | 1.18E-58 | positive |
| DIABLO | AC009432.2 | 0.740998915 | 2.30E-194 | positive |
| BRAF | AC009432.2 | 0.638959058 | 9.73E-129 | positive |
| MPG | AC008608.2 | 0.433848018 | 2.70E-52 | positive |
| ATRX | LINC02887 | 0.42359995 | 1.10E-49 | positive |
| DIABLO | AL035416.1 | 0.508666026 | 2.75E-74 | positive |
| BRAF | AL035416.1 | 0.47937435 | 5.07E-65 | positive |
| KLF9 | AC009102.2 | 0.606027628 | 1.49E-112 | positive |
| MAPK8 | ACAP2-IT1 | 0.469012659 | 5.93E-62 | positive |
| IPMK | ACAP2-IT1 | 0.476045927 | 5.04E-64 | positive |
| OTULIN | ACAP2-IT1 | 0.556141584 | 2.29E-91 | positive |
| CYLD | ACAP2-IT1 | 0.526134538 | 2.94E-80 | positive |
| MAP3K7 | ACAP2-IT1 | 0.467724146 | 1.40E-61 | positive |
| DIABLO | ACAP2-IT1 | 0.800321662 | 4.53E-249 | positive |
| CFLAR | ACAP2-IT1 | 0.448047988 | 4.65E-56 | positive |
| BRAF | ACAP2-IT1 | 0.770269696 | 2.72E-219 | positive |
| ATRX | ACAP2-IT1 | 0.509003683 | 2.13E-74 | positive |
| MAPK8 | AC007216.3 | 0.400376001 | 4.22E-44 | positive |
| OTULIN | AC007216.3 | 0.431184855 | 1.31E-51 | positive |
| CYLD | AC007216.3 | 0.439649341 | 8.23E-54 | positive |
| DIABLO | AC007216.3 | 0.818701741 | 6.32E-270 | positive |
| CFLAR | AC007216.3 | 0.440411292 | 5.18E-54 | positive |
| BRAF | AC007216.3 | 0.68635557 | 7.64E-156 | positive |
| ATRX | AC007216.3 | 0.542294454 | 4.23E-86 | positive |
| DIABLO | AP000704.1 | 0.478868127 | 7.21E-65 | positive |
| BRAF | AP000704.1 | 0.444493342 | 4.23E-55 | positive |
| OTULIN | AC104984.3 | 0.415224158 | 1.27E-47 | positive |
| CYLD | AC104984.3 | 0.428484272 | 6.42E-51 | positive |
| DIABLO | AC104984.3 | 0.769806797 | 7.20E-219 | positive |
| BRAF | AC104984.3 | 0.691685334 | 3.21E-159 | positive |
| ATRX | AC104984.3 | 0.462224184 | 5.33E-60 | positive |
| DIABLO | AC009090.3 | 0.535318172 | 1.54E-83 | positive |
| CFLAR | AC009090.3 | 0.436119477 | 6.94E-53 | positive |
| BRAF | AC009090.3 | 0.50892134 | 2.26E-74 | positive |
| ATRX | AC009090.3 | 0.501254578 | 7.41E-72 | positive |
| RNF31 | AC009090.3 | 0.403515136 | 7.87E-45 | positive |
| BACH2 | AC092068.2 | 0.446827369 | 9.95E-56 | positive |
| OTULIN | AL354833.1 | 0.431199834 | 1.30E-51 | positive |
| DIABLO | AL354833.1 | 0.720069211 | 1.60E-178 | positive |
| BRAF | AL354833.1 | 0.634993036 | 1.10E-126 | positive |
| ATRX | AL354833.1 | 0.495681843 | 4.55E-70 | positive |
| DIABLO | AC017104.2 | 0.461878562 | 6.69E-60 | positive |
| ATRX | AC017104.2 | 0.477915506 | 1.39E-64 | positive |
| DIABLO | AL034417.4 | 0.512955736 | 1.01E-75 | positive |
| CFLAR | AL034417.4 | 0.429697998 | 3.15E-51 | positive |
| BRAF | AL034417.4 | 0.458046054 | 8.08E-59 | positive |
| ATRX | AL034417.4 | 0.534569183 | 2.88E-83 | positive |
| RNF31 | AL034417.4 | 0.484447715 | 1.46E-66 | positive |
| ATRX | AL513477.2 | 0.406850383 | 1.29E-45 | positive |
| TSC1 | AL049780.1 | 0.489690324 | 3.50E-68 | positive |
| OTULIN | AL049780.1 | 0.41620499 | 7.35E-48 | positive |
| CYLD | AL049780.1 | 0.414558246 | 1.85E-47 | positive |
| DIABLO | AL049780.1 | 0.674178893 | 2.15E-148 | positive |
| CFLAR | AL049780.1 | 0.418102484 | 2.52E-48 | positive |
| BRAF | AL049780.1 | 0.624949538 | 1.29E-121 | positive |
| ATRX | AL049780.1 | 0.444468563 | 4.30E-55 | positive |
| RNF31 | AL049780.1 | 0.424568187 | 6.26E-50 | positive |
| DIABLO | AC010883.3 | 0.686185011 | 9.78E-156 | positive |
| BRAF | AC010883.3 | 0.570386127 | 4.77E-97 | positive |
| STUB1 | AC106886.2 | 0.444365329 | 4.58E-55 | positive |
| KLF9 | AC244453.3 | 0.423993902 | 8.73E-50 | positive |
| KLF9 | AL132642.1 | 0.455096557 | 5.39E-58 | positive |
| TSC1 | AC091185.1 | 0.541927295 | 5.79E-86 | positive |
| MAPK8 | AC091185.1 | 0.476039435 | 5.06E-64 | positive |
| OTULIN | AC091185.1 | 0.462902034 | 3.42E-60 | positive |
| CYLD | AC091185.1 | 0.49473029 | 9.13E-70 | positive |
| MAP3K7 | AC091185.1 | 0.43840799 | 1.75E-53 | positive |
| DIABLO | AC091185.1 | 0.54768205 | 4.04E-88 | positive |
| CFLAR | AC091185.1 | 0.512477409 | 1.47E-75 | positive |
| BRAF | AC091185.1 | 0.619170082 | 8.80E-119 | positive |
| ATRX | AC091185.1 | 0.580985077 | 1.85E-101 | positive |
| RNF31 | AC091185.1 | 0.451455888 | 5.46E-57 | positive |
| DIABLO | AP000893.2 | 0.680843106 | 2.00E-152 | positive |
| BRAF | AP000893.2 | 0.606705689 | 7.22E-113 | positive |
| DIABLO | LINC00937 | 0.574476105 | 9.89E-99 | positive |
| BRAF | LINC00937 | 0.456589343 | 2.07E-58 | positive |
| DIABLO | SHANK2-AS3 | 0.745987413 | 2.27E-198 | positive |
| BRAF | SHANK2-AS3 | 0.592343803 | 2.27E-106 | positive |
| OTULIN | AC087501.1 | 0.449549333 | 1.81E-56 | positive |
| CYLD | AC087501.1 | 0.478407757 | 9.91E-65 | positive |
| DIABLO | AC087501.1 | 0.748729615 | 1.30E-200 | positive |
| CFLAR | AC087501.1 | 0.45227805 | 3.24E-57 | positive |
| BRAF | AC087501.1 | 0.676864048 | 5.25E-150 | positive |
| ATRX | AC087501.1 | 0.50500973 | 4.43E-73 | positive |
| DIABLO | AL391095.3 | 0.535153767 | 1.77E-83 | positive |
| DIABLO | TBILA | 0.460241617 | 1.95E-59 | positive |
| BRAF | TBILA | 0.424814935 | 5.43E-50 | positive |
| CYLD | AP001330.4 | 0.406148947 | 1.90E-45 | positive |
| ATRX | AP001330.4 | 0.475481015 | 7.42E-64 | positive |
| DIABLO | AC124276.2 | 0.634623435 | 1.71E-126 | positive |
| BRAF | AC124276.2 | 0.522982267 | 3.74E-79 | positive |
| DIABLO | AL583722.3 | 0.616042502 | 2.84E-117 | positive |
| BRAF | AL583722.3 | 0.476694273 | 3.23E-64 | positive |
| DIABLO | TNS1-AS1 | 0.400730051 | 3.49E-44 | positive |
| BRAF | TNS1-AS1 | 0.428928493 | 4.95E-51 | positive |
| ATRX | TNS1-AS1 | 0.427088361 | 1.45E-50 | positive |
| DIABLO | AL355377.3 | 0.597469543 | 1.19E-108 | positive |
| BRAF | AL355377.3 | 0.55643124 | 1.77E-91 | positive |
| ATRX | AL355377.3 | 0.46372041 | 2.00E-60 | positive |
| DIABLO | AC012485.1 | 0.517712857 | 2.46E-77 | positive |
| BRAF | AC012485.1 | 0.448192324 | 4.25E-56 | positive |
| DIABLO | AC009107.1 | 0.669651065 | 1.02E-145 | positive |
| BRAF | AC009107.1 | 0.544555499 | 6.07E-87 | positive |
| DIABLO | AL135790.1 | 0.544516594 | 6.28E-87 | positive |
| BRAF | AL135790.1 | 0.500706214 | 1.12E-71 | positive |
| CYLD | U73169.1 | 0.416562071 | 6.01E-48 | positive |
| DIABLO | U73169.1 | 0.620966509 | 1.18E-119 | positive |
| CFLAR | U73169.1 | 0.45860448 | 5.63E-59 | positive |
| BRAF | U73169.1 | 0.550705273 | 2.87E-89 | positive |
| OTULIN | AL390242.1 | 0.461529767 | 8.40E-60 | positive |
| CYLD | AL390242.1 | 0.494714839 | 9.23E-70 | positive |
| DIABLO | AL390242.1 | 0.747131434 | 2.66E-199 | positive |
| CFLAR | AL390242.1 | 0.471578824 | 1.06E-62 | positive |
| BRAF | AL390242.1 | 0.692583011 | 8.52E-160 | positive |
| ATRX | AL390242.1 | 0.557437284 | 7.16E-92 | positive |
| MAPK8 | AC005021.1 | 0.425668615 | 3.31E-50 | positive |
| OTULIN | AC005021.1 | 0.44546221 | 2.32E-55 | positive |
| DIABLO | AC005021.1 | 0.631051828 | 1.13E-124 | positive |
| CFLAR | AC005021.1 | 0.426460629 | 2.09E-50 | positive |
| BRAF | AC005021.1 | 0.593361468 | 8.05E-107 | positive |
| ATRX | AC005021.1 | 0.446450379 | 1.26E-55 | positive |
| CYLD | FBXO30-DT | 0.413241536 | 3.85E-47 | positive |
| MAP3K7 | FBXO30-DT | 0.453204104 | 1.80E-57 | positive |
| DIABLO | FBXO30-DT | 0.494980484 | 7.60E-70 | positive |
| BRAF | FBXO30-DT | 0.526807451 | 1.71E-80 | positive |
| BACH2 | FBXO30-DT | 0.467599649 | 1.53E-61 | positive |
| ATRX | FBXO30-DT | 0.416121283 | 7.70E-48 | positive |
| KLF9 | FBXO30-DT | 0.417603426 | 3.34E-48 | positive |
| OTULIN | AL138733.1 | 0.425557594 | 3.53E-50 | positive |
| DIABLO | AL138733.1 | 0.737406567 | 1.54E-191 | positive |
| BRAF | AL138733.1 | 0.597719461 | 9.17E-109 | positive |
| DIABLO | SHROOM3-AS1 | 0.674582599 | 1.23E-148 | positive |
| BRAF | SHROOM3-AS1 | 0.521539754 | 1.18E-78 | positive |
| TSC1 | AL513218.1 | 0.420356446 | 7.02E-49 | positive |
| DIABLO | AL513218.1 | 0.49438126 | 1.18E-69 | positive |
| BRAF | AL513218.1 | 0.427756844 | 9.83E-51 | positive |
| RNF31 | AL513218.1 | 0.439870254 | 7.19E-54 | positive |
| MAP3K7 | HMGN3-AS1 | 0.506793826 | 1.15E-73 | positive |
| BRAF | HMGN3-AS1 | 0.439381878 | 9.68E-54 | positive |
| BACH2 | AC022239.1 | 0.604376732 | 8.60E-112 | positive |
| OTULIN | AC021237.1 | 0.41266934 | 5.29E-47 | positive |
| DIABLO | AC021237.1 | 0.741739843 | 5.92E-195 | positive |
| BRAF | AC021237.1 | 0.633288221 | 8.24E-126 | positive |
| TSC1 | AC024560.4 | 0.484760365 | 1.17E-66 | positive |
| DIABLO | AC024560.4 | 0.466453962 | 3.27E-61 | positive |
| BRAF | AC024560.4 | 0.460723431 | 1.42E-59 | positive |
| ATRX | AC024560.4 | 0.461640356 | 7.82E-60 | positive |
| OTULIN | AC090337.1 | 0.419353926 | 1.24E-48 | positive |
| CYLD | AC090337.1 | 0.431172837 | 1.32E-51 | positive |
| DIABLO | AC090337.1 | 0.722067075 | 5.68E-180 | positive |
| BRAF | AC090337.1 | 0.667506229 | 1.83E-144 | positive |
| DIABLO | H2AZ1-DT | 0.424017295 | 8.61E-50 | positive |
| BRAF | H2AZ1-DT | 0.410683748 | 1.59E-46 | positive |
| MAPK8 | AL355075.6 | 0.423339059 | 1.27E-49 | positive |
| IPMK | AL355075.6 | 0.406178496 | 1.86E-45 | positive |
| OTULIN | AL355075.6 | 0.516351675 | 7.17E-77 | positive |
| CYLD | AL355075.6 | 0.413511259 | 3.31E-47 | positive |
| MAP3K7 | AL355075.6 | 0.410584393 | 1.68E-46 | positive |
| DIABLO | AL355075.6 | 0.795685405 | 3.75E-244 | positive |
| BRAF | AL355075.6 | 0.689744543 | 5.55E-158 | positive |
| DIABLO | AP005329.1 | 0.562033835 | 1.11E-93 | positive |
| BRAF | AP005329.1 | 0.486839108 | 2.68E-67 | positive |
| CYLD | VIM-AS1 | 0.402768755 | 1.17E-44 | positive |
| DIABLO | AL354979.1 | 0.447075987 | 8.52E-56 | positive |
| OTULIN | AC010632.2 | 0.417024353 | 4.63E-48 | positive |
| CYLD | AC010632.2 | 0.448869126 | 2.78E-56 | positive |
| DIABLO | AC010632.2 | 0.777840524 | 2.37E-226 | positive |
| CFLAR | AC010632.2 | 0.417512935 | 3.52E-48 | positive |
| BRAF | AC010632.2 | 0.682214961 | 2.86E-153 | positive |
| ATRX | AC010632.2 | 0.545146095 | 3.65E-87 | positive |
| OTULIN | AC009159.2 | 0.44211666 | 1.83E-54 | positive |
| CYLD | AC009159.2 | 0.479689937 | 4.08E-65 | positive |
| DIABLO | AC009159.2 | 0.634327052 | 2.42E-126 | positive |
| BRAF | AC009159.2 | 0.634538933 | 1.88E-126 | positive |
| ATRX | AC009159.2 | 0.427137781 | 1.41E-50 | positive |
| TSC1 | BCDIN3D-AS1 | 0.449404659 | 1.99E-56 | positive |
| MAPK8 | BCDIN3D-AS1 | 0.429609683 | 3.32E-51 | positive |
| MAP3K7 | BCDIN3D-AS1 | 0.42136195 | 3.95E-49 | positive |
| DIABLO | BCDIN3D-AS1 | 0.565445968 | 4.79E-95 | positive |
| BRAF | BCDIN3D-AS1 | 0.557609598 | 6.13E-92 | positive |
| SIRT1 | BCDIN3D-AS1 | 0.405971702 | 2.09E-45 | positive |
| ATRX | BCDIN3D-AS1 | 0.488662376 | 7.31E-68 | positive |
| OTULIN | AC073529.1 | 0.448512618 | 3.48E-56 | positive |
| MAP3K7 | AC073529.1 | 0.41426999 | 2.17E-47 | positive |
| DIABLO | AC073529.1 | 0.586884238 | 5.50E-104 | positive |
| BRAF | AC073529.1 | 0.51662269 | 5.80E-77 | positive |
| BACH2 | GIHCG | 0.483904954 | 2.14E-66 | positive |
| DIABLO | ELOA-AS1 | 0.60740508 | 3.42E-113 | positive |
| BRAF | ELOA-AS1 | 0.508604261 | 2.89E-74 | positive |
| ATRX | ELOA-AS1 | 0.44541925 | 2.39E-55 | positive |
| RNF31 | ELOA-AS1 | 0.486627642 | 3.12E-67 | positive |
| MYC | EMSLR | 0.598529463 | 3.96E-109 | positive |
| TSC1 | AL133367.1 | 0.40813876 | 6.41E-46 | positive |
| TSC1 | EBLN3P | 0.55696605 | 1.09E-91 | positive |
| MAPK8 | EBLN3P | 0.539095657 | 6.43E-85 | positive |
| IPMK | EBLN3P | 0.475079984 | 9.77E-64 | positive |
| OTULIN | EBLN3P | 0.442191506 | 1.74E-54 | positive |
| CYLD | EBLN3P | 0.430660942 | 1.79E-51 | positive |
| MAP3K7 | EBLN3P | 0.557960324 | 4.47E-92 | positive |
| DIABLO | EBLN3P | 0.415077776 | 1.38E-47 | positive |
| BRAF | EBLN3P | 0.544106272 | 8.94E-87 | positive |
| SIRT1 | EBLN3P | 0.438944454 | 1.26E-53 | positive |
| ATRX | EBLN3P | 0.408365578 | 5.66E-46 | positive |
| MAPK8 | AL078459.1 | 0.400523164 | 3.90E-44 | positive |
| OTULIN | AL078459.1 | 0.43432712 | 2.03E-52 | positive |
| CYLD | AL078459.1 | 0.402002269 | 1.77E-44 | positive |
| DIABLO | AL078459.1 | 0.791844011 | 3.58E-240 | positive |
| BRAF | AL078459.1 | 0.693908551 | 1.19E-160 | positive |
| ATRX | AL078459.1 | 0.423727491 | 1.02E-49 | positive |
| DIABLO | AL158837.1 | 0.416079738 | 7.88E-48 | positive |
| DIABLO | AL358394.2 | 0.429404568 | 3.75E-51 | positive |
| MAPK8 | AC114939.1 | 0.401271188 | 2.62E-44 | positive |
| IPMK | AC114939.1 | 0.43006651 | 2.54E-51 | positive |
| OTULIN | AC114939.1 | 0.431868136 | 8.76E-52 | positive |
| CYLD | AC114939.1 | 0.462411484 | 4.72E-60 | positive |
| DIABLO | AC114939.1 | 0.732534543 | 8.89E-188 | positive |
| CFLAR | AC114939.1 | 0.466527937 | 3.12E-61 | positive |
| BRAF | AC114939.1 | 0.677133134 | 3.61E-150 | positive |
| ATRX | AC114939.1 | 0.523889723 | 1.80E-79 | positive |
| DIABLO | AL392089.1 | 0.568717537 | 2.28E-96 | positive |
| BRAF | AL392089.1 | 0.452962035 | 2.10E-57 | positive |
| DIABLO | AC005529.1 | 0.568553503 | 2.66E-96 | positive |
| BRAF | AC005529.1 | 0.507986254 | 4.63E-74 | positive |
| ATRX | AC005529.1 | 0.437722701 | 2.64E-53 | positive |
| DIABLO | AC011468.5 | 0.593974919 | 4.31E-107 | positive |
| BRAF | AC011468.5 | 0.566649157 | 1.57E-95 | positive |
| ATRX | AC011468.5 | 0.419779428 | 9.75E-49 | positive |
| AXL | AL161785.1 | 0.512985798 | 9.90E-76 | positive |
| DIABLO | AL121989.1 | 0.747791724 | 7.68E-200 | positive |
| BRAF | AL121989.1 | 0.63832186 | 2.09E-128 | positive |
| ATRX | AL121989.1 | 0.44264299 | 1.32E-54 | positive |
| DIABLO | GABPB1-AS1 | 0.537847973 | 1.84E-84 | positive |
| BRAF | GABPB1-AS1 | 0.491962867 | 6.81E-69 | positive |
| MAPK8 | AC048344.4 | 0.41516744 | 1.31E-47 | positive |
| OTULIN | AC048344.4 | 0.426145609 | 2.51E-50 | positive |
| MAP3K7 | AC048344.4 | 0.421486857 | 3.68E-49 | positive |
| DIABLO | AC048344.4 | 0.628626478 | 1.89E-123 | positive |
| BRAF | AC048344.4 | 0.622765465 | 1.54E-120 | positive |
| ATRX | AC048344.4 | 0.418083183 | 2.55E-48 | positive |
| DIABLO | AC091435.2 | 0.416664902 | 5.67E-48 | positive |
| TSC1 | AL157893.2 | 0.420762491 | 5.57E-49 | positive |
| CYLD | AL157893.2 | 0.424590619 | 6.18E-50 | positive |
| DIABLO | AL157893.2 | 0.408715803 | 4.68E-46 | positive |
| CFLAR | AL157893.2 | 0.420685752 | 5.82E-49 | positive |
| ATRX | AL157893.2 | 0.464287991 | 1.37E-60 | positive |
| RNF31 | AL157893.2 | 0.448511084 | 3.48E-56 | positive |
| DIABLO | RASGRF2-AS1 | 0.517044793 | 4.16E-77 | positive |
| BRAF | RASGRF2-AS1 | 0.466058268 | 4.26E-61 | positive |
| DIABLO | HIF1A-AS3 | 0.525284462 | 5.86E-80 | positive |
| BRAF | HIF1A-AS3 | 0.483259736 | 3.37E-66 | positive |
| DIABLO | AC003101.1 | 0.647293056 | 3.73E-133 | positive |
| BRAF | AC003101.1 | 0.518109545 | 1.80E-77 | positive |
| ATRX | FAM157C | 0.425207712 | 4.33E-50 | positive |
| RNF31 | FAM157C | 0.412856135 | 4.77E-47 | positive |
| OTULIN | SMAD1-AS2 | 0.426181775 | 2.46E-50 | positive |
| CYLD | SMAD1-AS2 | 0.432433555 | 6.27E-52 | positive |
| DIABLO | SMAD1-AS2 | 0.761501645 | 1.90E-211 | positive |
| BRAF | SMAD1-AS2 | 0.671786991 | 5.66E-147 | positive |
| DIABLO | AL158214.2 | 0.522737309 | 4.55E-79 | positive |
| BRAF | AL158214.2 | 0.442814489 | 1.19E-54 | positive |
| DIABLO | PLCH1-AS2 | 0.548584956 | 1.84E-88 | positive |
| BRAF | PLCH1-AS2 | 0.472897996 | 4.32E-63 | positive |
| ATRX | PLCH1-AS2 | 0.400111231 | 4.86E-44 | positive |
| BRAF | AC009902.3 | 0.420899821 | 5.15E-49 | positive |
| OTULIN | AC093297.2 | 0.457740697 | 9.85E-59 | positive |
| MAP3K7 | AC093297.2 | 0.413324949 | 3.67E-47 | positive |
| BRAF | AC093297.2 | 0.428337242 | 7.00E-51 | positive |
| TARDBP | AC093297.2 | 0.416802514 | 5.25E-48 | positive |
| RIPK3 | AC004477.2 | -0.412640055 | 5.38E-47 | negative |
| TSC1 | AC004477.2 | 0.445720283 | 1.98E-55 | positive |
| OTULIN | AC004477.2 | 0.431093741 | 1.39E-51 | positive |
| CYLD | AC004477.2 | 0.450787545 | 8.32E-57 | positive |
| DIABLO | AC004477.2 | 0.514487103 | 3.08E-76 | positive |
| CFLAR | AC004477.2 | 0.417021334 | 4.64E-48 | positive |
| BRAF | AC004477.2 | 0.591795741 | 3.95E-106 | positive |
| MPG | AC004477.2 | -0.440666894 | 4.43E-54 | negative |
| ATRX | AC004477.2 | 0.655929682 | 7.03E-138 | positive |
| TSC1 | AL078581.4 | 0.44531208 | 2.55E-55 | positive |
| MAPK8 | AL078581.4 | 0.434687949 | 1.64E-52 | positive |
| OTULIN | AL078581.4 | 0.463656096 | 2.08E-60 | positive |
| CYLD | AL078581.4 | 0.467702537 | 1.43E-61 | positive |
| DIABLO | AL078581.4 | 0.795360943 | 8.20E-244 | positive |
| CFLAR | AL078581.4 | 0.495679639 | 4.56E-70 | positive |
| BRAF | AL078581.4 | 0.705401638 | 2.93E-168 | positive |
| ATRX | AL078581.4 | 0.549587409 | 7.65E-89 | positive |
| RNF31 | AL078581.4 | 0.422145885 | 2.52E-49 | positive |
| TSC1 | AC073957.3 | 0.407615136 | 8.53E-46 | positive |
| TARDBP | SNHG1 | 0.490543093 | 1.90E-68 | positive |
| TSC1 | AC012467.2 | 0.459333444 | 3.51E-59 | positive |
| MAPK8 | AC012467.2 | 0.484588064 | 1.32E-66 | positive |
| MAP3K7 | AC012467.2 | 0.435219472 | 1.19E-52 | positive |
| BRAF | AC012467.2 | 0.402236639 | 1.56E-44 | positive |
| TSC1 | AC012020.1 | 0.404575264 | 4.44E-45 | positive |
| CYLD | AC012020.1 | 0.462620484 | 4.11E-60 | positive |
| DIABLO | AC012020.1 | 0.592217078 | 2.58E-106 | positive |
| CFLAR | AC012020.1 | 0.508078954 | 4.31E-74 | positive |
| BRAF | AC012020.1 | 0.540889952 | 1.40E-85 | positive |
| ATRX | AC012020.1 | 0.54060403 | 1.79E-85 | positive |
| OTULIN | AC034102.8 | 0.414410686 | 2.01E-47 | positive |
| CYLD | AC034102.8 | 0.419667868 | 1.04E-48 | positive |
| DIABLO | AC034102.8 | 0.700075942 | 1.09E-164 | positive |
| BRAF | AC034102.8 | 0.621050704 | 1.07E-119 | positive |
| TSC1 | AL139423.2 | 0.487024995 | 2.35E-67 | positive |
| MAPK8 | AL139423.2 | 0.429974542 | 2.68E-51 | positive |
| OTULIN | AL139423.2 | 0.458917724 | 4.60E-59 | positive |
| CYLD | AL139423.2 | 0.504854403 | 4.98E-73 | positive |
| DIABLO | AL139423.2 | 0.751364225 | 8.59E-203 | positive |
| CFLAR | AL139423.2 | 0.516610706 | 5.85E-77 | positive |
| BRAF | AL139423.2 | 0.712023558 | 8.20E-173 | positive |
| ATRX | AL139423.2 | 0.571832664 | 1.22E-97 | positive |
| RNF31 | AL139423.2 | 0.46979097 | 3.52E-62 | positive |
| OTULIN | AC118555.1 | 0.446520014 | 1.20E-55 | positive |
| DIABLO | AC118555.1 | 0.76375494 | 1.97E-213 | positive |
| BRAF | AC118555.1 | 0.620289178 | 2.52E-119 | positive |
| OTULIN | AL360091.2 | 0.442712225 | 1.27E-54 | positive |
| CYLD | AL360091.2 | 0.416601346 | 5.88E-48 | positive |
| DIABLO | AL360091.2 | 0.762682053 | 1.75E-212 | positive |
| BRAF | AL360091.2 | 0.659662345 | 5.70E-140 | positive |
| ATRX | AL360091.2 | 0.419674931 | 1.03E-48 | positive |
| TNF | LINC02701 | 0.424196621 | 7.76E-50 | positive |
| DIABLO | AC087276.3 | 0.702654206 | 2.09E-166 | positive |
| BRAF | AC087276.3 | 0.602465585 | 6.47E-111 | positive |
| ATRX | AC087276.3 | 0.47057498 | 2.08E-62 | positive |
| DIABLO | BET1-AS1 | 0.432008299 | 8.06E-52 | positive |
| BRAF | BET1-AS1 | 0.45351888 | 1.47E-57 | positive |
| DIABLO | AC005062.1 | 0.649399 | 2.72E-134 | positive |
| BRAF | AC005062.1 | 0.555905911 | 2.83E-91 | positive |
| ATRX | AC005062.1 | 0.416161038 | 7.53E-48 | positive |
| DIABLO | AC011476.2 | 0.502034029 | 4.14E-72 | positive |
| KLF9 | AC015922.2 | 0.471638816 | 1.01E-62 | positive |
| CFLAR | AC068491.2 | 0.44708666 | 8.47E-56 | positive |
| BRAF | AC068491.2 | 0.420010157 | 8.55E-49 | positive |
| ATRX | AC068491.2 | 0.577523584 | 5.31E-100 | positive |
| RNF31 | AC068491.2 | 0.464132872 | 1.52E-60 | positive |
| DIABLO | AL031673.1 | 0.426557493 | 1.98E-50 | positive |
| BRAF | AL031673.1 | 0.440800637 | 4.08E-54 | positive |
| ATRX | AL031673.1 | 0.411485429 | 1.02E-46 | positive |
| CYLD | AC011466.1 | 0.499348142 | 3.06E-71 | positive |
| DIABLO | AC011466.1 | 0.589636056 | 3.50E-105 | positive |
| CFLAR | AC011466.1 | 0.514237096 | 3.74E-76 | positive |
| BRAF | AC011466.1 | 0.59245592 | 2.02E-106 | positive |
| ATRX | AC011466.1 | 0.587359757 | 3.42E-104 | positive |
| OTULIN | CARS1-AS1 | 0.467665101 | 1.46E-61 | positive |
| CYLD | CARS1-AS1 | 0.444759004 | 3.59E-55 | positive |
| DIABLO | CARS1-AS1 | 0.812915933 | 4.11E-263 | positive |
| CFLAR | CARS1-AS1 | 0.413963419 | 2.57E-47 | positive |
| BRAF | CARS1-AS1 | 0.703273752 | 8.01E-167 | positive |
| ATRX | CARS1-AS1 | 0.410351605 | 1.90E-46 | positive |
| MAPK8 | FKBP14-AS1 | 0.440745084 | 4.22E-54 | positive |
| IPMK | FKBP14-AS1 | 0.462599075 | 4.17E-60 | positive |
| OTULIN | FKBP14-AS1 | 0.438185078 | 2.00E-53 | positive |
| CYLD | FKBP14-AS1 | 0.428271911 | 7.27E-51 | positive |
| MAP3K7 | FKBP14-AS1 | 0.428097215 | 8.06E-51 | positive |
| DIABLO | FKBP14-AS1 | 0.70841207 | 2.59E-170 | positive |
| CFLAR | FKBP14-AS1 | 0.470896335 | 1.67E-62 | positive |
| BRAF | FKBP14-AS1 | 0.655877756 | 7.51E-138 | positive |
| ATRX | FKBP14-AS1 | 0.529709776 | 1.60E-81 | positive |
| DIABLO | ZNRF3-IT1 | 0.539355389 | 5.16E-85 | positive |
| BRAF | ZNRF3-IT1 | 0.465538252 | 6.01E-61 | positive |
| DIABLO | AC008667.1 | 0.496666771 | 2.21E-70 | positive |
| MAPK8 | AL133243.3 | 0.454520869 | 7.78E-58 | positive |
| IPMK | AL133243.3 | 0.43686157 | 4.44E-53 | positive |
| OTULIN | AL133243.3 | 0.503436608 | 1.45E-72 | positive |
| CYLD | AL133243.3 | 0.506485909 | 1.45E-73 | positive |
| MAP3K7 | AL133243.3 | 0.430612506 | 1.84E-51 | positive |
| DIABLO | AL133243.3 | 0.842342807 | 1.57E-300 | positive |
| CFLAR | AL133243.3 | 0.492641027 | 4.17E-69 | positive |
| BRAF | AL133243.3 | 0.760810357 | 7.62E-211 | positive |
| ATRX | AL133243.3 | 0.579204746 | 1.05E-100 | positive |
| OTULIN | ARNTL2-AS1 | 0.490034178 | 2.74E-68 | positive |
| CYLD | ARNTL2-AS1 | 0.481773187 | 9.55E-66 | positive |
| DIABLO | ARNTL2-AS1 | 0.716001799 | 1.30E-175 | positive |
| CFLAR | ARNTL2-AS1 | 0.424576965 | 6.23E-50 | positive |
| BRAF | ARNTL2-AS1 | 0.647619197 | 2.49E-133 | positive |
| ATRX | ARNTL2-AS1 | 0.445711468 | 1.99E-55 | positive |
| DIABLO | TAX1BP1-AS1 | 0.583648516 | 1.36E-102 | positive |
| BRAF | TAX1BP1-AS1 | 0.50286973 | 2.22E-72 | positive |
| OTULIN | AC006270.1 | 0.427869845 | 9.20E-51 | positive |
| DIABLO | AC006270.1 | 0.659007625 | 1.33E-139 | positive |
| BRAF | AC006270.1 | 0.601809482 | 1.29E-110 | positive |
| TSC1 | RTCA-AS1 | 0.449588755 | 1.77E-56 | positive |
| CYLD | RTCA-AS1 | 0.435324769 | 1.12E-52 | positive |
| MAP3K7 | RTCA-AS1 | 0.430812822 | 1.63E-51 | positive |
| DIABLO | RTCA-AS1 | 0.43781815 | 2.50E-53 | positive |
| CFLAR | RTCA-AS1 | 0.494903325 | 8.04E-70 | positive |
| BRAF | RTCA-AS1 | 0.519903647 | 4.35E-78 | positive |
| ATRX | RTCA-AS1 | 0.626565672 | 2.03E-122 | positive |
| KLF9 | RTCA-AS1 | 0.477590376 | 1.74E-64 | positive |
| DIABLO | AC130469.1 | 0.444476555 | 4.27E-55 | positive |
| OTULIN | AL136984.1 | 0.452858971 | 2.24E-57 | positive |
| CYLD | AL136984.1 | 0.450563223 | 9.59E-57 | positive |
| DIABLO | AL136984.1 | 0.827164058 | 2.42E-280 | positive |
| BRAF | AL136984.1 | 0.717878636 | 6.00E-177 | positive |
| ATRX | AL136984.1 | 0.413373281 | 3.58E-47 | positive |
| DIABLO | XXYLT1-AS1 | 0.426442901 | 2.11E-50 | positive |
| DIABLO | AP001775.2 | 0.545001624 | 4.13E-87 | positive |
| BRAF | AP001775.2 | 0.502586423 | 2.74E-72 | positive |
| ATRX | AP001775.2 | 0.452676422 | 2.52E-57 | positive |
| MAPK8 | AC079921.1 | 0.41567123 | 9.91E-48 | positive |
| IPMK | AC079921.1 | 0.401890396 | 1.88E-44 | positive |
| OTULIN | AC079921.1 | 0.498611749 | 5.27E-71 | positive |
| CYLD | AC079921.1 | 0.540347111 | 2.23E-85 | positive |
| MAP3K7 | AC079921.1 | 0.402179571 | 1.61E-44 | positive |
| DIABLO | AC079921.1 | 0.765486679 | 5.70E-215 | positive |
| CFLAR | AC079921.1 | 0.477648041 | 1.67E-64 | positive |
| BRAF | AC079921.1 | 0.743360987 | 3.00E-196 | positive |
| ATRX | AC079921.1 | 0.513773524 | 5.37E-76 | positive |
| DIABLO | LINC02848 | 0.450544745 | 9.70E-57 | positive |
| TSC1 | C9orf147 | 0.408965582 | 4.08E-46 | positive |
| DIABLO | C9orf147 | 0.648015933 | 1.52E-133 | positive |
| BRAF | C9orf147 | 0.587158315 | 4.19E-104 | positive |
| ATRX | C9orf147 | 0.460954731 | 1.22E-59 | positive |
| OTULIN | AC027279.2 | 0.430948342 | 1.51E-51 | positive |
| CYLD | AC027279.2 | 0.420217619 | 7.60E-49 | positive |
| DIABLO | AC027279.2 | 0.714761272 | 9.84E-175 | positive |
| BRAF | AC027279.2 | 0.656808875 | 2.28E-138 | positive |
| ATRX | AC027279.2 | 0.490666056 | 1.74E-68 | positive |
| DIABLO | AP006623.1 | 0.406960198 | 1.22E-45 | positive |
| RNF31 | AP006623.1 | 0.450783141 | 8.34E-57 | positive |
| ATRX | AC136469.1 | 0.477736291 | 1.58E-64 | positive |
| TSC1 | AC002128.1 | 0.473810165 | 2.32E-63 | positive |
| MAPK8 | AC002128.1 | 0.417192927 | 4.21E-48 | positive |
| OTULIN | AC002128.1 | 0.457718963 | 9.99E-59 | positive |
| CYLD | AC002128.1 | 0.418074715 | 2.56E-48 | positive |
| DIABLO | AC002128.1 | 0.690719537 | 1.33E-158 | positive |
| CFLAR | AC002128.1 | 0.496879791 | 1.89E-70 | positive |
| BRAF | AC002128.1 | 0.626135185 | 3.33E-122 | positive |
| ATRX | AC002128.1 | 0.484052646 | 1.93E-66 | positive |
| RNF31 | AC002128.1 | 0.458675658 | 5.38E-59 | positive |
| DIABLO | AC025165.4 | 0.440772038 | 4.15E-54 | positive |
| TSC1 | AC018809.2 | 0.482970377 | 4.13E-66 | positive |
| MAPK8 | AC018809.2 | 0.412099683 | 7.25E-47 | positive |
| OTULIN | AC018809.2 | 0.4175708 | 3.40E-48 | positive |
| DIABLO | AC018809.2 | 0.604998582 | 4.45E-112 | positive |
| CFLAR | AC018809.2 | 0.411716234 | 8.97E-47 | positive |
| BRAF | AC018809.2 | 0.592835159 | 1.38E-106 | positive |
| ATRX | AC018809.2 | 0.465970804 | 4.51E-61 | positive |
| OTULIN | AC064874.1 | 0.444308296 | 4.74E-55 | positive |
| DIABLO | AC064874.1 | 0.79274558 | 4.24E-241 | positive |
| BRAF | AC064874.1 | 0.662176978 | 2.14E-141 | positive |
| MAP3K7 | DNAJC3-DT | 0.401819332 | 1.95E-44 | positive |
| BACH2 | AL590609.2 | 0.411602272 | 9.55E-47 | positive |
| CYLD | PRKX-AS1 | 0.402807949 | 1.15E-44 | positive |
| DIABLO | PRKX-AS1 | 0.734315221 | 3.84E-189 | positive |
| BRAF | PRKX-AS1 | 0.607141042 | 4.53E-113 | positive |
| ATRX | PRKX-AS1 | 0.424961684 | 4.99E-50 | positive |
| DIABLO | AL358937.1 | 0.472632957 | 5.17E-63 | positive |
| BRAF | AL358937.1 | 0.434601732 | 1.72E-52 | positive |
| OTULIN | NCK1-DT | 0.460182303 | 2.02E-59 | positive |
| CYLD | NCK1-DT | 0.477197538 | 2.28E-64 | positive |
| MAP3K7 | NCK1-DT | 0.467371618 | 1.78E-61 | positive |
| DIABLO | NCK1-DT | 0.544536075 | 6.17E-87 | positive |
| BRAF | NCK1-DT | 0.546621088 | 1.02E-87 | positive |
| ATRX | NCK1-DT | 0.569679097 | 9.27E-97 | positive |
| DIABLO | LINC00244 | 0.587217187 | 3.95E-104 | positive |
| BRAF | LINC00244 | 0.510274693 | 8.02E-75 | positive |
| DIABLO | AL031432.5 | 0.473562531 | 2.75E-63 | positive |
| ATRX | AL031432.5 | 0.417678642 | 3.20E-48 | positive |
| CYLD | AC006116.4 | 0.419975184 | 8.72E-49 | positive |
| DIABLO | AC006116.4 | 0.590914126 | 9.65E-106 | positive |
| CFLAR | AC006116.4 | 0.415084737 | 1.38E-47 | positive |
| BRAF | AC006116.4 | 0.563661326 | 2.49E-94 | positive |
| ATRX | AC006116.4 | 0.449484287 | 1.89E-56 | positive |
| DIABLO | AP000254.2 | 0.405803408 | 2.29E-45 | positive |
| DIABLO | AC102945.1 | 0.495803979 | 4.16E-70 | positive |
| BRAF | AC102945.1 | 0.424075338 | 8.33E-50 | positive |
| ATRX | AC102945.1 | 0.40847495 | 5.33E-46 | positive |
| DIABLO | AC084757.2 | 0.673888416 | 3.20E-148 | positive |
| BRAF | AC084757.2 | 0.585398912 | 2.41E-103 | positive |
| ATRX | AC084757.2 | 0.457749099 | 9.79E-59 | positive |
| BCL2 | AC107464.2 | 0.416068058 | 7.93E-48 | positive |
| BCL2L11 | AC107464.2 | 0.45476308 | 6.67E-58 | positive |
| MAPK8 | KANSL1L-AS1 | 0.415811895 | 9.16E-48 | positive |
| IPMK | KANSL1L-AS1 | 0.404161827 | 5.55E-45 | positive |
| OTULIN | KANSL1L-AS1 | 0.406643576 | 1.45E-45 | positive |
| CYLD | KANSL1L-AS1 | 0.419555551 | 1.11E-48 | positive |
| DIABLO | KANSL1L-AS1 | 0.698841866 | 7.15E-164 | positive |
| CFLAR | KANSL1L-AS1 | 0.44104064 | 3.53E-54 | positive |
| BRAF | KANSL1L-AS1 | 0.675244593 | 4.94E-149 | positive |
| ATRX | KANSL1L-AS1 | 0.520956608 | 1.88E-78 | positive |
| DIABLO | AC012645.2 | 0.586757689 | 6.24E-104 | positive |
| TSC1 | AC012531.1 | 0.403382678 | 8.45E-45 | positive |
| ATRX | AC012531.1 | 0.428957263 | 4.87E-51 | positive |
| DIABLO | HCFC1-AS1 | 0.495256715 | 6.21E-70 | positive |
| BRAF | HCFC1-AS1 | 0.42407083 | 8.35E-50 | positive |
| RNF31 | AC139887.1 | 0.411621679 | 9.45E-47 | positive |
| CYLD | AL357140.4 | 0.413898173 | 2.67E-47 | positive |
| DIABLO | AL357140.4 | 0.687113032 | 2.56E-156 | positive |
| CFLAR | AL357140.4 | 0.423209345 | 1.37E-49 | positive |
| BRAF | AL357140.4 | 0.592060017 | 3.02E-106 | positive |
| ATRX | AL357140.4 | 0.505937573 | 2.19E-73 | positive |
| DIABLO | ARHGEF38-IT1 | 0.720379475 | 9.54E-179 | positive |
| BRAF | ARHGEF38-IT1 | 0.582694842 | 3.47E-102 | positive |
| ATRX | FAM215B | 0.400282717 | 4.43E-44 | positive |
| DIABLO | AC017076.1 | 0.439401564 | 9.57E-54 | positive |
| BRAF | AC017076.1 | 0.421609355 | 3.43E-49 | positive |
| DIABLO | AL583824.1 | 0.755577326 | 2.45E-206 | positive |
| CFLAR | AL583824.1 | 0.411918032 | 8.02E-47 | positive |
| BRAF | AL583824.1 | 0.620404633 | 2.21E-119 | positive |
| DIABLO | C1orf195 | 0.78777241 | 4.79E-236 | positive |
| CFLAR | C1orf195 | 0.451706634 | 4.66E-57 | positive |
| BRAF | C1orf195 | 0.666487613 | 7.15E-144 | positive |
| ATRX | C1orf195 | 0.475679239 | 6.48E-64 | positive |
| MAPK8 | CDC42-IT1 | 0.412516708 | 5.76E-47 | positive |
| IPMK | CDC42-IT1 | 0.428244612 | 7.39E-51 | positive |
| OTULIN | CDC42-IT1 | 0.424316696 | 7.24E-50 | positive |
| CYLD | CDC42-IT1 | 0.448535854 | 3.43E-56 | positive |
| DIABLO | CDC42-IT1 | 0.819870864 | 2.48E-271 | positive |
| CFLAR | CDC42-IT1 | 0.469470697 | 4.36E-62 | positive |
| BRAF | CDC42-IT1 | 0.720698243 | 5.61E-179 | positive |
| ATRX | CDC42-IT1 | 0.546178888 | 1.49E-87 | positive |
| DIABLO | LINC02516 | 0.526585361 | 2.04E-80 | positive |
| BRAF | LINC02516 | 0.492020712 | 6.53E-69 | positive |
| DIABLO | THBS4-AS1 | 0.43144846 | 1.12E-51 | positive |
| BRAF | THBS4-AS1 | 0.459131139 | 4.00E-59 | positive |
| OTULIN | AL365356.1 | 0.468719027 | 7.22E-62 | positive |
| CYLD | AL365356.1 | 0.425416021 | 3.84E-50 | positive |
| DIABLO | AL365356.1 | 0.782331671 | 1.13E-230 | positive |
| BRAF | AL365356.1 | 0.690629449 | 1.52E-158 | positive |
| ATRX | AL365356.1 | 0.40756592 | 8.77E-46 | positive |
| MAPK8 | AC007619.1 | 0.429307681 | 3.96E-51 | positive |
| IPMK | AC007619.1 | 0.407411291 | 9.54E-46 | positive |
| OTULIN | AC007619.1 | 0.425920962 | 2.86E-50 | positive |
| CYLD | AC007619.1 | 0.500788662 | 1.05E-71 | positive |
| DIABLO | AC007619.1 | 0.763903133 | 1.46E-213 | positive |
| CFLAR | AC007619.1 | 0.510143959 | 8.87E-75 | positive |
| BRAF | AC007619.1 | 0.744810955 | 2.04E-197 | positive |
| ATRX | AC007619.1 | 0.631767805 | 4.90E-125 | positive |
| MAPK8 | AC079907.1 | 0.423286592 | 1.31E-49 | positive |
| OTULIN | AC079907.1 | 0.415078443 | 1.38E-47 | positive |
| MAP3K7 | AC079907.1 | 0.413109737 | 4.14E-47 | positive |
| DIABLO | AC079907.1 | 0.542822978 | 2.69E-86 | positive |
| BRAF | AC079907.1 | 0.47527662 | 8.54E-64 | positive |
| TSC1 | AL049840.2 | 0.468674384 | 7.44E-62 | positive |
| MAPK8 | AL049840.2 | 0.419863083 | 9.29E-49 | positive |
| OTULIN | AL049840.2 | 0.5056376 | 2.75E-73 | positive |
| CYLD | AL049840.2 | 0.510311281 | 7.80E-75 | positive |
| MAP3K7 | AL049840.2 | 0.406021973 | 2.03E-45 | positive |
| DIABLO | AL049840.2 | 0.755577054 | 2.45E-206 | positive |
| CFLAR | AL049840.2 | 0.515867234 | 1.05E-76 | positive |
| BRAF | AL049840.2 | 0.705530418 | 2.40E-168 | positive |
| ATRX | AL049840.2 | 0.531351833 | 4.14E-82 | positive |
| RNF31 | AL049840.2 | 0.432525242 | 5.93E-52 | positive |
| DIABLO | AC011139.1 | 0.48417298 | 1.77E-66 | positive |
| OTULIN | AC010978.1 | 0.452071595 | 3.70E-57 | positive |
| CYLD | AC010978.1 | 0.423249489 | 1.34E-49 | positive |
| DIABLO | AC010978.1 | 0.803213725 | 3.33E-252 | positive |
| BRAF | AC010978.1 | 0.699041817 | 5.28E-164 | positive |
| ATRX | AC010978.1 | 0.415930693 | 8.57E-48 | positive |
| OTULIN | AC253576.2 | 0.404130182 | 5.65E-45 | positive |
| DIABLO | AC253576.2 | 0.590808005 | 1.07E-105 | positive |
| BRAF | AC253576.2 | 0.559327835 | 1.30E-92 | positive |
| ATRX | AC253576.2 | 0.453034722 | 2.01E-57 | positive |
| DIABLO | AC108704.1 | 0.459602027 | 2.95E-59 | positive |
| BRAF | AC108704.1 | 0.410348085 | 1.91E-46 | positive |
| OTULIN | AC007731.3 | 0.430871737 | 1.58E-51 | positive |
| CYLD | AC007731.3 | 0.426664257 | 1.86E-50 | positive |
| DIABLO | AC007731.3 | 0.800737967 | 1.62E-249 | positive |
| CFLAR | AC007731.3 | 0.415881146 | 8.81E-48 | positive |
| BRAF | AC007731.3 | 0.660537199 | 1.83E-140 | positive |
| ATRX | AC007731.3 | 0.422732044 | 1.80E-49 | positive |
| TSC1 | AC008750.4 | 0.438348156 | 1.81E-53 | positive |
| DIABLO | AC008750.4 | 0.461315037 | 9.67E-60 | positive |
| CFLAR | AC008750.4 | 0.426555367 | 1.98E-50 | positive |
| BRAF | AC008750.4 | 0.457508617 | 1.14E-58 | positive |
| ATRX | AC008750.4 | 0.555616001 | 3.67E-91 | positive |
| RNF31 | AC008750.4 | 0.456693618 | 1.93E-58 | positive |
| CD40 | AL138724.1 | 0.436310366 | 6.19E-53 | positive |
| DIABLO | LINC02728 | 0.535327694 | 1.53E-83 | positive |
| BRAF | LINC02728 | 0.507545006 | 6.47E-74 | positive |
| TSC1 | AC034102.6 | 0.458586965 | 5.70E-59 | positive |
| DIABLO | AC034102.6 | 0.477807895 | 1.50E-64 | positive |
| CFLAR | AC034102.6 | 0.410550379 | 1.71E-46 | positive |
| BRAF | AC034102.6 | 0.461013192 | 1.18E-59 | positive |
| ATRX | AC034102.6 | 0.403103955 | 9.81E-45 | positive |
| RNF31 | AC034102.6 | 0.440430656 | 5.12E-54 | positive |
| DIABLO | AC106858.1 | 0.51525448 | 1.69E-76 | positive |
| BRAF | AC106858.1 | 0.50623281 | 1.75E-73 | positive |
| ATRX | AC106858.1 | 0.45006824 | 1.31E-56 | positive |
| OTULIN | AC018946.1 | 0.40088926 | 3.21E-44 | positive |
| CYLD | AC018946.1 | 0.418378845 | 2.16E-48 | positive |
| DIABLO | AC018946.1 | 0.769999943 | 4.80E-219 | positive |
| BRAF | AC018946.1 | 0.668924013 | 2.73E-145 | positive |
| ATRX | AC018946.1 | 0.425364192 | 3.95E-50 | positive |
| CYLD | AC091053.2 | 0.43013342 | 2.44E-51 | positive |
| DIABLO | AC091053.2 | 0.751185112 | 1.21E-202 | positive |
| CFLAR | AC091053.2 | 0.444753486 | 3.60E-55 | positive |
| BRAF | AC091053.2 | 0.645312872 | 4.30E-132 | positive |
| ATRX | AC091053.2 | 0.485005354 | 9.85E-67 | positive |
| OTULIN | ZBTB20-AS4 | 0.417780481 | 3.02E-48 | positive |
| CYLD | ZBTB20-AS4 | 0.410201778 | 2.07E-46 | positive |
| DIABLO | ZBTB20-AS4 | 0.722572165 | 2.43E-180 | positive |
| BRAF | ZBTB20-AS4 | 0.656203295 | 4.95E-138 | positive |
| ATRX | ZBTB20-AS4 | 0.40020487 | 4.62E-44 | positive |
| DIABLO | AL121583.1 | 0.478695651 | 8.12E-65 | positive |
| BRAF | AL121583.1 | 0.436455231 | 5.67E-53 | positive |
| DIABLO | AL049835.1 | 0.590523027 | 1.43E-105 | positive |
| BRAF | AL049835.1 | 0.53609059 | 8.07E-84 | positive |
| ATRX | AL049835.1 | 0.4786782 | 8.22E-65 | positive |
| CD40 | RFX5-AS1 | 0.449641064 | 1.71E-56 | positive |
| DIABLO | GPRC5D-AS1 | 0.509706114 | 1.24E-74 | positive |
| BRAF | GPRC5D-AS1 | 0.408133766 | 6.43E-46 | positive |
| MAP3K7 | AC027237.3 | 0.419750097 | 9.91E-49 | positive |
| DIABLO | AC027237.3 | 0.588707833 | 8.89E-105 | positive |
| BRAF | AC027237.3 | 0.559356988 | 1.26E-92 | positive |
| DIABLO | AUXG01000058.1 | 0.634170285 | 2.91E-126 | positive |
| BRAF | AUXG01000058.1 | 0.534726716 | 2.52E-83 | positive |
| ATRX | AC123768.1 | 0.433236496 | 3.89E-52 | positive |
| MAPK8 | AL359715.3 | 0.460131306 | 2.09E-59 | positive |
| IPMK | AL359715.3 | 0.437706286 | 2.67E-53 | positive |
| OTULIN | AL359715.3 | 0.433018198 | 4.43E-52 | positive |
| MAP3K7 | AL359715.3 | 0.49289983 | 3.46E-69 | positive |
| DIABLO | AL359715.3 | 0.638176276 | 2.49E-128 | positive |
| BRAF | AL359715.3 | 0.612219834 | 1.88E-115 | positive |
| ATRX | AL359715.3 | 0.421744166 | 3.18E-49 | positive |
| DIABLO | AC010768.2 | 0.553573633 | 2.27E-90 | positive |
| CFLAR | AC010768.2 | 0.469841143 | 3.40E-62 | positive |
| BRAF | AC010768.2 | 0.478848314 | 7.31E-65 | positive |
| ATRX | AC010768.2 | 0.46198362 | 6.24E-60 | positive |
| OTULIN | AL390236.1 | 0.425706546 | 3.24E-50 | positive |
| DIABLO | AL390236.1 | 0.701878144 | 6.90E-166 | positive |
| CFLAR | AL390236.1 | 0.427094738 | 1.45E-50 | positive |
| BRAF | AL390236.1 | 0.636467287 | 1.91E-127 | positive |
| ATRX | AL390236.1 | 0.40229306 | 1.52E-44 | positive |
| BRAF | AC104964.3 | 0.407983723 | 6.98E-46 | positive |
| ATRX | VPS13B-DT | 0.425295918 | 4.11E-50 | positive |
| OTULIN | AL442125.1 | 0.489183711 | 5.04E-68 | positive |
| CYLD | AL442125.1 | 0.414334066 | 2.09E-47 | positive |
| MAP3K7 | AL442125.1 | 0.434208142 | 2.18E-52 | positive |
| DIABLO | AL442125.1 | 0.679925774 | 7.27E-152 | positive |
| CFLAR | AL442125.1 | 0.409483715 | 3.07E-46 | positive |
| BRAF | AL442125.1 | 0.640725087 | 1.16E-129 | positive |
| MAPK8 | AC010320.4 | 0.41052716 | 1.73E-46 | positive |
| OTULIN | AC010320.4 | 0.474885864 | 1.12E-63 | positive |
| CYLD | AC010320.4 | 0.425287993 | 4.13E-50 | positive |
| DIABLO | AC010320.4 | 0.831219324 | 1.55E-285 | positive |
| BRAF | AC010320.4 | 0.708135179 | 4.01E-170 | positive |
| ATRX | AC010320.4 | 0.45717054 | 1.42E-58 | positive |
| OTULIN | AC020913.3 | 0.403457335 | 8.11E-45 | positive |
| CYLD | AC020913.3 | 0.412714399 | 5.16E-47 | positive |
| DIABLO | AC020913.3 | 0.674613671 | 1.18E-148 | positive |
| CFLAR | AC020913.3 | 0.451625121 | 4.90E-57 | positive |
| BRAF | AC020913.3 | 0.598779492 | 3.05E-109 | positive |
| ATRX | AC020913.3 | 0.436074982 | 7.13E-53 | positive |
| DIABLO | AL158825.2 | 0.591537165 | 5.14E-106 | positive |
| BRAF | AL158825.2 | 0.533184245 | 9.10E-83 | positive |
| ATRX | AL158825.2 | 0.500111306 | 1.74E-71 | positive |
| TSC1 | AC092910.3 | 0.437241802 | 3.53E-53 | positive |
| MAP3K7 | AC092910.3 | 0.405918315 | 2.15E-45 | positive |
| DIABLO | AL024498.1 | 0.507815679 | 5.27E-74 | positive |
| TNFSF10 | IL20RB-AS1 | 0.401300258 | 2.58E-44 | positive |
| CYLD | IL20RB-AS1 | 0.405414773 | 2.82E-45 | positive |
| OTULIN | AL591069.1 | 0.437227039 | 3.57E-53 | positive |
| DIABLO | AL591069.1 | 0.708650377 | 1.78E-170 | positive |
| BRAF | AL591069.1 | 0.626789901 | 1.57E-122 | positive |
| DIABLO | AC011773.1 | 0.615117778 | 7.88E-117 | positive |
| BRAF | AC011773.1 | 0.589738732 | 3.16E-105 | positive |
| BRAF | AC110597.1 | 0.437970446 | 2.28E-53 | positive |
| KLF9 | AC110597.1 | 0.435629733 | 9.31E-53 | positive |
| MAPK8 | AC116158.1 | 0.441470716 | 2.71E-54 | positive |
| IPMK | AC116158.1 | 0.426746495 | 1.77E-50 | positive |
| OTULIN | AC116158.1 | 0.483778497 | 2.34E-66 | positive |
| CYLD | AC116158.1 | 0.559928385 | 7.53E-93 | positive |
| MAP3K7 | AC116158.1 | 0.404200576 | 5.44E-45 | positive |
| DIABLO | AC116158.1 | 0.79746029 | 5.09E-246 | positive |
| CFLAR | AC116158.1 | 0.506746032 | 1.19E-73 | positive |
| BRAF | AC116158.1 | 0.745547562 | 5.17E-198 | positive |
| ATRX | AC116158.1 | 0.522712261 | 4.64E-79 | positive |
| DIABLO | AL035071.1 | 0.414630131 | 1.77E-47 | positive |
| DIABLO | KIAA1614-AS1 | 0.440101286 | 6.25E-54 | positive |
| OTULIN | ITGB5-AS1 | 0.471181825 | 1.38E-62 | positive |
| CYLD | ITGB5-AS1 | 0.410285147 | 1.98E-46 | positive |
| DIABLO | ITGB5-AS1 | 0.725401574 | 2.03E-182 | positive |
| BRAF | ITGB5-AS1 | 0.649494451 | 2.41E-134 | positive |
| DIABLO | AC011491.2 | 0.511674502 | 2.73E-75 | positive |
| BRAF | AC011491.2 | 0.405962611 | 2.10E-45 | positive |
| DIABLO | AC069272.1 | 0.556670557 | 1.43E-91 | positive |
| DIABLO | LINC01762 | 0.438206745 | 1.97E-53 | positive |
| BRAF | LINC01762 | 0.404801744 | 3.93E-45 | positive |
| DIABLO | AC010491.2 | 0.539065602 | 6.60E-85 | positive |
| BRAF | AC010491.2 | 0.534147481 | 4.09E-83 | positive |
| DIABLO | ST3GAL3-AS1 | 0.598111469 | 6.11E-109 | positive |
| BRAF | ST3GAL3-AS1 | 0.511143941 | 4.11E-75 | positive |
| ATRX | ST3GAL3-AS1 | 0.420735321 | 5.66E-49 | positive |
| DIABLO | AC064856.1 | 0.52095354 | 1.89E-78 | positive |
| BRAF | AC064856.1 | 0.508908555 | 2.29E-74 | positive |
| DIABLO | AL590422.1 | 0.55691729 | 1.14E-91 | positive |
| BRAF | AL590422.1 | 0.459228736 | 3.76E-59 | positive |
| MAPK8 | AC009299.2 | 0.421160418 | 4.44E-49 | positive |
| IPMK | AC009299.2 | 0.43893304 | 1.27E-53 | positive |
| OTULIN | AC009299.2 | 0.487184093 | 2.10E-67 | positive |
| CYLD | AC009299.2 | 0.457233893 | 1.37E-58 | positive |
| MAP3K7 | AC009299.2 | 0.417817615 | 2.96E-48 | positive |
| DIABLO | AC009299.2 | 0.735936209 | 2.15E-190 | positive |
| BRAF | AC009299.2 | 0.700958815 | 2.83E-165 | positive |
| ATRX | AC009299.2 | 0.448040671 | 4.67E-56 | positive |
| MAPK8 | AF230666.1 | 0.435463793 | 1.03E-52 | positive |
| IPMK | AF230666.1 | 0.443825985 | 6.39E-55 | positive |
| OTULIN | AF230666.1 | 0.462645557 | 4.05E-60 | positive |
| CYLD | AF230666.1 | 0.452607062 | 2.63E-57 | positive |
| MAP3K7 | AF230666.1 | 0.405024993 | 3.48E-45 | positive |
| DIABLO | AF230666.1 | 0.757297344 | 8.32E-208 | positive |
| BRAF | AF230666.1 | 0.707642692 | 8.72E-170 | positive |
| ATRX | AF230666.1 | 0.457357564 | 1.26E-58 | positive |
| TSC1 | NIPBL-DT | 0.479364121 | 5.11E-65 | positive |
| MAPK8 | NIPBL-DT | 0.421716221 | 3.23E-49 | positive |
| OTULIN | NIPBL-DT | 0.54131239 | 9.79E-86 | positive |
| CYLD | NIPBL-DT | 0.455623779 | 3.84E-58 | positive |
| MAP3K7 | NIPBL-DT | 0.47463003 | 1.33E-63 | positive |
| DIABLO | NIPBL-DT | 0.448027506 | 4.71E-56 | positive |
| BRAF | NIPBL-DT | 0.594026678 | 4.08E-107 | positive |
| ATRX | NIPBL-DT | 0.560954908 | 2.96E-93 | positive |
| RNF31 | NIPBL-DT | 0.40543769 | 2.79E-45 | positive |
| BCL2L11 | NIPBL-DT | 0.440902553 | 3.84E-54 | positive |
| DIABLO | AL161756.1 | 0.490924595 | 1.44E-68 | positive |
| BRAF | AL161756.1 | 0.406744496 | 1.37E-45 | positive |
| DIABLO | AC023855.1 | 0.653810633 | 1.05E-136 | positive |
| BRAF | AC023855.1 | 0.596927031 | 2.08E-108 | positive |
| ATRX | AC023855.1 | 0.409979169 | 2.34E-46 | positive |
| IPMK | AC107909.1 | 0.412451573 | 5.97E-47 | positive |
| OTULIN | AC107909.1 | 0.447003113 | 8.92E-56 | positive |
| CYLD | AC107909.1 | 0.422890218 | 1.65E-49 | positive |
| DIABLO | AC107909.1 | 0.738030291 | 5.02E-192 | positive |
| CFLAR | AC107909.1 | 0.42493681 | 5.06E-50 | positive |
| BRAF | AC107909.1 | 0.671864374 | 5.09E-147 | positive |
| ATRX | AC107909.1 | 0.525825891 | 3.78E-80 | positive |
| FASLG | AC007991.4 | 0.44436122 | 4.59E-55 | positive |
| TSC1 | AC109587.1 | 0.402750639 | 1.19E-44 | positive |
| MAPK8 | AC109587.1 | 0.488858836 | 6.36E-68 | positive |
| IPMK | AC109587.1 | 0.434679106 | 1.65E-52 | positive |
| OTULIN | AC109587.1 | 0.504560214 | 6.21E-73 | positive |
| CYLD | AC109587.1 | 0.580452048 | 3.11E-101 | positive |
| MAP3K7 | AC109587.1 | 0.507058637 | 9.37E-74 | positive |
| DIABLO | AC109587.1 | 0.784460112 | 9.35E-233 | positive |
| CFLAR | AC109587.1 | 0.526610438 | 2.00E-80 | positive |
| BRAF | AC109587.1 | 0.792813132 | 3.61E-241 | positive |
| ATRX | AC109587.1 | 0.561120521 | 2.55E-93 | positive |
| OTULIN | AC106799.1 | 0.478608911 | 8.62E-65 | positive |
| DIABLO | AC004584.1 | 0.549475418 | 8.44E-89 | positive |
| BRAF | AC004584.1 | 0.470506344 | 2.18E-62 | positive |
| DIABLO | LINC01429 | 0.59555508 | 8.54E-108 | positive |
| BRAF | LINC01429 | 0.504292718 | 7.60E-73 | positive |
| TNFRSF1A | AC006206.2 | 0.460462509 | 1.69E-59 | positive |
| EGFR | AC006206.2 | 0.5197807 | 4.80E-78 | positive |
| DIABLO | AC211476.2 | 0.782012653 | 2.32E-230 | positive |
| BRAF | AC211476.2 | 0.64633522 | 1.22E-132 | positive |
| ATRX | AC211476.2 | 0.42599652 | 2.74E-50 | positive |
| OTULIN | AC073592.1 | 0.410231619 | 2.03E-46 | positive |
| DIABLO | AC073592.1 | 0.775249141 | 6.65E-224 | positive |
| BRAF | AC073592.1 | 0.609206549 | 4.93E-114 | positive |
| AXL | AL359546.1 | 0.429981085 | 2.67E-51 | positive |
| DIABLO | LINC00427 | 0.41101 | 1.32E-46 | positive |
| TSC1 | AC006480.2 | 0.417774251 | 3.04E-48 | positive |
| MAPK8 | AC006480.2 | 0.440605512 | 4.60E-54 | positive |
| IPMK | AC006480.2 | 0.433355545 | 3.62E-52 | positive |
| OTULIN | AC006480.2 | 0.43859831 | 1.56E-53 | positive |
| CYLD | AC006480.2 | 0.445757773 | 1.93E-55 | positive |
| MAP3K7 | AC006480.2 | 0.408905795 | 4.21E-46 | positive |
| DIABLO | AC006480.2 | 0.725538592 | 1.61E-182 | positive |
| CFLAR | AC006480.2 | 0.4943315 | 1.22E-69 | positive |
| BRAF | AC006480.2 | 0.707696346 | 8.01E-170 | positive |
| ATRX | AC006480.2 | 0.59744691 | 1.22E-108 | positive |
| DIABLO | AC002456.1 | 0.437989908 | 2.25E-53 | positive |
| BRAF | AC002456.1 | 0.41059225 | 1.67E-46 | positive |
| OTULIN | AL132656.3 | 0.402151675 | 1.64E-44 | positive |
| CYLD | AL132656.3 | 0.409516098 | 3.01E-46 | positive |
| DIABLO | AL132656.3 | 0.742233809 | 2.39E-195 | positive |
| CFLAR | AL132656.3 | 0.437948591 | 2.31E-53 | positive |
| BRAF | AL132656.3 | 0.641765225 | 3.28E-130 | positive |
| ATRX | AL132656.3 | 0.457174542 | 1.42E-58 | positive |
| CYLD | AC011601.1 | 0.451797505 | 4.40E-57 | positive |
| DIABLO | AC011601.1 | 0.460324517 | 1.84E-59 | positive |
| BRAF | AC011601.1 | 0.440492675 | 4.93E-54 | positive |
| ATRX | AC011601.1 | 0.421477772 | 3.70E-49 | positive |
| OTULIN | ARAP1-AS2 | 0.487810875 | 1.34E-67 | positive |
| CYLD | ARAP1-AS2 | 0.492502222 | 4.61E-69 | positive |
| DIABLO | ARAP1-AS2 | 0.769346475 | 1.89E-218 | positive |
| CFLAR | ARAP1-AS2 | 0.468470186 | 8.53E-62 | positive |
| BRAF | ARAP1-AS2 | 0.712878458 | 2.07E-173 | positive |
| ATRX | ARAP1-AS2 | 0.462210542 | 5.38E-60 | positive |
| DIABLO | AC078789.1 | 0.615612701 | 4.57E-117 | positive |
| BRAF | AC078789.1 | 0.525244958 | 6.05E-80 | positive |
| OTULIN | TMEM75 | 0.447565238 | 6.28E-56 | positive |
| DIABLO | TMEM75 | 0.709418532 | 5.25E-171 | positive |
| BRAF | TMEM75 | 0.602644314 | 5.36E-111 | positive |
| OTULIN | AP005436.2 | 0.406594631 | 1.49E-45 | positive |
| DIABLO | AP005436.2 | 0.401097173 | 2.87E-44 | positive |
| OTULIN | C1orf147 | 0.467832901 | 1.31E-61 | positive |
| CYLD | C1orf147 | 0.412157072 | 7.03E-47 | positive |
| DIABLO | C1orf147 | 0.660661806 | 1.55E-140 | positive |
| CFLAR | C1orf147 | 0.441722352 | 2.32E-54 | positive |
| BRAF | C1orf147 | 0.555390223 | 4.49E-91 | positive |
| RNF31 | C1orf147 | 0.410610219 | 1.65E-46 | positive |
| DIABLO | AC011933.4 | 0.516966589 | 4.43E-77 | positive |
| CFLAR | AC011933.4 | 0.460620221 | 1.52E-59 | positive |
| BRAF | AC011933.4 | 0.463182146 | 2.84E-60 | positive |
| ATRX | AC011933.4 | 0.54404915 | 9.39E-87 | positive |
| DIABLO | AC002550.2 | 0.53575539 | 1.07E-83 | positive |
| BRAF | AC002550.2 | 0.502647413 | 2.62E-72 | positive |
| ATRX | AC002550.2 | 0.41155565 | 9.80E-47 | positive |
| KLF9 | HAND2-AS1 | 0.617981561 | 3.31E-118 | positive |
| DIABLO | AC002044.1 | 0.441682546 | 2.38E-54 | positive |
| BRAF | AC002044.1 | 0.403117673 | 9.74E-45 | positive |
| ATRX | AC002044.1 | 0.456682639 | 1.95E-58 | positive |
| OTULIN | FTX | 0.415131481 | 1.34E-47 | positive |
| DIABLO | FTX | 0.68084182 | 2.00E-152 | positive |
| BRAF | FTX | 0.641344384 | 5.47E-130 | positive |
| ATRX | FTX | 0.443886795 | 6.15E-55 | positive |
| DIABLO | AC091868.2 | 0.630045836 | 3.65E-124 | positive |
| BRAF | AC091868.2 | 0.494707318 | 9.28E-70 | positive |
| ATRX | AC091868.2 | 0.417538919 | 3.47E-48 | positive |
| DIABLO | EML6-AS1 | 0.521228491 | 1.52E-78 | positive |
| BRAF | EML6-AS1 | 0.439209855 | 1.07E-53 | positive |
| DIABLO | AC011120.1 | 0.672972519 | 1.12E-147 | positive |
| BRAF | AC011120.1 | 0.593289098 | 8.67E-107 | positive |
| BCL2 | AL592429.2 | 0.409728521 | 2.68E-46 | positive |
| CD40 | AL592429.2 | 0.425645827 | 3.36E-50 | positive |
| BCL2L11 | AL592429.2 | 0.436676173 | 4.97E-53 | positive |
| OTULIN | FLJ13224 | 0.401229988 | 2.68E-44 | positive |
| DIABLO | FLJ13224 | 0.712695512 | 2.78E-173 | positive |
| BRAF | FLJ13224 | 0.592937864 | 1.24E-106 | positive |
| ATRX | FLJ13224 | 0.434263983 | 2.11E-52 | positive |
| DIABLO | AC011370.1 | 0.485286697 | 8.07E-67 | positive |
| BRAF | AC011370.1 | 0.465237684 | 7.33E-61 | positive |
| BNIP3 | MIR205HG | 0.443206094 | 9.35E-55 | positive |
| AXL | LINC00842 | 0.559509301 | 1.10E-92 | positive |
| DIABLO | AC024257.4 | 0.599060841 | 2.28E-109 | positive |
| BRAF | AC024257.4 | 0.512948292 | 1.02E-75 | positive |
| DIABLO | AL355073.2 | 0.429345419 | 3.88E-51 | positive |
| BRAF | AL355073.2 | 0.517063527 | 4.10E-77 | positive |
| ATRX | AL355073.2 | 0.463473858 | 2.35E-60 | positive |
| DIABLO | AC004263.1 | 0.633824019 | 4.38E-126 | positive |
| BRAF | AC004263.1 | 0.422944699 | 1.60E-49 | positive |
| RNF31 | AC004263.1 | 0.403933793 | 6.28E-45 | positive |
| BCL2 | LINC00173 | 0.419431918 | 1.19E-48 | positive |
| BNIP3 | TM4SF19-AS1 | 0.466302933 | 3.62E-61 | positive |
| MAPK8 | AC093157.2 | 0.444000418 | 5.74E-55 | positive |
| IPMK | AC093157.2 | 0.413849391 | 2.74E-47 | positive |
| DIABLO | AC093157.2 | 0.54541296 | 2.90E-87 | positive |
| BRAF | AC093157.2 | 0.559435268 | 1.18E-92 | positive |
| ATRX | AC093157.2 | 0.425992231 | 2.75E-50 | positive |
| OTULIN | AC117834.1 | 0.438304179 | 1.86E-53 | positive |
| DIABLO | AC117834.1 | 0.742256688 | 2.29E-195 | positive |
| BRAF | AC117834.1 | 0.667741729 | 1.34E-144 | positive |
| MAP3K7 | AL513550.1 | 0.51753243 | 2.84E-77 | positive |
| DIABLO | AL513550.1 | 0.461740279 | 7.32E-60 | positive |
| BRAF | AL513550.1 | 0.505307423 | 3.54E-73 | positive |
| ATRX | AL513550.1 | 0.405590171 | 2.57E-45 | positive |
| OTULIN | AC074029.3 | 0.404571583 | 4.45E-45 | positive |
| CYLD | AC074029.3 | 0.410635678 | 1.63E-46 | positive |
| DIABLO | AC074029.3 | 0.630772673 | 1.57E-124 | positive |
| BRAF | AC074029.3 | 0.568570095 | 2.62E-96 | positive |
| ATRX | AC074029.3 | 0.421860458 | 2.97E-49 | positive |
| TSC1 | AL109811.1 | 0.413330616 | 3.66E-47 | positive |
| DIABLO | AL109811.1 | 0.492746835 | 3.86E-69 | positive |
| BRAF | AL109811.1 | 0.468062954 | 1.12E-61 | positive |
| RNF31 | AL109811.1 | 0.48870478 | 7.10E-68 | positive |
| OTULIN | AC004217.1 | 0.423521192 | 1.15E-49 | positive |
| CYLD | AC004217.1 | 0.418724268 | 1.77E-48 | positive |
| DIABLO | AC004217.1 | 0.73695358 | 3.48E-191 | positive |
| BRAF | AC004217.1 | 0.642697564 | 1.06E-130 | positive |
| ATRX | AC004217.1 | 0.410729259 | 1.55E-46 | positive |
| DIABLO | AC107308.1 | 0.44127575 | 3.05E-54 | positive |
| BRAF | AC107308.1 | 0.432262421 | 6.94E-52 | positive |
| DIABLO | AC009686.1 | 0.64637057 | 1.17E-132 | positive |
| BRAF | AC009686.1 | 0.536166121 | 7.58E-84 | positive |
| CD40 | AC003092.1 | 0.598239458 | 5.35E-109 | positive |
| HDAC9 | MYOSLID | 0.523252543 | 3.01E-79 | positive |
| TSC1 | AC024075.1 | 0.407016931 | 1.18E-45 | positive |
| CYLD | AC024075.1 | 0.45440333 | 8.39E-58 | positive |
| DIABLO | AC024075.1 | 0.471016389 | 1.54E-62 | positive |
| CFLAR | AC024075.1 | 0.558879129 | 1.95E-92 | positive |
| BRAF | AC024075.1 | 0.510009393 | 9.84E-75 | positive |
| BACH2 | AC024075.1 | 0.406013795 | 2.04E-45 | positive |
| ATRX | AC024075.1 | 0.620362911 | 2.32E-119 | positive |
| KLF9 | AC024075.1 | 0.504208555 | 8.10E-73 | positive |
| CYLD | AC007496.2 | 0.447989396 | 4.82E-56 | positive |
| DIABLO | AC007496.2 | 0.702236319 | 3.98E-166 | positive |
| CFLAR | AC007496.2 | 0.441863355 | 2.13E-54 | positive |
| BRAF | AC007496.2 | 0.627967687 | 4.05E-123 | positive |
| ATRX | AC007496.2 | 0.549124689 | 1.15E-88 | positive |
| DIABLO | AL157762.1 | 0.481715949 | 9.94E-66 | positive |
| BRAF | AL157762.1 | 0.414635255 | 1.77E-47 | positive |
| OTULIN | AC022395.1 | 0.471671126 | 9.91E-63 | positive |
| CYLD | AC022395.1 | 0.407001974 | 1.19E-45 | positive |
| DIABLO | AC022395.1 | 0.729320593 | 2.42E-185 | positive |
| BRAF | AC022395.1 | 0.677881263 | 1.27E-150 | positive |
| TSC1 | LINC00205 | 0.497802566 | 9.58E-71 | positive |
| USP22 | LINC00205 | 0.428280489 | 7.24E-51 | positive |
| MAP3K7 | LINC00205 | 0.423502118 | 1.16E-49 | positive |
| TSC1 | AL450998.2 | 0.54936157 | 9.33E-89 | positive |
| MAPK8 | AL450998.2 | 0.405055758 | 3.43E-45 | positive |
| OTULIN | AL450998.2 | 0.476067168 | 4.97E-64 | positive |
| CYLD | AL450998.2 | 0.565651987 | 3.96E-95 | positive |
| DIABLO | AL450998.2 | 0.674541813 | 1.30E-148 | positive |
| CFLAR | AL450998.2 | 0.602939411 | 3.93E-111 | positive |
| BRAF | AL450998.2 | 0.683628126 | 3.83E-154 | positive |
| MPG | AL450998.2 | -0.434544645 | 1.78E-52 | negative |
| ATRX | AL450998.2 | 0.64814569 | 1.30E-133 | positive |
| RNF31 | AL450998.2 | 0.561894724 | 1.26E-93 | positive |
| DIABLO | AC025741.1 | 0.595661955 | 7.65E-108 | positive |
| BRAF | AC025741.1 | 0.509632609 | 1.31E-74 | positive |
| ATRX | AC025741.1 | 0.430363727 | 2.13E-51 | positive |
| TSC1 | SDCBP2-AS1 | 0.422492263 | 2.07E-49 | positive |
| MAPK8 | SDCBP2-AS1 | 0.474842406 | 1.15E-63 | positive |
| IPMK | SDCBP2-AS1 | 0.425321912 | 4.05E-50 | positive |
| OTULIN | SDCBP2-AS1 | 0.508079743 | 4.31E-74 | positive |
| CYLD | SDCBP2-AS1 | 0.48395572 | 2.06E-66 | positive |
| MAP3K7 | SDCBP2-AS1 | 0.450969834 | 7.42E-57 | positive |
| DIABLO | SDCBP2-AS1 | 0.765604735 | 4.47E-215 | positive |
| CFLAR | SDCBP2-AS1 | 0.477302072 | 2.13E-64 | positive |
| BRAF | SDCBP2-AS1 | 0.745995791 | 2.24E-198 | positive |
| ATRX | SDCBP2-AS1 | 0.559046031 | 1.68E-92 | positive |
| OTULIN | AC007535.1 | 0.404742222 | 4.06E-45 | positive |
| DIABLO | AC007535.1 | 0.643197674 | 5.75E-131 | positive |
| BRAF | AC007535.1 | 0.574650252 | 8.38E-99 | positive |
| OTULIN | AC026401.1 | 0.425060149 | 4.71E-50 | positive |
| CYLD | AC026401.1 | 0.459009343 | 4.33E-59 | positive |
| DIABLO | AC026401.1 | 0.780715061 | 4.19E-229 | positive |
| CFLAR | AC026401.1 | 0.416435348 | 6.45E-48 | positive |
| BRAF | AC026401.1 | 0.684621578 | 9.25E-155 | positive |
| ATRX | AC026401.1 | 0.435324408 | 1.12E-52 | positive |
| DIABLO | AL451069.2 | 0.498781383 | 4.65E-71 | positive |
| ATRX | PLS3-AS1 | 0.506731158 | 1.20E-73 | positive |
| MYCN | MYCNOS | 0.945769764 | 0 | positive |
| DIABLO | AL353697.1 | 0.749719428 | 1.99E-201 | positive |
| BRAF | AL353697.1 | 0.600737237 | 3.97E-110 | positive |
| DIABLO | AC091117.2 | 0.648218812 | 1.18E-133 | positive |
| BRAF | AC091117.2 | 0.493561523 | 2.14E-69 | positive |
| DIABLO | AC025871.1 | 0.527507493 | 9.66E-81 | positive |
| BRAF | AC025871.1 | 0.478775935 | 7.68E-65 | positive |
| ATRX | AC025871.1 | 0.443387673 | 8.36E-55 | positive |
| DIABLO | AC091729.1 | 0.46410783 | 1.55E-60 | positive |
| BRAF | AC091729.1 | 0.400549563 | 3.85E-44 | positive |
| OTULIN | AL021328.1 | 0.412825189 | 4.85E-47 | positive |
| CYLD | AL021328.1 | 0.404319039 | 5.10E-45 | positive |
| DIABLO | AL021328.1 | 0.69101186 | 8.65E-159 | positive |
| CFLAR | AL021328.1 | 0.405622692 | 2.52E-45 | positive |
| BRAF | AL021328.1 | 0.621421934 | 7.04E-120 | positive |
| ATRX | AL021328.1 | 0.451574247 | 5.06E-57 | positive |
| OTULIN | SCAT2 | 0.478231508 | 1.12E-64 | positive |
| MAP3K7 | SCAT2 | 0.428978332 | 4.81E-51 | positive |
| DIABLO | SCAT2 | 0.659163892 | 1.09E-139 | positive |
| BRAF | SCAT2 | 0.56949695 | 1.10E-96 | positive |
| DIABLO | AC005753.3 | 0.560836093 | 3.30E-93 | positive |
| CFLAR | AC005753.3 | 0.413260565 | 3.81E-47 | positive |
| BRAF | AC005753.3 | 0.441338354 | 2.94E-54 | positive |
| AXL | AC009549.1 | 0.551700059 | 1.19E-89 | positive |
| KLF9 | AC009549.1 | 0.588928394 | 7.13E-105 | positive |
| OTULIN | WARS2-IT1 | 0.424541649 | 6.36E-50 | positive |
| CYLD | WARS2-IT1 | 0.461305755 | 9.73E-60 | positive |
| DIABLO | WARS2-IT1 | 0.777139102 | 1.10E-225 | positive |
| BRAF | WARS2-IT1 | 0.678580352 | 4.80E-151 | positive |
| ATRX | WARS2-IT1 | 0.44165094 | 2.43E-54 | positive |
| MAPK8 | AC139019.1 | 0.407624747 | 8.49E-46 | positive |
| OTULIN | AC139019.1 | 0.499254669 | 3.28E-71 | positive |
| CYLD | AC139019.1 | 0.437714495 | 2.66E-53 | positive |
| MAP3K7 | AC139019.1 | 0.402389228 | 1.44E-44 | positive |
| DIABLO | AC139019.1 | 0.671924368 | 4.69E-147 | positive |
| BRAF | AC139019.1 | 0.668438533 | 5.25E-145 | positive |
| ATRX | AC139019.1 | 0.502813904 | 2.31E-72 | positive |
| MAPK8 | AL359762.3 | 0.455195149 | 5.06E-58 | positive |
| IPMK | AL359762.3 | 0.452517584 | 2.79E-57 | positive |
| OTULIN | AL359762.3 | 0.48613131 | 4.44E-67 | positive |
| CYLD | AL359762.3 | 0.508803251 | 2.48E-74 | positive |
| MAP3K7 | AL359762.3 | 0.418108752 | 2.51E-48 | positive |
| DIABLO | AL359762.3 | 0.800045282 | 8.98E-249 | positive |
| CFLAR | AL359762.3 | 0.450837178 | 8.06E-57 | positive |
| BRAF | AL359762.3 | 0.729919762 | 8.57E-186 | positive |
| ATRX | AL359762.3 | 0.516821755 | 4.96E-77 | positive |
| DIABLO | AC120042.1 | 0.415426374 | 1.14E-47 | positive |
| OTULIN | AC007785.3 | 0.400989258 | 3.04E-44 | positive |
| DIABLO | AC007785.3 | 0.59751511 | 1.13E-108 | positive |
| BRAF | AC007785.3 | 0.520204103 | 3.43E-78 | positive |
| ATRX | AC007785.3 | 0.464620445 | 1.10E-60 | positive |
| DIABLO | AC021744.1 | 0.719077359 | 8.29E-178 | positive |
| BRAF | AC021744.1 | 0.591963929 | 3.33E-106 | positive |
| ATRX | AC021744.1 | 0.406860651 | 1.29E-45 | positive |
| FASLG | HLA-DQB1-AS1 | 0.404987481 | 3.56E-45 | positive |
| CYLD | HLA-DQB1-AS1 | 0.44665051 | 1.11E-55 | positive |
| CFLAR | HLA-DQB1-AS1 | 0.40853599 | 5.16E-46 | positive |
| TSC1 | AC134407.1 | 0.404660255 | 4.24E-45 | positive |
| CYLD | AC134407.1 | 0.419636767 | 1.06E-48 | positive |
| DIABLO | AC134407.1 | 0.632707276 | 1.63E-125 | positive |
| CFLAR | AC134407.1 | 0.520185795 | 3.48E-78 | positive |
| BRAF | AC134407.1 | 0.598720175 | 3.25E-109 | positive |
| STUB1 | AC134407.1 | -0.419847599 | 9.38E-49 | negative |
| MPG | AC134407.1 | -0.458997341 | 4.37E-59 | negative |
| ATRX | AC134407.1 | 0.700680495 | 4.34E-165 | positive |
| RNF31 | AC134407.1 | 0.475932876 | 5.45E-64 | positive |
| DIABLO | TTLL10-AS1 | 0.528246237 | 5.29E-81 | positive |
| MAPK8 | AC090739.1 | 0.420050538 | 8.35E-49 | positive |
| IPMK | AC090739.1 | 0.442116335 | 1.83E-54 | positive |
| OTULIN | AC090739.1 | 0.477013228 | 2.59E-64 | positive |
| CYLD | AC090739.1 | 0.434979574 | 1.37E-52 | positive |
| DIABLO | AC090739.1 | 0.780799973 | 3.47E-229 | positive |
| BRAF | AC090739.1 | 0.724225708 | 1.49E-181 | positive |
| ATRX | AC090739.1 | 0.447244807 | 7.67E-56 | positive |
| DIABLO | AC016586.1 | 0.560450512 | 4.69E-93 | positive |
| BRAF | AC016586.1 | 0.427954832 | 8.76E-51 | positive |
| DIABLO | AC000120.1 | 0.607482459 | 3.15E-113 | positive |
| BRAF | AC000120.1 | 0.559776674 | 8.64E-93 | positive |
| ATRX | AC000120.1 | 0.431196563 | 1.30E-51 | positive |
| DIABLO | EHD4-AS1 | 0.751494832 | 6.69E-203 | positive |
| CFLAR | EHD4-AS1 | 0.406542186 | 1.53E-45 | positive |
| BRAF | EHD4-AS1 | 0.603143607 | 3.17E-111 | positive |
| KLF9 | AL513217.1 | 0.649685868 | 1.90E-134 | positive |
| DIABLO | AC009803.1 | 0.468545101 | 8.12E-62 | positive |
| BRAF | AC009803.1 | 0.408242759 | 6.06E-46 | positive |
| TSC1 | AC018695.3 | 0.415459633 | 1.12E-47 | positive |
| DIABLO | AC018695.3 | 0.589211196 | 5.36E-105 | positive |
| BRAF | AC018695.3 | 0.531692737 | 3.13E-82 | positive |
| ATRX | AC018695.3 | 0.42489823 | 5.18E-50 | positive |
| DIABLO | AL035411.3 | 0.449018637 | 2.53E-56 | positive |
| BRAF | AL035411.3 | 0.436814646 | 4.57E-53 | positive |
| DIABLO | Z83847.1 | 0.600619382 | 4.49E-110 | positive |
| BRAF | Z83847.1 | 0.484531595 | 1.38E-66 | positive |
| TSC1 | AC232271.1 | 0.434317999 | 2.04E-52 | positive |
| DIABLO | AC037487.1 | 0.534783029 | 2.41E-83 | positive |
| BRAF | AC037487.1 | 0.465151219 | 7.76E-61 | positive |
| TRIM11 | AP001453.2 | 0.432607688 | 5.65E-52 | positive |
| SIRT3 | AP001453.2 | 0.42676674 | 1.75E-50 | positive |
| DIABLO | AC009093.10 | 0.502137782 | 3.83E-72 | positive |
| CFLAR | AC009093.10 | 0.415892002 | 8.76E-48 | positive |
| BRAF | AC009093.10 | 0.445618549 | 2.11E-55 | positive |
| ATRX | AC009093.10 | 0.401474118 | 2.35E-44 | positive |
| DIABLO | AC027801.5 | 0.522862976 | 4.11E-79 | positive |
| CFLAR | AC027801.5 | 0.411851047 | 8.32E-47 | positive |
| BRAF | AC027801.5 | 0.50414972 | 8.47E-73 | positive |
| ATRX | AC027801.5 | 0.474781225 | 1.20E-63 | positive |
| STUB1 | PPP1R14B-AS1 | 0.540599102 | 1.80E-85 | positive |
| MPG | PPP1R14B-AS1 | 0.539548471 | 4.38E-85 | positive |
| CYLD | AC010623.1 | 0.474768245 | 1.21E-63 | positive |
| DIABLO | AC010623.1 | 0.615969966 | 3.08E-117 | positive |
| BRAF | AC010623.1 | 0.568258763 | 3.51E-96 | positive |
| ATRX | AC010623.1 | 0.484484767 | 1.42E-66 | positive |
| TARDBP | AC022007.1 | 0.451713066 | 4.64E-57 | positive |
| OTULIN | PRDX6-AS1 | 0.476345622 | 4.10E-64 | positive |
| CYLD | PRDX6-AS1 | 0.548101221 | 2.81E-88 | positive |
| MAP3K7 | PRDX6-AS1 | 0.426634369 | 1.89E-50 | positive |
| DIABLO | PRDX6-AS1 | 0.635791912 | 4.27E-127 | positive |
| CFLAR | PRDX6-AS1 | 0.495066237 | 7.14E-70 | positive |
| BRAF | PRDX6-AS1 | 0.636699094 | 1.45E-127 | positive |
| BCL2 | PRDX6-AS1 | 0.451879377 | 4.17E-57 | positive |
| ATRX | PRDX6-AS1 | 0.532087757 | 2.26E-82 | positive |
| BCL2L11 | PRDX6-AS1 | 0.528708336 | 3.63E-81 | positive |
| CYLD | MAGI2-AS3 | 0.57586925 | 2.61E-99 | positive |
| DIABLO | MAGI2-AS3 | 0.479930066 | 3.45E-65 | positive |
| CFLAR | MAGI2-AS3 | 0.527439186 | 1.02E-80 | positive |
| BRAF | MAGI2-AS3 | 0.588986764 | 6.72E-105 | positive |
| AXL | MAGI2-AS3 | 0.43152323 | 1.07E-51 | positive |
| ATRX | MAGI2-AS3 | 0.505594637 | 2.85E-73 | positive |
| KLF9 | MAGI2-AS3 | 0.671439595 | 9.08E-147 | positive |
| DIABLO | PCCA-AS1 | 0.667577326 | 1.67E-144 | positive |
| BRAF | PCCA-AS1 | 0.523567198 | 2.34E-79 | positive |
| DIABLO | Z99572.1 | 0.409322785 | 3.35E-46 | positive |
| BRAF | Z99572.1 | 0.462323111 | 5.00E-60 | positive |
| ATRX | Z99572.1 | 0.439378975 | 9.70E-54 | positive |
| CD40 | LINC02757 | 0.50144788 | 6.42E-72 | positive |
| OTULIN | AC007546.1 | 0.424906788 | 5.15E-50 | positive |
| CYLD | AC007546.1 | 0.491467298 | 9.75E-69 | positive |
| DIABLO | AC007546.1 | 0.796732387 | 2.98E-245 | positive |
| CFLAR | AC007546.1 | 0.454885185 | 6.17E-58 | positive |
| BRAF | AC007546.1 | 0.657917543 | 5.46E-139 | positive |
| ATRX | AC007546.1 | 0.486422101 | 3.61E-67 | positive |
| DIABLO | MEIS1-AS2 | 0.593914881 | 4.58E-107 | positive |
| BRAF | MEIS1-AS2 | 0.549480642 | 8.40E-89 | positive |
| OTULIN | AL442128.2 | 0.412964086 | 4.49E-47 | positive |
| MAP3K7 | AL442128.2 | 0.418510225 | 2.00E-48 | positive |
| DIABLO | AL442128.2 | 0.538292819 | 1.27E-84 | positive |
| BRAF | AL442128.2 | 0.49955654 | 2.62E-71 | positive |
| OTULIN | AC022973.4 | 0.464676162 | 1.06E-60 | positive |
| DIABLO | AC022973.4 | 0.710915707 | 4.83E-172 | positive |
| BRAF | AC022973.4 | 0.632842244 | 1.39E-125 | positive |
| BCL2 | AC113361.1 | 0.430962759 | 1.50E-51 | positive |
| BCL2L11 | AC113361.1 | 0.416348923 | 6.78E-48 | positive |
| TSC1 | AC090589.3 | 0.541189881 | 1.09E-85 | positive |
| DIABLO | AC090589.3 | 0.533295928 | 8.30E-83 | positive |
| CFLAR | AC090589.3 | 0.492662362 | 4.11E-69 | positive |
| BRAF | AC090589.3 | 0.495508792 | 5.17E-70 | positive |
| ATRX | AC090589.3 | 0.477096832 | 2.45E-64 | positive |
| RNF31 | AC090589.3 | 0.486562559 | 3.27E-67 | positive |
| CYLD | MIR100HG | 0.418664075 | 1.84E-48 | positive |
| AXL | MIR100HG | 0.486280644 | 3.99E-67 | positive |
| KLF9 | MIR100HG | 0.706629315 | 4.29E-169 | positive |
| TSC1 | MUC20-OT1 | 0.454280346 | 9.07E-58 | positive |
| OTULIN | MUC20-OT1 | 0.423388734 | 1.24E-49 | positive |
| DIABLO | MUC20-OT1 | 0.544183764 | 8.36E-87 | positive |
| CFLAR | MUC20-OT1 | 0.402552332 | 1.32E-44 | positive |
| BRAF | MUC20-OT1 | 0.513826063 | 5.15E-76 | positive |
| ATRX | MUC20-OT1 | 0.482145557 | 7.36E-66 | positive |
| CYLD | LINC01358 | 0.447793192 | 5.45E-56 | positive |
| DIABLO | LINC01358 | 0.458218338 | 7.23E-59 | positive |
| DIABLO | AP002518.2 | 0.540363013 | 2.20E-85 | positive |
| BRAF | AP002518.2 | 0.504305732 | 7.53E-73 | positive |
| KLF9 | AP002518.2 | 0.470426503 | 2.30E-62 | positive |
| OTULIN | AL355336.1 | 0.408399106 | 5.56E-46 | positive |
| DIABLO | AL355336.1 | 0.749124922 | 6.16E-201 | positive |
| BRAF | AL355336.1 | 0.640675157 | 1.23E-129 | positive |
| RNF31 | AC008735.1 | 0.4674224 | 1.72E-61 | positive |
| CYLD | AC090826.3 | 0.411956727 | 7.85E-47 | positive |
| DIABLO | AC090826.3 | 0.563618546 | 2.59E-94 | positive |
| BRAF | AC090826.3 | 0.53234766 | 1.82E-82 | positive |
| TSC1 | RHOA-IT1 | 0.400126507 | 4.82E-44 | positive |
| CYLD | RHOA-IT1 | 0.418077368 | 2.56E-48 | positive |
| DIABLO | RHOA-IT1 | 0.656871546 | 2.10E-138 | positive |
| CFLAR | RHOA-IT1 | 0.496691627 | 2.17E-70 | positive |
| BRAF | RHOA-IT1 | 0.574722902 | 7.82E-99 | positive |
| ATRX | RHOA-IT1 | 0.543875242 | 1.09E-86 | positive |
| RNF31 | RHOA-IT1 | 0.45714621 | 1.44E-58 | positive |
| TSC1 | AC008870.2 | 0.509314057 | 1.68E-74 | positive |
| MAPK8 | AC008870.2 | 0.416127285 | 7.67E-48 | positive |
| OTULIN | AC008870.2 | 0.451293007 | 6.05E-57 | positive |
| CYLD | AC008870.2 | 0.420024868 | 8.48E-49 | positive |
| MAP3K7 | AC008870.2 | 0.444120886 | 5.32E-55 | positive |
| DIABLO | AC008870.2 | 0.614478908 | 1.59E-116 | positive |
| CFLAR | AC008870.2 | 0.417141427 | 4.34E-48 | positive |
| BRAF | AC008870.2 | 0.570000172 | 6.86E-97 | positive |
| ATRX | AC008870.2 | 0.43147213 | 1.11E-51 | positive |
| RNF31 | AC008870.2 | 0.432234305 | 7.05E-52 | positive |
| AXL | LINC00592 | 0.423649581 | 1.06E-49 | positive |
| DIABLO | AL162741.1 | 0.460602187 | 1.54E-59 | positive |
| DIABLO | AC009054.2 | 0.616959163 | 1.03E-117 | positive |
| BRAF | AC009054.2 | 0.521774947 | 9.81E-79 | positive |
| TSC1 | U91328.1 | 0.410831781 | 1.46E-46 | positive |
| BRAF | U91328.1 | 0.408038577 | 6.77E-46 | positive |
| DIABLO | AC006441.1 | 0.540254733 | 2.41E-85 | positive |
| BRAF | AC006441.1 | 0.473377977 | 3.12E-63 | positive |
| MPG | AC006441.1 | -0.407000331 | 1.19E-45 | negative |
| ATRX | AC006441.1 | 0.509947737 | 1.03E-74 | positive |
| DIABLO | AL390067.1 | 0.420230972 | 7.54E-49 | positive |
| KLF9 | AP000894.2 | 0.508351127 | 3.50E-74 | positive |
| TSC1 | AP001469.2 | 0.511326074 | 3.57E-75 | positive |
| DIABLO | AP001469.2 | 0.461817537 | 6.96E-60 | positive |
| BRAF | AP001469.2 | 0.495765889 | 4.28E-70 | positive |
| ATRX | AP001469.2 | 0.434538223 | 1.79E-52 | positive |
| RNF31 | AP001469.2 | 0.40626269 | 1.78E-45 | positive |
| DIABLO | CLYBL-AS1 | 0.713899137 | 3.98E-174 | positive |
| BRAF | CLYBL-AS1 | 0.544043044 | 9.44E-87 | positive |
| DIABLO | AC035139.1 | 0.413540868 | 3.26E-47 | positive |
| BCL2 | AC035139.1 | 0.479074953 | 6.24E-65 | positive |
| BCL2L11 | AC035139.1 | 0.434642314 | 1.68E-52 | positive |
| DIABLO | AC010148.1 | 0.43773237 | 2.63E-53 | positive |
| BRAF | AC010148.1 | 0.470052468 | 2.95E-62 | positive |
| ATRX | AC010148.1 | 0.457786174 | 9.56E-59 | positive |
| TSC1 | RNF213-AS1 | 0.463523796 | 2.27E-60 | positive |
| CYLD | RNF213-AS1 | 0.464651108 | 1.08E-60 | positive |
| DIABLO | RNF213-AS1 | 0.692681426 | 7.36E-160 | positive |
| CFLAR | RNF213-AS1 | 0.554898529 | 6.97E-91 | positive |
| BRAF | RNF213-AS1 | 0.614566921 | 1.44E-116 | positive |
| ATRX | RNF213-AS1 | 0.577813225 | 4.02E-100 | positive |
| RNF31 | RNF213-AS1 | 0.488144748 | 1.06E-67 | positive |
| DIABLO | AC064836.3 | 0.551765132 | 1.13E-89 | positive |
| BRAF | AC064836.3 | 0.43339837 | 3.53E-52 | positive |
| DIABLO | AL031985.4 | 0.616495425 | 1.72E-117 | positive |
| BRAF | AL031985.4 | 0.497152745 | 1.55E-70 | positive |
| OTULIN | AL451070.1 | 0.441181757 | 3.23E-54 | positive |
| CYLD | AL451070.1 | 0.416812693 | 5.22E-48 | positive |
| DIABLO | AL451070.1 | 0.758157857 | 1.52E-208 | positive |
| BRAF | AL451070.1 | 0.676211479 | 1.30E-149 | positive |
| ATRX | AL451070.1 | 0.405668757 | 2.46E-45 | positive |
| DIABLO | SIAH2-AS1 | 0.447760468 | 5.56E-56 | positive |
| BRAF | SIAH2-AS1 | 0.449848327 | 1.50E-56 | positive |
| ATRX | SIAH2-AS1 | 0.428635681 | 5.88E-51 | positive |
| GATA3 | AL157931.1 | 0.528451844 | 4.47E-81 | positive |
| OTULIN | AC015923.1 | 0.436811725 | 4.58E-53 | positive |
| CYLD | AC015923.1 | 0.42296363 | 1.58E-49 | positive |
| DIABLO | AC015923.1 | 0.654662919 | 3.55E-137 | positive |
| BRAF | AC015923.1 | 0.627353552 | 8.21E-123 | positive |
| DIABLO | AC004837.2 | 0.608900907 | 6.86E-114 | positive |
| BRAF | AC004837.2 | 0.542909052 | 2.50E-86 | positive |
| TSC1 | AC066613.1 | 0.418345394 | 2.20E-48 | positive |
| MAPK8 | AC066613.1 | 0.485279131 | 8.11E-67 | positive |
| IPMK | AC066613.1 | 0.439333982 | 9.97E-54 | positive |
| OTULIN | AC066613.1 | 0.469255185 | 5.04E-62 | positive |
| CYLD | AC066613.1 | 0.487973827 | 1.20E-67 | positive |
| MAP3K7 | AC066613.1 | 0.435434948 | 1.05E-52 | positive |
| DIABLO | AC066613.1 | 0.755107093 | 6.13E-206 | positive |
| CFLAR | AC066613.1 | 0.502512367 | 2.90E-72 | positive |
| BRAF | AC066613.1 | 0.708306972 | 3.06E-170 | positive |
| ATRX | AC066613.1 | 0.491848731 | 7.40E-69 | positive |
| DIABLO | AC096920.1 | 0.406490453 | 1.57E-45 | positive |
| OTULIN | AL158196.1 | 0.404921242 | 3.68E-45 | positive |
| DIABLO | AL158196.1 | 0.574010668 | 1.54E-98 | positive |
| BRAF | AL158196.1 | 0.491043459 | 1.32E-68 | positive |
| KLF9 | PGM5-AS1 | 0.524639745 | 9.86E-80 | positive |
| TSC1 | ZNF460-AS1 | 0.444610155 | 3.94E-55 | positive |
| DIABLO | ZNF460-AS1 | 0.58224501 | 5.39E-102 | positive |
| BRAF | ZNF460-AS1 | 0.537087397 | 3.50E-84 | positive |
[truncated: 325,370 more chars]
