# Supplementary material for: Construction and validation of a necroptosis-related lncRNA signature for predicting the prognosis of gastrointestinal cancer patients
Source: Front Immunol. 2025 Aug 14;16:1591252. doi: 10.3389/fimmu.2025.1591252 (PMC12391139; doi:10.3389/fimmu.2025.1591252)
Supplement: Supplementary file 3 [file Table3.doc]

**Supplementary Figures**

**Supplementary Fig. S1**

**
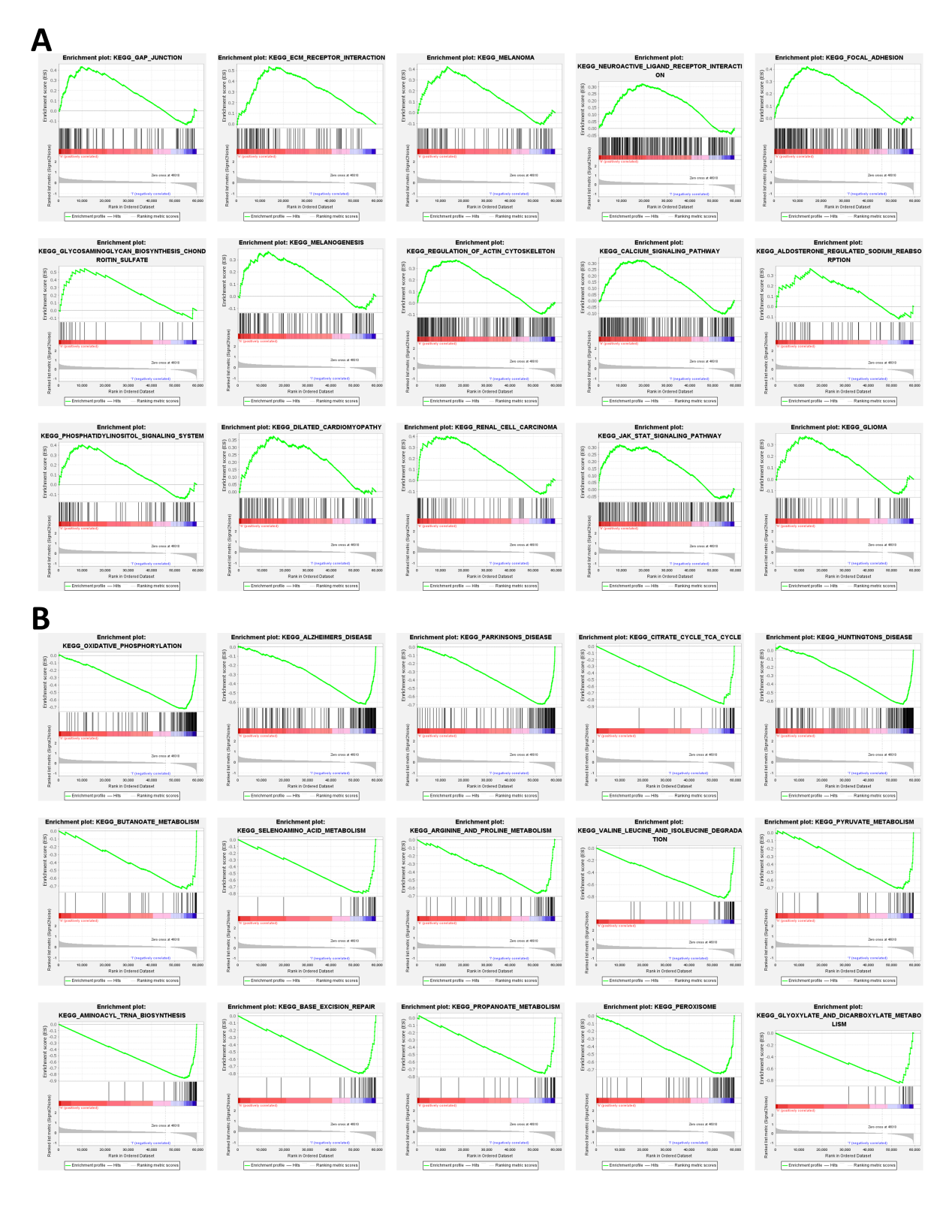
**

**Supplementary Fig. S1: GSEA of Necro-lnc score groups.** (A) GSEA of the top 15 pathways that were significantly enriched in the high-score group. (B) GSEA of the top 15 pathways that were significantly enriched in the low-score group.

**Supplementary Fig. S2**

**
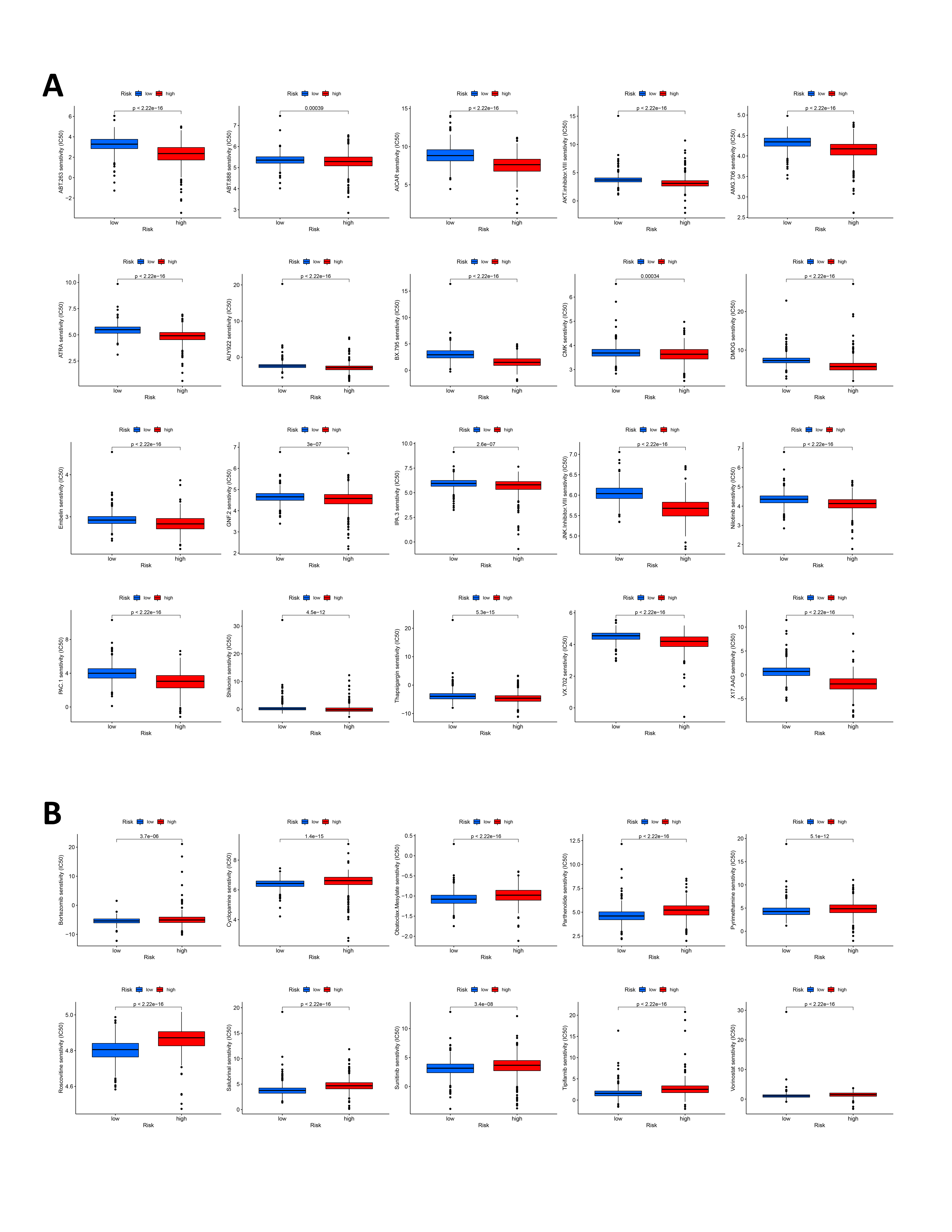
**

**Supplementary Fig. S2: Correlation between the Necro-lnc score and drug sensitivity.** (A) Analysis of the targeted drugs to which the high-score groups were more sensitive. (B) Analysis of the targeted drugs to which the low-score groups were more sensitive.

**Supplementary Fig. S3**

**
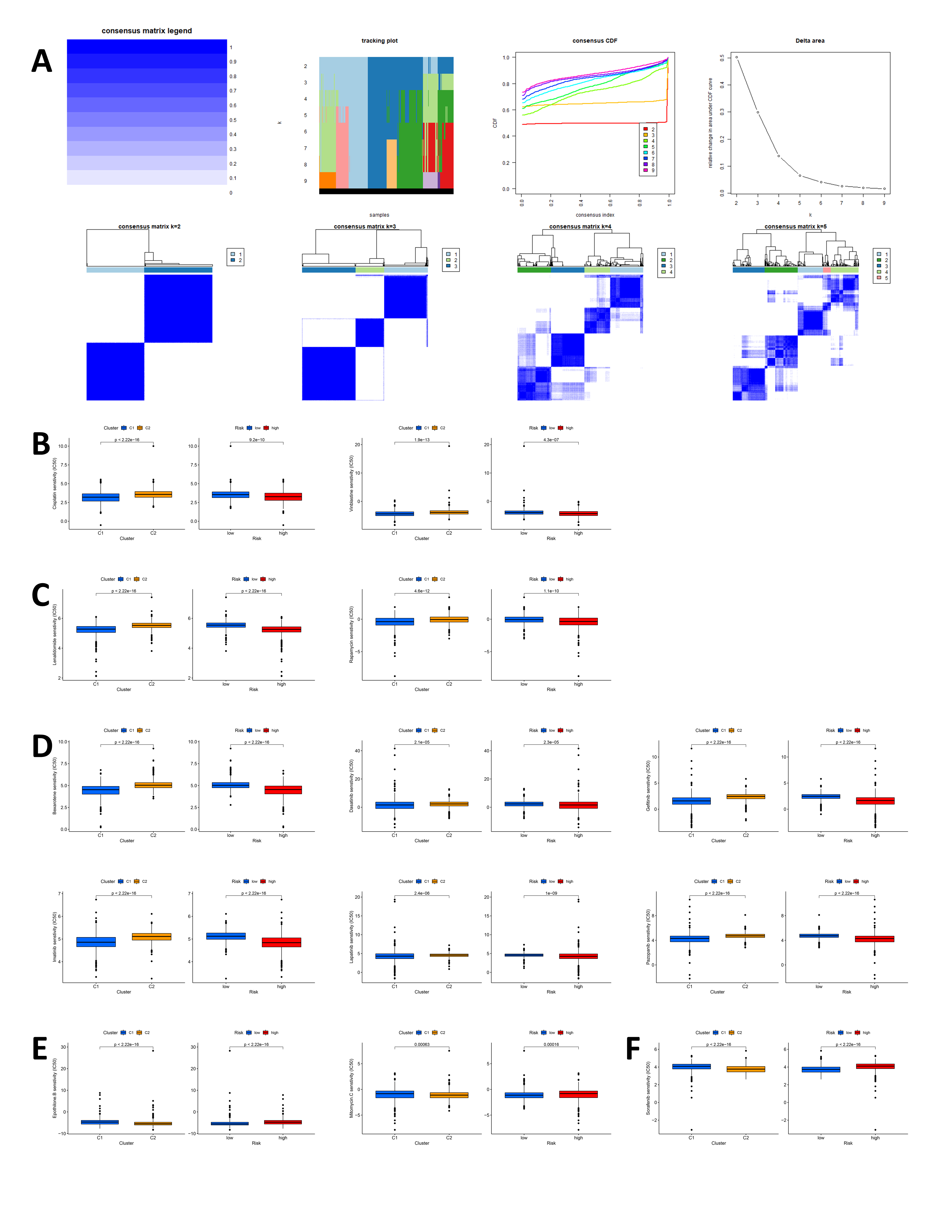
**

**Supplementary Fig. S3: Clustering analysis of the Necro-lnc score.** (A) Patients were divided into two clusters by ConsensusClusterPlus. (B) Comparison of the chemotherapeutic drugs, including cisplatin and vinblastine, to which the high-score group and Cluster 1 were more sensitive. (C) Comparison of the immunotherapeutic drugs, including lenalidomide and rapamycin, to which the high-score group and Cluster 1 were more sensitive. (D) Comparison of the targeted drugs to which the high-score group and Cluster 1 were more sensitive, including bexarotene, dasatinib, gefitinib, imatinib, lapatinib and pazopanib. (E) Comparison of the chemotherapeutic drugs, including epothilone B and mitomycin C, to which the low-score group and Cluster 2 were more sensitive. (F) Comparison of the targeted drugs, including sorafenib, to which the low-score group and Cluster 2 were more sensitive.
