# Supplementary figures and images for: Construction and validation of a necroptosis-related lncRNA signature for predicting the prognosis of gastrointestinal cancer patients
Source: Front Immunol. 2025 Aug 14;16:1591252. doi: 10.3389/fimmu.2025.1591252 (PMC12391139; doi:10.3389/fimmu.2025.1591252)

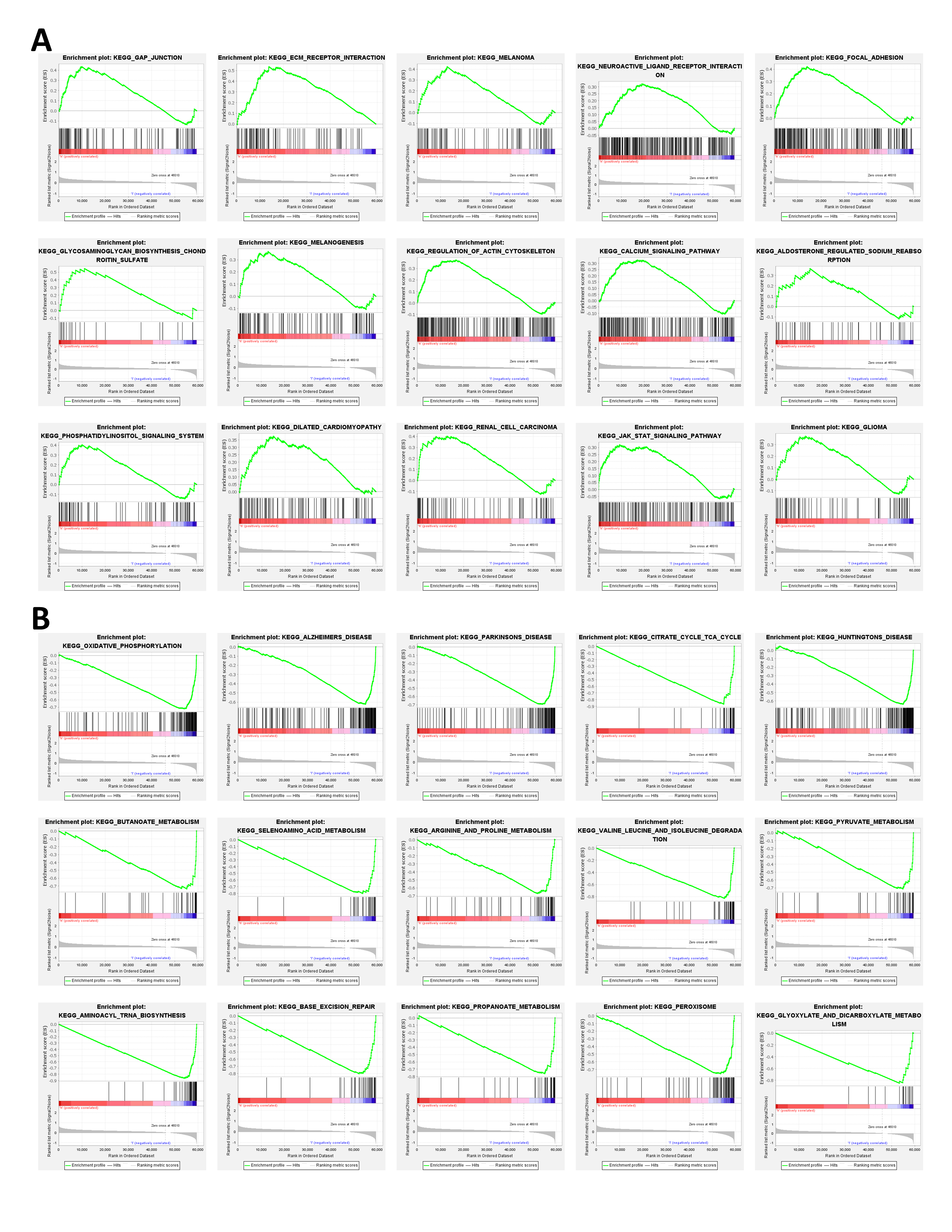

Supplement: Supplementary file 4 [file Image1.tif]

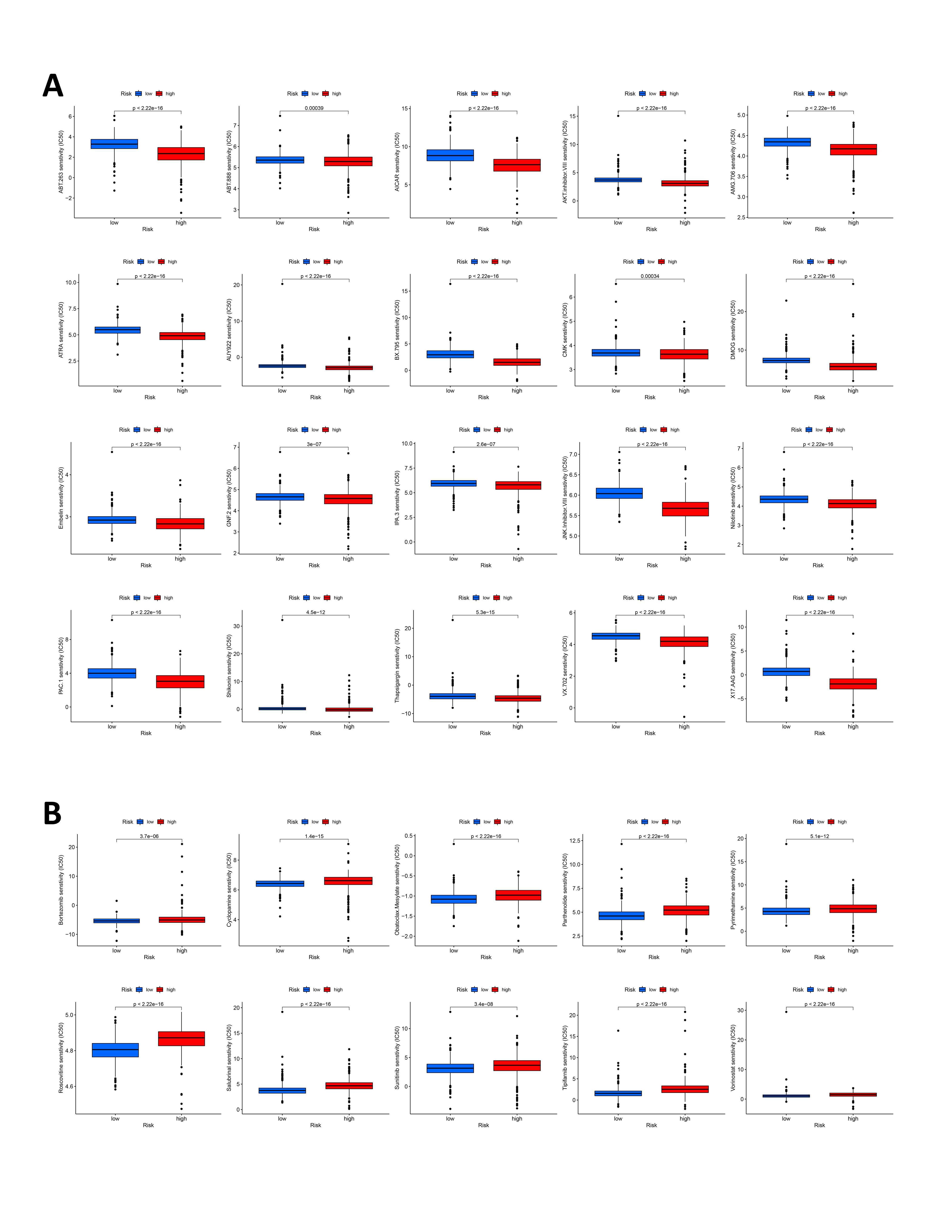

Supplement: Supplementary file 5 [file Image2.tif]

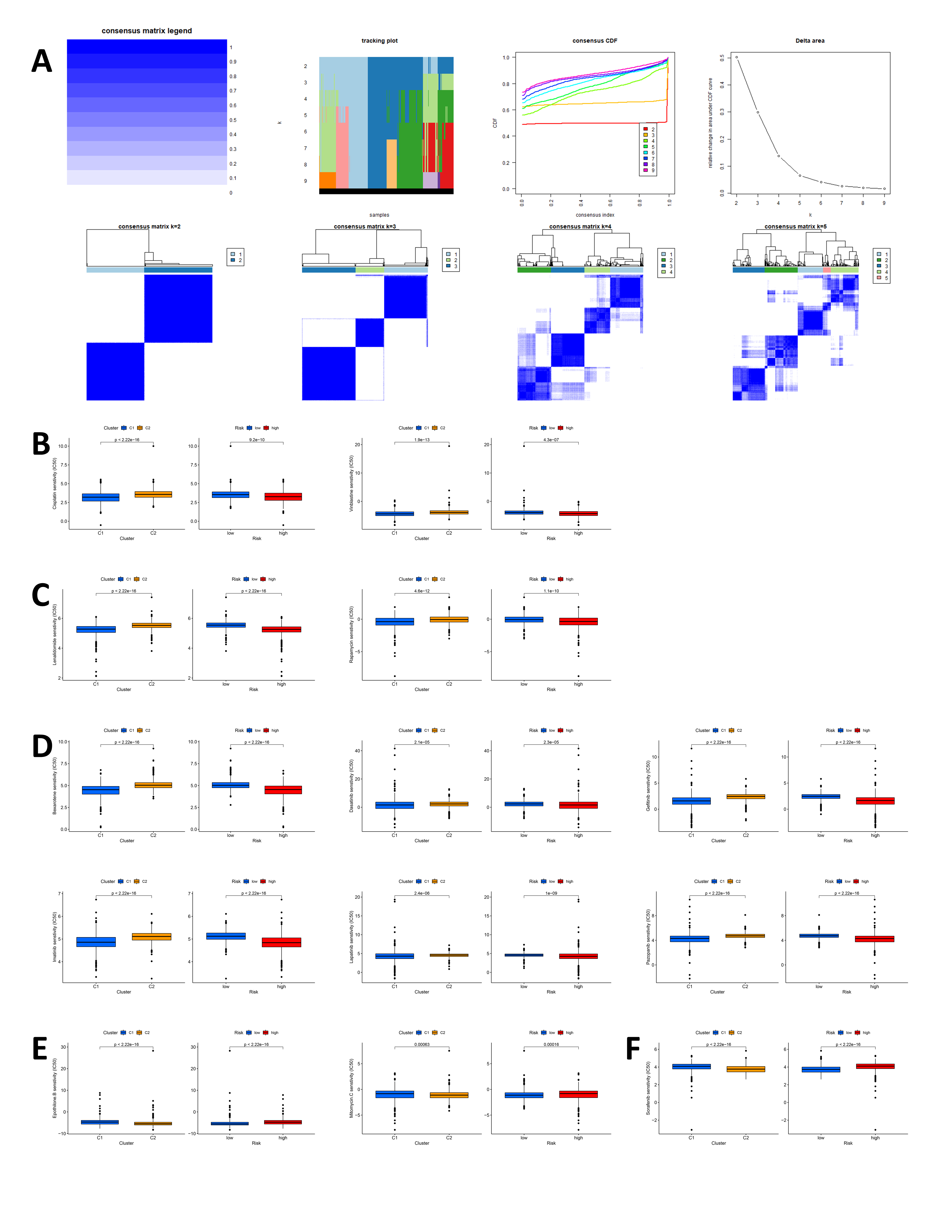

Supplement: Supplementary file 6 [file Image3.tif]
